# Composite selection signal analysis: Uncovering candidate genes and quantitative trait loci in Indian sheep breeds

**DOI:** 10.1371/journal.pone.0344299

**Published:** 2026-03-25

**Authors:** Sapna Nath, Satish Kumar Illa, Destaw Worku, Sabyasachi Mukherjee, Anupama Mukherjee, Vinod Kumar Yata

**Affiliations:** 1 College of Veterinary Science, Garividi, Sri Venkateswara Veterinary University, Andhra Pradesh, India; 2 Livestock Research Station, Garividi, Sri Venkateswara Veterinary University, Andhra Pradesh, India; 3 Department of Animal Sciences, College of Agriculture and Environmental Sciences, Bahir Dar University, Bahir Dar, Ethiopia; 4 Division of Animal Genetics and Breeding, Indian Council of Agricultural Research-NationalDairy Research Institute, Karnal, Haryana, India; 5 Department of Pharmacology, School of Allied and Healthcare Sciences, Malla Reddy University, Hyderabad, Telangana, India; Ain Shams University Faculty of Agriculture, EGYPT

## Abstract

Selective pressures, both natural and artificial, have significantly influenced the genomic architecture of domesticated sheep. Understanding their underlying molecular mechanisms is critical for developing efficient breeding programs to conserve and improve economically important traits in native breeds. In this study, we analysed high-density 50K SNP data from three Indigenous sheep breeds: Chanthangi (CHA, n = 29), Garole (GAR, n = 24), and Deccani (IDC, n = 26), each native to diverse climatic regions of India. We implemented a novel SNP-based de-correlated composite of multiple signals (DCMS) statistic, which integrates *p-*values from five selection metrics viz., FST, H1, H12, Tajima’s D, and nucleotide diversity (π) into a unified measure. The SNP-based DCMS approach offers finer resolution and complements window-based methods by enabling more precise localisation of selection signals and candidate genes. Multiple testing correction was applied at a False Discovery Rate (FDR) threshold of <5% to detect significant genomic regions**.** Comprehensive gene and quantitative trait loci (QTL) annotation and enrichment analysis of these regions were also performed for each breed. The DCMS analysis identified 21, 10, and 14 novel and breed-specific putative genes in the Chanthangi, Garole, and Deccani breeds, respectively, as well as 10, 28, and 13breed-specific QTL regions. The identified genes and QTLs are associated with diverse phenotypic traits, including growth and muscle development (*CNTNAP5, DOCK3*), reproduction (*TCERG1L, BUB1, UNC5C, C2CD5, BBX*), wool trait (*TPPP3, P2RY6*, *FGF10, POU2F1*, *FAM168A*), disease resistance (*MTSS1, B4GALNT3*), environment adaptation (*TRMT12, MAPKAPK3*), domestication (*LRRC36*). The QTLs identified are associated with body conformation (body measurements and bone area), production (milk fat yield), reproduction (total lambs born), disease resistance (hemonchus resistance, foot rot, and pneumonia susceptibility), and health (platelet count and entropion). Our SNP-based DCMS method enabled high-resolution detection of breed-specific selection signatures. It facilitated the discovery of both known and novel genomic regions, candidate genes, and QTLs unique to Indian sheep breeds. This comprehensive approach provides valuable insights into the molecular mechanisms underlying economically important traits and offers a robust foundation for targeted genetic improvement and conservation of indigenous sheep breeds.

## 1. Introduction

Natural selection enhances the fitness of individuals, thereby increasing the frequency of beneficial mutations in a population [1]. Selection pressure leaves distinct patterns on the genome, known as selection signatures [2,3], which provide fundamental insights into the evolutionary forces shaping genetic diversity. Selection signatures also aid in QTL mapping of quantitative traits in domestic animals and in identifying advantageous mutations in livestock populations [4]. SNP genotyping arrays have revolutionized the study of genomic variation and are now a standard tool for detecting selection signatures in populations. These arrays consist of genetically stable single-nucleotide polymorphisms distributed across the genome, facilitating large-scale genetic analyses. Several methods have been developed to identify signatures of selection, including those based on allele frequency spectra, linkage disequilibrium, and population differentiation [5,6]. Such analyses have been applied to various sheep breeds worldwide [7–9]. To increase resolution and accuracy, composite methods, such as the composite of signals (CMS), have been proposed that combine information from multiple statistics to enhance the detection of selection signatures [10]. Methods like the integral haplotype score are effective for detecting incomplete or ongoing selection. In comparison, XPEHH identifies signals that are nearly fixed within one group but variable across the other. Consequently, most studies in farm animals now use complementary methods to improve signal resolution [5,11].

To further enhance accuracy and resolution, genomic regions under selection can be detected with greater accuracy and higher resolution using composite methods. These approaches, such as the decorrelated composite of multiple signals (DCMS), effectively combine signals from multiple single statistics while accounting for their correlations [12,13]. This study used a high-density SNP array, the Illumina OvineSNP50 BeadChip, widely used in sheep genomic research, which includes polymorphic SNPs from both Indian and global sheep breeds, to generate genomic data for the three sheep breeds. Such high-density SNP data is crucial for understanding the genetic landscape of Indian sheep [14].

India boasts a substantial sheep population of 74.44 million, comprising 44 registered breeds, according to the 20^th^ Livestock Census [15]. These breeds exhibit significant genetic diversity and adaptability to a variety of ecological environments, from the cold, dry regions of the Western Himalayas to the hot, dry areas of Rajasthan and nearby states, the semi-arid Deccan Plateau, and the sub-humid regions of West Bengal and Odisha. Consequently, the observed morphological, physiological, and behavioral variations among these breeds are directly due to differences in the environments in which they were raised, reflecting their specific adaptations. The present study focuses on elucidating the genomic basis of these adaptations in the three Indian sheep breeds: Changthangi, Garole, and Deccani. The Changthangi breed is well adapted to the cold, arid environments of the high-altitude Himalayan regions. The Garole breed, renowned for its high twinning rate, is found in the hot, humid conditions of West Bengal. The Deccani breed is native to the arid regions of the Deccan Plateau and is characterized by a black coat and coarse wool [16].

Genomics can help identify variants associated with productivity, disease resistance, and adaptability to diverse environmental conditions, enabling more informed selection and conservation decisions. Genomic selection programs can optimise traits such as growth rate, wool quality, and reproductive efficiency while preserving the unique characteristics of native sheep. However, despite the rich phenotypic diversity observed in Indian sheep, most genomic studies have focused on either a single statistic or broad, window-based comparative frameworks, often overlooking the fine-scale selection signatures unique to indigenous breeds [17–20].

To address these gaps, the present study applies a novel SNP-based DCMS approach, integrating five univariate statistics (FST, H1, H12, Tajima’s D, and nucleotide diversity π) for high-resolution detection of selection signatures in three indigenous Indian sheep breeds: Changthangi, Deccani, and Garole. Unlike previous window-based or cross-population studies, our SNP-level strategy enables precise localization of candidate genes and QTLs under selection, facilitating the identification of both known and novel trait-associated loci. Additionally, we perform comprehensive breed-specific gene and QTL annotation and enrichment analysis, linking genomic regions under selection to economically important traits and providing actionable insights for genetic improvement and conservation of Indian sheep.

## 2. Materials and methods

### 2.1. Ethics statement

The research did not involve human or animal participants, and all data examined were publicly available. Therefore, ethical approval or consent was not necessary.

### 2.2. Animal selection and genotyping

In the present study, the high-density SNP genotypes of three Indian sheep breeds, i.e., Changthangi (CHA, n = 29), Deccani (IDC, n = 24), and Garole (GAR, n = 26), were obtained using the Illumina Ovine SNP50 BeadChip, which was sourced from the WIDDE (Web-Interfaced next generation Database dedicated to genetic Diversity Exploration) [21]. This database is designed to store and manage high-density genotyping datasets for bovine and ovine species. The Illumina Ovine SNP50 BeadChip consists of 54,241 SNPs distributed across the sheep genome, with an average density of one SNP every 51 kb. Quality control (QC) of the genotypes was performed based on the following criteria: minor allele frequency (maf < 0.05), Hardy-Weinberg equilibrium (HWE p < 0.0001), individuals with missing genotypes (--mind < 0.1), and SNPs with missing genotype data (geno < 0.1) [20]. A total of 41,997 SNPs were retained for further analysis. These QC criteria were applied to eliminate low-quality SNPs and to advance to subsequent phases of the analysis. Our study employed stringent SNP-level quality control criteria to ensure robust and accurate variant calls. SNPs with unknown genomic coordinates or located on sex chromosomes were excluded, restricting analyses to well-mapped autosomal loci**.** The SNPs with precise coordinates based on the OAR (*Ovis aries* Reference) Rambouillet reference genome assembly were included for analysis.

### 2.3. Principal component analysis

Principal component analysis (PCA) was performed on SNP genotypes from the studied sheep breeds to investigate the structure and clustering of individuals in the dataset. PCA categorized individuals into distinct clusters, and selection signatures were then analyzed within each cluster. The PCA analysis was performed in R using the *snprelate* package [22].

### 2.4. De-correlated composite of multiple selection signals

In this study, we computed the DCMS statistic by combining five univariate statistics: FST, H1, H12, Tajima’s D, and nucleotide diversity π [23–27].

The computation of the DCMS statistic for a locus l is given below


$$\begin{array}{c} {DCMS}_{l\;}=\;\sum {\mathrm{\backslash nolimits}}_{t=1}^{n}\frac{\text{log}\left\lfloor \frac{1-P_{lt}}{P_{lt}}\right\rfloor }{\sum_{i=1}^{n}\left|r_{it}\right|} \end{array}$$ (1)

The DCMS statistic combined the *p-*values of individual statistics and their correlations to improve the sensitivity and robustness of detecting selection signals. The DCMS statistic was composed of two components. The numerator component included *P*_*lt*_, which indicated the *p-*value at a given locus ‘*l*’ of each univariate statistic ‘*t*’. The denominator contained the correlation parameter ‘*r*_*it*_’, which accounted for the covariance structure among all the included univariate statistics [13].

### 2.5. Univariate statistics in DCMS

#### 2.5.1. Fixation index (F_ST_).

The F_ST_ index denoted the degree of differentiation between the populations. The functions in *PLINKV1.9*, such as –-fst and –-within, were used to determine the FST values for each SNP, and the negative FST values were changed to zeros. Later, the FST values were smoothed using the *runmed* function in R. The F_ST_ values for each breed were estimated.

#### 2.5.2. Haplotype homozygosity statistics (H1 and H12).

Haplotype homozygosity assesses the conservation of haplotypes and can help identify regions under selective pressure. The haplotypes in each data set were determined using the SHAPEIT2 program [28]. The conditional states parameter was kept at its default value for haplotype phasing. The *SNePv1.1* program computed the effective population size for each breed, which was then used to phase the chromosomes [29]. A high-resolution Ovine recombination map was used to correct for recombination rate variation across all autosomes [30]. An R script was used to convert the phased haplotypes into the input files required by the H12_H1H2.py program. The window size of SNPs was kept to 14, and the step size was 1 (-window 14 -jump 1) [26].

#### 2.5.3. Tajima’s D and nucleotide diversity (π).

Tajima’s D detects departures from neutrality, with negative values indicating potential selective sweeps, while nucleotide diversity (π) measures the genetic variation within a population. The statistics Tajima’s D and (π) at each coordinate in the dataset of all the studied breeds were computed using the *vcftools* program [31], and the estimates were computed for each chromosome separately. A 300 MB nonoverlapping sliding window (-Tajima 300) was used to compute Tajima’s D index. Furthermore, *p*-values were assigned to SNPs within the bin. Meanwhile, the –site-pi option in *vcftools* is used to compute the π values for each SNP. The *runmed* function smoothed the *p-*values as outlined in [27].

#### 2.5.4. DCMS estimation.

The DCMS statistic was computed by integrating the *p*-values from five univariate selection statistics (FST, H1, H12, Tajima’s D, and nucleotide diversity π) using the the stat_to_*p*-value function within the *MINOTAUR* R package, based on fractional ranks [32]. To account for correlations among these individual statistics, a correlation matrix of order n x n was computed using the *covNAMcd* function from the *rrcovNA* R package (alpha = 0.75, nsamp = 50,000) [33]. This correlation matrix served as input for DCMS estimation, and the resulting DCMS values were normalized using the rlm function from the *MASS* R package [34], as described in Yurchenko et al. (2018) [27]. This approach yields a composite signal that accounts for the covariance structure among the individual statistics, thereby improving the robustness of selection signal detection [13]. To reduce the error rate from multiple tests [35]. The false discovery rate (FDR) was controlled using *q*-values transformed from *p*-values, as described in [36], to identify significant genomic regions [36].

### 2.6. Identification of functional genes and QTL regions

In this genome-wide selection signature analysis, the *p*-value represents the probability of observing a test statistic at least as extreme as the observed value under the null hypothesis of no selection, with smaller values indicating stronger evidence of a selection signal. However, testing thousands of SNPs simultaneously inflates the risk of false positives, which is controlled by applying false discovery rate (FDR) correction to produce *q*-values—the expected proportion of false discoveries among significant results. A *q*-value threshold of 0.05 ensures that, on average, at most 5% of declared significant loci are false positives, providing a reliable, genome-wide-corrected interpretation of the selection signatures [36,37]. The putative genomic regions were identified using the *q*-value. A *q-*value threshold of 0.05 was chosen to identify genomic regions with strong evidence of selection, while adjacent SNPs with *q-*values greater than 0.1 were excluded to reduce false positives. The gene and QTL annotation of the candidate genomic regions was carried out using the Genomic Annotation in Livestock for positional candidate Loci (*GALLO*) package v4.2.0 [38]. The ‘.gtf’ file related to ARS-UI_Ramb_v2.0 of the sheep genome was accessed from NCBI, and the corresponding ‘.gff’ file, which belonged to the ARS-UI_Ramb_v2.0, was downloaded from the Sheep QTL database [39].

Candidate genes and quantitative trait loci (QTLs) located within the significant genomic regions are considered putative if they fall within an interval of 0.5 Mb upstream and downstream. Furthermore, QTL enrichment analysis was performed to identify overrepresented traits within the significant regions, using a genome-wide approach and a false discovery rate (FDR) threshold of 0.05.

## 3. Results

### 3.1. Quality control of genotypic data

The quality control of genotypic data included filtering SNPs with minor allele frequency (maf < 0.05), assessing Hardy-Weinberg equilibrium (hwe < 0.0001), and setting missingness thresholds for SNP genotypes (geno < 0.1) and individual genotypes (mind < 0.1). The SNPs excluded for maf, hwe, geno, and mind were 3845, 299, 678, and 0, respectively. Furthermore, SNPs with unknown coordinates (n = 1232) on chromosomes other than the autosomes were removed. The final dataset contains 41997 variants for downstream analysis.

### 3.2. Principal component analysis (PCA)

**Fig 1:**
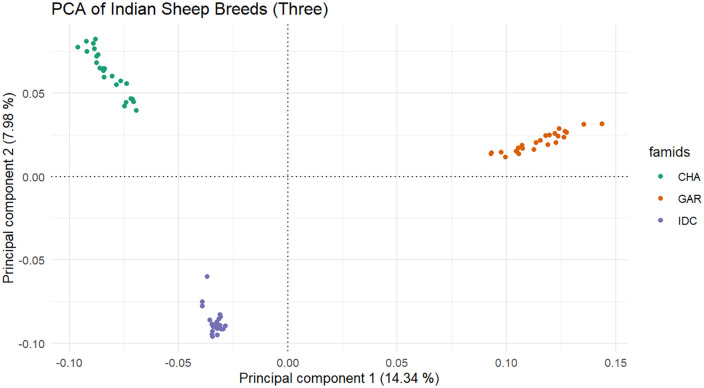
The PCA analysis showing the population structure of three Indian sheep breeds. The PCA plot reveals genetic differentiation and clustering among individuals of these breeds. The x-axis, representing Principal Component 1 (PC1), accounts for 14.34% of the total genetic variation and mainly distinguishes Garole from the Changthangi and Deccani breeds. An additional 7.98% of the variation is explained by Principal Component 2 (PC2) on the y-axis, further separating the Changthangi and Deccani breeds into two distinct clusters. This figure clearly demonstrates genetic stratification and unique ancestry in these breeds, reflecting their distinct evolutionary histories and adaptations to different environments**.**

Principal component analysis (PCA) was employed to ascertain distinct clusters of SNP genotypes among the three Indian sheep breeds under investigation. The results of PCA are presented in Fig 1. The PCA confirmed the genetic differentiation among the three breeds, as indicated by their clear separation into distinct clusters. PC1 accounted for 14.34% of the variation, highlighting the strongest genetic differences between the populations, while PC2 explained an additional 7.98% of the total variation, capturing finer distinctions within the populations.

### 3.3. De-correlated composite of multiple selection signals (DCMS)

The DCMS analysis identified 45 breed-specific regions under selection, with 21 in Chanthangi, 10 in Garole, and 14 in Deccani sheep genomes. The total size of these regions was 2871.54 Kb for Chanthangi, 2857.84 Kb for Garole, and 2513.30 Kb for Deccani. The average length of these regions was 136.74 ± 229.77 Kb in Chanthangi, 259.80 ± 310.04 Kb in Garole, and 167.55 ± 216.86 Kb in Deccani. The genome lengths covered ranged from 1.83 Kb to 1.08 Mb in Chanthangi, 34.11 Kb to 1080.32 Kb in Garole, and 2.20 Kb to 817.32 Kb in Deccani**.**

#### 3.3.1. Chanthangi sheep (gene annotation).

In the present study, the gene annotation of the significant genomic regions uncovered twenty-one potential candidate genes related to a variety of traits in this sheep, including *CNTNAP5* (body growth), *MTSS1* (host resistance to bacterial infections), *TRMT12* (immune system and environment adaptation), *TPPP3* (hair follicle growth), *LRRC36* (domestication and early development), *STARD10* (resilience to paratuberculosis), *P2RY6*, *FGF10* and *FAM168A* (hair follicle morphogenesis), *ARHGEF17* and *NIM1K* (meat quality)*, GHR* (litter size). These genes were located on OAR 2, 3, 9, 14, 15, and 26. A greater number of significant genomic regions were identified on OAR 15 (Table 1).

**Table 1:** Gene annotation in significant genomic regions in CHANTHANGI sheep.

CHR^*^	BP^*^	START COORDINATE	END COORDINATE	Width	Strand	Gene Name	*Pvalue*	*Q*value
2	190267950	189526649	190606970	1080322	+	*CNTNAP5*	*4.95e-05*	*0.032*
3	106221882	106222211	106279403	57193	+	*FBLN7*	*1.29e-05*	*0.015*
3	107958569	107917450	107989336	71887	+	*TBC1D15*	*1.28e-05*	*0.015*
9	28518494	28426199	28586681	160483	+	*MTSS1*	*0.008*	*0.048*
9	28610511	28596736	28628449	31714	+	*TATDN1*	*9.16e-05*	*0.045*
9	28651053	28650081	28651912	1832	–	*TRMT12*	*0.000*	*0.048*
9	28651053	28627373	28650325	22953	–	*RNF139*	*0.000*	*0.048*
14	35090205	35080140	35173351	93212	+	*NFATC3*	*4.59e-06*	*0.009*
14	34566935	34510632	34569231	58600	–	*TPPP3*	*7.71e-05*	*0.042*
14	34566935	34504860	34579222	74363	+	*LRRC36*	*7.71e-05*	*0.042*
15	51070453	51028830	51075998	47169	–	*STARD10*	*2.34e-08*	*0.000*
15	51107183	51099187	51353916	254730	–	*FCHSD2*	*2.34e-08*	*0.000*
15	51474825	51404925	51511918	106994	+	*P2RY2*	*1.39e-08*	*0.000*
15	51517148	51481919	51516379	34461	+	*P2RY6*	*1.39e-08*	*0.000*
15	51578641	51521915	51579398	57484	+	*ARHGEF17*	*6.12e-10*	*2.57e-05*
15	51591704	51585751	51604071	18321	+	*RELT*	*3.07e-09*	*6.44e-05*
15	51632422	51613275	51814944	201670	–	*FAM168A*	*1.19e-05*	*0.015*
16	30667706	30604422	30702581	98160	+	*FGF10*	*7.11e-06*	*0.010*
16	31588015	31575745	31651791	76047	–	*NIM1K*	*4.12e-05*	*0.031*
16	31687647	31660770	31688311	27542	–	*ZNF131*	*1.85e-05*	*0.018*
16	32196927	32069778	32366181	296404	–	*GHR*	*9.62e-07*	*0.003*

*CHR – Chromosome; *BP – Base Position.

#### 3.3.2. Quantitative trait loci (QTL) annotation and enrichment.

Table 2 presents the results of QTL annotation in Chanthangi sheep. Our study identified significant genomic regions annotated with 10 QTLs on CHR 2, 3, 14, 15, and 16.

**Table 2:** Quantitative trait loci annotation in significant genomic regions in CHANTHANGI sheep.

CHR^*^	BP^*^	START COORDINATE	END COORDINATE	Name	Flank Marker	*q*-value
2	190267950	190457084	190457088	Body weight	rs424714738	1.61E-03
3	107991182	108401221	108401225	Body weight	rs426980328	0.026
14	35090205	35417680	35417684	Body weight	rs417231209	<0.05
15	51070453	50955799	50955803	Milk fat yield	rs404288918	0.0006245
15	51474825	51832265	51832269	Platelet count	rs413264572	5.50E-06
16	31588015	32070073	32070077	Total lambs born	rs161146164	<0.05
16	31654423	32118461	32118465	Total lambs born	rs426666828	<0.05
16	32569758	32119256	32119260	Milk fat yield	rs422431427	0.00031338
16	32569758	32192693	32192697	Body weight	rs408124092	<0.0001
16	32569758	32192693	32192697	Soft tissue depth at GR site	rs408124092	0.0014

*CHR – Chromosome; *BP – Base Position.

**Fig 2:**
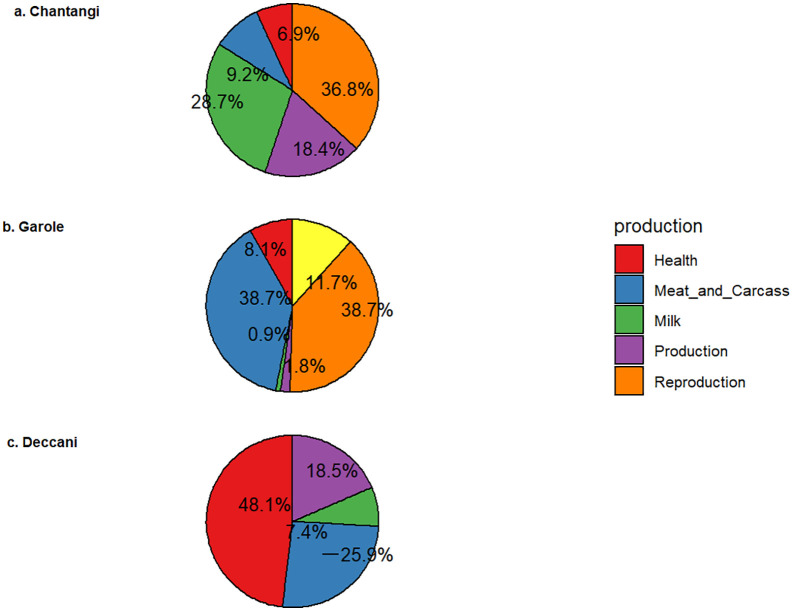
Percent QTL classes annotated in three Indian sheep breeds. This figure displays three pie charts (a, b, and **c)**, each depicting the percentage contributions of various trait categories to the total identified QTLs in a specific breed. The legend positioned on the right clarifies the trait classes represented by each color: Health (red), Meat and Carcass (blue), Milk (green), Production (purple), and Reproduction (orange). **a. Chanthangi:** The QTLs in Chanthangi sheep are primarily associated with **Reproduction (36.8%),** followed by Milk (28.7%), Production (18.4%), Meat and Carcass (9.2%), and Health (6.9%). **b. Garole:** In Garole sheep, the predominant QTL categories are **Meat and Carcass (38.7%) and Reproduction (38.7%),** with lesser contributions from Health (8.1%), Production (11.7%), and Milk (1.8%). **c. Deccani:** Deccani sheep exhibit a significant prevalence of QTLs associated with **health traits (48.1%),** followed by those related to production (18.5%). Additional categories include milk (7.4%) and meat and carcass traits, which are reported as −25.9%.

The annotated traits are known to be associated with body weight, milk fat yield, platelet count, total lambs born, and soft tissue depth at the GR site. The QTLs identified in each breed were categorized into various trait classes, including body weight, reproduction, milk yield, and health, and the enrichment of QTLs is shown in Fig 2. The highest percentage of QTLs annotated in the significant regions of Chanthangi were milk (37.5%), followed by reproduction (34.38%). The other classes of QTLs annotated were meat and carcass (9.38%), health (9.38%), and production (milk fat yield) (9.38%). The QTL enrichment analysis revealed the top significant (False Discovery Rate (FDR)-corrected *p*-value – 0.05) QTL total lambs born on OAR 16.

#### 3.3.3. Garole sheep (gene annotation).

In this study, several putative genes were identified in the significant genomic regions located on OAR 1, 2, 3, 6, 16, and 18 (Table 3).

**Table 3:** Gene annotation in significant genomic regions in GAROLE sheep.

CHR^*^	BP^*^	START COORDINATE	END COORDINATE	Width	Strand	Gene	*Pvalue*	*Qvalue*
1	173424721	173196985	173483458	286474	+	*BBX*	*2.01e-05*	*0.015*
2	189539157	189526649	190606970	1080322	+	*CNTNAP5*	*8.19e-05*	*0.042*
3	193287569	193251622	193339539	87918	+	*C2CD5*	*8.81e-07*	*0.003*
3	212967581	212887405	212973893	86489	+	*IQSEC3*	*6.47e-06*	*0.007*
3	213086338	213068112	213128627	60516	–	*KDM5A*	*1.07e-05*	*0.009*
3	213195386	213175874	213273093	97220	+	*B4GALNT3*	*7.64e-06*	*0.007*
6	29809690	29597358	30017600	420243	+	*UNC5C*	*6.21e-06*	*0.007*
16	65681259	65471620	65927572	455953	–	*ADCY2*	*7.56e-05*	*0.041*
18	46100361	46097255	46131370	34116	–	*CLEC14A*	*4.00e-05*	*0.024*
22	49602008	49599602	49793506	193905	–	*TCERG1L*	*6.19e-06*	*0.007*

*CHR – Chromosome; *BP – Base Position.

However, OAR 3 anchors more potential candidate genes in the GAR breed. In this study, we identified eleven putative genes associated with a wide range of characteristics, including *BBX* (teat number), *CNTNAP5* (body growth), *C2CD5* (fecundity), *KDM5A* (follicular development), *B4GALNT3* (paratuberculosis resistance), *UNC5C* (litter size), *ADCY2* and *TCERG1L* (reproduction).

#### 3.3.4. Quantitative trait loci (QTL) annotation and enrichment.

The present study presents the QTL annotation findings for the GAR breed, as shown in Table 4.

**Table 4:** Quantitative trait loci annotation in significant genomic regions in GAROLE sheep.

CHR^*^	BP^*^	START COORDINATE	END COORDINATE	Name	Flank Marker	*q*-value
1	173424721	173432597	173432601	Teat number	rs430157497	<0.05
1	173424721	173534798	173534802	Teat number	rs426598066	<0.05
2	190019411	190457084	190457088	Body weight	rs424714738	1.61E-3
3	192453314	192442586	192442590	Entropion	rs411640492	3.19E-04
3	192935483	192442586	192442590	Entropion	rs411640492	3.19E-04
3	193336800	193016493	193016497	Entropion	rs404605277	1.12E-04
3	212967581	213021781	213021785	Entropion	rs159913124	3.61E-04
3	213209196	213213768	213213772	Entropion	rs418752307	2.81E-04
6	29809690	29344134	29344138	Bone area	rs418089550	1.12E-5
6	29809690	29366036	29366040	Milk fat yield	rs424558688	0.036982472
6	29847992	30096782	30096786	Total lambs born	rs421635584	<0.05
18	46159259	46086980	46086984	Body weight	rs426187704	<0.05
18	46100361	46086980	46086984	Body depth	rs426187704	<0.01
18	46062053	46086980	46086984	Body weight	rs426187704	<0.05
18	46062053	46086980	46086984	Body length	rs426187704	<0.05
18	46159259	46086980	46086984	Body circumference	rs426187704	<0.05
18	46159259	46086980	46086984	Body depth	rs426187704	<0.01
18	46159259	46086980	46086984	Rump width	rs426187704	<0.05
18	46159259	46087004	46087008	Body weight	rs404696179	<0.05
18	46159259	46087004	46087008	Body length	rs404696179	<0.01
18	46100361	46087004	46087008	Body height	rs404696179	<0.01
18	46159259	46087004	46087008	Body circumference	rs404696179	<0.05
18	46062053	46087004	46087008	Body height	rs404696179	<0.05
18	46159259	46087646	46087650	Chest width	rs405457403	<0.05
18	46159259	46087646	46087650	Shin circumference	rs405457403	<0.05
18	46159259	46087646	46087650	Body depth	rs405457403	<0.05
18	47232764	47716852	47716856	Haemonchus contortus resistance	rs428856771	3.23E-4
22	49796078	49873766	49873770	Lambs born alive	rs402605786	9.79E-3

*CHR – Chromosome; *BP – Base Position.

Our study revealed significant genomic regions annotated with 28 QTLs on CHR 1, 2, 3, 6, 18, and 22. The annotated traits are known to be associated with teat number, body weight, milk fat yield, entropion, bone area, total lambs born, body depth, body length, body circumference, shin circumference, body weight, body height, chest width, rump width, lambs born alive and hemonchus contortus resistance. Furthermore, the annotated QTLs are categorised into different classes, and the enrichment of QTLs is depicted in Fig 2A. Garole’s most significant genomic regions had the greatest proportion of QTLs associated with health (38.74%) and production (38.74%). The remaining percentage of QTL classes annotated in the breed were reproduction (11.71%), exterior (8.11%), milk (1.8%) and meat and carcass (0.9%). An analysis of QTL enrichment revealed the three most significant QTLs (False Discovery Rate (FDR)-corrected *p*-value < 0.05) associated with total lambs born, teat number, and entropion on CHR 6, 1, and 3.

#### 3.3.5. Deccani sheep (gene annotation).

The gene annotation findings are summarised in Table 5.

**Table 5:** Gene annotation in significant genomic regions in DECCANI sheep.

CHR^*^	BP*	START COORDINATE	END COORDINATE	width	strand	Gene	*p-value*	*q-*value
1	119053600	119027142	119081828	54687	+	*MAEL*	*9.08e-09*	*4.24e-05*
1	131092601	130838877	131151750	312874	+	*APP*	*3.55e-07*	*0.000*
1	119491972	119428361	119624526	196166	+	*POU2F1*	*1.33e-05*	*0.007*
3	104706333	104671436	104716760	45325	–	*MALL*	*9.21e-13*	*1.29e-08*
3	104775041	104716342	104784266	67925	–	*NPHP1*	*9.21e-13*	*1.29e-08*
3	104885280	104870169	104915494	45326	–	*BUB1*	*1.00e-13*	*4.20e-09*
3	212967581	212887405	212973893	86489	+	*IQSEC3*	*0.000*	*0.044*
3	104917562	104914948	105286674	371727	+	*ACOXL*	*1.64e-11*	*1.15e-07*
4	101967194	101379345	102196659	817315	+	*CHRM2*	*1.64e-11*	*1.15e-07*
19	49657211	49447036	49738575	291540	–	*DOCK3*	*6.78e-06*	*0.005*
19	49778830	49759229	49789524	30296	–	*MAPKAPK3*	*5.45e-06*	*0.004*
2	52731947	52731991	52734550	2560	+	*HINT2*	*5.26e-07*	*0.001*
2	52790093	52790313	52801134	10822	–	*RGP1*	*5.26e-07 0.001*	*0.001*
2	52790093	52790904	52793100	2197	+	*MSMP*	*5.26e-07 0.001*	*0.001*

*CHR – Chromosome; *BP – Base Position.

The gene annotation of the significant genomic regions revealed fourteen potential candidate genes related to a variety of traits in this sheep, including *BUB1* (oocyte and follicle development), *POU2F1* (wool colour), *CHRM2* (hair follicle growth), *DOCK3* (muscle development), and *MAPKAPK3* (heat stress response). These genomic regions harbour candidate genes associated with various traits and are located on CHR 1, 2, 3, 4, and 19, with the largest number of significant genomic regions on CHR 19.

#### 3.3.6. Quantitative trait loci (QTL) annotation and enrichment.

The findings outlined in Table 6 detail the results of QTL annotation in Deccani sheep.

**Table 6:** Shows the quantitative trait loci annotation results in significant genomic regions in DECCANI sheep.

CHR^*^	BP^*^	start_pos	end_pos	Name	FlankMarker	*q*-value
1	131092601	130616460	130616464	somatic cell count	rs409795528	<0.05
3	212967581	213021781	213021785	Entropion	rs159913124	3.61E-04
3	212967581	213213768	213213772	Entropion	rs418752307	2.81E-04
4	101967194	101960285	101960289	Monocyte number	rs399619443	2.55E-6
4	101967194	102026928	102026932	Footrot susceptibility	rs416121047	2.84E-6
4	101967194	102039511	102039515	Pneumonia susceptibility	rs403394816	<0.10
21	41665172	41243033	41243037	Body length	rs406947061	<0.05
2	52731947	52720586	52720590	Body weight	rs426272889	<0.05
2	52790093	52737532	52737536	Body weight	rs160159557	<0.05
2	52790093	52848103	52848107	Water holding capacity	rs420647640	<0.05
2	116594826	116133033	116133037	Horn type	rs416045857	<0.05
2	116594826	116839657	116839661	Horn type	rs405044765	<0.05
2	116953479	116872298	116872302	Horn type	rs402400571	<0.05

*CHR – Chromosome; *BP – Base Position.

**Fig 3:**
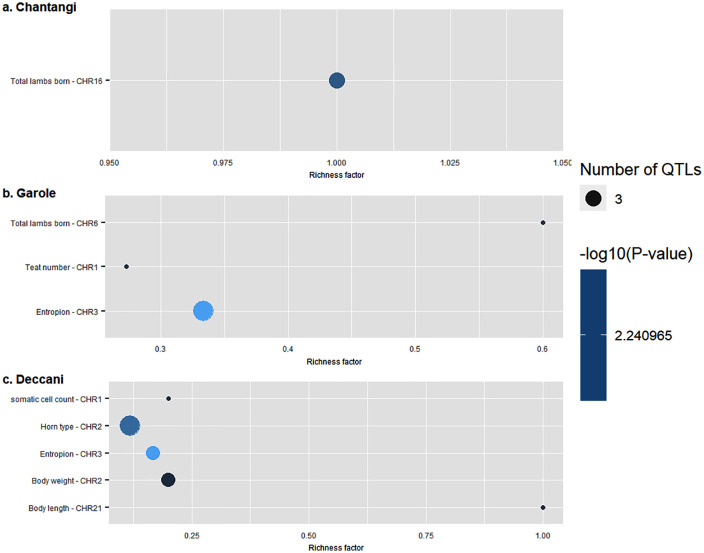
QTL classes enriched in three Indian sheep breeds. This figure shows three scatter plots (a, b, and c) illustrating enriched QTL classes in genomic regions selected for a specific breed. The y-axis lists enriched trait categories and their corresponding chromosomes. The x-axis displays the ‘Richness factor,’ which indicates the level of enrichment for each QTL class. Each bubble on the plot represents an enriched QTL class. The size of each bubble corresponds to the ‘Number of QTLs’ identified for that trait, and the color reflects the statistical significance of the enrichment, measured by -log10(*p*-value). **a. Chanthangi**: Shows enrichment for ‘Total lambs born’ on Chromosome 16 (CHR16). **b. Garole**: Shows enrichment for ‘Total lambs born’ on CHR6, ‘Teat number’ on CHR1, and ‘Entropion’ on CHR3. **c. Deccani**: Indicates enrichment for ‘Somatic cell count’ on CHR1, ‘Horn type’ on CHR2, ‘Entropion’ on CHR3, ‘Body weight’ on CHR2, and ‘Body length’ on CHR21.

**Fig 4:**
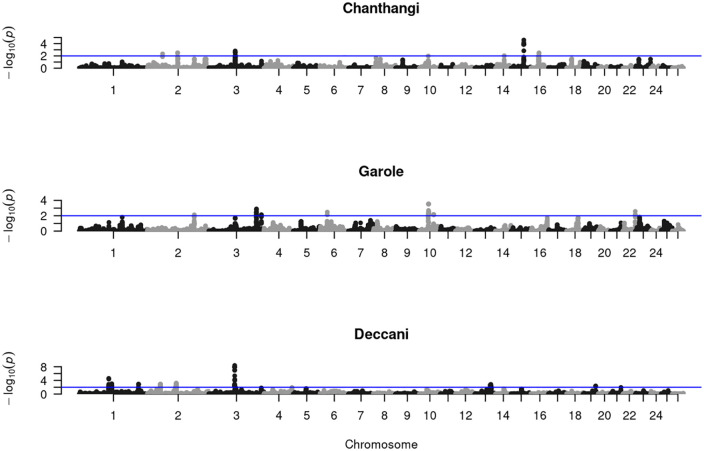
Manhattan plot showing signatures of selection in three Indian sheep breeds. This figure includes three separate Manhattan plots, each depicting a sheep breed: a) Chanthangi, b) Garole, and c) Deccani. Each plot shows the genomic regions under selection pressure. The x-axis of all plots denotes the chromosomal position, with individual chromosomes (1 to 26) arranged sequentially and distinguished by alternating grey and black hues. Whereas, the y-axis of the Chanthangi and Garole plots ranges from 0 to 4, and in the Deccani plot, the y-axis scale extends from 0 to 8, which shows the negative base-10 logarithm of the *p-*value (-log10(*p-*value)), derived from the DCMS statistic. The horizontal blue line in each plot indicates the genome-wide significance threshold; SNPs clustering above this threshold highlights genomic regions that have undergone significant selection sweeps. Chanthangi shows prominent selection signals on chromosome 15, Garole on chromosomes 3 and 10, and Deccani on chromosome 3.

The study has identified significant genomic regions annotated with 13 QTLs on CHR 1, 2, 3, 4, and 21. The QTLs are associated with the following traits: somatic cell count, Entropion, Monocyte number, Footrot susceptibility, Pneumonia susceptibility, Body length, Body weight, water-holding capacity, and horn type. Furthermore, the annotated QTLs are grouped into various categories, and their distribution is illustrated in Fig 2. The regions with the most significant impact on the evolution of the Deccani breed had the highest percentage of QTLs annotated for exterior traits (48.15%), followed by health traits (25.93%). The other classes of QTLs annotated were production (18.52%), and meat and carcass (7.41%). The QTL enrichment analysis identified the top-most significant QTLs (False Discovery Rate (FDR)-corrected *p*-value <0.05) on CHR 1,2, and 3, controlling traits such as somatic cell count, horn type, Entropion, Body weight, and Body length (Fig 3). Furthermore, the signatures of selection in the genomes of the studied breeds are shown in Fig 4.

In addition, the summary of putative genes is presented in Table 7.

**Table 7:** Summary of putative genes identified in Indian sheep.

CHR^*^	BP^*^	START COORDINATE	END COORDINATE	Gene	Trait
2	189539157	189526649	190606970	*CNTNAP5*	Growth and muscle development
3	193287569	193251622	193339539	*C2CD5*	Reproduction
9	28518494	28426199	28586681	*MTSS1*	Disease resistance
3	213195386	213175874	213273093	*B4GALNT3*	Disease resistance
9	28651053	28650081	28651912	*TRMT12*	Adaptation
3	104885280	104870169	104915494	*BUB1*	Reproduction
6	29809690	29597358	30017600	*UNC5C*	Reproduction
14	34566935	34504860	34579222	*LRRC36*	Domestication
19	49657211	49447036	49738575	*DOCK3*	Growth and muscle development
19	49778830	49759229	49789524	*MAPKAPK3*	Adaptation
15	51517148	51481919	51516379	*P2RY6*	Wool trait
16	30667706	30604422	30702581	*FGF10*	Wool trait

## 4. Discussion

This study used an SNP-based, decorrelated composite of multiple signals (DCMS) to identify the genetic basis of important economic and adaptation traits that influence the genomic landscape in three indigenous Indian sheep breeds: Chanthangi, Garole, and Deccani. It uncovered novel, breed-specific genomic regions, genes, and quantitative trait loci (QTLs) under selection in these breeds. The identified regions provided key insights into the molecular mechanisms responsible for traits such as growth, reproduction, wool production, disease resistance, and environmental adaptation. This research offers a strong foundation for developing genetic improvement strategies and conservation programs for these valuable indigenous breeds.

### 4.1. Principal component analysis (PCA)

Principal Component Analysis (PCA) demonstrated distinct genetic differentiation and clustering patterns among the studied Indian sheep breeds (Fig 1). This apparent population stratification, however, was inconsistent with previous findings by Ahmad et al. (2021) [40]. who reported a lack of distinct clustering among the breeds they investigated based on the first principal component. This divergence in results may be attributed to Ahmad et al.‘s inclusion of a broader range of breeds, which likely introduced additional genetic variation into their analysis.

### 4.2. Chanthangi sheep

#### 4.2.1. Gene annotation.

India’s diverse sheep production systems are adapted to a wide spectrum of climates, ranging from the cold Northern Himalayan foothills to the hot and arid Central Peninsular region and the humid Eastern region. Reflecting these varied environmental pressures, our findings indicated that none of the identified candidate genes within the detected genomic regions showed significant selection signatures consistently across all three analyzed breeds. Nonetheless, the identified genomic regions harbored potential candidate genes associated with a broad spectrum of traits, including body growth, reproduction, fecundity, heat-stress response, host resistance to bacterial infections, domestication, environmental adaptation, hair follicle growth, meat quality, and litter size. The minimal overlap observed in significant genomic regions among the breeds strongly suggests the prevalence of breed-specific selection processes driven by local adaptations throughout their evolutionary history.

The present study identified *CNTNAP5* (Contactin-associated protein-like 5B) as a significant genomic region located on CHR 2, and as a protein-coding gene. This gene belongs to the neurexin family and plays an important role as a cell adhesion molecule and receptor in the nervous systems of most vertebrates. The mechanism of action of *CNTNAP5* is similar to that of the brain-derived neurotrophic factor (*BDNF*) gene, which influences body mass index. Like other epidermal growth factors, it potentially influences growth by enhancing the functions of the brain that regulate growth [41,42]. Genomic investigations of morphological traits in Sudanese goats revealed that the *CNTNAP5* gene, located on chromosome 2, was associated with the bicostal diameter [43].

The indigenous sheep of India are known for resistance to infectious agents. Our findings also identified specific genomic regions in Chanthangi sheep that harbor the *MTSS1* gene on chromosome CHR9, which is associated with resistance to bacterial infections. These findings were consistent with the breed characteristics of Chanthangi. The bacteria’s endotoxins disrupt the endothelial lining in the host cell and cause infection. However, the *MTSS1* gene alters the host endothelium and improves its integrity, thereby preventing infections [44]. The DCMS analysis also revealed significant genomic regions containing the *STARD10* gene, which is reportedly involved in resistance to paratuberculosis [45]. It regulates bile acid metabolism by modulating the PPARα-mediated pathway [46]. The study identified an essential candidate gene, *LRRC36*, associated with domestication in sheep. *LRRC36* is a leucine rich repeat containing 36 gene differentially expressed in testis and lung tissues. Studies on the structural variant landscape in sheep and goats revealed that the *LRRC36* gene was involved in the early domestication [47]. Another candidate gene, *TRMT12*, a tRNA methyltransferase, is fundamental for tRNA modification, which is vital for regulating mRNA translation and protein homeostasis. Although direct studies on its role in environmental adaptation in sheep are currently absent, its molecular function suggests a plausible contribution to cellular resilience against diverse stressors [48,49]. By ensuring efficient protein synthesis, *TRMT12* could mediate cellular adaptation under challenging environmental conditions, making it a compelling candidate gene in indigenous sheep breeds.

Chanthangi sheep are primarily reared by the Changpa, a nomadic tribe of the Northern hills, along with Chanthangi goats. This dual-purpose sheep breed, maintained for both wool and mutton, contributes significantly to the livelihoods of nomadic tribes. Shepherd’s income mainly derives from selling wool and meat. Our study highlighted critical candidate genes associated with hair follicle development, including *P2RY6* (OAR 15) and *FGF10* (OAR 16). A gene expression study in mice demonstrated the role of *P2RY6* in controlling the *MEK1/2*‒b-catenin signalling pathways and the *MST‒LATS1‒YAP* signal cascades, indicating its function in keratinocyte growth and development [50]. Moreover, a gene expression study on coarse sheep fetal skin showed that *FGF10* was involved in hair follicle development via competitive adsorption of miR-184, which produces *FGF10* from chi-circRNA-0001141 [51].

We also identified genes under selection in Chanthangi sheep, including *ARHGEF17* and *NIM1K,* which are known to be related to meat quality. Weighted single-step genome-wide association studies (GWAS) revealed a significant association between the *ARHGEF17* gene and meat traits in the Chinese yellow-feathered chicken population [52]. *ARHGEF17* is a key component of actin cytoskeletal organization and enhances guanine nucleotide exchange factor activity. Transcriptomic and epigenomic studies in the pectoral muscles of chicken embryos demonstrated *NIM1K’*s significant role in muscle fibre formation. *NIM1K* enables ATP binding, magnesium ion binding, and protein serine/threonine kinase activity and is involved in protein phosphorylation [53]. Notably, several of these genes, such as *CNTNAP5, DOCK3*, *TCERG1L, BUB1, UNC5C, C2CD5, BBX, TPPP3, FGF10, POU2F1*, *FAM168A*, *MTSS1, B4GALNT3*, *TRMT12, MAPKAPK3,* and *LRRC36,* were not detected in earlier window-based DCMS analyses [20]**.** This highlights the advantage of SNP-level resolution for mapping breed-specific loci that are potentially overlooked by broader window-based approaches.

#### 4.2.2. Quantitative trait loci (QTL) annotation and enrichment.

The DCMS analysis revealed important QTL regions associated with body weight on OAR 2, 3, 14, and 16. The flanking markers or SNPs in the QTL regions, include rs424714738, rs426980328, rs417231209, and rs408124092, respectively. A genome-wide association study in Australian Merino sheep identified a significant QTL region on OAR2 associated with body weight. The study also found an orthologous region on OAR6, similar to human and bovine genomic regions associated with height and weight [54]. Our study also identified the same QTL as significant across the studied breeds. Zhang et al., 2016 identified *MEF2B* and *TRHDE* gene polymorphisms linked to body weight at 4 months of age in New Ujumqin Sheep on OAR 3 [55]. DCMS analysis revealed the same QTL region under selection in Indian populations. In Baluchi sheep, GWAS confirmed the QTL peak associated with 8-month body weight [56]. We identified a QTL on OAR 14 in Chanthangi sheep. Armstrong et al., (2018) investigated genetic polymorphisms in the *GHR* gene and demonstrated associations with carcass traits in grazing Texel sheep; the same QTL peak was observed in the Chanthangi sheep [57].

We also identified QTL related to platelet count and milk yield on OAR15 in Chanthangi sheep. Gonzalez et al., (2013) reported QTL association with mean corpuscular haemoglobin concentration (MCHC, P = 6.2 × 10 − 14) and decreased mean corpuscular volume in Columbia, Polypay, and Rambouillet sheep [58]. DCMS analyses also identified platelet count QTL in significant genomic regions of Chanthangi sheep. Genomic investigation in the crossbred dairy sheep population revealed milk yield QTL regions [59]. We found the same QTL regions in Chanthangi.

### 4.3. Garole sheep

The Garole sheep were renowned for their petite stature, remarkable fertility, superior meat quality, and ability to thrive in the saline marshlands of the hot, humid Sundarban region in West Bengal. This breed was also found across southern Bangladesh. Its small size resulted from adaptation to a harsh climate. Although genetic potential was limited, these sheep could be raised with minimal input management (Pan & Sahoo, 2008) [60]. The breed was highly valued for its fecundity-related genes.

#### 4.3.1. Gene annotation.

The gene annotation revealed candidate genes associated with essential traits in Garole sheep. We identified the *BBX* gene on OAR1, which is associated with supernumerary teats in sheep. Interestingly, a genome-wide association study of Wadi sheep in China identified candidate genes linked to the supernumerary nipple phenotype, including BBX [61]. *BBX* participates in cell proliferation and breast tumors, producing a high-mobility group domain (*HMG*) transcription factor observed in breast tumors [62]. DCMS analysis revealed the *CNTNAP5* gene on OAR 2, which regulates body growth, and it is also annotated in the Chanthangi [43]. The same genomic region was selected for in both breeds. We identified candidate genes on OAR 3 in GAR sheep, including *C2CD5, IQSEC3, KDM5A,* and *B4GALNT3*. Expression studies in sheep oviductal mRNAs and lncRNAs highlighted C*2CD5* [63], which is involved in calcium-dependent phospholipid binding and insulin receptor signalling, linked to prolificacy and litter size, aligning with the breed history. *KDM5A* plays a role in zygote genome activation in cloned goat embryos [64]. DCMS analysis also highlighted *KDM5A* in GAR sheep. *UNC5C,* another candidate gene under selection, was confirmed to influence litter size in Hu sheep [65]. *UNC5C* is a part of the *UNC-5* family of netrin receptors and is associated with conception rate in cattle [66]. Multiple genome-wide studies confirmed its role in sheep litter size [19,67,68].

The *ADCY2* gene, known for pleiotropic effects, produces *cAMP*, a signalling molecule in response to G protein signal transduction, leading to increased *IL-6* and influencing Brucellosis [69,70]. It also enhances estradiol expression, which is important for reproduction [71]. We identified *ADCY2* in our study.

#### 4.3.2. Quantitative trait loci (QTL) annotation and enrichment.

DCMS analysis identified QTL regions associated with teat number, body weight, body measurements, entropion, resistance to the Hemonchus infection, total lambs born, and total lambs alive.

We identified the peak QTL region at 201.6 cM; including markers rs430157497 and rs426598066. This region on OAR1, surrounding *BBX* and *CD47*, was associated with supernumerary teats in Wadi sheep in China [61]. A significant region on OAR6 was linked to bone area in Garole sheep; prior GWAS in Scottish Blackface lambs identified genes influencing bone density and area [72].

Garole sheep are known for their prolificacy and are valued for their litter size. Studies have confirmed genomic regions on OAR 6 and 22 that are potentially related to litter size in multiple breeds, including Wadi, Hu, Icelandic, Finnsheep, Romanov, and Lori-Bakhtiari [73,74]. Genes in these regions, including *BMPR1B*, *CTNNB1*, and *LHCGR*, significantly affect litter size. Remarkably, this QTL aligns with Garole’s breeding history.

A study on the Somatostatin Receptor Subtype 1 (SSTR1) gene polymorphism confirmed an association with growth traits in Hulun Buir Sheep. We observed QTL on OAR18 related to various body measurements such as body weight, length, circumference, rump width, body depth, chest width, and shin circumference [75].

### 4.4. Deccani sheep

#### 4.4.1. Gene annotation.

The Deccani breed is the only pure-black, coarse-wool breed indigenous to India**.** Our study identified significant genomic regions harboring candidate genes *MAEL, APP*, and *POU2F1*. *POU2F1* regulated melanin production by binding with *SLC7A11* and inhibiting its function [76]*.* It also affected other genes (*SLC7A11, MITF, SLC24A5, MC1R,* and *ASIP*), which impact melanin production and wool colour [77], supporting Deccani’s unique traits. Another region on OAR3 contained *MALL, NPHP1, BUB1, IQSEC3,* and *ACOXL. MALL* and *NPHP1* regulated cell division and cell-matrix adhesion signalling. The *BUB1* gene encodes a protein involved in the DNA damage response. *IQSEC3* controlled the small GTPase-mediated signal transduction, and *ACOXL* was associated with fatty acid beta-oxidation via acyl-CoA oxidase. Previous genomic studies also confirmed the role of this region in immune response [78]. Interestingly, we also identified this region.

We detected a region on OAR2 containing *HINT2, RGP1*, and *MSMP*, genes controlling growth and fat deposition. GWAS in Ethiopian sheep linked these genes to fat deposition and growth [79]. *HINT2* is involved in growth and fat deposition by facilitating the hydrolysis of various bonds, such as C-O, C-N, and C-C. *RGP1* gene promoted vesicle transport to the trans-Golgi network, and *MSMP* encoded a member of the beta-microseminoprotein family. Other studies have reported the associations with fat deposition and growth [80,81].

On OAR 14, *DOCK3* was a candidate gene associated with muscle development in cattle. Studies in Bashbay sheep highlighted the role of *DOCK3* in skeletal muscle development [82]. The *DOCK* gene family comprises guanine nucleotide exchange factors that play a crucial role in myoblast fusion and migration, expressed in skeletal muscle through *RAC1* and *WAVE* signalling pathways [83].

#### 4.4.2. Quantitative trait loci (QTL) annotation and enrichment.

The QTL analysis uncovered regions under selection controlling somatic cell count, entropion, footrot, pneumonia susceptibility, monocyte number, body weight, body length, water-holding capacity, and horn length. DCMS analysis identified a region associated with somatic cell count. A GWAS reported significant associations with milk production, composition, coagulation, and cheese traits, including somatic cell count, in Assaf and Churra dairy sheep [84].

Monocytes are a type of white blood cell vital to the immune response. Differentiated monocytes play a role in phagocytosis and antigen presentation, thereby supporting adaptive immunity. In sheep, monocytes and macrophages counteract the septicemic conditions caused by Coxiella burnetii and lentivirus [85]. GWAS in Rambouillet, Polypay and Columbia sheep identified loci associated with the monocyte count on multiple chromosomes, including OAR 1, 2, 3, 4, 9, 10, 15, and 16. We found the loci on OAR4 associated with the monocyte count in the Deccani sheep genome.

Two loci on chromosome 4 associated with foot rot and pneumonia susceptibility have been identified. A GWAS study in Katahdin, Blackbelly, and European-influenced crossbred sheep identified loci on chromosome 4 associated with foot rot susceptibility [86].

The genomic analyses revealed a significant association between body length and body weight on chromosomes 21 and 2. Notably, previous genome-wide association studies (GWAS) in Qira Black and German Merino sheep have also demonstrated associations between QTL regions and body measurements, including body weight traits [87].

The present study identified two robust genomic associations on chromosome 2 in Deccani sheep: one related to water-holding capacity (a meat quality trait) and the other to horn type. Deccani sheep are valued by consumers for their superior meat quality and distinctive physical characteristics, especially horn morphology, which is unique to the breed. Consistent with our findings, a GWAS on Colombian Creole hair sheep also reported strong associations between genomic regions on chromosome 2 and water-holding capacity in meat [88]. Furthermore, another genomic study elucidated the genomic basis of horn polymorphism in wild Soay sheep, revealing a strong association with regions on chromosome 2 [89].

The findings align with previous genomic research on Chanthangi sheep [18] reinforcing the identification of significant genomic regions and their associations. Similar studies [17,18,20], highlight candidate genes influencing growth, wool production, and disease resistance. These results validate existing genomic insights and contribute to understanding the genetic foundations of key traits. Consistency across studies emphasizes these region’s importance in sheep breeding programs.

This study uncovered breed- and trait-specific signature genomic regions and candidate genes in Indian sheep. Our DCMS analysis showed minimal overlap with regions reported by Muthuswamy et al. (2025). Overlapping regions included *STARD10, FCHSD2, P2RY2*, and *P2RY6* on *OAR* 15 in Changthangi, *UNC5C* on *OAR* 6 in Garole, and *APP* on *OAR* 1 in Deccani sheep. Concordance across both SNP-centric and window-based DCMS approaches underscores the robustness of the selection signals at these loci. However, the limited overlap also suggests that each analytical method captures unique aspects of the genomic landscape under selection: window-based methods may be more sensitive to broader regions with weaker, cumulative signals, and SNP-centric approaches offer greater precision, pinpointing individual loci with stronger effects.

The SNP-based de-correlated composite of multiple signals (DCMS) approach used in this study revealed high-resolution breed-specific selection signatures in Indian sheep. This method provided essential insights into the mechanisms underlying adaptation to challenging environments and the development of significant economic traits. The findings have important implications for the sustainable management and genetic improvement of indigenous livestock. The candidate genes and Quantitative Trait Loci (QTLs) identified in this study serve as key targets for marker-assisted selection to develop more productive and resilient animals. This study also emphasizes the importance of the native genetic resources, supporting conservation efforts to maintain their adaptive traits and diversity for future generations.

The results of this research align with prior investigations in identifying candidate genes and genomic regions linked to body weight, litter size, and disease resistance [17,18,20]. The PCA analysis in this study showed distinct clustering, contradicting the previous study on Indian sheep [18]. Furthermore, the minimal overlap between our identified selection signatures and those reported in earlier window-based DCMS analyses [20] for these breeds underscores a crucial distinction: our SNP-centric approach provides superior resolution, facilitating the identification of loci that may have been missed by broader genomic interval analyses, rather than indicating a disagreement in the underlying biological phenomena.

A key limitation of the present study is the absence of direct experimental validation for the identified candidate genes and quantitative trait loci. While our computational genomic approach provides strong evidence for selection signatures and putative functional associations, the precise biological mechanisms and phenotypic effects of these loci were not experimentally confirmed. Future research involving functional genomics experiments, such as gene editing or targeted expression studies, and detailed phenotypic validation on larger populations, would be crucial to conclusively establish the roles of these candidate regions in Indian sheep breeds.

## 5. Conclusions

We identified 51 putative QTL regions encompassing 45 protein-coding genes in Indian sheep breeds under selection and adapted to unique and distinct local climates. The putative QTLs and functional candidate genes revealed in the study reflect the historical adaptation and selection pressures experienced by these breeds**.** The identified signature genomic regions were associated with economic traits, including body growth, fecundity, heat stress response, domestication, environmental adaptation, hair follicle growth, horn type, and meat quality yield. Importantly, our analyses uncovered breed- and trait-specific genomic regions, such as those linked to hair follicle growth in Chanthangi, fecundity and lamb survivability in Garole, and meat quality traits in Deccani, which had not been previously reported in window-based studies. These results provide new insights into the molecular mechanisms underlying important traits and local adaptation in Indian sheep. Additionally, our comprehensive approach provides valuable genomic resources for future breeding programs to improve the genetics of indigenous Indian sheep breeds and conserve them. While these findings offer valuable insights and expand genomic resources, further experimental validation is warranted to confirm the functional roles of the identified candidate genes and QTLs. In conclusion, our SNP-based DCMS analysis revealed novel genes and QTLs associated with economically important traits in Indian sheep breeds, expanding the genomic resources available for their improvement and conservation. This work lays a foundation for precision breeding and sustainable management of indigenous livestock adapted to challenging environments.

## References

[1] Darwin C. 1859 The origin of species. London: Murray; 1962.

[2] Fay JC, Wu CI. Hitchhiking under positive Darwinian selection. Genetics. 2000;155(3):1405–13. doi: 10.1093/genetics/155.3.1405 10880498 PMC1461156

[3] Jensen JD, Foll M, Bernatchez L. The past, present and future of genomic scans for selection. Mol Ecol. 2016;25(1):1–4. doi: 10.1111/mec.13493 26745554

[4] Kim Y, Nielsen R. Linkage disequilibrium as a signature of selective sweeps. Genetics. 2004;1524:1513–24. doi: 10.1534/genetics.103.025387 PMC1470945 15280259

[5] Qanbari S, Simianer H. Mapping signatures of positive selection in the genome of livestock. Livest Sci. 2014;166:133–43. doi: 10.1016/j.livsci.2014.05.003

[6] Illa SK, Mukherjee S, Nath S, Mukherjee A. Genome-wide scanning for signatures of selection revealed the putative genomic regions and candidate genes controlling milk composition and coat color traits in sahiwal cattle. Front Genet. 2021;12:699422. doi: 10.3389/fgene.2021.699422 34306039 PMC8299338

[7] Karsli T. Genome-wide genetic characterization and selection signatures in Anatolian Merino sheep. Arch Anim Breed. 2025;68:161–9. doi: 10.5194/aab-68-161-2025 PMC13240681 42256732

[8] Karabaş M, Yılmaz O. Identification of selection signatures and genetic diversity in the sheep. Trop Anim Health Prod. 2025;57(2):68. doi: 10.1007/s11250-025-04307-9 39964635 PMC11836209

[9] Rodrigues JL, Braga LG, Watanabe RN, Schenkel FS, Berry DP, Buzanskas ME, et al. Genetic diversity and selection signatures in sheep breeds. J Appl Genet. 2025;66(3):675–87. doi: 10.1007/s13353-025-00941-z 39883377 PMC12367903

[10] Grossman SR, Shylakhter I, Karlsson EK, Byrne EH, Morales S, Frieden G, et al. A composite of multiple signals distinguishes causal variants in regions of positive selection. Science. 2010;327(5967):883–6. doi: 10.1126/science.1183863 20056855

[11] Staubach F, Lorenc A, Messer PW, Tang K, Petrov DA, Tautz D. Genome patterns of selection and introgression of haplotypes in natural populations of the house mouse (Mus musculus). PLoS Genet. 2012;8(8):e1002891. doi: 10.1371/journal.pgen.1002891 22956910 PMC3431316

[12] Lotterhos KE, Card DC, Schaal SM, Wang L, Collins C, Verity B. Composite measures of selection can improve the signal-to-noise ratio in genome scans. Methods Ecol Evol. 2017. doi: 10.1111/2041-210X.12774

[13] Ma Y, Ding X, Qanbari S, Weigend S, Zhang Q, Simianer H. Properties of different selection signature statistics and a new strategy for combining them. Heredity (Edinb). 2015;115(5):426–36. doi: 10.1038/hdy.2015.42 25990878 PMC4611237

[14] Kijas JW, Lenstra JA, Hayes B, Boitard S, Porto Neto LR, San Cristobal M, et al. Genome-wide analysis of the world’s sheep breeds reveals high levels of historic mixture and strong recent selection. PLoS Biol. 2012;10(2):e1001258. doi: 10.1371/journal.pbio.1001258 22346734 PMC3274507

[15] DADHF. Annual report. 2023.

[16] NBAGR. NBAGR Annual Report. 2022. pp. 1–23. Available from: https://nbagr.icar.gov.in/wp-content/uploads/2023/09/NBAGR_AR-2022.pdf

[17] Saravanan KA, Panigrahi M, Kumar H, Parida S, Bhushan B, Gaur GK, et al. Genomic scans for selection signatures revealed candidate genes for adaptation and production traits in a variety of cattle breeds. Genomics. 2021;113(3):955–63. doi: 10.1016/j.ygeno.2021.02.009 33610795

[18] Ahmad SF, Mehrotra A, Charles S, Ganai NA. Analysis of selection signatures reveals important insights into the adaptability of high-altitude Indian sheep breed Changthangi. Gene. 2021;799:145809. doi: 10.1016/j.gene.2021.145809 34224833

[19] Eydivandi S, Roudbar MA, Ardestani SS, Momen M, Sahana G. A selection signatures study among Middle Eastern and European sheep breeds. J Anim Breed Genet. 2021;138(5):574–88. doi: 10.1111/jbg.12536 33453096

[20] Muthusamy M, Akinsola OM, Pal P, Ramasamy C, Ramasamy S, Thiruvenkadan AK. Comparative genomic insights into adaptation, selection signatures, and population dynamics in indigenous Indian sheep and foreign breeds. 2025:1–16. doi: 10.3389/fgene.2025.1621960 PMC12408274 40919434

[21] Sempéré G, Moazami-Goudarzi K, Eggen A, Laloë D, Gautier M, Flori L. WIDDE: a Web-Interfaced next generation database for genetic diversity exploration, with a first application in cattle. BMC Genomics. 2015;16:940. doi: 10.1186/s12864-015-2181-1 26573482 PMC4647285

[22] Zheng X, Levine D, Shen J, Gogarten SM, Laurie C, Weir BS. A high-performance computing toolset for relatedness and principal component analysis of SNP data. Bioinformatics. 2012;28(24):3326–8. doi: 10.1093/bioinformatics/bts606 23060615 PMC3519454

[23] Nei M, Li WH. Mathematical model for studying genetic variation in terms of restriction endonucleases. Proc Natl Acad Sci U S A. 1979;76(10):5269–73. doi: 10.1073/pnas.76.10.5269 291943 PMC413122

[24] Weir BS, Cockerham CC. Estimating F-statistics for the analysis of population structure. Evolution. 1984;38(6):1358–70. doi: 10.1111/j.1558-5646.1984.tb05657.x 28563791

[25] Tajima F. Statistical method for testing the neutral mutation hypothesis by DNA polymorphism. Genetics. 1989;123(3):585–95. doi: 10.1093/genetics/123.3.585 2513255 PMC1203831

[26] Garud NR, Messer PW, Buzbas EO, Petrov DA. Recent selective sweeps in North American Drosophila melanogaster show signatures of soft sweeps. PLoS Genet. 2015;11(2):e1005004. doi: 10.1371/journal.pgen.1005004 25706129 PMC4338236

[27] Yurchenko AA, Daetwyler HD, Yudin N, Schnabel RD, Vander Jagt CJ, Soloshenko V, et al. Scans for signatures of selection in Russian cattle breed genomes reveal new candidate genes for environmental adaptation and acclimation. Sci Rep. 2018;8(1):12984. doi: 10.1038/s41598-018-31304-w 30154520 PMC6113280

[28] Delaneau O, Marchini J, Zagury J-F. A linear complexity phasing method for thousands of genomes. Nat Methods. 2011;9(2):179–81. doi: 10.1038/nmeth.1785 22138821

[29] Barbato M, Orozco-terWengel P, Tapio M, Bruford MW. SNeP: a tool to estimate trends in recent effective population size trajectories using genome-wide SNP data. Front Genet. 2015;6:109. doi: 10.3389/fgene.2015.00109 25852748 PMC4367434

[30] Petit M, Astruc J-M, Sarry J, Drouilhet L, Fabre S, Moreno CR, et al. Variation in recombination rate and its genetic determinism in sheep populations. Genetics. 2017;207(2):767–84. doi: 10.1534/genetics.117.300123 28978774 PMC5629338

[31] Danecek P, Auton A, Abecasis G, Albers CA, Banks E, DePristo MA, et al. The variant call format and VCFtools. Bioinformatics. 2011. doi: 10.1093/bioinformatics/btr330 PMC3137218 21653522

[32] Verity R, Collins C, Card D, Schaal S, Wang L, Lotterhos K. MINOTAUR: A platform for the analysis and visualization of multivariate results from genome scans with R Shiny. 2016. doi: 10.1101/062158 27473028

[33] Todorov V, Templ M, Filzmoser P. Detection of multivariate outliers in business survey data with incomplete information. Adv Data Anal Classif. 2010;5(1):37–56. doi: 10.1007/s11634-010-0075-2

[34] Venables WN, Ripley BD. Modern Applied Statistics with S. 4 ed. World. 2002.

[35] Benjamini Y, Hochberg Y. Controlling the false discovery rate: a practical and powerful approach to multiple testing. J R Stat Soc Series B: Stat Methodol. 1995;57(1):289–300. doi: 10.1111/j.2517-6161.1995.tb02031.x

[36] Storey JD, Tibshirani R. Statistical significance for genomewide studies. Proc Natl Acad Sci U S A. 2003;100(16):9440–5. doi: 10.1073/pnas.1530509100 12883005 PMC170937

[37] Benjamini Y, Hochberg Y. Controlling the false discovery rate: a practical and powerful approach to multiple testing. J R Stat Soc Series B: Stat Methodol. 1995;57(1):289–300. doi: 10.1111/j.2517-6161.1995.tb02031.x

[38] Fonseca PAS, Suárez-Vega A, Marras G, Cánovas Á. GALLO: An R package for genomic annotation and integration of multiple data sources in livestock for positional candidate loci. Gigascience. 2020;9(12):giaa149. doi: 10.1093/gigascience/giaa149 33377911 PMC7772745

[39] Hu Z-L, Park CA, Reecy JM. Building a livestock genetic and genomic information knowledgebase through integrative developments of Animal QTLdb and CorrDB. Nucleic Acids Res. 2019;47(D1):D701–10. doi: 10.1093/nar/gky1084 30407520 PMC6323967

[40] Ahmad SF, Mehrotra A, Charles S, Ganai NA. Analysis of selection signatures reveals important insights into the adaptability of high-altitude Indian sheep breed Changthangi. Gene. 2021;799:145809. doi: 10.1016/j.gene.2021.145809 34224833

[41] Hirschhorn J, Ingelsson E, Genet N. Association analyses of 249,796 individuals reveal eighteen new loci associated with body mass index. Nat Genet. 2011;42:937–48. doi: 10.1038/ng.686 PMC3014648 20935630

[42] Zielke LG, Bortfeldt RH, Tetens J, Brockmann GA. BDNF contributes to the genetic variance of milk fat yield in german holstein cattle. Front Genet. 2011;2:16. doi: 10.3389/fgene.2011.00016 22303313 PMC3268571

[43] Rahmatalla SA, Arends D, Reissmann M, Wimmers K, Reyer H, Brockmann GA. Genome-wide association study of body morphological traits in Sudanese goats. Anim Genet. 2018;49(5):478–82. doi: 10.1111/age.12686 30062755

[44] Duan Y, Su P, Gu Y, Lv X, Cao X, Wang S, et al. A study of the resistance of Hu sheep lambs to Escherichia coli F17 based on whole genome sequencing. Animals (Basel). 2024;14(1):161. doi: 10.3390/ani14010161 38200892 PMC10778179

[45] Purdie AC, Plain KM, Begg DJ, de Silva K, Whittington RJ. Gene expression profiles during subclinical Mycobacterium avium subspecies paratuberculosis infection in sheep can predict disease outcome. Sci Rep. 2019;9(1):8245. doi: 10.1038/s41598-019-44670-w 31160677 PMC6547741

[46] Ito M, Yamanashi Y, Toyoda Y, Izumi-Nakaseko H, Oda S, Sugiyama A, et al. Disruption of Stard10 gene alters the PPARα-mediated bile acid homeostasis. Biochim Biophys Acta. 2013;1831(2):459–68. doi: 10.1016/j.bbalip.2012.11.008 23200860

[47] Yang J, Wang D-F, Huang J-H, Zhu Q-H, Luo L-Y, Lu R, et al. Structural variant landscapes reveal convergent signatures of evolution in sheep and goats. Genome Biol. 2024;25(1):148. doi: 10.1186/s13059-024-03288-6 38845023 PMC11155191

[48] Wang K, Zheng M, Ren Y. Overexpression of TRMT12 may independently predict poor overall survival in patients with head and neck squamous cell carcinoma. Onco Targets Ther. 2019;12:7269–79. doi: 10.2147/OTT.S212200 31564910 PMC6733347

[49] Rodriguez V, Chen Y, Elkahloun A, Dutra A, Pak E, Chandrasekharappa S. Chromosome 8 BAC array comparative genomic hybridization and expression analysis identify amplification and overexpression of TRMT12 in breast cancer. Genes Chromosomes Cancer. 2007;46(7):694–707. doi: 10.1002/gcc.20454 17440925

[50] Xu P, Wang C, Xiang W, Liang Y, Li Y, Zhang X, et al. P2RY6 Has a Critical Role in Mouse Skin Carcinogenesis by Regulating the YAP and β-Catenin Signaling Pathways. J Invest Dermatol. 2022;142(9):2334–2342.e8. doi: 10.1016/j.jid.2022.02.017 35304248

[51] Tian D, Pei Q, Jiang H, Guo J, Ma X, Han B, et al. Comprehensive analysis of the expression profiles of mRNA, lncRNA, circRNA, and miRNA in primary hair follicles of coarse sheep fetal skin. BMC Genomics. 2024;25:1–15. doi: 10.1186/s12864-024-10427-7 38849762 PMC11161951

[52] Pan R, Qi L, Xu Z, Zhang D, Nie Q, Zhang X, et al. Weighted single-step GWAS identified candidate genes associated with carcass traits in a Chinese yellow-feathered chicken population. Poult Sci. 2024;103(2):103341. doi: 10.1016/j.psj.2023.103341 38134459 PMC10776626

[53] Gu S, Huang Q, Sun C, Wen C, Yang N. Transcriptomic and epigenomic insights into pectoral muscle fiber formation at the late embryonic development in pure chicken lines. Poult Sci. 2024;103(8):103882. doi: 10.1016/j.psj.2024.103882 38833745 PMC11190745

[54] Al-Mamun HA, Kwan P, Clark SA, Ferdosi MH, Tellam R, Gondro C. Genome-wide association study of body weight in Australian Merino sheep reveals an orthologous region on OAR6 to human and bovine genomic regions affecting height and weight. Genet Sel Evol. 2015;47(1):66. doi: 10.1186/s12711-015-0142-4 26272623 PMC4536601

[55] Zhang L, Ma X, Xuan J, Wang H, Yuan Z, Wu M, et al. Identification of MEF2B and TRHDE gene polymorphisms related to growth traits in a new ujumqin sheep population. PLoS One. 2016;11(7):e0159504. doi: 10.1371/journal.pone.0159504 27472808 PMC4966928

[56] Pasandideh M, Gholizadeh M, Rahimi-Mianji G. A genome-wide association study revealed five SNPs affecting 8-month weight in sheep. Anim Genet. 2020;51(6):973–6. doi: 10.1111/age.12996 32910467

[57] Armstrong E, Ciappesoni G, Iriarte W, Da Silva C, Macedo F, Navajas EA, et al. Novel genetic polymorphisms associated with carcass traits in grazing Texel sheep. Meat Sci. 2018;145:202–8. doi: 10.1016/j.meatsci.2018.06.014 29982074

[58] Gonzalez MV, Mousel MR, Herndon DR, Jiang Y, Dalrymple BP, Reynolds JO, et al. A divergent Artiodactyl MYADM-like repeat is associated with erythrocyte traits and weight of lamb weaned in domestic sheep. PLoS One. 2013;8(8):e74700. doi: 10.1371/journal.pone.0074700 24023702 PMC3758307

[59] Li H, Wu X-L, Tait RG Jr, Bauck S, Thomas DL, Murphy TW, et al. Genome-wide association study of milk production traits in a crossbred dairy sheep population using three statistical models. Anim Genet. 2020;51(4):624–8. doi: 10.1111/age.12956 32510640

[60] Pan S, Sahoo AK. The Garole sheep–history, management, production and current status. In: Walkden-Brown SW, van der Werf JHJ, Gupta VS, editors. Proceedings of ‘Use of the FecB (Booroola) gene in sheep-breeding programs’. Canberra: Australian Centre for International Agricultural Research; 2008. pp. 133: 32–43.

[61] Peng W-F, Xu S-S, Ren X, Lv F-H, Xie X-L, Zhao Y-X, et al. A genome-wide association study reveals candidate genes for the supernumerary nipple phenotype in sheep (Ovis aries). Anim Genet. 2017;48(5):570–9. doi: 10.1111/age.12575 28703336

[62] Wen J, Toomer KH, Chen Z, Cai X. Genome-wide analysis of alternative transcripts in human breast cancer. Breast Cancer Res Treat. 2015;151(2):295–307. doi: 10.1007/s10549-015-3395-2 25913416 PMC5070939

[63] Chen W, Li Z, Zhong R, Sun W, Chu M. Expression profiles of oviductal mRNAs and lncRNAs in the follicular phase and luteal phase of sheep (Ovis aries) with 2 fecundity gene (FecB) genotypes. G3: Genes Genomes Genetics. 2024;14:1–16. doi: 10.1093/g3journal/jkad270 PMC10755197 38051961

[64] Deng M, Zhang G, Cai Y, Liu Z, Zhang Y, Meng F, et al. DNA methylation dynamics during zygotic genome activation in goat. Theriogenology. 2020;156:144–54. doi: 10.1016/j.theriogenology.2020.07.008 32731098

[65] Bao J, Xiong J, Huang J, Yang P, Shang M, Zhang L. Genetic diversity, selection signatures, and genome-wide association study identify candidate genes related to litter size in Hu sheep. Int J Mol Sci. 2024;25(17):9397. doi: 10.3390/ijms25179397 39273345 PMC11395453

[66] Sugimoto M, Gotoh Y, Kawahara T, Sugimoto Y. Molecular effects of polymorphism in the 3’UTR of Unc-5 homolog C associated with conception rate in Holsteins. PLoS One. 2015;10(7):e0131283. doi: 10.1371/journal.pone.0131283 26147436 PMC4493121

[67] Zhao F, Xie R, Fang L, Xiang R, Yuan Z, Liu Y, et al. Analysis of 206 whole-genome resequencing reveals selection signatures associated with breed-specific traits in Hu sheep. Evol Appl. 2024;17(6):e13697. doi: 10.1111/eva.13697 38911262 PMC11192971

[68] Zhong T, Hou D, Zhao Q, Zhan S, Wang L, Li L, et al. Comparative whole-genome resequencing to uncover selection signatures linked to litter size in Hu Sheep and five other breeds. BMC Genomics. 2024;25(1):480. doi: 10.1186/s12864-024-10396-x 38750582 PMC11094944

[69] Karaoglan I, Pehlivan S, Namiduru M, Pehlivan M, Kilinçarslan C, Balkan Y, et al. TNF-alpha, TGF-beta, IL-10, IL-6 and IFN-gamma gene polymorphisms as risk factors for brucellosis. New Microbiol. 2009;32(2):173–8. 19579695

[70] Li X, Wu Q, Zhang X, Li C, Zhang D, Li G, et al. Whole-genome resequencing to study Brucellosis susceptibility in sheep. Front Genet. 2021;12:653927. doi: 10.3389/fgene.2021.653927 34306007 PMC8297390

[71] Zhao G, Li X, Miao H, Chen S, Hou Y. Estrogen promotes cAMP production in mesenchymal stem cells by regulating ADCY2. Int J Stem Cells. 2020;13(1):55–64. doi: 10.15283/ijsc19139 32114743 PMC7119214

[72] Matika O, Riggio V, Anselme-Moizan M, Law AS, Pong-Wong R, Archibald AL, et al. Genome-wide association reveals QTL for growth, bone and in vivo carcass traits as assessed by computed tomography in Scottish Blackface lambs. Genet Sel Evol. 2016;48:11. doi: 10.1186/s12711-016-0191-3 26856324 PMC4745175

[73] Xu S-S, Gao L, Xie X-L, Ren Y-L, Shen Z-Q, Wang F, et al. Genome-wide association analyses highlight the potential for different genetic mechanisms for litter size among sheep breeds. Front Genet. 2018;9:118. doi: 10.3389/fgene.2018.00118 29692799 PMC5902979

[74] Abdoli R, Mirhoseini SZ, Ghavi Hossein-Zadeh N, Zamani P, Moradi MH, Ferdosi MH, et al. Runs of homozygosity and cross-generational inbreeding of Iranian fat-tailed sheep. Heredity (Edinb). 2023;130(6):358–67. doi: 10.1038/s41437-023-00611-y 37016136 PMC10238534

[75] Li X, Ding N, Zhang Z, Tian D, Han B, Liu S. Identification of Somatostatin Receptor Subtype 1 (SSTR1) gene polymorphism and their association with growth traits in Hulun Buir sheep. Genes. 2022;13. doi: 10.3390/genes13010077 PMC8775034 35052417

[76] Chen Y, Hu S, Mu L, Zhao B, Wang M, Yang N, et al. Slc7a11 Modulated by POU2F1 is Involved in Pigmentation in Rabbit. Int J Mol Sci. 2019;20(10):2493. doi: 10.3390/ijms20102493 31137576 PMC6566412

[77] Yang N, Zhao B, Hu S, Bao Z, Liu M, Chen Y, et al. Characterization of POU2F1 gene and its potential impact on the expression of genes involved in fur color formation in rex rabbit. Genes (Basel). 2020;11(5):575. doi: 10.3390/genes11050575 32443864 PMC7288328

[78] Saravanan KA, Panigrahi M, Kumar H, Bhushan B, Dutt T, Mishra BP. Genome-wide analysis of genetic diversity and selection signatures in three Indian sheep breeds. Livest Sci. 2021;243:104367. doi: 10.1016/j.livsci.2020.104367

[79] Ahbara A, Bahbahani H, Almathen F, Al Abri M, Agoub MO, Abeba A, et al. Genome-wide variation, candidate regions and genes associated with fat deposition and tail morphology in Ethiopian indigenous sheep. Front Genet. 2019;9:699. doi: 10.3389/fgene.2018.00699 30687385 PMC6334744

[80] Casas E, Shackelford SD, Keele JW, Stone RT, Kappes SM, Koohmaraie M. Quantitative trait loci affecting growth and carcass composition of cattle segregating alternate forms of myostatin. J Anim Sci. 2000;78(3):560–9. doi: 10.2527/2000.783560x 10764062

[81] McClure MC, Morsci NS, Schnabel RD, Kim JW, Yao P, Rolf MM, et al. A genome scan for quantitative trait loci influencing carcass, post-natal growth and reproductive traits in commercial Angus cattle. Anim Genet. 2010;41(6):597–607. doi: 10.1111/j.1365-2052.2010.02063.x 20477797

[82] Wu X, Fang M, Liu L, Wang S, Liu J, Ding X, et al. Genome wide association studies for body conformation traits in the Chinese Holstein cattle population. 2013. 10.1186/1471-2164-14-897 PMC3879203 24341352

[83] Samani A, Karuppasamy M, English KG, Siler CA, Wang Y, Widrick JJ. Glucose metabolism. 2024;37:1–27. doi: 10.1096/fj.202300386RR.DOCK3 PMC10539028 37742307

[84] Marina H, Pelayo R, Suárez-Vega A, Gutiérrez-Gil B, Esteban-Blanco C, Arranz JJ. Genome-wide association studies (GWAS) and post-GWAS analyses for technological traits in Assaf and Churra dairy breeds. J Dairy Sci. 2021;104(11):11850–66. doi: 10.3168/jds.2021-20510 34454756

[85] Oliveira RD, Mousel MR, Gonzalez MV, Durfee CJ, Davenport KM, Murdoch BM, et al. A high-density genome-wide association with absolute blood monocyte count in domestic sheep identifies novel loci. PLoS One. 2022;17(5):e0266748. doi: 10.1371/journal.pone.0266748 35522671 PMC9075649

[86] Cinar MU, Oliveira RD, Hadfield TS, Lichtenwalner A, Brzozowski RJ, Settlemire CT, et al. Genome-wide association with footrot in hair and wool sheep. Front Genet. 2024;14:1297444. doi: 10.3389/fgene.2023.1297444 38288162 PMC10822918

[87] Tuersuntuoheti M, Zhang J, Zhou W, Zhang C-L, Liu C, Chang Q, et al. Exploring the growth trait molecular markers in two sheep breeds based on Genome-wide association analysis. PLoS One. 2023;18(3):e0283383. doi: 10.1371/journal.pone.0283383 36952432 PMC10035858

[88] Revelo HA, López-Alvarez D, Palacios YA, Vergara OD, Yánez MB, Ariza MF, et al. Genome-wide association study reveals candidate genes for traits related to meat quality in Colombian Creole hair sheep. Trop Anim Health Prod. 2023;55(6):357. doi: 10.1007/s11250-023-03688-z 37823994 PMC10570192

[89] Johnston SE, McEwan JC, Pickering NK, Kijas JW, Beraldi D, Pilkington JG, et al. Genome-wide association mapping identifies the genetic basis of discrete and quantitative variation in sexual weaponry in a wild sheep population. Mol Ecol. 2011;20(12):2555–66. doi: 10.1111/j.1365-294X.2011.05076.x 21651634

