# Supplementary material for: Composite selection signal analysis: Uncovering candidate genes and quantitative trait loci in Indian sheep breeds
Source: PLoS One. 2026 Mar 25;21(3):e0344299. doi: 10.1371/journal.pone.0344299 (PMC13016353; doi:10.1371/journal.pone.0344299)

PLOS ONE

Composite Selection Signal Analysis: Uncovering Candidate Genes and Quantitative Trait Loci in Indian Sheep Breeds

--Manuscript Draft--

**Manuscript Number:**	PONE-D-24-51732
**Article Type:**	Research Article
**Full Title:**	Composite Selection Signal Analysis: Uncovering Candidate Genes and Quantitative Trait Loci in Indian Sheep Breeds
**Short Title:**	Composite Selection Signal Analysis
**Corresponding Author:**	Destaw Worku Worku<br>Injibara University<br>Injibara, Amhara ETHIOPIA
**Keywords:**	DCMS; Indian Sheep; SNP; QTL; Selection Signature
**Abstract:**	Natural and artificial selection are fundamental demographic forces that contribute significantly to the shaping of genomes. Exploring the molecular mechanisms implicated in the adaptation processes can assist in creating efficient breeding programs designed to preserve and improve the genetic characteristics of native sheep. The study used a novel method that combines the p-values into a single measure to investigate the genomic regions under selection. This method improves the resolution power and prioritises the candidate genes more efficiently than other single statistics. The 50K SNP data on three Indigenous sheep breeds (n=79) were Chantangi (CHA, n= 29), Garole (GAR, n=24), and Deccani (IDC, n=26) native to diverse regions of India were used in this study. The study combined five statistics into a composite selection signal called a De-Correlated composite of multiple signals (DCMS). The p-values of all the single statistics were corrected for multiple testing at the False Discovery Rate of < 5%, and the putative genomic regions were detected. Gene and quantitative trait loci (QTL) annotation of the significant regions was performed. The findings included Novel putative genes related to a wide variety of characteristics, including MTSS1 (host resistance to bacterial infections), TRMT12 (environment adaptation), TPPP3, P2RY6, FGF10 and FAM168A (hair follicle growth), LRRC36 (domestication), BBX (teat number), CNTNAP5 (body growth), C2CD5 and UNC5C (fecundity), B4GALNT3 (paratuberculosis resistance), TCERG1L (reproduction), BUB1 (oocyte and follicle development), POU2F1 (wool colour), DOCK3 (muscle development), MAPKAPK3 (heat stress response). The QTLs identified are associated with milk fat yield, platelet count, entropion, bone area, total lambs born, body measurements, hemonchus resistance, foot rot and pneumonia susceptibility and entropion. The DCMS method pinpointed novel genomic regions and putative genes more efficiently than other single statistics and could provide reliable comprehension of the molecular mechanisms involved in all the essential traits in the Indian sheep.
**Order of Authors:**	Destaw Worku
	Sapna Nath
	Satish Kumar Illa
	Sabyasachi Mukherjee
	Anupama Mukherjee
	Vinod Kumar Yata
**Additional Information:**	
**Question**	**Response**
**Financial Disclosure**<br><br>Enter a financial disclosure statement that	The author(s) received no specific funding for this work.

Powered by Editorial Manager® and ProduXion Manager® from Aries Systems Corporation

describes the sources of funding for the work included in this submission. Review the submission guidelines for detailed requirements. View published research articles from *PLOS ONE* for specific examples.<br><br>This statement is required for submission and **will appear in the published article** if the submission is accepted. Please make sure it is accurate.<br><br>**Funded studies**<br>Enter a statement with the following details:<br>• Initials of the authors who received each award<br>• Grant numbers awarded to each author<br>• The full name of each funder<br>• URL of each funder website<br>• Did the sponsors or funders play any role in the study design, data collection and analysis, decision to publish, or preparation of the manuscript?<br><br>Did you receive funding for this work?	
**Competing Interests**<br><br>Use the instructions below to enter a competing interest statement for this submission. On behalf of all authors, disclose any competing interests that could be perceived to bias this work—acknowledging all financial support and any other relevant financial or non-financial competing interests.<br><br>This statement is required for submission and **will appear in the published article** if the submission is accepted. Please make sure it is accurate and that any funding sources listed in your Funding Information later in the submission form are also declared in your Financial Disclosure statement.<br><br>View published research articles from *PLOS ONE* for specific examples.	The authors have declared that no competing interests exist.

**NO authors have competing interests**<br><br>Enter: *The authors have declared that no competing interests exist.*<br><br>**Authors with competing interests**<br><br>Enter competing interest details beginning with this statement:<br><br>*I have read the journal's policy and the authors of this manuscript have the following competing interests: [insert competing interests here]*<br><br>* typeset	
**Ethics Statement**<br><br>Enter an ethics statement for this submission. This statement is required if the study involved:<br><br>• Human participants<br>• Human specimens or tissue<br>• Vertebrate animals or cephalopods<br>• Vertebrate embryos or tissues<br>• Field research<br><br>Write "N/A" if the submission does not require an ethics statement.<br><br>General guidance is provided below. Consult the submission guidelines for detailed instructions. **Make sure that all information entered here is included in the Methods section of the manuscript.**	Sri Venkateswara Veterinary University, Tirupati, Andhra Pradesh

**Format for specific study types**<br><br>**Human Subject Research (involving human participants and/or tissue)**<br>• Give the name of the institutional review board or ethics committee that approved the study<br>• Include the approval number and/or a statement indicating approval of this research<br>• Indicate the form of consent obtained (written/oral) or the reason that consent was not obtained (e.g. the data were analyzed anonymously)<br><br>**Animal Research (involving vertebrate animals, embryos or tissues)**<br>• Provide the name of the Institutional Animal Care and Use Committee (IACUC) or other relevant ethics board that reviewed the study protocol, and indicate whether they approved this research or granted a formal waiver of ethical approval<br>• Include an approval number if one was obtained<br>• If the study involved *non-human primates*, add *additional details* about animal welfare and steps taken to ameliorate suffering<br>• If anesthesia, euthanasia, or any kind of animal sacrifice is part of the study, include briefly which substances and/or methods were applied<br><br>**Field Research**<br><br>Include the following details if this study involves the collection of plant, animal, or other materials from a natural setting:<br>• Field permit number<br>• Name of the institution or relevant body that granted permission	
**Data Availability**<br><br>Authors are required to make all data underlying the findings described fully available, without restriction, and from the time of publication. PLOS allows rare exceptions to address legal and ethical concerns. See the PLOS Data Policy and FAQ for detailed information.	No - some restrictions will apply

Powered by Editorial Manager® and ProduXion Manager® from Aries Systems Corporation

A Data Availability Statement describing where the data can be found is required at submission. Your answers to this question constitute the Data Availability Statement and **will be published in the article**, if accepted.<br><br>**Important:** Stating 'data available on request from the author' is not sufficient. If your data are only available upon request, select 'No' for the first question and explain your exceptional situation in the text box.<br><br>Do the authors confirm that all data underlying the findings described in their manuscript are fully available without restriction?	
**Describe where the data may be found in full sentences. If you are copying our sample text, replace any instances of XXX with the appropriate details.**<br><br>• If the data are **held or will be held in a public repository**, include URLs, accession numbers or DOIs. If this information will only be available after acceptance, indicate this by ticking the box below. For example: *All XXX files are available from the XXX database (accession number(s) XXX, XXX.).*<br>• If the data are all contained **within the manuscript and/or Supporting Information files**, enter the following: *All relevant data are within the manuscript and its Supporting Information files.*<br>• If neither of these applies but you are able to provide **details of access elsewhere**, with or without limitations, please do so. For example:<br><br>*Data cannot be shared publicly because of [XXX]. Data are available from the XXX Institutional Data Access / Ethics Committee (contact via XXX) for researchers who meet the criteria for access to confidential data.*<br><br>*The data underlying the results presented in the study are available from (include the name of the third party*	The genotypic data and other material related to this study are available with the corresponding author

and contact information or URL).<br>• This text is appropriate if the data are owned by a third party and authors do not have permission to share the data.<br><br>* typeset	
Additional data availability information:	

Click here to access/download;Manuscript;Manuscript_Destaw.doc

Composite Selection Signal Analysis: Uncovering Candidate Genes and Quantitative Trait Loci in Indian Sheep Breeds

Sapna Nath[a¶], Satish Kumar Illa[b¶], Destaw Worku[c*], Sabyasachi Mukherjee[d], Anupama Mukherjee[d], Vinod Kumar Yata[e]

[a] College of Veterinary Science, Garividi, Sri Venkateswara Veterinary University, Andhra Pradesh, India.

[b] Livestock Research Station, Garividi, Sri Venkateswara Veterinary University, Andhra Pradesh, India.

[c] Department of Animal Science, College of Agriculture, Food and Climate Science, Injibara University, Ethiopia

[d] Division of Animal Genetics and Breeding, Indian Council of Agricultural Research-National Dairy Research Institute, Karnal, Haryana, India.

[e] Department of Molecular Biology, Central University of Andhra Pradesh, Ananthapuramu, Andhra Pradesh, India

¶Authors contributed equally to the work

*Corresponding author: destawworku@gmail.com

Abstract:

Natural and artificial selection are fundamental demographic forces that contribute significantly to the shaping of genomes. Exploring the molecular mechanisms implicated in the adaptation processes can assist in creating efficient breeding programs designed to preserve and improve the genetic characteristics of native sheep. The study used a novel method that combines the *p*-values into a single measure to investigate the genomic regions under selection. This method improves the resolution power and prioritises the candidate genes more efficiently than other single statistics. The 50K SNP data on three Indigenous sheep breeds (n=79) were Chantangi (CHA, n= 29), Garole (GAR, n=24), and Deccani (IDC, n=26) native to diverse regions of India were used in this study. The study combined five statistics into a composite selection signal called a De-Correlated composite of multiple signals (DCMS). The *p*-values of all the single statistics were corrected for multiple testing at the False Discovery Rate of < 5%, and the putative genomic regions were detected. Gene and quantitative trait loci (QTL) annotation of the significant regions was performed. The findings included Novel putative genes related to a wide variety of characteristics, including *MTSS1* (host resistance to bacterial infections), *TRMT12* (environment adaptation), *TPPP3, P2RY6, FGF10* and *FAM168A* (hair follicle growth), *LRRC36* (domestication), *BBX* (teat number), *CNTNAP5* (body growth), *C2CD5* and *UNC5C* (fecundity), *B4GALNT3* (paratuberculosis resistance), *TCERG1L* (reproduction), *BUB1* (oocyte and follicle development), *POU2F1* (wool colour), *DOCK3* (muscle development), *MAPKAPK3* (heat stress response). The QTLs identified are associated with milk fat yield, platelet count, entropion, bone area, total lambs born, body measurements, hemonchus resistance, foot rot and pneumonia susceptibility and entropion. The DCMS method pinpointed novel genomic regions and putative genes more efficiently than other single statistics and could provide reliable comprehension of the molecular mechanisms involved in all the essential traits in the Indian sheep.

Keywords: DCMS, Indian Sheep, SNP, QTL, Selection Signature

Introduction:

Natural selection enhances the fitness value of individuals and allows them to reproduce. Hence, the frequency of beneficial mutations increases in the populations. [1]. Thus, selection pressure on the genome leaves a distinct pattern, and those unique footprints are referred to as selection signatures [2,3]. However, complicated evolutionary processes, including population admixture, inbreeding, population bottlenecks, genetic drift, and mutations, obscure the patterns of selection signatures.

SNP genotyping arrays have revolutionized the study of genomic variations in populations, and nowadays, they are commonly used in genomic studies. These arrays consist of genetically stable single nucleotide polymorphisms distributed across the genome. Several methods are described in the literature to identify the signatures of selection based on allele frequency spectrum, linkage disequilibrium, and population differentiation [4,5]. However, Grossman et al., 2010 proposed a method that combines different signals into a composite of signals (CMS) to enhance the details of the genomic region. This method is believed to be more sensitive and robust than the single selection signal method. The integral haplotype score method gives better signals if the genome experiences incomplete or ongoing selection. Meanwhile, the XPEHH method efficiently pinpoints the signals that are almost fixed. Hence, most of the studies on farm animals used complementary methods to improve the resolution of signals [4,7].

However, Lotterhos et al., 2017; Ma et al., 2015, proposed an efficient method called a de-correlated composite of multiple signals (DCMS) that combines the results obtained from univariate analysis. The covariance structure among the output signals of single methods is accounted for to obtain more robust and sensitive signals. DCMS provides accurate signals owing to its better resolution power. Hence, this method is preferred in selection signature analysis. The OvineSNP50 Bead Chip (Illumina Inc., San Diego, CA) is the most commonly used SNP array to study the variations of the sheep genome. This SNP array was developed based on the single nucleotide polymorphisms

present in seventy-five (75) sheep breeds, which are of paramount economic importance [10].

The sheep production systems in India are diverse and include cold arid (Western Himalayas), hot arid (Rajasthan and surrounding states), semi-arid (Deccan plateau and adjacent areas), and sub-humid regions (West Bengal and Orrissa). According to the 19th Census in India, the sheep population is 74.44 million heads, including 44 registered breeds [11]. The Indian sheep genetic resources comprise diverse morphological, physiological, and behavioral attributes developed over centuries of adaptation to various ecological niches and production systems.

Despite the rich phenotypic diversity observed in Indian sheep, comprehensive genomic studies that aim to uncover these traits' molecular basis have been relatively limited. However, few studies on selection signatures in Indian sheep using single statistic or a combination of univariate statistics were reported [12,13]. Eydivandi et al., 2021 studied the selection signature analysis in Indian sheep using a combined statistic accounting for the correlations. However, the study did not use the composite signal method to detect the signals within the breed. Our study used the composite signals method (DCMS), which is more robust, sensitive and advantageous than other univariate statistics methods to detect the selection signals within the genome of three Indian sheep breeds (Chantangi, Deccani and Garole) that are adapted to diverse ecological niche and production systems of India. Further, the candidate genes and putative quantitative trait loci (QTL) were annotated, and qtl enrichment was studied.

Materials & Methods

Animals and Genotyping

In the present study, the high dense genotypes of three Indian sheep breeds i. e., Changthangi (CHA, n = 29), Deccani (IDC, n = 24), and Garole (GAR, n = 26) genotyped with Illumina Ovine SNP50 BeadChip were obtained from WIDDE [14]. The Illumina Ovine SNP50 BeadChip consist of 54,241 SNPs located with an average density of one SNP per 51 kb. The Plink v1.9 program [15] was used to filter the poor-quality SNPs for

further analysis. The quality control (QC) of genotypes included the minor allele frequency (maf <0.05), Hardy Weinberg Equilibrium (hwe 0.0001), Individuals with missing genotypes (--mind< 0.1) and missing genotypes (geno<0.1). A total of 41,997 SNPs were retained for further analysis. The SNPs with unknown coordinates and SNPs on X-chromosomes were excluded. The SNPs with precise coordinates based on OAR Ramboulliet reference genome assembly were included for analysis.

Principal Component Analysis:

The principal component analysis (PCA) was performed on the SNP genotypes of the studied sheep breeds to investigate the structure and clustering of the individuals in the dataset. The PCA analysis was implemented in the R environment using the *snprelate* package [16].

De-Correlated Composite of Multiple Selection Signals

As detailed in Yurchenko et al., 2018, the present study utilised the Composite of multiple signals method (DCMS) by combining five different univariate statistics such as FST, Haplotype Homozygosity (H1), Modified Haplotype Homozygosity (H12), Tajima's D index, and Nucleotide diversity (pi) [18–21].

The computation of the DCMS statistic for a loci l is given below

$$DCMS_l = \sum_{t=1}^{n} \frac{\log\left[\frac{1-P_{lt}}{P_{lt}}\right]}{\sum_{i=1}^{n}|r_{it}|}$$

The DCMS statistic is composed of two components. The numerator component includes P_{lt}, which indicates the p-value at a given loci 'l' of each univariate statistic 't'. The denominator contains the correlation parameter 'r_{it}', which accounts for the covariance structure among all the included univariate statistics. It is also described as weighing factors among the statistics that describe the correlations between the statistics [8].

Univariate statistics in DCMS

Fixation Index (F_{ST}):

The F_{ST} index denotes the degree of differentiation between the populations. The functions in *PLINKV1.9*, such as --fst and --within, were used to determine the FST values for each SNP, and the negative F_{ST} values were changed to zeros. Later, the F_{ST} values were smoothed using the *runmed* function in the R program. The F_{ST} values for each breed were estimated.

Haplotype Homozygosity Statistics (H1 and H12):

The haplotypes in each data set were determined using the SHAPEIT2 version program [22]. The conditional states parameter was kept as the default for the phasing of the haplotypes. The SNePv1.1 program computed the effective population size of each breed, and the estimate for each breed was used in the phasing of the chromosomes [23]. A high-resolution Ovine recombination map was used to correct the variations due to the recombination rates at all the autosomes [24]. An R script was used to convert the phased haplotypes to the input files as required in the H12_H1H2.py program. The window size of SNPs was kept to 14, and the step size was 1 (-window 14 -jump 1) [21].

Tajima's D and Nucleotide Diversity (p)

The statistics Tajima's D and (pi) at each coordinate in the dataset of all the studied breeds were computed using the vcftools program [25], and the estimates were computed for each chromosome separately. The nonoverlapping sliding window of 300 MB (-Tajima 300) was used to compute the Tajima's D index. Further, the *p-values* were allotted to the SNPs present in the bin. Meanwhile, the –site-pi function in the vcftools program is employed to compute the pi values of each SNP. The runmed function smooths the *p*-values as outlined in [17].

DCMS Estimation

The stat_to_p-value function in the MINOTAUR package was implemented based on the five statistics, depending on the functional ranks [26]. All five statistic parameters

(H1, H12, Tajima's D index, pi, and FST) for each SNP were combined into a new composite signal as DCMS. The covNAMcd function of the rrcovNA R package (alpha = 0.75, nsamp = 50,000) was utilised to compute a correlation matrix of n x n order [27]. The output thus obtained was given as input for DCMS estimation, and the DCMS values were normalised using the rlm function of the MASS package in the R environment [28] as described in Yurchenko et al. (2018). The [29]A correction that reduces the error rate from multiple tests was applied to q-values transformed from p-values [30].

Identification of Functional Genes and QTL regions

The putative genomic regions were determined based on the q-value. Genomic regions are considered to be putative if the q-values of the SNPs within the boundary are lower than 0.05 and the q-values adjacent to the putative SNPs are more significant than 0.1. The gene and QTL annotation of the candidate genomic regions was carried out using the Genomic Annotation in Livestock for positional candidate Loci (GALLO) package v4.2.0 [31]. The '.gtf' file related to ARS-UI_Ramb_v2.0 of the sheep genome was accessed from NCBI, and the corresponding '.gff' belonged to the ARS-UI_Ramb_v2.0 was downloaded from the Sheep QTLdatabase [32].

The candidate genes and quantitative trait loci (QTLs) located within the significant genomic regions are considered to be putative if they are distributed within an interval of 0.5 Mb upstream and downstream. Further, the QTL enrichment analysis was performed on all the QTLs in the interval through a genome-wide approach. The criteria for QTL enrichment results were based on a false discovery rate (FDR) of less than 0.05.

Results:

Quality Control of genotypic data:

The quality control of genotypic data included the filtering of SNPs with minor allele frequency (maf<0.05), Hardy Weinberg equilibrium of the SNPs (hwe<0.0001),

missingness of the genotypes (geno <0.1) and missingness genotypes (mind <0.1) in the individuals (--mind 0.1). The SNPs excluded for maf, hwe, Geno and mind were , respectively. Further, the SNPs with unknown coordinates on the chromosomes other than the autosomes were removed. The final data set contains 41997 variants used for further downstream analysis.

Principal Component Analysis (PCA)

The principal component analysis (PCA) was employed to ascertain the distinct clusters of SNP genotypes for the three Indian sheep breeds under investigation. The results of PCA are presented in Figure 1. The findings demonstrate the clear delineation of the three populations into separate clusters. The first principal component (PC1) expounded the highest variation of 14.34 % of the total genomic variation. However, the PC2 accounted for 7.98 % of the total variation.

De-Correlated Composite of Multiple Selection Signals (DCMS)

Chantangi sheep:

Gene Annotation:

The DCMS analysis revealed twenty-one putative regions in the Chantangi sheep genome. The total size of the genome under selection was 2871.54 Kb. The mean length of the genome under selection was 136.74±229.77 Kb, which ranged between 1080.32 and 1.83 Kb. These genes were located on BTA 2, 3, 9, 14, 15 and 26. A greater number of significant genomic regions were identified on BTA 15. The gene annotation of the significant genomic regions uncovered twenty-one potential candidate genes related to a variety of traits in this sheep, including *CNTNAP5* (body growth), *MTSS1* (host resistance to bacterial infections), *TRMT12* (immune system and environment adaptation), *TPPP3* (hair follicle growth), *LRRC36* (domestication and early development), *STARD10* (resilience to paratuberculosis), *P2RY6*, *FGF10* and *FAM168A* (hair follicle morphogenesis), *ARHGEF17* and *NIM1K* (meat quality), *GHR* (litter size).

Quantitative Trait Loci (QTL) Annotation and Enrichment:

Table 3 presents the results of QTL annotation in CHA sheep. Our study revealed the significant genomic regions annotated with ten QTLs on OAR 2, 3, 14, 15, and 16. The annotated traits are known to be associated with body weight, milk fat yield, platelet count, total lambs born, and soft tissue depth at the GR site. Further, the QTLs annotated are classified into different classes, and the enrichment of QTLs is shown in Figure 2A. The highest per cent of QTLs annotated in the significant regions of CHA were milk (37.5%), followed by reproduction (34.38%). The other classes of QTLs annotated were meat and carcass (9.38%), health (9.38%) and production (9.38%). The QTL enrichment analysis revealed the top significant QTL total lambs born on OAR 16.

Garole sheep:

Gene Annotation:

The DCMS analysis found eleven (11 No's) potential regions in the Garole sheep genome covering the overall size of the genome, which was 2857.84 Kb. The average size of the genome under selection was found to be 259.80±310.04 Kb. The maximum and minimum size of the genome ranged between 1080.32 and 34.11 Kb. Several putative genes were harboured in this study's significant genomic regions and were situated on OAR 1, 2, 3, 6, 16 and 18. However, OAR 3 anchors more potential candidate genes in the GAR breed. In this study, we identified eleven putative genes associated with a wide range of characteristics, including *BBX* (teat number), *CNTNAP5* (body growth), *C2CD5* (fecundity), *KDM5A* (follicular development), *B4GALNT3* (paratuberculosis resistance), *UNC5C* (litter size), *ADCY2* and *TCERG1L* (reproduction).

Quantitative Trait Loci (QTL) Annotation and Enrichment:

The present study unveils the findings of QTL annotation in the GAR breed, shown in Table 5. Our study revealed the significant genomic regions annotated with twenty-eight QTLs on OAR 1, 2, 3, 6, 18 and 22. The annotated traits are known to be associated with teat number, body weight, milk fat yield, entropion, bone area, total lambs born, body depth, body length, body circumference, shin circumference, body weight, body height, chest width, rump width, lambs born alive and hemonchus contortus resistance.

Furthermore, the annotated QTLs are categorised into different classes, and the enrichment of QTLs is depicted in Figure 2A. GAR's most significant genomic regions had the greater proportion of QTLs associated with health (38.74%) and production (38.74%). The remaining per cent of QTL classes annotated in the breed were reproduction (11.71%), exterior (8.11%), milk (1.8%) and meat and carcass (0.9%). An analysis of QTL enrichment disclosed the three most remarkable QTLs associated with the total lambs born, teat number and entropion on OAR 6, 1 and 3.

Deccani sheep:

Gene Annotation:

The analysis carried out by the DCMS has uncovered fourteen putative regions in the Deccani sheep genome. The genome size under selection was determined to be 2513.30 Kb, with an average length of 167.55±216.86 Kb. The size of the genomic regions under selection pressure ranged from 2.20 to 817.32 Kb. The findings of gene annotation are summarised in Table 4. The gene annotation of the significant genomic regions revealed fourteen potential candidate genes related to a variety of traits in this sheep, including *BUB1* (oocyte and follicle development), *POU2F1* (wool colour), *CHRM2* (hair follicle growth), *DOCK3* (muscle development), *MAPKAPK3* (heat stress response). These genomic regions dock potential candidate genes associated with various traits and are located on OAR 1, 2, 3, 4 and 19, with a maximum number of significant genomic regions on BTA 19.

Quantitative Trait Loci (QTL) Annotation and Enrichment:

The findings outlined in Table 3 detail the results of QTL annotation in IDC sheep. The study has identified significant genomic regions annotated with thirteen QTLs on OAR 1, 2, 3, 4 and 21. The QTLs are linked to the traits viz., somatic cell count, Entropion, Monocyte number, Footrot susceptibility, Pneumonia susceptibility, Body length, Body weight, Water holding capacity, and horn type. Furthermore, the annotated QTLs are sorted into various categories, and the distribution of QTLs is illustrated in Figure 3A. The regions with the most significant impact on the evolvement of the IDC breed had the highest percentage of QTLs annotated for exterior (48.15%), followed by health

(25.93%). The other classes of QTLs annotated were production (18.52%), and meat and carcass (7.41%). The QTL enrichment analysis unveiled the top most significant QTLs on OAR 1,2,3 controlling traits like somatic cell count, horn type, Entropion, Body weight, and Body length (Figure 3B).

Discussion:

This study unveiled comprehensive information about the genomic regions under selection in three Indian sheep breeds adapted to the country's highly harsh climatic conditions. The DCMS analysis uncovered the potential candidate genes linked to various traits of economic importance in sheep. The present investigation used a novel statistic that combines *p*-values obtained from five different univariate statistics into a composite signal that accounts for the correlation among all univariate statistics. However, few studies have used two or more complementary statistics to study the selection signatures in these breeds, and the correlation between the statistics is not appropriately accounted for [13,33]. However, a recent study explored the genomic regions of Indian sheep using four complementary statistics accounting for their absolute correlations between the statistics [34]. This study mainly compared selection signatures in Iranian breeds with a comparison note on Indian and Afghani breeds. This study combined five univariate statistics, such as H1, H12, F_{ST}, TajimaD and Nucleotide diversity, into a composite signal that accounts for correlations between them. It is well documented in the literature that the DCMS method provides absolute and accurate information compared to the results obtained from univariate statistics [6,8,9]. Hence, this study used composite signals to exhaustively study the signatures of the within-breed selections in Indian sheep breeds. As a result, the selection signatures analysis unveiled the potential candidate genes and quantitative traits in the genomes of the studied breeds.

Principal Component Analysis (PCA)

Principal Component Analysis (PCA) is a fundamental underpinning for exploratory data analysis. In short, the PCA computes the eigenvectors from the covariance matrix

of the markers genotyped in the samples. Subsequently, the genotypic coefficients of the principal components will be visualized through plotting to illustrate the clustering resulting from population stratification. Thus, the dimensionality of the huge genotype matrix is reduced to principal components. The distinctive clustering of breeds into three categories, as depicted in the PC plot presented here, was not entirely consistent with the findings of Ahmad et al., 2021. The researchers could not differentiate three sheep breeds into separate clusters based on the first principal component. This could be due to the inclusion of a more significant number of other breeds, representing an additional variation source. The study's findings indicated that the breeds evolved into unique and more specialised breeds owing to the prevailing local climate conditions. In brief, the Garole is a prolific sheep breed found in the hot and humid regions of West Bengal; Chantangi is a woolly sheep distributed in the cold regions of the Himalayas. Likewise, Deccani is well adapted to the arid regions of central India (Nbagr.res.in).

Chantangi sheep:

Gene Annotation:

The sheep production system in India spans a cold climate in the foothills of the Northern Himalayas to a hot, arid climate in Central Peninsular India and hot and humid regions of Eastern India. Based on our findings, none of the identified genes within the detected regions demonstrated significance across all three analysed breeds. However, the potential genomic regions docked the genes linked to body growth, reproduction, fecundity, heat stress response, host resistance to bacterial infections, domestication, environmental adaptation, hair follicle growth, meat quality, and litter size. The study revealed minimal overlap of the genomic regions among the breeds, suggesting breed-specific selection processes in the past.

The significant genomic regions of the CHA breed consist of the genes *CNTNAP5* (body growth), *MTSS1* (host resistance to bacterial infections), *TRMT12* (immune system and environment adaptation), *TPPP3* (reproduction), *LRRC36* (domestication and early

development), *STARD10* (resilience to paratuberculosis), *P2RY6* and *FGF10* (hair follicle morphogenesis), *ARHGEF17* and *NIM1K* (meat quality), and *GHR* (litter size).

The present study found the *CNTNAP5* (Contactin-associated protein-like 5B) in the significant genomic regions harboured on OAR2, a protein-coding gene. This gene belongs to the neurexin family and plays a significant role as a cell adhesion molecule and receptor in the nervous system of most vertebrates. The mechanism of action of *CNTNAP5* is comparable with the brain-derived neurotrophic factor (*BDNF*) gene, which affects the body mass index. Like other epidermal growth factors, it potentially affects growth by enhancing the functions of the brain that have a role in growth regulation [35,36]. The genomic investigation of the morphological traits of Sudanese goats revealed that the *CNTNAP5* gene located on chromosome 2 is associated with the bicoastal diameter [37].

The indigenous sheep of India are known for their resistance to infectious agents. Our findings also identified specific genomic regions in Chantangi sheep that harbour the *MTSS1* gene on chromosome OAR9 related to resistance to bacterial infections. These findings are consistent with the breed characteristics of Chantangi. The bacteria's endotoxins disrupt the endothelial lining in the host cell and cause infection. However, the *MTSS1* gene alters the host endothelium and improves its integrity to avoid infections [38]. The DCMS analysis also revealed significant genomic regions that anchor the *STARD10* gene, reportedly involved in the resistance to paratuberculosis [39]. The study found two essential candidate genes, TRMT12 and LRRC36, associated with environmental adaptation and domestication in sheep. The study on the structural variant landscape in sheep and goats revealed that the LRRC36 gene was involved in the early domestication of sheep and goats [40].

Chantangi sheep are reared mainly by Changpa, a nomadic tribe of the Northern hills, along with the Chantangi goats. This dual-purpose sheep breed, maintained for wool and mutton, contributes significantly to the livelihood of the nomadic tribes. The income of the shepherds mainly was incurred from selling wool and meat. Interestingly, our study highlighted the critical candidate genes associated with hair

follicle development, such as *P2RY6* (OAR 15) and *FGF10* (OAR 16). A gene expression study in mice uncovered the role of *P2RY6* in controlling the MEK1/2–b-catenin signalling pathways and MST–LATS1–YAP signal cascades. It indicated the gene function in the growth and development of keratinocytes [41]. Moreover, gene expression investigation on the hair follicles of coarse sheep fetal skin revealed that the *FGF10* gene is involved in the hair follicle development by the process of competitive adsorption of miR-184 that produces *FGF10* from chi-circRNA-0001141 [42].

We also identified genes under selection in CHA sheep, including *ARHGEF17* and *NIM1K,* which are known to be related to meat quality. Weighted single-step genome-wide association study (GWAS) findings have revealed a significant association between the *ARHGEF17* gene and meat traits within the Chinese yellow-feathered chicken population [43]. Transcriptomic and epigenomic studies in the pectoral muscles in chicken embryos that belong to pure lines revealed the significant role of the *NIM1K* gene in muscle fibre formation [44].

Quantitative Trait Loci (QTL) Annotation and Enrichment:

The DCMS analysis revealed important QTL regions associated with body weight on OAR 2, 3, 14 and 16. The flanking markers or SNPs in the QTL regions, including rs424714738, rs426980328, rs417231209 and rs408124092, respectively. Interestingly, A genome-wide association study in Australian Merino sheep identified a significant QTL region on OAR2 associated with body weight. The study also found an orthologous region on OAR6, similar to human and bovine genomic regions associated with height and weight [45]. Our study also found the same QTL as significant regions in the studied breeds. In a study, Zhang et al., 2016 identified the *MEF2B* and *TRHDE* gene polymorphisms linked to body weight at four months of age in New Ujumqin Sheep Populations located on OAR 3 [46]. The DCMS analysis revealed the same QTL region under selection in the Indian sheep populations. In Baluchi sheep, the genome-wide association study confirmed the location of the QTL peak associated with 8-month body weight [47]. Interestingly, we found the QTL on OAR 14 in the CHA sheep.

Likewise, Armstrong et al., (2018) investigated the genetic polymorphisms in the *GHR* gene and revealed their association with carcass traits in grazing Texel sheep. Interestingly, the same QTL peak was observed in the CHA sheep.

We also identified QTL related to platelet count and milk yield on OAR15 in CHA sheep. Interestingly, Gonzalez et al., (2013) reported the QTL association with mean corpuscular haemoglobin concentration (MCHC, $P=6.2\times10{-}14$) and decreased mean corpuscular volume in Columbia, Polypay, and Ramboulliet sheep. The DCMS analyses also identified the QTL related to the platelet count in the significant genomic regions of CHA sheep. Using three statistical models, the genomic investigation in the crossbred dairy sheep population revealed the QTL regions with the milk yield [50]. We also found the QTL region in the CHA sheep population. We also found the same QTLs annotated in the significant genomic regions of CHA.

Garole sheep:

The Garole sheep is renowned for its petite stature, remarkable fertility, better meat quality and ability to thrive in the saline marshlands of the hot and humid Sundarban region in West Bengal. This breed is also distributed across the southern regions of Bangladesh. Its small size is a result of its adaptation to the harsh climate. Although the genetic potential is relatively limited, these sheep could be raised within a management system requiring minimal inputs (Pan & Sahoo, 2008). This particular breed is highly esteemed due to its fecundity-related genes.

Gene Annotation:

The gene annotation results revealed a few candidate genes associated with essential traits in Garole sheep. We identified the *BBX* gene on OAR 1, which is involved in the supernumerary teats in sheep. Interestingly, a genome-wide association study in Wadi sheep of China reported the candidate genes linked to the supernumerary nipple phenotype in sheep [51]. This study unveiled a few candidate genes related to the trait, including the *BBX* gene. The *BBX* gene is involved in the processes such as cell proliferation and breast tumors. It produces a high mobility group domain (HMG)

transcription factor that can be seen in breast tumors [52]. The DCMS analysis revealed *the CNTNAP5* gene on OAR 2, which regulates the body growth in sheep, and this significant region was also annotated in the CHA breed [37].

In this region, we identified a few candidate genes on OAR 3 in GAR sheep, including the *C2CD5, IQSEC3, KDM5A*, and *B4GALNT3* genes. An expression study in the sheep on oviductal mRNAs and lncRNAs in the follicular and luteal phase uncovered the C2CD5 gene [53]. Our study also identified the C2CD5 gene, which is linked with prolificacy and increased litter size in sheep. This is one of the interesting genes under selection, which is aligned with the demographic history of this sheep breed. Another gene, *KDM5A,* plays a significant role in the zygote genome activation in the cloned embryos in goats [54]. The DCMS analysis also pinpointed the *KDM5A* gene in our study of GAR sheep. We identified another promising candidate gene, *UNC5C,* under selection in GAR sheep. A genome-wide study in Hu sheep confirmed the role of the *UNC5C* gene in litter size in Hu sheep along with the *BMPR1B* gene [55]. According to Sugimoto et al., the *UNC5C* is a protein-coding gene that is a part of the UNC-5 family of netrin receptors and is associated with the conception rate in cattle [56]. Several whole genome studies confirmed the gene role in litter size in Hu sheep [57–59].

Another gene of interest in this study is the ADCY2 gene, known for its pleiotropic effects. It produces cAMP, a signalling molecule in response to G protein signal transduction. This ultimately leads to increased IL-6 formation, a significant change in Brucellosis [60,61]. This gene also plays a significant role in reproduction, as it enhances the expression of estradiol [62]. Interestingly, we identified this gene in our study.

Quantitative Trait Loci (QTL) Annotation and Enrichment:

The DCMS analysis identified several QTL regions associated with teat number, body weight, body measurements, entropion, resistance to the Hemonchus infection, total lambs born, and total lambs alive.

Our study identified the peak QTL region at 201.6 (cM); the markers were rs430157497 and rs426598066. This genomic region on OAR1 surrounding the functional candidate

genes, viz., BBX and CD47, was associated with supernumerary teats in the Wadi sheep of China [51]. One of the significant genomic regions on OAR6 is associated with bone area in Garole sheep. Previous research has identified a genomic region containing putative genes influencing bone density and area in Scottish Blackface lambs through a Genome-Wide Association Study (GWAS) [63].

The Garole sheep is known for its prolificacy and is much valued among breeders owing to its litter size. Few studies confirmed the genomic regions on OAR 6 and 22 that were potentially related to litter size, especially in the Wadi, Hu, Icelandic, Finnsheep, Romanov, and Lori-Bakhtiari sheep breeds [64,65]. The genes in the above potential region, including *BMPR1B*, *CTNNB1*, and *LHCGR*, are known to have a significant effect on litter size in some sheep breeds. Remarkably, the QTL associated with the litter size is consistent with the breeding history of the Garole breed.

The study on Somatostatin Receptor Subtype 1 (SSTR1) Gene Polymorphism and its relation with growth traits in the Hulun Buir Sheep and confirmed their significant association polymorphism [66]. Interestingly, we observed a greater number of QTLs on OAR18 associated with various body measurements such as body weight, length, depth, circumference, rump width, body depth, chest width and shin circumference.

Deccani sheep:

Gene Annotation:

The Deccani breed represents the sole pure black coarse wool breed indigenous to India**.** Our study observed a significant genomic region harbouring a few candidate genes, such as MAEL, APP, and *POU2F1*. The *POU2F1* gene controls melanin production by binding with SLC7A11 and preventing its function [67]. This gene also affects the role of other candidate genes, including SLC7A11, MITF, SLC24A5, MC1R, and ASIP, and thus impacts melanin production and wool colour [68]. The results support the unique traits of the Deccani sheep breed.

Another remarkable region on OAR3 identified in this study consists of several significant genes viz., *MALL, NPHP1, BUB1, IQSEC3* and *ACOXL*. Previous genomic studies on these breeds also confirmed the role of this region in conferring the immune response to several factors [33]. Surprisingly, we also identified the genomic region in our study.

We detected another signature genomic region on OAR2, which comprises the genes controlling sheep growth and fat deposition, including HINT2, RGP1, and *MSMP.* GWAS in Indigenous sheep of Ethiopia revealed a strong association of these genes with fat deposition [69] and growth traits, and a few other studies also reported the same association in sheep [70,71].

In the present study, we identified another significant genomic portion of OAR14 associated with muscle development in cattle. *DOCK3* is a functional candidate gene in the genomic signature region. The molecular study in Bashbay sheep pinpointed the significant role of the *DOCK3* gene in the significant development of skeletal muscle in the specific breed (Zhang et al., 2021).

Quantitative Trait Loci (QTL) Annotation and Enrichment:

The QTL analysis uncovered a few putative signature genomic regions under selection, including those QTLs controlling traits such as somatic cell count, entropion, foot rot and pneumonia susceptibility, monocyte number, body weight, body length, water holding capacity and horn length.

The DCMS analyses identified a significant putative genomic region associated with somatic cell count in this sheep. In a GWAS study, significant associations between SNPs and milk production, composition, coagulation, and cheese traits in Assaf and Churra dairy sheep were reported in the literature; somatic cell count is one of the associated traits in the study [72].

Monocytes are a type of white blood cell formed in the bone marrow. They play a vital role in the body's immune response. Differentiated monocytes play a role in

phagocytosis and antigen presentation, contributing to adaptive immunity. In sheep, monocytes and macrophages act synergistically to counteract the septicemic conditions of Coxiella burnetii and lentivirus [73]. The genome-wide association study in Ramboulliet, Polypay and Columbia sheep identified the novel loci associated with the monocyte count on OAR 1, 2, 3, 4, 9, 10, 15, and 16. The genomic analysis revealed the loci associated with the monocyte count in the Deccani sheep genome on OAR4.

The DCMS analyses identified two other significant loci on chromosome 4 associated with foot rot and pneumonia susceptibility in sheep. Interestingly, the GWAS study in Katahdin, Blackbelly, and European-influenced crossbred sheep determined the strong associations of foot rot susceptibility in hairy sheep [74]. Their study uncovered a few loci on chromosome 4 related to foot rot susceptibility.

The genomic analyses revealed a significant association between body length and body weight on chromosomes 21 and 2. Interestingly, a genome-wide association investigation on Qira Black and German Merino sheep indicated the association between the QTL region with the body measurements and body weight traits [75].

The present study identified two solid genomic associations on chromosome 2 in Deccani sheep related to water-holding capacity (a meat quality trait) and horn type. Deccani sheep are valued among consumers for their better meat quality traits. In addition, the sheep's physical characteristics, especially horns, are unique to this breed. Interestingly, the GWAS on the Colombian Creole hair sheep showed strong associations between the genomic regions on chromosome 2 and the water-holding capacity in meat [76]. Another genomic study demonstrated the genomic basis for the horn polymorphism in wild Soay sheep. The study reported a strong association of the genomic portions on chromosome 2 [77].

Conclusion:

We identified 51 putative QTL regions encompassing 45 protein-coding genes in Indian sheep breeds under selection and adapted to unique and distinct local climates. However, previous studies on the selection signatures of these breeds have been reported using few single statistics. Our study used a novel composite selection

signature method (The de-correlated composite selection signal method) combining *p*-values into a single measure. This method improves the resolution power of the genomic signals and prioritises the functional candidate genes more effectively than single statistics. The putative QTLs and functional candidate genes revealed in the study were based on the historical background of these breeds. The identified signature genomic regions were associated with economic traits such as body growth, fecundity, heat stress response, domestication, environmental adaptation, hair follicle growth, horn type and meat quality yield. Interestingly, this study could pinpoint the genomic regions associated with hair follicle growth in CHA, fecundity and number of lambs born alive in GAR, and meat quality traits in IDC. Our research findings indicate the requisite knowledge to comprehend better the molecular processes implicated in crucial traits and local adaptation. However, whole genome studies with better depth of sequencing, greater population size, and phenotypic observations could uncover the potential candidate genes under selection.

Declarations

Ethics approval and consent to participate

Not applicable

Consent for publication

Not applicable

Availability of data and materials

The genotypic data and other material related to this study are available with the corresponding author. Correspondence can be sent via request mail to satish.vety@gmail.com

Competing interests

The authors declare that they have no competing interests.

Funding

This research received no specific grant from the public, commercial, or not-for-profit funding agencies.

Authors' contributions

SKI and SN conceived the idea, SN, SKI, DWM, SSM, AM and VKY performed the entire bioinformatics analysis, SKI and SN wrote the manuscript, SSM, DWM, AM and VKY edited and proofread the manuscript, and DWM and SKI supervised the whole project. All authors read the manuscript and approved the final manuscript.

Acknowledgements

The first and second authors duly acknowledge the Sri Venkateswara Veterinary University, Tirupati, Andhra Pradesh, for the necessary help and support in conducting this study.

References:

[1] Darwin C. 1859 The origin of species. London: Murray: 1962.

[2] Fay JC, Wu CI. Hitchhiking under positive Darwinian selection. Genetics 2000;155:1405–13. https://doi.org/10.1093/genetics/155.3.1405.

[3] Jensen JD, Foll M, Bernatchez L. The past, present and future of genomic scans for selection. Mol Ecol 2016. https://doi.org/10.1111/mec.13493.

[4] Qanbari S, Simianer H. Mapping signatures of positive selection in the genome of livestock. Livest Sci 2014;166:133–43. https://doi.org/10.1016/j.livsci.2014.05.003.

[5] Illa SK, Mukherjee S, Nath S, Mukherjee A. Genome-Wide Scanning for Signatures of Selection Revealed the Putative Genomic Regions and Candidate Genes Controlling Milk Composition and Coat Color Traits in Sahiwal Cattle 2021;12:1–14. https://doi.org/10.3389/fgene.2021.699422.

[6] Grossman SR, Shylakhter I, Karlsson EK, Byrne EH, Morales S, Frieden G, et al. A composite of multiple signals distinguishes causal variants in regions of positive selection. Science (80-) 2010. https://doi.org/10.1126/science.1183863.

[7] Staubach F, Lorenc A, Messer PW, Tang K, Petrov DA, Tautz D. Genome Patterns of Selection and Introgression of Haplotypes in Natural Populations of the House Mouse (Mus musculus). PLoS Genet 2012;8. https://doi.org/10.1371/journal.pgen.1002891.

[8] Ma Y, Ding X, Qanbari S, Weigend S, Zhang Q, Simianer H. Properties of different selection signature statistics and a new strategy for combining them. Heredity (Edinb) 2015. https://doi.org/10.1038/hdy.2015.42.

[9] Lotterhos KE, Card DC, Schaal SM, Wang L, Collins C, Verity B. Composite measures of selection can improve the signal-to-noise ratio in genome scans. Methods Ecol Evol 2017. https://doi.org/10.1111/2041-210X.12774.

[10] Kijas JW, Lenstra JA, Hayes B, Boitard S, Neto LR, Cristobal MS, et al. Genome-wide analysis of the world's sheep breeds reveals high levels of historic mixture and strong recent selection. PLoS Biol 2012;10.

https://doi.org/10.1371/journal.pbio.1001258.

[11] DADHF. Annual report. 2023.

[12] Saravanan KA, Panigrahi M, Kumar H, Parida S, Bhushan B, Gaur GK, et al. Genomic scans for selection signatures revealed candidate genes for adaptation and production traits in a variety of cattle breeds. Genomics 2021;113:955–63. https://doi.org/10.1016/j.ygeno.2021.02.009.

[13] Ahmad SF, Mehrotra A, Charles S, Ganai NA. Analysis of selection signatures reveals important insights into the adaptability of high-altitude Indian sheep breed Changthangi. Gene 2021;799:145809. https://doi.org/10.1016/j.gene.2021.145809.

[14] Sempéré G, Moazami-Goudarzi K, Eggen A, Laloë D, Gautier M, Flori L. WIDDE: A Web-Interfaced next generation database for genetic diversity exploration, with a first application in cattle. BMC Genomics 2015;16:1–8. https://doi.org/10.1186/s12864-015-2181-1.

[15] Chang CC, Chow CC, Tellier LCAM, Vattikuti S, Purcell SM, Lee JJ. Second-generation PLINK: Rising to the challenge of larger and richer datasets. Gigascience 2015;4:1–16. https://doi.org/10.1186/s13742-015-0047-8.

[16] Zheng X, Levine D, Shen J, Gogarten SM, Laurie C, Weir BS. A high-performance computing toolset for relatedness and principal component analysis of SNP data. Bioinformatics 2012. https://doi.org/10.1093/bioinformatics/bts606.

[17] Yurchenko AA, Daetwyler HD, Yudin N, Schnabel RD, Vander Jagt CJ, Soloshenko V, et al. Scans for signatures of selection in Russian cattle breed genomes reveal new candidate genes for environmental adaptation and acclimation. Sci Rep 2018;8:1–16. https://doi.org/10.1038/s41598-018-31304-w.

[18] Nei M, Li WH. Mathematical model for studying genetic variation in terms of restriction endonucleases. Proc Natl Acad Sci U S A 1979. https://doi.org/10.1073/pnas.76.10.5269.

[19] Weir BS, Cockerham CC. Estimating F-statistics for the analysis of population structure. Evolution (N Y) 1984. https://doi.org/10.1111/j.1558-5646.1984.tb05657.x.

[20] Tajima F. Statistical method for testing the neutral mutation hypothesis by DNA polymorphism. Genetics 1989.

[21] Garud NR, Messer PW, Buzbas EO, Petrov DA. Recent Selective Sweeps in North American Drosophila melanogaster Show Signatures of Soft Sweeps. PLoS Genet 2015. https://doi.org/10.1371/journal.pgen.1005004.

[22] Delaneau O, Marchini J, Zagury JF. A linear complexity phasing method for thousands of genomes. Nat Methods 2012. https://doi.org/10.1038/nmeth.1785.

[23] Barbato M, Orozco-terWengel P, Tapio M, Bruford MW. SNeP: A tool to estimate trends in recent effective population size trajectories using genome-wide SNP data. Front Genet 2015. https://doi.org/10.3389/fgene.2015.00109.

[24] Petit M, Astruc JM, Sarry J, Drouilhet L, Fabre S, Moreno CR, et al. Variation in recombination rate and its genetic determinism in sheep populations. Genetics 2017;207:767–84. https://doi.org/10.1534/genetics.117.300123.

[25] Danecek P, Auton A, Abecasis G, Albers CA, Banks E, DePristo MA, et al. The variant call format and VCFtools. Bioinformatics 2011. https://doi.org/10.1093/bioinformatics/btr330.

[26] Verity R, Collins C, Card D, Schaal S, Wang L, Lotterhos K. MINOTAUR: A platform for the analysis and visualization of multivariate results from genome scans with R Shiny. Minotaur A Platf Anal Vis Multivar Results from Genome Scans with R Shiny 2016. https://doi.org/10.1101/062158.

[27] Todorov V, Templ M, Filzmoser P. Detection of multivariate outliers in business survey data with incomplete information. Adv Data Anal Classif 2011. https://doi.org/10.1007/s11634-010-0075-2.

[28] Venables WN, Ripley BD. Modern Applied Statistics with S Fourth edition by. 2002.

[29] Hochberg YB and Y. Controlling the False Discovery Rate: A Practical and Powerful Approach to Multiple Testing. J R Stat Soc 1995;57:289–300.

[30] Storey JD, Tibshirani R. Statistical significance for genomewide studies. Proc Natl Acad Sci U S A 2003. https://doi.org/10.1073/pnas.1530509100.

[31] Fonseca PAS, Suárez-Vega A, Marras G, Cánovas Á. GALLO: An R package for

genomic annotation and integration of multiple data sources in livestock for positional candidate loci. Gigascience 2020. https://doi.org/10.1093/gigascience/giaa149.

[32] Hu ZL, Park CA, Reecy JM. Building a livestock genetic and genomic information knowledgebase through integrative developments of Animal QTLdb and CorrDB. Nucleic Acids Res 2019. https://doi.org/10.1093/nar/gky1084.

[33] Saravanan KA, Panigrahi M, Kumar H, Bhushan B, Dutt T, Mishra BP. Genome-wide analysis of genetic diversity and selection signatures in three Indian sheep breeds. Livest Sci 2021;243:104367. https://doi.org/10.1016/j.livsci.2020.104367.

[34] Eydivandi S, Roudbar MA, Karimi MO, Sahana G. Genomic scans for selective sweeps through haplotype homozygosity and allelic fixation in 14 indigenous sheep breeds from Middle East and South Asia. Sci Rep 2021;11:1–18. https://doi.org/10.1038/s41598-021-82625-2.

[35] Hirschhorn J, Ingelsson E, Genet N. Association analyses of 249,796 individuals reveal eighteen new loci associated with body mass index HHS Public Access Author manuscript 2011;42:937–48. https://doi.org/10.1038/ng.686.Association.

[36] Zielke LG, Bortfeldt RH, Tetens J, Brockmann GA. BDNF contributes to the genetic variance of milk fat yield in German Holstein cattle. Front Genet 2011;2:1–8. https://doi.org/10.3389/fgene.2011.00016.

[37] Rahmatalla SA, Arends D, Reissmann M, Wimmers K, Reyer H, Brockmann GA.

Genome-wide association study of body morphological traits in Sudanese goats. Anim Genet 2018;49:478–82. https://doi.org/10.1111/age.12686.

[38] Duan Y, Su P, Gu Y, Lv X, Cao X, Wang S, et al. A Study of the Resistance of Hu Sheep Lambs to Escherichia coli F17 Based on Whole Genome Sequencing. Animals 2024;14:1–12. https://doi.org/10.3390/ani14010161.

[39] Purdie AC, Plain KM, Begg DJ, de Silva K, Whittington RJ. Gene expression profiles during subclinical Mycobacterium avium subspecies paratuberculosis infection in sheep can predict disease outcome. Sci Rep 2019;9:1–15. https://doi.org/10.1038/s41598-019-44670-w.

[40] Yang J, Wang DF, Huang JH, Zhu QH, Luo LY, Lu R, et al. Structural variant landscapes reveal convergent signatures of evolution in sheep and goats. Genome Biol 2024;25:1–46. https://doi.org/10.1186/s13059-024-03288-6.

[41] Xu P, Wang C, Xiang W, Liang Y, Li Y, Zhang X, et al. P2RY6 Has a Critical Role in Mouse Skin Carcinogenesis by Regulating the YAP and β-Catenin Signaling Pathways. J Invest Dermatol 2022;142:2334-2342.e8. https://doi.org/10.1016/j.jid.2022.02.017.

[42] Tian D, Pei Q, Jiang H, Guo J, Ma X, Han B, et al. Comprehensive analysis of the expression profiles of mRNA, lncRNA, circRNA, and miRNA in primary hair follicles of coarse sheep fetal skin. BMC Genomics 2024;25:1–15. https://doi.org/10.1186/s12864-024-10427-7.

[43] Pan R, Qi L, Xu Z, Zhang D, Nie Q, Zhang X, et al. Weighted single-step GWAS identified candidate genes associated with carcass traits in a Chinese yellow-feathered chicken population. Poult Sci 2024;103:1–14. https://doi.org/10.1016/j.psj.2023.103341.

[44] Gu S, Huang Q, Sun C, Wen C, Yang N. Transcriptomic and epigenomic insights into pectoral muscle fiber formation at the late embryonic development in pure chicken lines. Poult Sci 2024;103:103882. https://doi.org/10.1016/j.psj.2024.103882.

[45] Al-Mamun HA, Kwan P, Clark SA, Ferdosi MH, Tellam R, Gondro C. Genome-wide association study of body weight in Australian Merino sheep reveals an orthologous region on OAR6 to human and bovine genomic regions affecting height and weight. Genet Sel Evol 2015;47:1–11. https://doi.org/10.1186/s12711-015-0142-4.

[46] Zhang L, Ma X, Xuan J, Wang H, Yuan Z, Wu M, et al. Identification of MEF2B and TRhDE gene polymorphisms related to growth traits in a new ujumqin sheep population. PLoS One 2016;11:1–12. https://doi.org/10.1371/journal.pone.0159504.

[47] Pasandideh M, Gholizadeh M, Rahimi-Mianji G. A genome-wide association study revealed five SNPs affecting 8-month weight in sheep. Anim Genet 2020;51:973–6. https://doi.org/10.1111/age.12996.

[48] Armstrong E, Ciappesoni G, Iriarte W, Da Silva C, Macedo F, Navajas EA, et al. Novel genetic polymorphisms associated with carcass traits in grazing Texel sheep. Meat Sci 2018;145:202–8. https://doi.org/10.1016/j.meatsci.2018.06.014.

[49] Gonzalez M V., Mousel MR, Herndon DR, Jiang Y, Dalrymple BP, Reynolds JO, et al. A Divergent Artiodactyl MYADM-like Repeat Is Associated with Erythrocyte Traits and Weight of Lamb Weaned in Domestic Sheep. PLoS One 2013;8. https://doi.org/10.1371/journal.pone.0074700.

[50] Li H, Wu XL, Tait RG, Bauck S, Thomas DL, Murphy TW, et al. Genome-wide association study of milk production traits in a crossbred dairy sheep population using three statistical models. Anim Genet 2020;51:624–8. https://doi.org/10.1111/age.12956.

[51] Peng WF, Xu SS, Ren X, Lv FH, Xie XL, Zhao YX, et al. A genome-wide association study reveals candidate genes for the supernumerary nipple phenotype in sheep (Ovis aries). Anim Genet 2017;48:570–9. https://doi.org/10.1111/age.12575.

[52] Wen J, Toomer KH, Chen Z, Cai X. Genome-wide analysis of alternative transcripts in human breast cancer. Breast Cancer Res Treat 2015;151:295–307. https://doi.org/10.1007/s10549-015-3395-2.

[53] Chen W, Li Z, Zhong R, Sun W, Chu M. Expression profiles of oviductal mRNAs and lncRNAs in the follicular phase and luteal phase of sheep (Ovis aries) with 2

fecundity gene (FecB) genotypes. G3 Genes, Genomes, Genet 2024;14:1–16. https://doi.org/10.1093/g3journal/jkad270.

[54] Deng M, Zhang G, Cai Y, Liu Z, Zhang Y, Meng F, et al. DNA methylation dynamics during zygotic genome activation in goat. Theriogenology 2020;156:144–54. https://doi.org/10.1016/j.theriogenology.2020.07.008.

[55] Bao J, Xiong J, Huang J, Yang P, Shang M, Zhang L. Genetic Diversity, Selection Signatures, and Genome-Wide Association Study Identify Candidate Genes Related to Litter Size in Hu Sheep. Int J Mol Sci 2024;25. https://doi.org/10.3390/ijms25179397.

[56] Sugimoto M, Gotoh Y, Kawahara T, Sugimoto Y. Molecular effects of polymorphism in the 3'UTR of unc-5 homolog C associated with conception rate in Holsteins. PLoS One 2015;10:1–14. https://doi.org/10.1371/journal.pone.0131283.

[57] Zhao F, Xie R, Fang L, Xiang R, Yuan Z, Liu Y, et al. Analysis of 206 whole-genome resequencing reveals selection signatures associated with breed-specific traits in Hu sheep. Evol Appl 2024;17:1–12. https://doi.org/10.1111/eva.13697.

[58] Zhong T, Hou D, Zhao Q, Zhan S, Wang L, Li L, et al. Comparative whole-genome resequencing to uncover selection signatures linked to litter size in Hu Sheep and five other breeds. BMC Genomics 2024;25:1–13. https://doi.org/10.1186/s12864-024-10396-x.

[59] Eydivandi S, Roudbar MA, Ardestani SS, Momen M, Sahana G. A selection signatures study among Middle Eastern and European sheep breeds. J Anim Breed Genet 2021;138:574–88. https://doi.org/10.1111/jbg.12536.

[60] Karaoglan I, Pehlivan S, Namiduru M, Pehlivan M, Kilinçarslan C, Balkan Y, et al. TNF-α, TGF-β, IL-10, IL-6 and IFN-γ gene polymorphisms as risk factors for brucellosis. New Microbiol 2009;32:173–8.

[61] Li X, Wu Q, Zhang X, Li C, Zhang D, Li G, et al. Whole-Genome Resequencing to Study Brucellosis Susceptibility in Sheep. Front Genet 2021;12:1–9. https://doi.org/10.3389/fgene.2021.653927.

[62] Zhao G, Li X, Miao H, Chen S, Hou Y. Estrogen promotes cAMP production in Mesenchymal stem cells by regulating ADCY2. Int J Stem Cells 2020;13:55–64. https://doi.org/10.15283/IJSC19139.

[63] Matika O, Riggio V, Anselme-Moizan M, Law AS, Pong-Wong R, Archibald AL, et al. Genome-wide association reveals QTL for growth, bone and in vivo carcass traits as assessed by computed tomography in Scottish Blackface lambs. Genet Sel Evol 2016;48:1–15. https://doi.org/10.1186/s12711-016-0191-3.

[64] Xu SS, Gao L, Xie XL, Ren YL, Shen ZQ, Wang F, et al. Genome-wide association analyses highlight the potential for different genetic mechanisms for litter size among sheep breeds. Front Genet 2018;9:1–14. https://doi.org/10.3389/fgene.2018.00118.

[65] Abdoli R, Mirhoseini SZ, Ghavi Hossein-Zadeh N, Zamani P, Moradi MH, Ferdosi MH, et al. Runs of homozygosity and cross-generational inbreeding of Iranian fat-tailed sheep. Heredity (Edinb) 2023;130:358–67. https://doi.org/10.1038/s41437-023-00611-y.

[66] Li X, Ding N, Zhang Z, Tian D, Han B, Liu S, et al. Identification of Somatostatin Receptor Subtype 1 (SSTR1) Gene Polymorphism and Their Association with Growth Traits in Hulun Buir Sheep. Genes (Basel) 2022;13. https://doi.org/10.3390/genes13010077.

[67] Chen Y, Hu S, Mu L, Zhao B, Wang M, Yang N, et al. Slc7a11 modulated by POU2f1 is involved in pigmentation in rabbit. Int J Mol Sci 2019;20:1–16. https://doi.org/10.3390/ijms20102493.

[68] Yang N, Zhao B, Hu S, Bao Z, Liu M, Chen Y, et al. Characterization of POU2F1 gene and its potential impact on the expression of genes involved in fur color formation in rex rabbit. Genes (Basel) 2020;11. https://doi.org/10.3390/genes11050575.

[69] Ahbara A, Bahbahani H, Almathen F, Abri M Al, Agoub MO, Abeba A, et al. Genome-wide variation, candidate regions and genes associated with fat deposition and tail morphology in Ethiopian indigenous sheep. Front Genet 2019;10:1–21. https://doi.org/10.3389/fgene.2018.00699.

[70] Casas E, Shackelford SD, Keele JW, Stone RT, Kappes SM, Koohmaraie M.

Quantitative trait loci affecting growth and carcass composition of cattle segregating alternate forms of myostatin. J Anim Sci 2000;78:560–9. https://doi.org/10.2527/2000.783560x.

[71] McClure MC, Morsci NS, Schnabel RD, Kim JW, Yao P, Rolf MM, et al. A genome scan for quantitative trait loci influencing carcass, post-natal growth and reproductive traits in commercial Angus cattle. Anim Genet 2010;41:597–607. https://doi.org/10.1111/j.1365-2052.2010.02063.x.

[72] Marina H, Pelayo R, Suárez-Vega A, Gutiérrez-Gil B, Esteban-Blanco C, Arranz JJ. Genome-wide association studies (GWAS) and post-GWAS analyses for technological traits in Assaf and Churra dairy breeds. J Dairy Sci 2021;104:11850–66. https://doi.org/10.3168/jds.2021-20510.

[73] Oliveira RD, Mousel MR, Gonzalez M V., Durfee CJ, Davenport KM, Murdoch BM, et al. A high-density genome-wide association with absolute blood monocyte count in domestic sheep identifies novel loci. PLoS One 2022;17:1–15. https://doi.org/10.1371/journal.pone.0266748.

[74] Cinar MU, Oliveira RD, Hadfield TS, Lichtenwalner A, Brzozowski RJ, Settlemire CT, et al. Genome-wide association with footrot in hair and wool sheep. Front Genet 2023;14:1–9. https://doi.org/10.3389/fgene.2023.1297444.

[75] Tuersuntuoheti M, Zhang J, Zhou W, Zhang CL, Liu C, Chang Q, et al. Exploring the growth trait molecular markers in two sheep breeds based on Genome-wide

association analysis. PLoS One 2023;18:1–18. https://doi.org/10.1371/journal.pone.0283383.

[76] Revelo HA, López-Alvarez D, Palacios YA, Vergara OD, Yánez MB, Ariza MF, et al. Genome-wide association study reveals candidate genes for traits related to meat quality in Colombian Creole hair sheep. Trop Anim Health Prod 2023;55:1–13. https://doi.org/10.1007/s11250-023-03688-z.

[77] Johnston SE, McEwan JC, Pickering NK, Kijas JW, Beraldi D, Pilkington JG, et al. Genome-wide association mapping identifies the genetic basis of discrete and quantitative variation in sexual weaponry in a wild sheep population. Mol Ecol 2011;20:2555–66. https://doi.org/10.1111/j.1365-294X.2011.05076.x.

Table1 shows the genomic regions of Indian sheep breeds identified as being under selection through DCMS analysis

S.No.	Breed	Total Size	Mean ±	Maximum	Minimum	Total

		(kb)	Standard Deviation (Kb)	Length (kb)	Length (kb)	No. of genes
01	Chantangi (CHA)	2871.54	136.74±229.77	1080.32	1.83	21
02	Garole (GAR)	2857.84	259.80±310.04	1080.32	34.11	10
03	Deccani (IDC)	2513.30	167.55±216.86 Kb	817.32	2.20	14

Table2 shows the results of gene annotation in significant genomic regions in CHANTANGI sheep

S.No.	CHR	BP	START COORDINATE	END COORDINATE	Width	Strand	Gene Name
1	2	190267950	189526649	190606970	1080322	+	*CNTNAP5*
2	3	106221882	106222211	106279403	57193	+	*FBLN7*
3	3	107958569	107917450	107989336	71887	+	*TBC1D15*
4	9	28518494	28426199	28586681	160483	+	*MTSS1*
5	9	28610511	28596736	28628449	31714	+	*TATDN1*
6	9	28651053	28650081	28651912	1832	-	*TRMT12*
7	9	28651053	28627373	28650325	22953	-	*RNF139*
8	14	35090205	35080140	35173351	93212	+	*NFATC3*
9	14	34566935	34510632	34569231	58600	-	*TPPP3*
10	14	34566935	34504860	34579222	74363	+	*LRRC36*
11	15	51070453	51028830	51075998	47169	-	*STARD10*
12	15	51107183	51099187	51353916	254730	-	*FCHSD2*
13	15	51474825	51404925	51511918	106994	+	*P2RY2*
14	15	51517148	51481919	51516379	34461	+	*P2RY6*
15	15	51578641	51521915	51579398	57484	+	*ARHGEF17*
16	15	51591704	51585751	51604071	18321	+	*RELT*
17	15	51632422	51613275	51814944	201670	-	*FAM168A*
18	16	30667706	30604422	30702581	98160	+	*FGF10*
19	16	31588015	31575745	31651791	76047	-	*NIM1K*
20	16	31687647	31660770	31688311	27542	-	*ZNF131*
21	16	32196927	32069778	32366181	296404	-	*GHR*

Table4 shows the gene annotation results in significant genomic regions in GAROLE sheep.

S.No.	CHR	BP	START COORDINATE	END COORDINATE	Width	Strand	gene_name

1	1	173424721	173196985	173483458	286474	+	*BBX*
2	2	189539157	189526649	190606970	1080322	+	*CNTNAP5*
3	3	193287569	193251622	193339539	87918	+	*C2CD5*
4	3	212967581	212887405	212973893	86489	+	*IQSEC3*
5	3	213086338	213068112	213128627	60516	-	*KDM5A*
6	3	213195386	213175874	213273093	97220	+	*B4GALNT3*
7	6	29809690	29597358	30017600	420243	+	*UNC5C*
8	16	65681259	65471620	65927572	455953	-	*ADCY2*
9	18	46100361	46097255	46131370	34116	-	*CLEC14A*
10	22	49602008	49599602	49793506	193905	-	*TCERG1L*

Table5 shows the gene annotation results in significant genomic regions in DECCANI sheep.

S.No	CHR	BP	START COORDINATE	END COORDINATE	width	strand	Gene Name
1	1	119053600	119027142	119081828	54687	+	*MAEL*
2	1	131092601	130838877	131151750	312874	+	*APP*
3	1	119491972	119428361	119624526	196166	+	*POU2F1*
4	3	104706333	104671436	104716760	45325	-	*MALL*
5	3	104775041	104716342	104784266	67925	-	*NPHP1*
6	3	104885280	104870169	104915494	45326	-	*BUB1*
7	3	212967581	212887405	212973893	86489	+	*IQSEC3*
8	3	104917562	104914948	105286674	371727	+	*ACOXL*
9	4	101967194	101379345	102196659	817315	+	*CHRM2*
10	19	49657211	49447036	49738575	291540	-	*DOCK3*
11	19	49778830	49759229	49789524	30296	-	*MAPKAPK3*
12	2	52731947	52731991	52734550	2560	+	*HINT2*
13	2	52790093	52790313	52801134	10822	-	*RGP1*
14	2	52790093	52790904	52793100	2197	+	*MSMP*

Table6 shows the Quantitative Trait Loci annotation results in significant genomic regions in CHANTANGI sheep.

CHR	BP	START COORDINATE	END COORDINATE	Name	Flank Marker	P-value
2	190267950	190457084	190457088	Body weight	rs424714738	1.61E-03
3	107991182	108401221	108401225	Body weight	rs426980328	0.026
14	35090205	35417680	35417684	Body weight	rs417231209	<0.05

15	51070453	50955799	50955803	Milk fat yield	rs404288918	0.0006245
15	51474825	51832265	51832269	Platelet count	rs413264572	5.50E-06
16	31588015	32070073	32070077	Total lambs born	rs161146164	<0.05
16	31654423	32118461	32118465	Total lambs born	rs426666828	<0.05
16	32569758	32119256	32119260	Milk fat yield	rs422431427	0.00031338
16	32569758	32192693	32192697	Body weight	rs408124092	<0.0001
16	32569758	32192693	32192697	Soft tissue depth at GR site	rs408124092	0.0014

Table7 shows the Quantitative Trait Loci annotation results in significant genomic regions in GAROLE sheep.

CHR	BP	START COORDINATE	END COORDINATE	Name	Flank Marker	P-value
1	173424721	173432597	173432601	Teat number	rs430157497	<0.05
1	173424721	173534798	173534802	Teat number	rs426598066	<0.05
2	190019411	190457084	190457088	Body weight	rs424714738	1.61E-3
3	192453314	192442586	192442590	Entropion	rs411640492	3.19E-04
3	192935483	192442586	192442590	Entropion	rs411640492	3.19E-04
3	193336800	193016493	193016497	Entropion	rs404605277	1.12E-04
3	212967581	213021781	213021785	Entropion	rs159913124	3.61E-04
3	213209196	213213768	213213772	Entropion	rs418752307	2.81E-04
6	29809690	29344134	29344138	Bone area	rs418089550	1.12E-5
6	29809690	29366036	29366040	Milk fat yield	rs424558688	0.036982472
6	29847992	30096782	30096786	Total lambs born	rs421635584	<0.05
18	46159259	46086980	46086984	Body weight	rs426187704	<0.05
18	46100361	46086980	46086984	Body depth	rs426187704	<0.01
18	46062053	46086980	46086984	Body weight	rs426187704	<0.05
18	46062053	46086980	46086984	Body length	rs426187704	<0.05
18	46159259	46086980	46086984	Body circumference	rs426187704	<0.05
18	46159259	46086980	46086984	Body depth	rs426187704	<0.01
18	46159259	46086980	46086984	Rump width	rs426187704	<0.05
18	46159259	46087004	46087008	Body weight	rs404696179	<0.05
18	46159259	46087004	46087008	Body length	rs404696179	<0.01
18	46100361	46087004	46087008	Body height	rs404696179	<0.01
18	46159259	46087004	46087008	Body circumference	rs404696179	<0.05
18	46062053	46087004	46087008	Body height	rs404696179	<0.05
18	46159259	46087646	46087650	Chest width	rs405457403	<0.05
18	46159259	46087646	46087650	Shin	rs405457403	<0.05

				circumference		
18	46159259	46087646	46087650	Body depth	rs405457403	<0.05
18	47232764	47716852	47716856	Haemonchus contortus resistance	rs428856771	3.23E-4
22	49796078	49873766	49873770	Lambs born alive	rs402605786	9.79E-3

Table8 shows the Quantitative Trait Loci annotation results in significant genomic regions in DECCANI sheep.

CHR	BP	start_pos	end_pos	Name	FlankMarker	P-value
1	131092601	130616460	130616464	somatic cell count	rs409795528	<0.05
3	212967581	213021781	213021785	Entropion	rs159913124	3.61E-04
3	212967581	213213768	213213772	Entropion	rs418752307	2.81E-04
4	101967194	101960285	101960289	Monocyte number	rs399619443	2.55E-6
4	101967194	102026928	102026932	Footrot susceptibility	rs416121047	2.84E-6
4	101967194	102039511	102039515	Pneumonia susceptibility	rs403394816	<0.10
21	41665172	41243033	41243037	Body length	rs406947061	<0.05
2	52731947	52720586	52720590	Body weight	rs426272889	<0.05
2	52790093	52737532	52737536	Body weight	rs160159557	<0.05
2	52790093	52848103	52848107	Water holding capacity	rs420647640	<0.05
2	116594826	116133033	116133037	Horn type	rs416045857	<0.05
2	116594826	116839657	116839661	Horn type	rs405044765	<0.05
2	116953479	116872298	116872302	Horn type	rs402400571	<0.05

Figure1: PCA analysis showing the population structure of the three Indian sheep breeds.

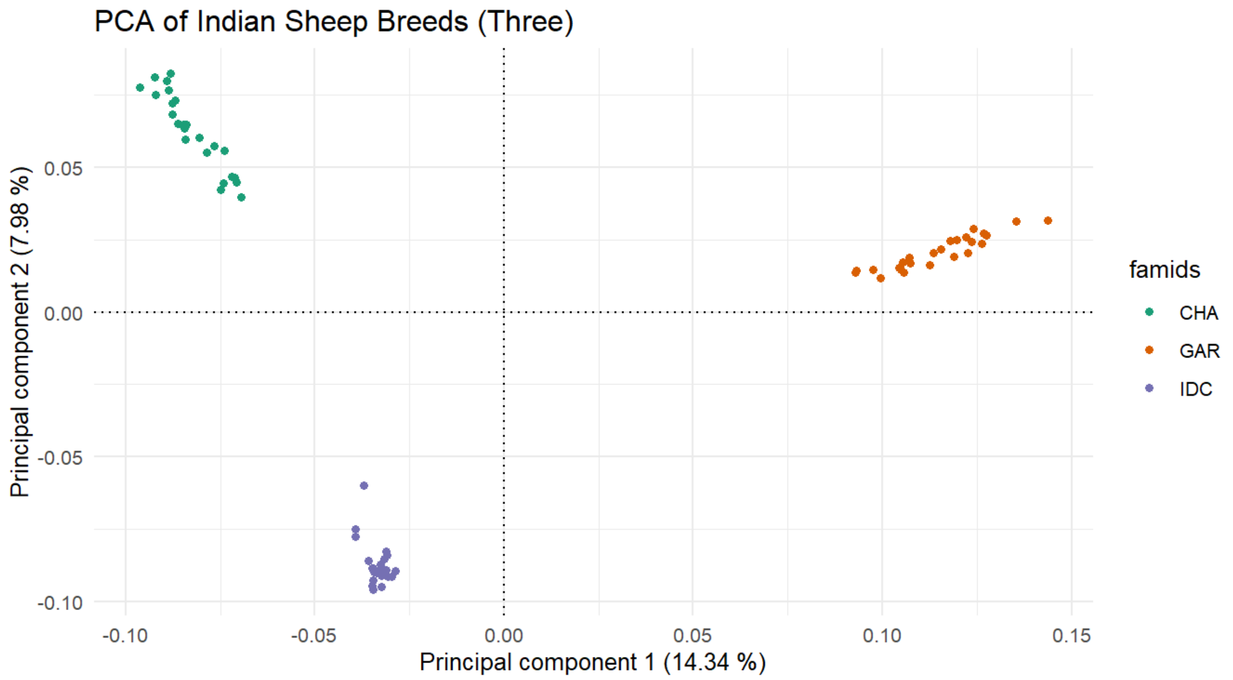
PCA of Indian Sheep Breeds (Three)
Principal component 2 (7.98 %)
0.05
0.00
-0.05
-0.10
-0.10
-0.05
0.00
0.05
0.10
0.15
Principal component 1 (14.34 %)
famids
CHA
GAR
IDC

Figure 2 Percent QTL classes annotated in three Indian sheep breeds

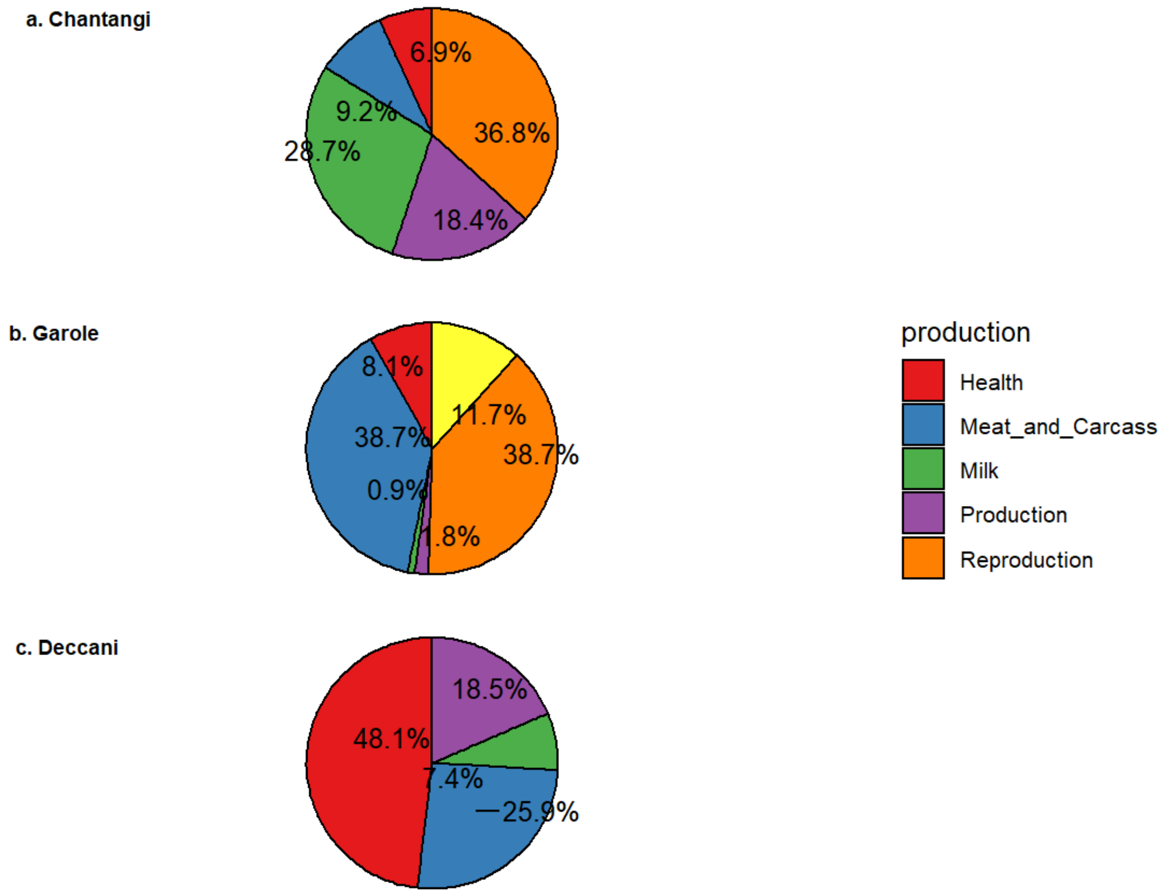

Figure 3 QTL classes enriched in three Indian sheep breeds

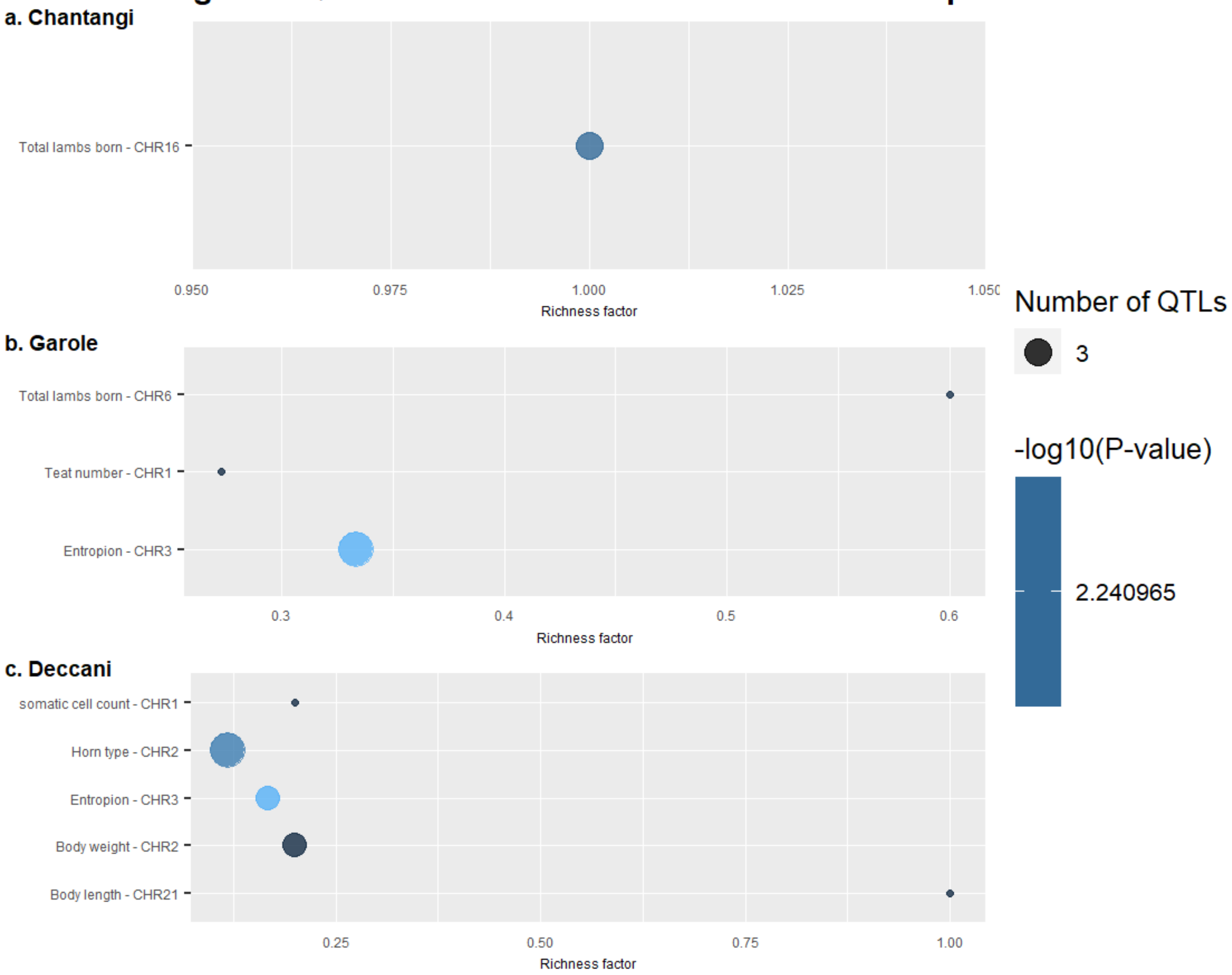

Supplement: Attachment — Submitted filename: Reviewed manuscript-PONE-D-24-51732_reviewer.pdf [file pone.0344299.s001.pdf]
