# Supplementary material for: Composite selection signal analysis: Uncovering candidate genes and quantitative trait loci in Indian sheep breeds
Source: PLoS One. 2026 Mar 25;21(3):e0344299. doi: 10.1371/journal.pone.0344299 (PMC13016353; doi:10.1371/journal.pone.0344299)
Supplement: Attachment — Submitted filename: Reviewed PONE-D-24-51732_R1_reviewer.pdf [file pone.0344299.s005.pdf]

PLOS ONE

Composite Selection Signal Analysis: Uncovering Candidate Genes and Quantitative Trait Loci in Indian Sheep Breeds

--Manuscript Draft--

**Manuscript Number:**	PONE-D-24-51732R1
**Article Type:**	Research Article
**Full Title:**	Composite Selection Signal Analysis: Uncovering Candidate Genes and Quantitative Trait Loci in Indian Sheep Breeds
**Short Title:**	Composite Selection Signal Analysis
**Corresponding Author:**	Destaw Worku Worku<br>Injibara University<br>Injibara, Amhara ETHIOPIA
**Keywords:**	DCMS; Indian Sheep; SNP; QTL; Selection Signature
**Abstract:**	Natural and artificial selection are fundamental demographic forces that contribute significantly to the shaping of genomes. Exploring the molecular mechanisms implicated in the adaptation processes can assist in creating efficient breeding programs designed to preserve and improve the genetic characteristics of native sheep. The study used a novel method that combines the p-values into a single measure to investigate the genomic regions under selection. This method improves the resolution power and prioritises the candidate genes more efficiently than other single statistics. The 50K SNP data on three Indigenous sheep breeds (n=79) were Chantangi (CHA, n= 29), Garole (GAR, n=24), and Deccani (IDC, n=26) native to diverse regions of India were used in this study. The study combined five statistics into a composite selection signal called a De-Correlated composite of multiple signals (DCMS). The p-values of all the single statistics were corrected for multiple testing at the False Discovery Rate of < 5%, and the putative genomic regions were detected. Gene and quantitative trait loci (QTL) annotation of the significant regions was performed. The findings included Novel putative genes related to a wide variety of characteristics, including MTSS1 (host resistance to bacterial infections), TRMT12 (environment adaptation), TPPP3, P2RY6, FGF10 and FAM168A (hair follicle growth), LRRC36 (domestication), BBX (teat number), CNTNAP5 (body growth), C2CD5 and UNC5C (fecundity), B4GALNT3 (paratuberculosis resistance), TCERG1L (reproduction), BUB1 (oocyte and follicle development), POU2F1 (wool colour), DOCK3 (muscle development), MAPKAPK3 (heat stress response). The QTLs identified are associated with milk fat yield, platelet count, entropion, bone area, total lambs born, body measurements, hemonchus resistance, foot rot and pneumonia susceptibility and entropion. The DCMS method pinpointed novel genomic regions and putative genes more efficiently than other single statistics and could provide reliable comprehension of the molecular mechanisms involved in all the essential traits in the Indian sheep.
**Order of Authors:**	Destaw Worku
	Sapna Nath
	Satish Kumar Illa
	Sabyasachi Mukherjee
	Anupama Mukherjee
	Vinod Kumar Yata
**Response to Reviewers:**	Manuscript Number: PONE-D-24-51732<br>Title: Composite Selection Signal Analysis: Uncovering Candidate Genes and Quantitative Trait Loci in Indian Sheep Breeds<br>S.No.Reviewers CommentsResponse to Reviewer’s Comments<br>01Certain paragraphs in the introduction and discussion are redundant and could be condensed.Thank you for pointing that out. The redundancy part in the discussion is removed

Powered by Editorial Manager® and ProduXion Manager® from Aries Systems Corporation

	02Specific Comments:<br>The abstract provides a good summary but lacks specific details about key findings. Including numerical results or specific genes/QTLs would enhance its informativeness.Thank you for bringing that issue to our attention. The main findings and significant numerical results have been clearly detailed in the abstract section.<br>03The introduction is comprehensive but overly descriptive in some parts. Focus more on the knowledge gap and the study's unique contributions.I am grateful for your bringing this matter to my attention. I have incorporated a paragraph that addresses the existing knowledge gaps and underscores the distinctiveness of the current study.<br>04Include more references to recent studies on selection signals and genomic studies in sheep from diverse geographical contexts.Thank you for your valuable input. Recent research on selection signals and global studies on sheep have been included in the references.<br>05Describe the data quality control steps in more detail. For example, how were outlier SNPs or missing data handled?Thank you for your comments about it. Detailed the steps for controlling data quality in the methods section.<br>06Clarify the composite selection signal (CSS) methodology. Provide justification for using this approach over others (e.g., iHS, XP-EHH).I'm thankful for your notice on this matter. The methodology of composite selection signals and its advantages compared to alternative methods were incorporated.<br>07Were any statistical corrections applied to account for population structure or relatedness among individuals?Thank you for your comment. Pi hat values are computed for all individuals in the study, and the genomic relatedness among them is low. As a result, all individuals are included in the selection signature analysis.<br>08The presentation of the results is clear, but:<br>Include figures or tables summarizing key candidate genes or QTLs identified.I appreciate you bringing that to my attention. The text included a table that summarizes the important candidate genes.<br>09Highlight specific loci and their putative roles in traits relevant to Indian sheep (e.g., drought tolerance, disease resistance).I'm grateful for your insight on this. The proposed genetic locations associated with traits such as drought tolerance and disease resistance were highlighted in the updated version.<br>10Consider including a heatmap or Manhattan plot for better visualisation of selection signals.I appreciate you pointing that out to me. The manuscript now includes a Manhattan plot that effectively illustrates the selection signals identified in our analysis.<br>11The discussion lacks depth in interpreting biological functions of identified genes. Thanks for shedding light on that. The specifics regarding the molecular functions of the proposed genes were included in the discussion section.<br>12Provide insights into how these genes/QTLs align with known traits in sheep.Thanks for making me aware of it. The specified details have been incorporated into the manuscript in accordance with the recommendations provided.<br>13Compare findings with results from other studies (e.g., on sheep from different regions or breeds).Thanks for the comment. Comparison of findings of this study with other studies were included in the revised manuscript.<br>14Discuss potential limitations of the study, such as sample size or marker density, and how they might affect the robustness of the conclusions.I'm grateful for your comment. The potential limitations of the study are added in the manuscript.<br>15Figures are clear but lack detailed legends. Add more context to explain what the reader should infer from each figure.Thank you for pointing out. However, the existing pie chart has the common legend, that explains the name of the QTL classes.<br>16Tables summarizing selection signals should include effect sizes and p-values where relevantThanks for the comment. Changes incorporated as per the comment.<br><br>Manuscript Number: PONE-D-24-51732<br>Title:Title: Composite Selection Signal Analysis: Uncovering Candidate Genes and Quantitative Trait Loci in Indian Sheep Breeds<br>Reviewer 02<br>Specific Comments<br>S.No.Reviewer's CommentsResponse<br>01Link Between Genomic Regions and Local Adaptation: While the discussion mentions the environmental adaptations of the breeds, it could further elaborate on how these adaptations might have influenced the specific genomic regions under selection. For example, more direct comparisons to how the genetic variations might correlate with the climate or geographical features of the areas where these breeds are

	found could enhance the reader's understanding of the adaptive significance of these genes.Thank you for the suggestion. Incorporated the text as suggested<br>02Consistency in Gene References: Some genes are mentioned repeatedly (e.g., CNTNAP5), but the context of how they were identified in different breeds could be more explicitly connected to avoid confusion. For instance, you might clarify how CNTNAP5's role in body growth is shared across breeds like CHA and GAR, yet each breed's unique genomic context could influence the gene's effect.I'm grateful for your suggestion and have integrated the text as suggested.<br>03Discussion on Statistical Methodology: The use of the DCMS method is discussed, but more detailed insight into why this method improves the resolution power of the genomic signals could help readers understand the methodological benefits. Briefly touching on the weaknesses of other statistical approaches and how the DCMS method overcomes them would strengthen the argumentThanks for the recommendation; I've added the text accordingly.<br>04Gene Functionality and Mechanisms: While candidate genes are identified, more information on their molecular mechanisms or the processes through which they impact the phenotypic traits (e.g., how MTSS1 influences bacterial resistance) could help provide a clearer understanding of their biological relevanceI am thankful for your suggestion and have made the changes to the text as proposed.<br><br>05Comparison with Other Studies: The discussion of prior research, such as the studies from Ahmad et al. (2021) and Zhang et al. (2016), provides useful context. However, it would be helpful to briefly explain how the findings in the current study either align or contrast with these studies, reinforcing the study's contribution to the fieldI appreciate your suggestion and have appended the changes to the text as proposed.<br><br>General Comments<br>01Rephrase as "False Discovery Rate (FDR) threshold of < 5%"Modified as suggested<br>02Genes could be presented by trait type e.g., morphology (putative genes with sub-trait), growth (putative genes with sub-trait), reproduction (putative genes with sub-trait), adaptation (putative genes with sub-trait), disease resistance (putative genes with sub-trait), etc.Changes made as per the correction- Genes rearranged into sub-traits.<br>03QTLs could be grouped by trait also.The QTLs were grouped according to the the trait - modified as per the recommendations<br>04Referencing [1] is just akwarding as the sentence approaches to introduce a concept and hence remove the referenceRemoved the reference as suggested<br>05Explain why the concept of selection signatures is important or how understanding them benefits evolutionary biology.Updated as advised<br>06The phrase "mutations" seems redundant, as mutations are already mentioned in the previous sentence, so restructure the present sentence.Restructure the sentence as recommended<br>07This is clear but could be more engaging. Consider adding a sentence or two on how SNP arrays have specifically impacted genomic studies, perhaps by providing concrete examples of successful applications.Enhanced as proposed<br>08Give slight details about XPEHHRefined based on your feedback<br>09How does DCMS method work or why it seems more robust and sensitive? Edited in line with your comments.<br>10This sentence seems to appear abruptly. A sentence or two linking the SNP arrays discussed previously to the OvineSNP50 Bead Chip would provide a smoother transition.Revised according to your recommendation<br>11Authors might add a bit more context about why the OvineSNP50 is specifically chosen for Indian sheep genome studies. For example, is it due to its size, variety of SNPs, or its validation in Indian sheep breeds? Updated as advised<br>12Authors might use terms like "cold arid regions of the Western Himalayas" or "semi-arid regions of the Deccan Plateau", etc. for clarityAdjusted as indicated<br>13Correct spelling (Odisha)Corrected as suggested<br>14This is a strong statement that highlights the gap in research. Authors could emphasize why this gap is significant, perhaps briefly mentioning how genomics could benefit sheep production in India.Amended as suggested<br>15 Rephrase for clarity, such as: "In this study, we applied the composite signals method (DCMS) to detect selection signatures in the genomes of three Indian sheep breeds (Chantangi, Deccani, and Garole), which are adapted to diverse ecological

	niches and production systems across India.Changed as per your suggestion.<br>16Briefly mention why these particular breeds are chosen. Are they important economically, or do they exhibit distinct phenotypic traits?Updated following your suggestion<br>17It will be better to represent in a single sentence highlighting the goat or objective of the study. Amended in accordance with your feedback<br>18Rephrase for better flow: "In the present study, we analyzed high-density genotypes from three Indian sheep breeds: Changthangi (CHA, n = 29), Deccani (IDC, n = 24), and Garole (GAR, n = 26), genotyped using the Illumina Ovine SNP50 BeadChip, which were obtained from WIDDE [14]."Refined as you advised<br>19Consider providing a bit more context about WIDDE (e.g., what it stands for or its relevance) for clarity.Enhanced based on your thoughts<br>20For clarity, authors might say: "The Illumina Ovine SNP50 BeadChip consists of 54,241 SNPs distributed across the sheep genome, with an average density of one SNP every 51 kb."Rephrased as recommended<br>21Improve for clarity and consistency: "Quality control (QC) of the genotypes was performed based on the following criteria: minor allele frequency (maf < 0.05), Hardy-Weinberg equilibrium (HWE p < 0.0001), individuals with missing genotypes (--mind < 0.1), and SNPs with missing genotype data (geno < 0.1)."Updated following your suggestion<br>22It would be helpful to clarify why these specific thresholds (maf, HWE, etc.) were chosen.<br>23Authors might briefly explain why X-chromosome SNPs were excluded, especially if it's relevant to the study design (e.g., if focusing only on autosomes).Explained following your feedback<br>24Authors could expand this purpose of PCA slightly on what they hoped to achieve with PCA (e.g., confirming population structure or identifying outliers).Expanded as suggested<br>25Mention briefly what the "snprelate" package does. For example, authors could say: "The PCA was performed using the 'snprelate' package in R, which is designed for efficient analysis of large-scale genotypic data."Refined as you advised.<br>26Authors might also consider adding a sentence explaining why DCMS is preferred in this context or how it enhances the analysis of selection signals.Enhanced as proposed<br>27The present study utilized the Composite of Multiple Signals (DCMS) method, as detailed in Yurchenko et al. (2018), combining five univariate statistics: FST, Haplotype Homozygosity (H1), Modified Haplotype Homozygosity (H12), Tajima's D, and Nucleotide Diversity (π) [18–21]."Corrected following your input<br>28Correct as 'locus'Edited as per your suggestion<br>29Consider adding a more intuitive explanation of why these components are combined (e.g., "The DCMS statistic combines both the p-values of individual statistics and their correlations to improve the sensitivity and robustness of detecting selection signals.").Refined based on your feedback<br>30briefly mention why FST is used (e.g., to quantify genetic differentiation).Updated following your suggestion<br>31explain a bit more about why these statistics are important and how they contribute to detecting selection signals (e.g., "Haplotype homozygosity measures the extent to which haplotypes are conserved, which can help identify regions under selective pressure.").Explained, following your advice<br>32consider elaborating on how they relate to detecting selection. For example, "Tajima's D detects departures from neutrality, with negative values indicating potential selective sweeps, while nucleotide diversity (π) measures the genetic variation within a population."Enhanced as proposed<br>33Authors might rephrase as "The MINOTAUR package was used to compute p-values for the five statistics based on their functional ranks, integrating multiple signals into a composite measure."Altered as per the comment<br>34 Consider adding a sentence or two that briefly explains the role of these steps in improving the accuracy of the results.Adapted per your suggestion<br>35 Modify the criteria for determining putative regions as "A q-value threshold of 0.05 was chosen to identify genomic regions with strong evidence of selection, while adjacent SNPs with q-values greater than 0.1 were excluded to reduce false positives.").Changed in response to your advice<br>36clarify how the QTL enrichment analysis is performed. Authors might write as "QTL enrichment analysis was performed to identify overrepresented traits within the

Powered by Editorial Manager® and ProduXion Manager® from Aries Systems Corporation

significant regions, using a genome-wide approach and a false discovery rate (FDR) threshold of 0.05.").Revised based on your input
37The terms are essentially redundant. Consider: "missingness thresholds for SNP genotypes (geno <0.1) and individual genotypes (mind <0.1)."Altered per your guidance
38Exact number or percentages of SNPs excluded for each criterion are missing. Included in accordance with your feedback
39Exact number or percentage of SNPs excluded for this criterion is missing. Providing these numbers or percentages of all criteria would add more transparency to the data quality process.Included in accordance with your feedback
40Modify as "The PCA confirmed the genetic differentiation among the three breeds, as indicated by their clear separation into distinct clusters.Corrected following your input
41 May be presented as "PC1 accounted for 14.34% of the variation, highlighting the strongest genetic differences between the populations, while PC2 explained an additional 7.98% of the total variation, capturing finer distinctions."Refined as you advised
42correct whether it is "Chantangi" or "Changthangi." Corrected as per your suggestion
43The size range for genomic regions under selection (from 1080.32 to 1.83 Kb) is inconsistent with the typical size of genomic regions under selection in animal breeding studies. Consider reviewing these figures (DCMS Metrics) for accuracy.We agreed to the statement you pointed out. However, the average length of genomic regions under selection was consistent with the literature, but not range. In our study, the range of genomic size we observed was not consistent with the literature.
44It would help to summarize the QTL classes before delving into breed-specific information by adding one sentence like "The QTLs identified in each breed were categorized into various trait classes, including body weight, reproduction, milk yield, and health."Added a sentence following your suggestion
45but the results could be more digestible if key QTLs from the tables were also summarized in the text. For example, "The most significant QTL associated with milk yield in CHA sheep was found on OAR 16, as shown in Table 3."Modified the sentence as per your feedback
46Recheck to ensure the consistency and biological relevance of the findings.
47Can be expanded to mention any statistical findings or significance levels, if availableThe significance levels were added in the sentence
48 Rephrase as "The genomic regions under selection ranged in size from 2.20 to 817.32 Kb, with an average length of 167.55 ± 216.86 Kb."Altered per your guidance.
49Could be expanded by specifying if any of these findings were novel or aligned with previous studies.
Expanded following your suggestions
50Could be expanded by specifying if any of these findings were novel or aligned with previous studies.Expanded following your suggestions
51Can be expanded to mention any statistical findings or significance levels, if available.The significance levels were added in the sentence
52Consider rephrasing as: "Few studies have used multiple complementary statistics to analyze selection signatures in these breeds, and many do not account for correlations between these statistics."Changed in response to your advice
53Elaborate on how these adaptations might have influenced the specific genomic regions under selection. For example, more direct comparisons to how the genetic variations might correlate with the climate or geographical features of the areas where these breeds are found could enhance the reader's understanding of the adaptive significance of these genes.Refined as per your advice
54 Explain how the findings in the current study either align or contrast with these studies.Specified the following, per your suggestions
55Crosscheck for completeness of citations. Checked the reference citations as per your suggestion
56Check for the journal style – Table headingChecked in response to your advice

S.No.Reviewers CommentsResponse to Reviewer’s Comments
Reviewer # 03
01The manuscript, especially the discussion part is exceedingly long with redundancy in a few parts which is repeated in the results and discussion sectionThank you for pointing out it. The redundancy part in the discussion is removed

Powered by Editorial Manager® and ProduXion Manager® from Aries Systems Corporation

	02Check the spelling "Chantangi" which is wrongly written Chanthangi is replaced with Chantangi<br>03Concise the discussion part and avoid redundancy. Thank you for highlighting it. The Discussion part is concised as suggested.
Additional Information:	
Question	Response
**Financial Disclosure**<br><br>Enter a financial disclosure statement that describes the sources of funding for the work included in this submission. Review the submission guidelines for detailed requirements. View published research articles from *PLOS ONE* for specific examples.<br><br>This statement is required for submission and **will appear in the published article** if the submission is accepted. Please make sure it is accurate.<br><br>**Funded studies**<br>Enter a statement with the following details:<br>• Initials of the authors who received each award<br>• Grant numbers awarded to each author<br>• The full name of each funder<br>• URL of each funder website<br>• Did the sponsors or funders play any role in the study design, data collection and analysis, decision to publish, or preparation of the manuscript?<br><br>Did you receive funding for this work?	The author(s) received no specific funding for this work.
**Competing Interests**<br><br>Use the instructions below to enter a competing interest statement for this submission. On behalf of all authors, disclose any competing interests that could be perceived to bias this work—acknowledging all financial support and any other relevant financial or non-financial competing interests.<br><br>This statement is required for submission	The authors have declared that no competing interests exist.

and **will appear in the published article** if the submission is accepted. Please make sure it is accurate and that any funding sources listed in your Funding Information later in the submission form are also declared in your Financial Disclosure statement.<br><br>View published research articles from *PLOS ONE* for specific examples.<br><br>**NO authors have competing interests**<br><br>Enter: *The authors have declared that no competing interests exist.*<br><br>**Authors with competing interests**<br><br>Enter competing interest details beginning with this statement:<br><br>*I have read the journal's policy and the authors of this manuscript have the following competing interests: [insert competing interests here]*<br><br>* typeset	
**Ethics Statement**<br><br>Enter an ethics statement for this submission. This statement is required if the study involved:<br><br>• Human participants<br>• Human specimens or tissue<br>• Vertebrate animals or cephalopods<br>• Vertebrate embryos or tissues<br>• Field research<br><br>Write "N/A" if the submission does not require an ethics statement.<br><br>General guidance is provided below. Consult the submission guidelines for detailed instructions. **Make sure that all information entered here is included in the Methods section of the manuscript.**	Sri Venkateswara Veterinary University, Tirupati, Andhra Pradesh

Powered by Editorial Manager® and ProduXion Manager® from Aries Systems Corporation

**Format for specific study types**<br><br>**Human Subject Research (involving human participants and/or tissue)**<br>• Give the name of the institutional review board or ethics committee that approved the study<br>• Include the approval number and/or a statement indicating approval of this research<br>• Indicate the form of consent obtained (written/oral) or the reason that consent was not obtained (e.g. the data were analyzed anonymously)<br><br>**Animal Research (involving vertebrate animals, embryos or tissues)**<br>• Provide the name of the Institutional Animal Care and Use Committee (IACUC) or other relevant ethics board that reviewed the study protocol, and indicate whether they approved this research or granted a formal waiver of ethical approval<br>• Include an approval number if one was obtained<br>• If the study involved *non-human primates*, add *additional details* about animal welfare and steps taken to ameliorate suffering<br>• If anesthesia, euthanasia, or any kind of animal sacrifice is part of the study, include briefly which substances and/or methods were applied<br><br>**Field Research**<br><br>Include the following details if this study involves the collection of plant, animal, or other materials from a natural setting:<br>• Field permit number<br>• Name of the institution or relevant body that granted permission	
**Data Availability**<br><br>Authors are required to make all data underlying the findings described fully available, without restriction, and from the time of publication. PLOS allows rare exceptions to address legal and ethical concerns. See the PLOS Data Policy and FAQ for detailed information.	Yes - all data are fully available without restriction

A Data Availability Statement describing where the data can be found is required at submission. Your answers to this question constitute the Data Availability Statement and **will be published in the article**, if accepted.<br><br>**Important:** Stating 'data available on request from the author' is not sufficient. If your data are only available upon request, select 'No' for the first question and explain your exceptional situation in the text box.<br><br>Do the authors confirm that all data underlying the findings described in their manuscript are fully available without restriction?	
**Describe where the data may be found in full sentences. If you are copying our sample text, replace any instances of XXX with the appropriate details.**<br><br>• If the data are **held or will be held in a public repository**, include URLs, accession numbers or DOIs. If this information will only be available after acceptance, indicate this by ticking the box below. For example: *All XXX files are available from the XXX database (accession number(s) XXX, XXX.).*<br>• If the data are all contained **within the manuscript and/or Supporting Information files**, enter the following: *All relevant data are within the manuscript and its Supporting Information files.*<br>• If neither of these applies but you are able to provide **details of access elsewhere**, with or without limitations, please do so. For example:<br><br>*Data cannot be shared publicly because of [XXX]. Data are available from the XXX Institutional Data Access / Ethics Committee (contact via XXX) for researchers who meet the criteria for access to confidential data.*<br><br>*The data underlying the results presented in the study are available from (include the name of the third party*	The genotypic data of indigenous Sheep breeds of India (Chantangi, Garole, and Deccani; n=29, 24, and 26, respectively) were obtained from the WIDDE database with the following link: http://widde.toulouse.inra.fr/widde/widde/main.do?module=sheep.

and contact information or URL).<br>• This text is appropriate if the data are owned by a third party and authors do not have permission to share the data.<br><br>* typeset	
Additional data availability information:	

Powered by Editorial Manager® and ProduXion Manager® from Aries Systems Corporation

Manuscript
Click here to access/download;Manuscript;Revised Manuscript with out Track Changes.doc

Composite Selection Signal Analysis: Uncovering Candidate Genes and Quantitative Trait Loci in Indian Sheep Breeds

Sapna Nath[1¶], Satish Kumar Illa[2¶], Destaw Worku Mengistu[3*], Sabyasachi Mukherjee[4], Anupama Mukherjee[4], Vinod Kumar Yata[5]

[1]College of Veterinary Science, Garividi, Sri Venkateswara Veterinary University, Andhra Pradesh, India.

[2]Livestock Research Station, Garividi, Sri Venkateswara Veterinary University, Andhra Pradesh, India.

[3]Department of Animal Science, Injibara University, Ethiopia

[4]Division of Animal Genetics and Breeding, Indian Council of Agricultural Research-National Dairy Research Institute, Karnal, Haryana, India.

[5]Department of Molecular Biology, Central University of Andhra Pradesh, Ananthapuramu, Andhra Pradesh, India

*Corresponding author

Email: destawworku@gmail.com

¶Authors contributed equally to the work

Abstract:

Natural and artificial selection are fundamental demographic forces that contribute significantly to the shaping of genomes. Exploring the molecular mechanisms implicated in the adaptation processes can assist in creating efficient breeding programs designed to preserve and improve the genetic characteristics of native sheep. The study used a novel method that combines the *p*-values into a single measure to investigate the genomic regions under selection. This method improves the resolution power and prioritises the candidate genes more efficiently than other single statistics. The 50K SNP data on three Indigenous sheep breeds (n=79) were Chantangi (CHA, n= 29), Garole (GAR, n=24), and Deccani (IDC, n=26) native to diverse regions of India and were used in this study. The study combined five statistics into a composite selection signal called a De-Correlated composite of multiple signals (DCMS). The *p*-values of all the single statistics were corrected for multiple testing at the False Discovery Rate (FDR) threshold of < 5%, and the putative genomic regions were detected. Gene and quantitative trait loci (QTL) annotation of the significant regions was performed. The DCMS analysis identified 21, 10, and 14 novel putative genes in the Chanthangi, Garole, and Deccani breeds, respectively. Furthermore, the study also revealed 10, 28, and 13 significant quantitative trait loci regions within the studied breeds. The identified genes and QTLs are related to a wide variety of characteristics, including growth and muscle development (*CNTNAP5, DOCK3*), reproduction (*TCERG1L, BUB1, UNC5C, C2CD5, BBX*), wool trait (*TPPP3, P2RY6, FGF10, POU2F1, FAM168A*), disease resistance

(*MTSS1, B4GALNT3*), environment adaptation (*TRMT12, MAPKAPK3*), domestication (*LRRC36*). The QTLs identified are associated with body conformation (body measurements and bone area), production (milk fat yield), reproduction (total lambs born), disease resistance (hemonchus resistance, foot rot and pneumonia susceptibility), and health (platelet count and entropion). The DCMS method pinpointed novel genomic regions and putative genes more efficiently than other single statistics. It could provide a reliable comprehension of the molecular mechanisms involved in all the essential traits in the Indian sheep.

Keywords: DCMS, Indian Sheep, SNP, QTL, Selection Signature

Introduction:

Natural selection enhances the fitness value of individuals and allows them to reproduce. Hence, the frequency of beneficial mutations increases in the population [1]. Thus, selection pressure on the genome leaves a distinct pattern, and those unique footprints are referred to as selection signatures [2,3]. It provides fundamental insights into the evolutionary forces that alter the genetic makeup of individuals, aids in QTL mapping of quantitative traits in domestic animals, and also reveals beneficial putative mutations with a selection advantage in livestock populations [4]. However, complicated evolutionary processes, including population admixture, inbreeding, population bottlenecks, genetic drift, and mutations, obscure the patterns of selection signatures.

SNP genotyping arrays have revolutionized the study of genomic variations in populations, and nowadays, they are commonly used in genomic studies. These arrays consist of genetically stable single nucleotide polymorphisms distributed across the genome. These SNP arrays provide a deeper understanding of evolution, domestication, breed diversity, the formulation of population theories, the analysis of genetic processes underlying complex traits, and the enhancement of traits through genomic selection. Several methods are described in the literature to identify the signatures of selection based on allele frequency spectrum, linkage disequilibrium, and population differentiation [5,6]. The selection signature analysis previously described has been utilised in various sheep breeds distributed worldwide [7–9]. However, Grossman et al., 2010 proposed a method that combines different signals into a composite of signals (CMS) to enhance the details of the genomic region. This method is believed to be more sensitive and robust than the single selection signal method. The integral haplotype score method yields more reliable signals when the genome undergoes incomplete or ongoing selection. Meanwhile, the XPEHH method efficiently identifies the signals that are almost fixed. It analyzes the genomes of populations to determine the alleles that are becoming fixed in one group but remain variable in others. Hence, most of the studies on farm animals used complementary methods to improve the resolution of signals [5,11].

However, Lotterhos et al., 2017; Ma et al., 2015, proposed an efficient method called a de-correlated composite of multiple signals (DCMS) that combines the results obtained from univariate analysis. The covariance structure among the output signals of

single methods is accounted for to obtain more robust and sensitive signals. DCMS provides accurate signals owing to its better resolution power. The composite signals compute P-values by ranking genomic data, while univariate statistics rely on traditional probability and significance frameworks. Therefore, the DCMS provides advantages over univariate methods due to their lack of standardization, and this method is preferred in the selection signature analysis. SNP arrays are widely used genotyping tools for determining selection signatures in livestock. The OvineSNP50 Bead Chip (Illumina Inc., San Diego, CA) is the most widely used SNP array for studying variations in the sheep genome. This array was developed by incorporating highly polymorphic SNPs from sheep breeds worldwide, with a few Indian sheep breeds also included. [14].

The sheep production systems in India are diverse and include cold arid regions of the Western Himalayas, hot arid regions of Rajasthan and neighbouring states, semi-arid regions of the Deccan Plateau and sub-humid regions of West Bengal and Odisha. According to the 19th Census in India, the sheep population is 74.44 million heads, including 44 registered breeds [15]. The Indian sheep genetic resources comprise diverse morphological, physiological, and behavioral attributes developed over centuries of adaptation to various ecological niches and production systems.

Genomics can aid in identifying variants associated with productivity, disease resistance, and adaptability to diverse environmental conditions, enabling more informed selection decisions. Genomic selection programs can optimise traits such as

growth rate, wool quality, and reproductive efficiency while also helping to preserve the unique characteristics of native sheep. Despite the rich phenotypic diversity observed in Indian sheep, comprehensive genomic studies aimed at uncovering the molecular basis of these traits have been relatively limited. However, few studies on selection signatures in Indian sheep using a single statistic or a combination of univariate statistics have been reported [16,17]. Eydivandi et al., 2021 studied the selection signature analysis in Indian sheep using a combined statistic accounting for the correlations. However, the study did not use the composite signal method to detect the signals within the breed. In this study, the composite signals method (DCMS) was utilized to identify selection signatures in the genomes of three Indian sheep breeds (Chanthangi, Deccani, and Garole), which are adapted to different ecological niches and production systems across India. Additionally, gene annotation and QTL enrichment were performed.

Materials & Methods

Animals and Genotyping

In the present study, the high dense genotypes of three Indian sheep breeds i. e., Changthangi (CHA, n = 29), Deccani (IDC, n = 24), and Garole (GAR, n = 26) genotyped with Illumina Ovine SNP50 BeadChip were obtained from WIDDE (Web-Interfaced next generation Database dedicated to genetic Diversity Exploration) [18]. It is specifically designed to store and manage high-density genotyping datasets pertaining

to bovine and ovine species. The Illumina Ovine SNP50 BeadChip consists of 54,241 SNPs distributed across the sheep genome, with an average density of one SNP every 51 kb. Quality control (QC) of the genotypes was performed based on the following criteria: minor allele frequency (maf < 0.05), Hardy-Weinberg equilibrium (HWE p < 0.0001), individuals with missing genotypes (--mind < 0.1), and SNPs with missing genotype data (geno < 0.1) [19]. These criteria were applied to eliminate low-quality SNPs and advance to subsequent phases of the analysis. A total of 41,997 SNPs were retained for further analysis. The SNPs with unknown coordinates and those on X-chromosomes were excluded, as the study aims to uncover only the genes on autosomes. The SNPs with precise coordinates based on OAR Ramboulliet reference genome assembly were included for analysis.

Ethics Statement:

The research does not involve human or animal participants, and the data examined are publicly available. Therefore, no ethical approval or consent is necessary.

Principal Component Analysis:

The principal component analysis (PCA) was performed on the SNP genotypes of the studied sheep breeds to investigate the structure and clustering of the individuals in the dataset. PCA categorizes individuals into several specific clusters, and an analysis of selection signatures will be conducted on those individuals within each cluster. The PCA analysis was implemented in the R environment using the *snprelate* package [20].

De-Correlated Composite of Multiple Selection Signals

DCMS is a novel combined statistic used to detect selection signals, as it provides more reliable and precise estimates than other univariate statistics. The present study utilized the Composite of Multiple Signals (DCMS) method, as detailed in Yurchenko et al. (2018), combining five univariate statistics: FST, Haplotype Homozygosity (H1), Modified Haplotype Homozygosity (H12), Tajima's D, and Nucleotide Diversity (π) [21–24].

The computation of the DCMS statistic for a locus l is given below

$$DCMS_l = \sum_{t=1}^{n} \frac{\log\left|\frac{1-P_{lt}}{P_{lt}}\right|}{\sum_{i=1}^{n}|r_{it}|} \qquad (1)$$

The DCMS statistic combines the p-values of individual statistics and their correlations to improve the sensitivity and robustness of detecting selection signals. The DCMS statistic is composed of two components. The numerator component includes P_{lt}, which indicates the p-value at a given locus 'l' of each univariate statistic 't'. The denominator contains the correlation parameter 'r_{it}', which accounts for the covariance structure among all the included univariate statistics. It is also described as weighing factors among the statistics that describe the correlations between the statistics [12].

Univariate statistics in DCMS

Fixation Index (F_{ST}):

The F_{ST} index denotes the degree of differentiation between the populations. The functions in *PLINKV1.9*, such as --fst and --within, were used to determine the FST values for each SNP, and the negative F_{ST} values were changed to zeros. Later, the F_{ST} values were smoothed using the *runmed* function in the R program. The F_{ST} values for each breed were estimated.

Haplotype Homozygosity Statistics (H1 and H12):

Haplotype homozygosity assesses the conservation of haplotypes, which can aid in identifying regions subject to selective pressure. The haplotypes in each data set were determined using the SHAPEIT2 version program [25]. The conditional states parameter was kept as the default for the phasing of the haplotypes. The SNePv1.1 program computed the effective population size of each breed, and the estimate for each breed was used in the phasing of the chromosomes [26]. A high-resolution Ovine recombination map was used to correct the variations due to the recombination rates at all the autosomes [27]. An R script was used to convert the phased haplotypes to the input files as required in the H12_H1H2.py program. The window size of SNPs was kept to 14, and the step size was 1 (-window 14 -jump 1) [24].

Tajima's D and Nucleotide Diversity (π)

Tajima's D detects departures from neutrality, with negative values indicating potential selective sweeps, while nucleotide diversity (π) measures the genetic variation within a population. The statistics Tajima's D and (π) at each coordinate in the dataset of all the studied breeds were computed using the vcftools program [28], and the estimates were

computed for each chromosome separately. The nonoverlapping sliding window of 300 MB (-Tajima 300) was used to compute the Tajima's D index. Further, the *p-values* were allotted to the SNPs present in the bin. Meanwhile, the –site-pi function in the vcftools program is employed to compute the π values of each SNP. The runmed function smooths the *p*-values as outlined in [29].

DCMS Estimation

The stat_to_p-value function in the MINOTAUR package was implemented based on the five statistics, depending on the functional ranks [30]. All five statistic parameters (H1, H12, Tajima's D index, π, and FST) for each SNP were combined into a new composite signal as DCMS. The covNAMcd function of the rrcovNA R package (alpha = 0.75, nsamp = 50,000) was utilised to compute a correlation matrix of n x n order [31]. The correlation matrix is useful for accounting for the correlations among the univariate statistics, which combine to produce a composite statistic. The output thus obtained was given as input for DCMS estimation, and the DCMS values were normalised using the rlm function of the MASS package in the R environment [32] as described in Yurchenko et al. (2018). The [33]A correction that reduces the error rate from multiple tests was applied to q-values transformed from p-values [34].

Identification of Functional Genes and QTL regions

The putative genomic regions were determined based on the q-value. A q-value threshold 0.05 was chosen to identify genomic regions with strong evidence of selection, while adjacent SNPs with q-values greater than 0.1 were excluded to reduce false

positives. The gene and QTL annotation of the candidate genomic regions was carried out using the Genomic Annotation in Livestock for positional candidate Loci (GALLO) package v4.2.0 [35]. The '.gtf' file related to ARS-UI_Ramb_v2.0 of the sheep genome was accessed from NCBI, and the corresponding '.gff' belonged to the ARS-UI_Ramb_v2.0 was downloaded from the Sheep QTLdatabase [36].

The candidate genes and quantitative trait loci (QTLs) located within the significant genomic regions are considered to be putative if they are distributed within an interval of 0.5 Mb upstream and downstream. Further, QTL enrichment analysis was performed to identify overrepresented traits within the significant regions, using a genome-wide approach and a false discovery rate (FDR) threshold of 0.05.

Results:

Quality Control of genotypic data:

The quality control of genotypic data included the filtering of SNPs with minor allele frequency (maf<0.05), Hardy Weinberg equilibrium of the SNPs (hwe<0.0001), missingness thresholds for SNP genotypes (geno <0.1) and individual genotypes (mind <0.1. The SNPs excluded for maf, hwe, geno and mind were 3845, 299, 678 and 0, respectively. Further, the SNPs with unknown coordinates (n=1232) on the chromosomes other than the autosomes were removed. The final data set contains 41997 variants used for further downstream analysis.

Principal Component Analysis (PCA)

The principal component analysis (PCA) was employed to ascertain the distinct clusters of SNP genotypes for the three Indian sheep breeds under investigation. The results of PCA are presented in Fig 1. The PCA confirmed the genetic differentiation among the three breeds, as indicated by their clear separation into distinct clusters. PC1 accounted for 14.34% of the variation, highlighting the strongest genetic differences between the populations, while PC2 explained an additional 7.98% of the total variation, capturing finer distinctions.

De-Correlated Composite of Multiple Selection Signals (DCMS)

Chanthangi sheep:

Gene Annotation:

The DCMS analysis revealed twenty-one putative regions in the Chanthangi sheep genome. The total size of the genome under selection was 2871.54 Kb. The mean genome length under selection was 136.74±229.77 Kb, ranging from 1.08 to 1.83 Kb (Table 1).

Table 1. shows the genomic regions of Indian sheep breeds identified as being under selection through DCMS analysis

S.No.	Breed	Total Size (kb)	Mean ± Standard Deviation (Kb)	Maximum Length (kb)	Minimum Length (kb)	Total No. of genes
01	Chanthangi (CHA)	2871.54	136.74±229.77	1080.32	1.83	21
02	Garole (GAR)	2857.84	259.80±310.04	1080.32	34.11	10
03	Deccani (IDC)	2513.30	167.55±216.86 Kb	817.32	2.20	14

These genes were located on BTA 2, 3, 9, 14, 15, and 26. A greater number of significant genomic regions were identified on BTA 15 (Table 2).

Table 2. shows the results of gene annotation in significant genomic regions in CHANTHANGI sheep

CHR*	BP*	START COORDINATE	END COORDINATE	Width	Strand	Gene Name	*Pvalue*	Qvalue
2	190267950	189526649	190606970	1080322	+	*CNTNAP5*	*4.95e-05*	*0.032*
3	106221882	106222211	106279403	57193	+	*FBLN7*	*1.29e-05*	*0.015*
3	107958569	107917450	107989336	71887	+	*TBC1D15*	*1.28e-05*	*0.015*
9	28518494	28426199	28586681	160483	+	*MTSS1*	*0.008*	*0.048*
9	28610511	28596736	28628449	31714	+	*TATDN1*	*9.16e-05*	*0.045*
9	28651053	28650081	28651912	1832	-	*TRMT12*	*0.000*	*0.048*
9	28651053	28627373	28650325	22953	-	*RNF139*	*0.000*	*0.048*
14	35090205	35080140	35173351	93212	+	*NFATC3*	*4.59e-06*	*0.009*
14	34566935	34510632	34569231	58600	-	*TPPP3*	*7.71e-05*	*0.042*
14	34566935	34504860	34579222	74363	+	*LRRC36*	*7.71e-05*	*0.042*
15	51070453	51028830	51075998	47169	-	*STARD10*	*2.34e-08*	*0.000*
15	51107183	51099187	51353916	254730	-	*FCHSD2*	*2.34e-08*	*0.000*
15	51474825	51404925	51511918	106994	+	*P2RY2*	*1.39e-08*	*0.000*
15	51517148	51481919	51516379	34461	+	*P2RY6*	*1.39e-08*	*0.000*
15	51578641	51521915	51579398	57484	+	*ARHGEF17*	*6.12e-10*	*2.57e-05*
15	51591704	51585751	51604071	18321	+	*RELT*	*3.07e-09*	*6.44e-05*
15	51632422	51613275	51814944	201670	-	*FAM168A*	*1.19e-05*	*0.015*
16	30667706	30604422	30702581	98160	+	*FGF10*	*7.11e-06*	*0.010*
16	31588015	31575745	31651791	76047	-	*NIM1K*	*4.12e-05*	*0.031*
16	31687647	31660770	31688311	27542	-	*ZNF131*	*1.85e-05*	*0.018*
16	32196927	32069778	32366181	296404	-	*GHR*	*9.62e-07*	*0.003*

*CHR – Chromosome; *BP – Base Position

The gene annotation of the significant genomic regions uncovered twenty-one potential candidate genes related to a variety of traits in this sheep, including *CNTNAP5* (body growth), *MTSS1* (host resistance to bacterial infections), *TRMT12* (immune system and environment adaptation), *TPPP3* (hair follicle growth), *LRRC36* (domestication and early development), *STARD10* (resilience to paratuberculosis), *P2RY6*, *FGF10* and

FAM168A (hair follicle morphogenesis), *ARHGEF17* and *NIM1K* (meat quality), *GHR* (litter size).

Quantitative Trait Loci (QTL) Annotation and Enrichment:

Table 3 presents the results of QTL annotation in Chanthangi sheep. Our study revealed the significant genomic regions annotated with ten QTLs on OAR 2, 3, 14, 15, and 16.

Table 3. shows the Quantitative Trait Loci annotation results in significant genomic regions in CHANTHANGI sheep.

CHR*	BP*	START COORDINATE	END COORDINATE	Name	Flank Marker	P-value
2	190267950	190457084	190457088	Body weight	rs424714738	1.61E-03
3	107991182	108401221	108401225	Body weight	rs426980328	0.026
14	35090205	35417680	35417684	Body weight	rs417231209	<0.05
15	51070453	50955799	50955803	Milk fat yield	rs404288918	0.0006245
15	51474825	51832265	51832269	Platelet count	rs413264572	5.50E-06
16	31588015	32070073	32070077	Total lambs born	rs161146164	<0.05
16	31654423	32118461	32118465	Total lambs born	rs426666828	<0.05
16	32569758	32119256	32119260	Milk fat yield	rs422431427	0.00031338
16	32569758	32192693	32192697	Body weight	rs408124092	<0.0001
16	32569758	32192693	32192697	Soft tissue depth at GR site	rs408124092	0.0014

*CHR – Chromosome; *BP – Base Position

The annotated traits are known to be associated with body weight, milk fat yield, platelet count, total lambs born, and soft tissue depth at the GR site. The QTLs identified in each breed were categorized into various trait classes, including body weight, reproduction, milk yield, and health, and the enrichment of QTLs is shown in Fig 2. The highest per cent of QTLs annotated in the significant regions of Chanthangi were milk (37.5%), followed by reproduction (34.38%). The other classes of QTLs annotated were meat and carcass (9.38%), health (9.38%) and production (9.38%). The

QTL enrichment analysis revealed the top significant (False Discovery Rate (FDR)-corrected p-value - 0.05) QTL total lambs born on OAR 16.

Garole sheep:

Gene Annotation:

The DCMS analysis found eleven (11 No's) potential regions in the Garole sheep genome covering the overall size of the genome, which was 2857.84 Kb. The average size of the genome under selection was found to be 259.80±310.04 Kb (Table 1). The maximum and minimum size of the genome ranged between 1080.32 and 34.11 Kb. Several putative genes were harboured in this study's significant genomic regions and were situated on OAR 1, 2, 3, 6, 16 and 18 (Table 4).

Table 4. shows the gene annotation results in significant genomic regions in GAROLE sheep.

CHR*	BP*	START COORDINATE	END COORDINATE	Width	Strand	Gene	*Pvalue*	*Qvalue*
1	173424721	173196985	173483458	286474	+	*BBX*	*2.01e-05*	*0.015*
2	189539157	189526649	190606970	1080322	+	*CNTNAP5*	*8.19e-05*	*0.042*
3	193287569	193251622	193339539	87918	+	*C2CD5*	*8.81e-07*	*0.003*
3	212967581	212887405	212973893	86489	+	*IQSEC3*	*6.47e-06*	*0.007*
3	213086338	213068112	213128627	60516	-	*KDM5A*	*1.07e-05*	*0.009*
3	213195386	213175874	213273093	97220	+	*B4GALNT3*	*7.64e-06*	*0.007*
6	29809690	29597358	30017600	420243	+	*UNC5C*	*6.21e-06*	*0.007*
16	65681259	65471620	65927572	455953	-	*ADCY2*	*7.56e-05*	*0.041*
18	46100361	46097255	46131370	34116	-	*CLEC14A*	*4.00e-05*	*0.024*
22	49602008	49599602	49793506	193905	-	*TCERG1L*	*6.19e-06*	*0.007*

*CHR – Chromosome; *BP – Base Position

However, OAR 3 anchors more potential candidate genes in the GAR breed. In this study, we identified eleven putative genes associated with a wide range of

characteristics, including *BBX* (teat number), *CNTNAP5* (body growth), *C2CD5* (fecundity), *KDM5A* (follicular development), *B4GALNT3* (paratuberculosis resistance), *UNC5C* (litter size), *ADCY2* and *TCERG1L* (reproduction).

Quantitative Trait Loci (QTL) Annotation and Enrichment:

The present study unveils the findings of QTL annotation in the GAR breed, shown in Table 5.

Table 5. shows the Quantitative Trait Loci annotation results in significant genomic regions in GAROLE sheep.

CHR*	BP*	START COORDINATE	END COORDINATE	Name	Flank Marker	P-value
1	173424721	173432597	173432601	Teat number	rs430157497	<0.05
1	173424721	173534798	173534802	Teat number	rs426598066	<0.05
2	190019411	190457084	190457088	Body weight	rs424714738	1.61E-3
3	192453314	192442586	192442590	Entropion	rs411640492	3.19E-04
3	192935483	192442586	192442590	Entropion	rs411640492	3.19E-04
3	193336800	193016493	193016497	Entropion	rs404605277	1.12E-04
3	212967581	213021781	213021785	Entropion	rs159913124	3.61E-04
3	213209196	213213768	213213772	Entropion	rs418752307	2.81E-04
6	29809690	29344134	29344138	Bone area	rs418089550	1.12E-5
6	29809690	29366036	29366040	Milk fat yield	rs424558688	0.036982472
6	29847992	30096782	30096786	Total lambs born	rs421635584	<0.05
18	46159259	46086980	46086984	Body weight	rs426187704	<0.05
18	46100361	46086980	46086984	Body depth	rs426187704	<0.01
18	46062053	46086980	46086984	Body weight	rs426187704	<0.05
18	46062053	46086980	46086984	Body length	rs426187704	<0.05
18	46159259	46086980	46086984	Body circumference	rs426187704	<0.05
18	46159259	46086980	46086984	Body depth	rs426187704	<0.01
18	46159259	46086980	46086984	Rump width	rs426187704	<0.05
18	46159259	46087004	46087008	Body weight	rs404696179	<0.05
18	46159259	46087004	46087008	Body length	rs404696179	<0.01
18	46100361	46087004	46087008	Body height	rs404696179	<0.01
18	46159259	46087004	46087008	Body circumference	rs404696179	<0.05

18	46062053	46087004	46087008	Body height	rs404696179	<0.05
18	46159259	46087646	46087650	Chest width	rs405457403	<0.05
18	46159259	46087646	46087650	Shin circumference	rs405457403	<0.05
18	46159259	46087646	46087650	Body depth	rs405457403	<0.05
18	47232764	47716852	47716856	Haemonchus contortus resistance	rs428856771	3.23E-4
22	49796078	49873766	49873770	Lambs born alive	rs402605786	9.79E-3

*CHR – Chromosome; *BP – Base Position

Our study revealed the significant genomic regions annotated with twenty-eight QTLs on OAR 1, 2, 3, 6, 18 and 22. The annotated traits are known to be associated with teat number, body weight, milk fat yield, entropion, bone area, total lambs born, body depth, body length, body circumference, shin circumference, body weight, body height, chest width, rump width, lambs born alive and hemonchus contortus resistance. Furthermore, the annotated QTLs are categorised into different classes, and the enrichment of QTLs is depicted in Fig 2A. Garole's most significant genomic regions had the greater proportion of QTLs associated with health (38.74%) and production (38.74%). The remaining per cent of QTL classes annotated in the breed were reproduction (11.71%), exterior (8.11%), milk (1.8%) and meat and carcass (0.9%). An analysis of QTL enrichment disclosed the three most remarkable QTLs (False Discovery Rate (FDR)-corrected p-value - 0.05) associated with the total lambs born, teat number and entropion on OAR 6, 1 and 3.

Deccani sheep:

Gene Annotation:

The analysis carried out by the DCMS has uncovered fourteen putative regions in the Deccani sheep genome. The genomic regions under selection ranged in size from 2.20 to 817.32 Kb, with an average length of 167.55 ± 216.86 Kb (Table 1). The findings of gene annotation are summarised in Table 6.

Table 6. shows the gene annotation results in significant genomic regions in DECCANI sheep.

S.No	CHR*	BP*	START COORDINATE	END COORDINATE	width	strand	Gene	*Pvalue*	Qvalue
1	1	119053600	119027142	119081828	54687	+	*MAEL*	9.08e-09	4.24e-05
2	1	131092601	130838877	131151750	312874	+	*APP*	3.55e-07	0.000
3	1	119491972	119428361	119624526	196166	+	*POU2F1*	1.33e-05	0.007
4	3	104706333	104671436	104716760	45325	-	*MALL*	9.21e-13	1.29e-08
5	3	104775041	104716342	104784266	67925	-	*NPHP1*	9.21e-13	1.29e-08
6	3	104885280	104870169	104915494	45326	-	*BUB1*	1.00e-13	4.20e-09
7	3	212967581	212887405	212973893	86489	+	*IQSEC3*	0.000	0.044
8	3	104917562	104914948	105286674	371727	+	*ACOXL*	1.64e-11	1.15e-07
9	4	101967194	101379345	102196659	817315	+	*CHRM2*	1.64e-11	1.15e-07
10	19	49657211	49447036	49738575	291540	-	*DOCK3*	6.78e-06	0.005
11	19	49778830	49759229	49789524	30296	-	*MAPKAPK3*	5.45e-06	0.004
12	2	52731947	52731991	52734550	2560	+	*HINT2*	5.26e-07	0.001
13	2	52790093	52790313	52801134	10822	-	*RGP1*	5.26e-07 0.001	0.001
14	2	52790093	52790904	52793100	2197	+	*MSMP*	5.26e-07 0.001	0.001

*CHR – Chromosome; *BP – Base Position

The gene annotation of the significant genomic regions revealed fourteen potential candidate genes related to a variety of traits in this sheep, including *BUB1* (oocyte and follicle development), *POU2F1* (wool colour), *CHRM2* (hair follicle growth), *DOCK3* (muscle development), *MAPKAPK3* (heat stress response). These genomic regions dock potential candidate genes associated with various traits and are located on OAR 1, 2, 3, 4 and 19, with a maximum number of significant genomic regions on BTA 19.

Quantitative Trait Loci (QTL) Annotation and Enrichment:

The findings outlined in Table 7 detail the results of QTL annotation in Deccani sheep.

Table 7. shows the Quantitative Trait Loci annotation results in significant genomic regions in DECCANI sheep.

CHR*	BP*	start_pos	end_pos	Name	FlankMarker	P-value
1	131092601	130616460	130616464	somatic cell count	rs409795528	<0.05
3	212967581	213021781	213021785	Entropion	rs159913124	3.61E-04
3	212967581	213213768	213213772	Entropion	rs418752307	2.81E-04
4	101967194	101960285	101960289	Monocyte number	rs399619443	2.55E-6
4	101967194	102026928	102026932	Footrot susceptibility	rs416121047	2.84E-6
4	101967194	102039511	102039515	Pneumonia susceptibility	rs403394816	<0.10
21	41665172	41243033	41243037	Body length	rs406947061	<0.05
2	52731947	52720586	52720590	Body weight	rs426272889	<0.05
2	52790093	52737532	52737536	Body weight	rs160159557	<0.05
2	52790093	52848103	52848107	Water holding capacity	rs420647640	<0.05
2	116594826	116133033	116133037	Horn type	rs416045857	<0.05
2	116594826	116839657	116839661	Horn type	rs405044765	<0.05
2	116953479	116872298	116872302	Horn type	rs402400571	<0.05

*CHR – Chromosome; *BP – Base Position

The study has identified significant genomic regions annotated with thirteen QTLs on OAR 1, 2, 3, 4 and 21. The QTLs are linked to the traits viz., somatic cell count,

Entropion, Monocyte number, Footrot susceptibility, Pneumonia susceptibility, Body length, Body weight, Water holding capacity, and horn type. Furthermore, the annotated QTLs are sorted into various categories, and the distribution of QTLs is illustrated in Fig 2. The regions with the most significant impact on the evolvement of the Deccani breed had the highest percentage of QTLs annotated for exterior (48.15%), followed by health (25.93%). The other classes of QTLs annotated were production (18.52%), and meat and carcass (7.41%). The QTL enrichment analysis unveiled the top most significant QTLs (False Discovery Rate (FDR)-corrected p-value - 0.05) on OAR 1,2,3 controlling traits like somatic cell count, horn type, Entropion, Body weight, and Body length (Fig 3). Further, the signatures of selection in the genome of the studied breeds are depicted in Fig 4.

In addition, the summary of putative genes is presented in the Table 8.

Table 8. Summary of putative genes identified in Indian sheep

CHR*	BP*	START COORDINATE	END COORDINATE	Gene	Trait
2	189539157	189526649	190606970	*CNTNAP5*	Growth and muscle development
3	193287569	193251622	193339539	*C2CD5*	Reproduction
9	28518494	28426199	28586681	*MTSS1*	Disease resistance
3	213195386	213175874	213273093	*B4GALNT3*	Disease resistance
9	28651053	28650081	28651912	*TRMT12*	Adaptation
3	104885280	104870169	104915494	*BUB1*	Reproduction
6	29809690	29597358	30017600	*UNC5C*	Reproduction
14	34566935	34504860	34579222	*LRRC36*	Domestication
19	49657211	49447036	49738575	*DOCK3*	Growth and muscle development
19	49778830	49759229	49789524	*MAPKAPK3*	Adaptation
15	51517148	51481919	51516379	*P2RY6*	Wool trait
16	30667706	30604422	30702581	*FGF10*	Wool trait

Discussion:

This study unveiled comprehensive information about the genomic regions under selection in three Indian sheep breeds adapted to the country's highly harsh climatic conditions. The DCMS analysis uncovered the potential candidate genes linked to various traits of economic importance in sheep. The complex and obscured evolutionary histories of livestock species indicate that a single univariate statistic may not adequately capture the full selection signals present in their genomes. The outcome of the univariate genome scan methods is mostly biased as they are greatly affected by the outliers, which determine the selection signals. Further, the application of background selection to neutral loci linked to recessive alleles could reveal biases in demographic inference. If a species exhibits widespread linkage disequilibrium, the impact of non-independence among loci on statistical outcomes remains uncertain, particularly when selection is present [13]. Keeping this in view, the present investigation used a novel statistic that combines *p*-values obtained from five different univariate statistics into a composite signal that accounts for the correlation among all univariate statistics. Few studies have used multiple complementary statistics to analyze selection signatures in these breeds, and many do not account for correlations between these statistics [17,37]. However, a recent study explored the genomic regions of Indian sheep using four complementary statistics accounting for their absolute correlations between the statistics [38]. This study mainly compared selection signatures in Iranian breeds with a comparison note on Indian and Afghani breeds. This study combined five univariate statistics, such as H1, H12, F_{ST}, TajimaD and Nucleotide

diversity, into a composite signal that accounts for correlations between them. It is well documented in the literature that the DCMS method provides absolute and accurate information compared to the results obtained from univariate statistics [10,12,13]. Hence, this study used composite signals to exhaustively study the signatures of the within-breed selections in Indian sheep breeds. As a result, the selection signatures analysis unveiled the potential candidate genes and quantitative traits in the genomes of the studied breeds.

Principal Component Analysis (PCA)

Principal Component Analysis (PCA) is a fundamental underpinning for exploratory data analysis. In short, the PCA computes the eigenvectors from the covariance matrix of the markers genotyped in the samples. Subsequently, the genotypic coefficients of the principal components will be visualized through plotting to illustrate the clustering resulting from population stratification. Thus, the dimensionality of the huge genotype matrix is reduced to principal components. The distinctive clustering of breeds into three categories, as depicted in the PC plot presented here, was not entirely consistent with the findings of Ahmad et al., 2021. The researchers could not differentiate three sheep breeds into separate clusters based on the first principal component. This could be due to the inclusion of a more significant number of other breeds, representing an additional variation source. Regional climatic conditions and environmental factors predominantly influence distribution patterns of livestock. These factors encompass temperature ranges, precipitation patterns, topography, and vegetation types, all of

which play critical roles in shaping the sustainable demographics of livestock. The study's findings indicated that the breeds evolved into unique and more specialised breeds owing to the prevailing local climate conditions. This research identified several candidate genes and genomic regions linked to local adaptation in sheep, highlighting essential QTLs associated with domestication, environmental adaptation, heat and cold tolerance, and resistance to bacterial diseases. In summary, the prevailing climatic and ecological conditions have facilitated the adaptation of the examined sheep breeds, each exhibiting specialized traits suited to their environments. The Garole breed, known for its high fecundity, thrives in the hot and humid conditions of West Bengal. In contrast, the Chanthangi breed, characterized by its wool quality, is predominantly found in the colder regions of the Himalayas. Similarly, the Deccani breed has adapted effectively to the arid environments of central India, showcasing its resilience to dry conditions (Nbagr.res.in).

Chanthangi sheep:

Gene Annotation:

The sheep production system in India spans a cold climate in the foothills of the Northern Himalayas to a hot, arid climate in Central Peninsular India and hot and humid regions of Eastern India. Based on our findings, none of the identified genes within the detected regions demonstrated significance across all three analysed breeds. However, the potential genomic regions docked the genes linked to body growth, reproduction, fecundity, heat stress response, host resistance to bacterial infections,

domestication, environmental adaptation, hair follicle growth, meat quality, and litter size. The study revealed minimal overlap of the genomic regions among the breeds, suggesting breed-specific selection processes in the past.

~~The significant genomic regions of the Chanthangi breed consist of the genes *CNTNAP5* (body growth), *MTSS1* (host resistance to bacterial infections), *TRMT12* (immune system and environment adaptation), *TPPP3* (reproduction), *LRRC36* (domestication and early development), *STARD10* (resilience to paratuberculosis), *P2RY6* and *FGF10* (hair follicle morphogenesis), *ARHGEF17* and *NIM1K* (meat quality), and *GHR* (litter size).~~

The present study found the *CNTNAP5* (Contactin-associated protein-like 5B) in the significant genomic regions harboured on OAR2, a protein-coding gene. This gene belongs to the neurexin family and plays a significant role as a cell adhesion molecule and receptor in the nervous system of most vertebrates. The mechanism of action of *CNTNAP5* is comparable with the brain-derived neurotrophic factor (*BDNF*) gene, which affects the body mass index. Like other epidermal growth factors, it potentially affects growth by enhancing the functions of the brain that have a role in growth regulation [39,40]. The genomic investigation of the morphological traits of Sudanese goats revealed that the *CNTNAP5* gene located on chromosome 2 is associated with the bicoastal diameter [41].

The indigenous sheep of India are known for their resistance to infectious agents. Our findings also identified specific genomic regions in Chanthangi sheep that harbour the *MTSS1* gene on chromosome OAR9 related to resistance to bacterial infections. These

findings are consistent with the breed characteristics of Chanthangi. The bacteria's endotoxins disrupt the endothelial lining in the host cell and cause infection. However, the *MTSS1* gene alters the host endothelium and improves its integrity to avoid infections [42]. The DCMS analysis also revealed significant genomic regions that anchor the *STARD10* gene, reportedly involved in the resistance to paratuberculosis [43]. It is involved in regulating bile acid metabolism by modulating the PPARα-mediated pathway [44]. The study found two essential candidate genes, *TRMT12* and *LRRC36*, associated with environmental adaptation and domestication in sheep. The study on the structural variant landscape in sheep and goats revealed that the *LRRC36* gene was involved in the early domestication of sheep and goats [45]. The *TRMT12* gene facilitates the codon-interactions while de-coding at the ribosomes, whereas, *LRRC36* is a leucine rich repeat containing 36 gene differentially expressed in testis and lung tissues.

Chanthangi sheep are reared mainly by Changpa, a nomadic tribe of the Northern hills, along with the Chanthangi goats. This dual-purpose sheep breed, maintained for wool and mutton, contributes significantly to the livelihood of the nomadic tribes. The income of the shepherds mainly was deployed from selling wool and meat. Interestingly, our study highlighted the critical candidate genes associated with hair follicle development, such as *P2RY6* (OAR 15) and *FGF10* (OAR 16). A gene expression study in mice uncovered the role of *P2RY6* in controlling the MEK1/2–b-catenin signalling pathways and MST–LATS1–YAP signal cascades. It indicated the gene function in the growth and development of keratinocytes [46]. Moreover, gene

expression investigation on the hair follicles of coarse sheep fetal skin revealed that the *FGF10* gene is involved in the hair follicle development by the process of competitive adsorption of miR-184 that produces *FGF10* from chi-circRNA-0001141 [47].

We also identified genes under selection in Chanthangi sheep, including *ARHGEF17* and *NIM1K,* which are known to be related to meat quality. Weighted single-step genome-wide association study (GWAS) findings have revealed a significant association between the *ARHGEF17* gene and meat traits within the Chinese yellow-feathered chicken population [48]. Further, Whereas, *ARHGEF17* is a key component of actin cytoskeleton organization and it enhances the guanyl-nucleotide exchange factor activity. Transcriptomic and epigenomic studies in the pectoral muscles in chicken embryos that belong to pure lines revealed the significant role of the *NIM1K* gene in muscle fibre formation, *NIM1K* gene Enables ATP binding activity; magnesium ion binding activity; and protein serine/threonine kinase activity and involved in protein phosphorylation [49].

Quantitative Trait Loci (QTL) Annotation and Enrichment:

The DCMS analysis revealed important QTL regions associated with body weight on OAR 2, 3, 14 and 16. The flanking markers or SNPs in the QTL regions, including rs424714738, rs426980328, rs417231209 and rs408124092, respectively. Interestingly, A genome-wide association study in Australian Merino sheep identified a significant QTL region on OAR2 associated with body weight. The study also found an orthologous region on OAR6, similar to human and bovine genomic regions associated

with height and weight [50]. Our study also found the same QTL as significant regions in the studied breeds. In a study, Zhang et al., 2016 identified the *MEF2B* and *TRHDE* gene polymorphisms linked to body weight at four months of age in New Ujumqin Sheep Populations located on OAR 3 [51]. The DCMS analysis revealed the same QTL region under selection in the Indian sheep populations. In Baluchi sheep, the genome-wide association study confirmed the location of the QTL peak associated with 8-month body weight [52]. Interestingly, we found the QTL on OAR 14 in the Chanthangi sheep. Likewise, Armstrong et al., (2018) investigated the genetic polymorphisms in the *GHR* gene and revealed their association with carcass traits in grazing Texel sheep. Interestingly, the same QTL peak was observed in the Chanthangi sheep.

We also identified QTL related to platelet count and milk yield on OAR15 in Chanthangi sheep. Interestingly, Gonzalez et al., (2013) reported the QTL association with mean corpuscular haemoglobin concentration (MCHC, P=6.2×10−14) and decreased mean corpuscular volume in Columbia, Polypay, and Ramboulliet sheep. The DCMS analyses also identified the QTL related to the platelet count in the significant genomic regions of Chanthangi sheep. Using three statistical models, the genomic investigation in the crossbred dairy sheep population revealed the QTL regions with the milk yield [55]. We also found the QTL region in the Chanthangi sheep population. We also found the same QTLs annotated in the significant genomic regions of Chanthangi.

Garole sheep:

The Garole sheep is renowned for its petite stature, remarkable fertility, better meat quality and ability to thrive in the saline marshlands of the hot and humid Sundarban region in West Bengal. This breed is also distributed across the southern regions of Bangladesh. Its small size is a result of its adaptation to the harsh climate. Although the genetic potential is relatively limited, these sheep could be raised within a management system requiring minimal inputs (Pan & Sahoo, 2008). This particular breed is highly esteemed due to its fecundity-related genes.

Gene Annotation:

The gene annotation results revealed a few candidate genes associated with essential traits in Garole sheep. We identified the *BBX* gene on OAR 1, which is involved in the supernumerary teats in sheep. Interestingly, a genome-wide association study in Wadi sheep of China reported the candidate genes linked to the supernumerary nipple phenotype in sheep [56]. This study unveiled a few candidate genes related to the trait, including the *BBX* gene. The *BBX* gene is involved in processes such as cell proliferation and breast tumors. It produces a high mobility group domain (HMG) transcription factor that can be seen in breast tumors [57]. The DCMS analysis revealed *the CNTNAP5* gene on OAR 2, which regulates the body growth in sheep, and this significant region was also annotated in the Chanthangi breed [41]. The same genomic region is similarly subject to selection in the Chanthangi breed, suggesting that the variants within this gene are undergoing selective pressure and have demonstrated influence on body growth in both breeds.

In this region, we identified a few candidate genes on OAR 3 in GAR sheep, including the *C2CD5, IQSEC3, KDM5A*, and *B4GALNT3* genes. An expression study in the sheep on oviductal mRNAs and lncRNAs in the follicular and luteal phase uncovered the C2CD5 gene [58]. Our study also identified the C2CD5 gene, which is involved in calcium-dependent phospholipid binding activity and regulates the insulin receptor signalling pathway. This is one of the interesting genes under selection linked with prolificacy and increased litter size in sheep, which is aligned with the demographic history of this sheep breed. Another gene, *KDM5A,* plays a significant role in the zygote genome activation in the cloned embryos in goats [59]. The DCMS analysis also pinpointed the *KDM5A* gene in our study of GAR sheep. We identified another promising candidate gene, *UNC5C,* under selection in GAR sheep. A genome-wide study in Hu sheep confirmed the role of the *UNC5C* gene in litter size in Hu sheep along with the *BMPR1B* gene [60]. According to Sugimoto et al., the *UNC5C* is a protein-coding gene that is a part of the UNC-5 family of netrin receptors and is associated with the conception rate in cattle [61]. Several whole genome studies confirmed the gene role in litter size in Hu sheep [62–64].

Another gene of interest in this study is the *ADCY2* gene, known for its pleiotropic effects. It produces cAMP, a signalling molecule in response to G protein signal transduction. This ultimately leads to increased IL-6 formation, a significant change in Brucellosis [65,66]. This gene also plays a significant role in reproduction, as it enhances the expression of estradiol [67]. Interestingly, we identified this gene in our study.

Quantitative Trait Loci (QTL) Annotation and Enrichment:

The DCMS analysis identified several QTL regions associated with teat number, body weight, body measurements, entropion, resistance to the Hemonchus infection, total lambs born, and total lambs alive.

Our study identified the peak QTL region at 201.6 (cM); the markers were rs430157497 and rs426598066. This genomic region on OAR1 surrounding the functional candidate genes, viz., BBX and CD47, was associated with supernumerary teats in the Wadi sheep of China [56]. One of the significant genomic regions on OAR6 is associated with bone area in Garole sheep. Previous research has identified a genomic region containing putative genes influencing bone density and area in Scottish Blackface lambs through a Genome-Wide Association Study (GWAS) [68].

The Garole sheep is known for its prolificacy and is much valued among breeders owing to its litter size. A few studies confirmed the genomic regions on OAR 6 and 22 that were potentially related to litter size, especially in the Wadi, Hu, Icelandic, Finnsheep, Romanov, and Lori-Bakhtiari sheep breeds [69,70]. The genes in the above potential region, including *BMPR1B*, *CTNNB1*, and *LHCGR*, are known to significantly affect litter size in some sheep breeds. Remarkably, the QTL associated with the litter size is consistent with the breeding history of the Garole breed.

The study on Somatostatin Receptor Subtype 1 (SSTR1) Gene Polymorphism and its relation with growth traits in the Hulun Buir Sheep and confirmed their significant association polymorphism [71]. Interestingly, we observed more QTLs on OAR18

associated with various body measurements such as body weight, length, circumference, rump width, body depth, chest width and shin circumference.

Deccani sheep:

Gene Annotation:

The Deccani breed represents the sole pure black coarse wool breed indigenous to India**.** Our study observed a significant genomic region harbouring a few candidate genes, such as MAEL, APP, and *POU2F1*. The *POU2F1* gene controls melanin production by binding with SLC7A11 and preventing its function [72]. This gene also affects the role of other candidate genes, including SLC7A11, MITF, SLC24A5, MC1R, and ASIP, and thus impacts melanin production and wool colour [73]. The results support the unique traits of the Deccani sheep breed.

Another remarkable region on OAR3 identified in this study consists of several significant genes viz., *MALL, NPHP1, BUB1, IQSEC3* and *ACOXL*. The *MALL* and *NPHP1* genes operate cell division and facilitate cell-matrix adhesion signalling. The *BUB1* gene encodes a protein that plays a role in DNA damage response. *IQSEC3* gene controls the small GTPase mediated signal transduction and *ACOXL* is associated with fatty acid beta-oxidation using acyl-CoA oxidase. Previous genomic studies on these breeds also confirmed the role of this region in conferring the immune response to several factors [37]. Surprisingly, our study also identified the genomic region.

We detected another signature genomic region on OAR2, which comprises the genes controlling sheep growth and fat deposition, including *HINT2, RGP1*, and *MSMP*. GWAS in Indigenous sheep of Ethiopia revealed a strong association of these genes with fat deposition [74] and growth traits. The HINT2 gene is involved in growth and fat deposition by facilitating the hydrolysis of various bonds, such as C-O, C-N, and C-C. Meanwhile, the RGP1 gene promotes the retrograde movement of membrane-bound vesicles to the trans-Golgi network, and the MSMP gene secretes a member of the beta-microseminoprotein family. A few other studies have reported the association of these genes with fat deposition and growth in sheep. [75,76].

In the present study, we identified another significant genomic portion of OAR14 associated with muscle development in cattle. *DOCK3* is a functional candidate gene in the genomic signature region. The molecular study in Bashbay sheep pinpointed the significant role of the *DOCK3* gene in the significant development of skeletal muscle in the specific breed (Zhang et al., 2021). The DOCK gene family is an 11-member class of guanine nucleotide exchange factors that play a crucial role in myoblast fusion and migration across various tissues. Additionally, these proteins are prominently expressed within the skeletal muscle system by stimulating the RAC1 and WAVE signalling pathways in skeletal muscle [77].

Quantitative Trait Loci (QTL) Annotation and Enrichment:

The QTL analysis uncovered a few putative signature genomic regions under selection, including those QTLs controlling traits such as somatic cell count, entropion, foot rot

and pneumonia susceptibility, monocyte number, body weight, body length, water holding capacity and horn length.

The DCMS analyses identified a significant putative genomic region associated with somatic cell count in this sheep. In a GWAS study, significant associations between SNPs and milk production, composition, coagulation, and cheese traits in Assaf and Churra dairy sheep were reported in the literature; somatic cell count is one of the associated traits in the study [78].

Monocytes are a type of white blood cell formed in the bone marrow. They play a vital role in the body's immune response. Differentiated monocytes play a role in phagocytosis and antigen presentation, contributing to adaptive immunity. In sheep, monocytes and macrophages act synergistically to counteract the septicemic conditions of Coxiella burnetii and lentivirus [79]. The genome-wide association study in Ramboulliet, Polypay and Columbia sheep identified the novel loci associated with the monocyte count on OAR 1, 2, 3, 4, 9, 10, 15, and 16. The genomic analysis revealed the loci associated with the monocyte count in the Deccani sheep genome on OAR4.

The DCMS analyses identified two other significant loci on chromosome 4 associated with foot rot and pneumonia susceptibility in sheep. Interestingly, the GWAS study in Katahdin, Blackbelly, and European-influenced crossbred sheep determined the strong associations of foot rot susceptibility in hairy sheep [80]. Their study uncovered a few loci on chromosome 4 related to foot rot susceptibility.

The genomic analyses revealed a significant association between body length and body weight on chromosomes 21 and 2. Interestingly, a genome-wide association

investigation on Qira Black and German Merino sheep indicated the association between the QTL region with the body measurements and body weight traits [81].

The present study identified two solid genomic associations on chromosome 2 in Deccani sheep related to water-holding capacity (a meat quality trait) and horn type. Deccani sheep are valued among consumers for their better meat quality traits. In addition, the sheep's physical characteristics, especially horns, are unique to this breed. Interestingly, the GWAS on the Colombian Creole hair sheep showed strong associations between the genomic regions on chromosome 2 and the water-holding capacity in meat [82]. Another genomic study demonstrated the genomic basis for the horn polymorphism in wild Soay sheep. The study reported a strong association of the genomic portions on chromosome 2 [83].

The findings of this study closely align with previous genomic research on Chantangi sheep [14], reinforcing the results concerning significant genomic regions and their associations with various traits. Similar to earlier studies [13,14,48], our investigation highlights key candidate genes that influence crucial characteristics such as growth, wool production, and disease resistance. These findings not only validate the genomic insights previously established for Indian sheep breeds but also contribute to a deeper understanding of the genetic foundations of these traits across different sheep populations. The consistency across studies emphasises the importance of these genomic regions in sheep breeding programmes aimed at enhancing desirable traits.

Conclusion:

We identified 51 putative QTL regions encompassing 45 protein-coding genes in Indian sheep breeds under selection and adapted to unique and distinct local climates. However, previous studies on the selection signatures of these breeds have been reported using few single statistics. Our study used a novel composite selection signature method (The de-correlated composite selection signal method) combining *p*-values into a single measure. This method improves the resolution power of the genomic signals and prioritises the functional candidate genes more effectively than single statistics. The putative QTLs and functional candidate genes revealed in the study were based on the historical background of these breeds. The identified signature genomic regions were associated with economic traits, including body growth, fecundity, heat stress response, domestication, environmental adaptation, hair follicle growth, horn type, and meat quality yield. Interestingly, this study identified the genomic regions associated with hair follicle growth in Chanthangi, fecundity and the number of lambs born alive in Garole, and meat quality traits in Deccani. Our research findings indicate the requisite knowledge to better comprehend the molecular processes implicated in crucial traits and local adaptation. The potential limitations of the present study include the small sample size and the ascertainment bias in the genomic data. The genotypic data from the SNP chip used in the selection signature studies is derived from a restricted set of known markers. Furthermore, alleles with a low minor allele frequency (MAF) are often overlooked. This ascertainment bias may result in misleading findings. The bottleneck effect, founder effects, and reduced recombination rates can create patterns that resemble those caused by natural selection in small sample

sizes. However, whole genome studies with better depth of sequencing, greater population size, and phenotypic observations could uncover the potential candidate genes under selection.

Declarations

Ethics approval and consent to participate

Not applicable

Consent for publication

Not applicable

Data Availability Statement

The genotypic data of indigenous Sheep breeds of India (Chantangi, Garole, and Deccani; n=29, 24, and 26, respectively) were obtained from the WIDDE database with the following link: http://widde.toulouse.inra.fr/widde/widde/main.do?module=sheep.

Competing interests

The authors declare that they have no competing interests.

Funding

This research received no specific grant from the public, commercial, or not-for-profit funding agencies.

Authors' contributions

SKI and SN conceived the idea, SN, SKI, DWM, SSM, AM and VKY performed the entire bioinformatics analysis, SKI and SN wrote the manuscript, SSM, DWM, AM and VKY edited and proofread the manuscript, and DWM and SKI supervised the whole project. All authors read the manuscript and approved the final manuscript.

Acknowledgements

The first and second authors duly acknowledge the Sri Venkateswara Veterinary University, Tirupati, Andhra Pradesh, for the necessary help and support in conducting this study.

References:

[1] Darwin C. 1859The origin of species. London: Murray: 1962.

[2] Fay JC, Wu CI. Hitchhiking under positive Darwinian selection. Genetics 2000;155:1405–13. https://doi.org/10.1093/genetics/155.3.1405.

[3] Jensen JD, Foll M, Bernatchez L. The past, present and future of genomic scans for selection. Mol Ecol 2016. https://doi.org/10.1111/mec.13493.

[4] Kim Y, Nielsen R. Linkage Disequilibrium as a Signature of Selective Sweeps 2004;1524:1513–24. https://doi.org/10.1534/genetics.103.025387.

[5] Qanbari S, Simianer H. Mapping signatures of positive selection in the genome of livestock. Livest Sci 2014;166:133–43. https://doi.org/10.1016/j.livsci.2014.05.003.

[6] Illa SK, Mukherjee S, Nath S, Mukherjee A. Genome-Wide Scanning for Signatures of Selection Revealed the Putative Genomic Regions and Candidate Genes Controlling Milk

Composition and Coat Color Traits in Sahiwal Cattle 2021;12:1–14. https://doi.org/10.3389/fgene.2021.699422.

[7] Karsli T. Genome-wide genetic characterization and selection signatures in Anatolian Merino sheep. Arch Anim Breed 2025;68:161–9. https://doi.org/10.5194/aab-68-161-2025.

[8] Karabaş M, Yılmaz O. Identification of selection signatures and genetic diversity in the sheep. Trop Anim Health Prod 2025;57:68. https://doi.org/10.1007/s11250-025-04307-9.

[9] Rodrigues JL, Braga LG, Watanabe RN, Schenkel FS, Berry DP, Buzanskas ME, et al. Genetic diversity and selection signatures in sheep breeds. J Appl Genet 2025. https://doi.org/10.1007/s13353-025-00941-z.

[10] Grossman SR, Shylakhter I, Karlsson EK, Byrne EH, Morales S, Frieden G, et al. A composite of multiple signals distinguishes causal variants in regions of positive selection. Science (80-) 2010. https://doi.org/10.1126/science.1183863.

[11] Staubach F, Lorenc A, Messer PW, Tang K, Petrov DA, Tautz D. Genome Patterns of Selection and Introgression of Haplotypes in Natural Populations of the House Mouse (Mus musculus). PLoS Genet 2012;8. https://doi.org/10.1371/journal.pgen.1002891.

[12] Ma Y, Ding X, Qanbari S, Weigend S, Zhang Q, Simianer H. Properties of different selection signature statistics and a new strategy for combining them. Heredity (Edinb) 2015. https://doi.org/10.1038/hdy.2015.42.

[13] Lotterhos KE, Card DC, Schaal SM, Wang L, Collins C, Verity B. Composite measures of selection can improve the signal-to-noise ratio in genome scans. Methods Ecol Evol 2017.

https://doi.org/10.1111/2041-210X.12774.

[14] Kijas JW, Lenstra JA, Hayes B, Boitard S, Neto LR, Cristobal MS, et al. Genome-wide analysis of the world's sheep breeds reveals high levels of historic mixture and strong recent selection. PLoS Biol 2012;10. https://doi.org/10.1371/journal.pbio.1001258.

[15] DADHF. Annual report. 2023.

[16] Saravanan KA, Panigrahi M, Kumar H, Parida S, Bhushan B, Gaur GK, et al. Genomic scans for selection signatures revealed candidate genes for adaptation and production traits in a variety of cattle breeds. Genomics 2021;113:955–63. https://doi.org/10.1016/j.ygeno.2021.02.009.

[17] Ahmad SF, Mehrotra A, Charles S, Ganai NA. Analysis of selection signatures reveals important insights into the adaptability of high-altitude Indian sheep breed Changthangi. Gene 2021;799:145809. https://doi.org/10.1016/j.gene.2021.145809.

[18] Sempéré G, Moazami-Goudarzi K, Eggen A, Laloë D, Gautier M, Flori L. WIDDE: A Web-Interfaced next generation database for genetic diversity exploration, with a first application in cattle. BMC Genomics 2015;16:1–8. https://doi.org/10.1186/s12864-015-2181-1.

[19] Cao Y, Jia P, Wu Z, Huang M, Chen S, Zhang J, et al. A novel SNP of MYO1A gene associated with heat-tolerance in Chinese cattle. Anim Biotechnol 2020;0:1–6. https://doi.org/10.1080/10495398.2020.1837147.

[20] Zheng X, Levine D, Shen J, Gogarten SM, Laurie C, Weir BS. A high-performance computing toolset for relatedness and principal component analysis of SNP data.

Bioinformatics 2012. https://doi.org/10.1093/bioinformatics/bts606.

[21] Nei M, Li WH. Mathematical model for studying genetic variation in terms of restriction endonucleases. Proc Natl Acad Sci U S A 1979. https://doi.org/10.1073/pnas.76.10.5269.

[22] Weir BS, Cockerham CC. Estimating F-statistics for the analysis of population structure. Evolution (N Y) 1984. https://doi.org/10.1111/j.1558-5646.1984.tb05657.x.

[23] Tajima F. Statistical method for testing the neutral mutation hypothesis by DNA polymorphism. Genetics 1989.

[24] Garud NR, Messer PW, Buzbas EO, Petrov DA. Recent Selective Sweeps in North American Drosophila melanogaster Show Signatures of Soft Sweeps. PLoS Genet 2015. https://doi.org/10.1371/journal.pgen.1005004.

[25] Delaneau O, Marchini J, Zagury JF. A linear complexity phasing method for thousands of genomes. Nat Methods 2012. https://doi.org/10.1038/nmeth.1785.

[26] Barbato M, Orozco-terWengel P, Tapio M, Bruford MW. SNeP: A tool to estimate trends in recent effective population size trajectories using genome-wide SNP data. Front Genet 2015. https://doi.org/10.3389/fgene.2015.00109.

[27] Petit M, Astruc JM, Sarry J, Drouilhet L, Fabre S, Moreno CR, et al. Variation in recombination rate and its genetic determinism in sheep populations. Genetics 2017;207:767–84. https://doi.org/10.1534/genetics.117.300123.

[28] Danecek P, Auton A, Abecasis G, Albers CA, Banks E, DePristo MA, et al. The variant call format and VCFtools. Bioinformatics 2011. https://doi.org/10.1093/bioinformatics/btr330.

[29] Yurchenko AA, Daetwyler HD, Yudin N, Schnabel RD, Vander Jagt CJ, Soloshenko V, et al. Scans for signatures of selection in Russian cattle breed genomes reveal new candidate genes for environmental adaptation and acclimation. Sci Rep 2018;8:1–16. https://doi.org/10.1038/s41598-018-31304-w.

[30] Verity R, Collins C, Card D, Schaal S, Wang L, Lotterhos K. MINOTAUR: A platform for the analysis and visualization of multivariate results from genome scans with R Shiny. Minotaur A Platf Anal Vis Multivar Results from Genome Scans with R Shiny 2016. https://doi.org/10.1101/062158.

[31] Todorov V, Templ M, Filzmoser P. Detection of multivariate outliers in business survey data with incomplete information. Adv Data Anal Classif 2011. https://doi.org/10.1007/s11634-010-0075-2.

[32] Venables WN, Ripley BD. Modern Applied Statistics with S Fourth edition by. 2002.

[33] Hochberg YB and Y. Controlling the False Discovery Rate: A Practical and Powerful Approach to Multiple Testing. J R Stat Soc 1995;57:289–300.

[34] Storey JD, Tibshirani R. Statistical significance for genomewide studies. Proc Natl Acad Sci U S A 2003. https://doi.org/10.1073/pnas.1530509100.

[35] Fonseca PAS, Suárez-Vega A, Marras G, Cánovas Á. GALLO: An R package for genomic annotation and integration of multiple data sources in livestock for positional candidate loci. Gigascience 2020. https://doi.org/10.1093/gigascience/giaa149.

[36] Hu ZL, Park CA, Reecy JM. Building a livestock genetic and genomic information knowledgebase through integrative developments of Animal QTLdb and CorrDB. Nucleic

Acids Res 2019. https://doi.org/10.1093/nar/gky1084.

[37] Saravanan KA, Panigrahi M, Kumar H, Bhushan B, Dutt T, Mishra BP. Genome-wide analysis of genetic diversity and selection signatures in three Indian sheep breeds. Livest Sci 2021;243:104367. https://doi.org/10.1016/j.livsci.2020.104367.

[38] Eydivandi S, Roudbar MA, Karimi MO, Sahana G. Genomic scans for selective sweeps through haplotype homozygosity and allelic fixation in 14 indigenous sheep breeds from Middle East and South Asia. Sci Rep 2021;11:1–18. https://doi.org/10.1038/s41598-021-82625-2.

[39] Hirschhorn J, Ingelsson E, Genet N. Association analyses of 249,796 individuals reveal eighteen new loci associated with body mass index HHS Public Access Author manuscript 2011;42:937–48. https://doi.org/10.1038/ng.686.Association.

[40] Zielke LG, Bortfeldt RH, Tetens J, Brockmann GA. BDNF contributes to the genetic variance of milk fat yield in German Holstein cattle. Front Genet 2011;2:1–8. https://doi.org/10.3389/fgene.2011.00016.

[41] Rahmatalla SA, Arends D, Reissmann M, Wimmers K, Reyer H, Brockmann GA. Genome-wide association study of body morphological traits in Sudanese goats. Anim Genet 2018;49:478–82. https://doi.org/10.1111/age.12686.

[42] Duan Y, Su P, Gu Y, Lv X, Cao X, Wang S, et al. A Study of the Resistance of Hu Sheep Lambs to Escherichia coli F17 Based on Whole Genome Sequencing. Animals 2024;14:1–12. https://doi.org/10.3390/ani14010161.

[43] Purdie AC, Plain KM, Begg DJ, de Silva K, Whittington RJ. Gene expression profiles

during subclinical Mycobacterium avium subspecies paratuberculosis infection in sheep can predict disease outcome. Sci Rep 2019;9:1–15. https://doi.org/10.1038/s41598-019-44670-w.

[44] Ito M, Yamanashi Y, Toyoda Y, Izumi-Nakaseko H, Oda S, Sugiyama A, et al. Disruption of Stard10 gene alters the PPARα-mediated bile acid homeostasis. Biochim Biophys Acta - Mol Cell Biol Lipids 2013;1831:459–68. https://doi.org/10.1016/j.bbalip.2012.11.008.

[45] Yang J, Wang DF, Huang JH, Zhu QH, Luo LY, Lu R, et al. Structural variant landscapes reveal convergent signatures of evolution in sheep and goats. Genome Biol 2024;25:1–46. https://doi.org/10.1186/s13059-024-03288-6.

[46] Xu P, Wang C, Xiang W, Liang Y, Li Y, Zhang X, et al. P2RY6 Has a Critical Role in Mouse Skin Carcinogenesis by Regulating the YAP and β-Catenin Signaling Pathways. J Invest Dermatol 2022;142:2334-2342.e8. https://doi.org/10.1016/j.jid.2022.02.017.

[47] Tian D, Pei Q, Jiang H, Guo J, Ma X, Han B, et al. Comprehensive analysis of the expression profiles of mRNA, lncRNA, circRNA, and miRNA in primary hair follicles of coarse sheep fetal skin. BMC Genomics 2024;25:1–15. https://doi.org/10.1186/s12864-024-10427-7.

[48] Pan R, Qi L, Xu Z, Zhang D, Nie Q, Zhang X, et al. Weighted single-step GWAS identified candidate genes associated with carcass traits in a Chinese yellow-feathered chicken population. Poult Sci 2024;103:1–14. https://doi.org/10.1016/j.psj.2023.103341.

[49] Gu S, Huang Q, Sun C, Wen C, Yang N. Transcriptomic and epigenomic insights into pectoral muscle fiber formation at the late embryonic development in pure chicken lines. Poult Sci 2024;103:103882. https://doi.org/10.1016/j.psj.2024.103882.

[50] Al-Mamun HA, Kwan P, Clark SA, Ferdosi MH, Tellam R, Gondro C. Genome-wide association study of body weight in Australian Merino sheep reveals an orthologous region on OAR6 to human and bovine genomic regions affecting height and weight. Genet Sel Evol 2015;47:1–11. https://doi.org/10.1186/s12711-015-0142-4.

[51] Zhang L, Ma X, Xuan J, Wang H, Yuan Z, Wu M, et al. Identification of MEF2B and TRhDE gene polymorphisms related to growth traits in a new ujumqin sheep population. PLoS One 2016;11:1–12. https://doi.org/10.1371/journal.pone.0159504.

[52] Pasandideh M, Gholizadeh M, Rahimi-Mianji G. A genome-wide association study revealed five SNPs affecting 8-month weight in sheep. Anim Genet 2020;51:973–6. https://doi.org/10.1111/age.12996.

[53] Armstrong E, Ciappesoni G, Iriarte W, Da Silva C, Macedo F, Navajas EA, et al. Novel genetic polymorphisms associated with carcass traits in grazing Texel sheep. Meat Sci 2018;145:202–8. https://doi.org/10.1016/j.meatsci.2018.06.014.

[54] Gonzalez M V., Mousel MR, Herndon DR, Jiang Y, Dalrymple BP, Reynolds JO, et al. A Divergent Artiodactyl MYADM-like Repeat Is Associated with Erythrocyte Traits and Weight of Lamb Weaned in Domestic Sheep. PLoS One 2013;8. https://doi.org/10.1371/journal.pone.0074700.

[55] Li H, Wu XL, Tait RG, Bauck S, Thomas DL, Murphy TW, et al. Genome-wide association study of milk production traits in a crossbred dairy sheep population using three statistical models. Anim Genet 2020;51:624–8. https://doi.org/10.1111/age.12956.

[56] Peng WF, Xu SS, Ren X, Lv FH, Xie XL, Zhao YX, et al. A genome-wide association study reveals candidate genes for the supernumerary nipple phenotype in sheep (Ovis

aries). Anim Genet 2017;48:570–9. https://doi.org/10.1111/age.12575.

[57] Wen J, Toomer KH, Chen Z, Cai X. Genome-wide analysis of alternative transcripts in human breast cancer. Breast Cancer Res Treat 2015;151:295–307. https://doi.org/10.1007/s10549-015-3395-2.

[58] Chen W, Li Z, Zhong R, Sun W, Chu M. Expression profiles of oviductal mRNAs and lncRNAs in the follicular phase and luteal phase of sheep (Ovis aries) with 2 fecundity gene (FecB) genotypes. G3 Genes, Genomes, Genet 2024;14:1–16. https://doi.org/10.1093/g3journal/jkad270.

[59] Deng M, Zhang G, Cai Y, Liu Z, Zhang Y, Meng F, et al. DNA methylation dynamics during zygotic genome activation in goat. Theriogenology 2020;156:144–54. https://doi.org/10.1016/j.theriogenology.2020.07.008.

[60] Bao J, Xiong J, Huang J, Yang P, Shang M, Zhang L. Genetic Diversity, Selection Signatures, and Genome-Wide Association Study Identify Candidate Genes Related to Litter Size in Hu Sheep. Int J Mol Sci 2024;25. https://doi.org/10.3390/ijms25179397.

[61] Sugimoto M, Gotoh Y, Kawahara T, Sugimoto Y. Molecular effects of polymorphism in the 3’UTR of unc-5 homolog C associated with conception rate in Holsteins. PLoS One 2015;10:1–14. https://doi.org/10.1371/journal.pone.0131283.

[62] Zhao F, Xie R, Fang L, Xiang R, Yuan Z, Liu Y, et al. Analysis of 206 whole-genome resequencing reveals selection signatures associated with breed-specific traits in Hu sheep. Evol Appl 2024;17:1–12. https://doi.org/10.1111/eva.13697.

[63] Zhong T, Hou D, Zhao Q, Zhan S, Wang L, Li L, et al. Comparative whole-genome

resequencing to uncover selection signatures linked to litter size in Hu Sheep and five other breeds. BMC Genomics 2024;25:1–13. https://doi.org/10.1186/s12864-024-10396-x.

[64] Eydivandi S, Roudbar MA, Ardestani SS, Momen M, Sahana G. A selection signatures study among Middle Eastern and European sheep breeds. J Anim Breed Genet 2021;138:574–88. https://doi.org/10.1111/jbg.12536.

[65] Karaoglan I, Pehlivan S, Namiduru M, Pehlivan M, Kilinçarslan C, Balkan Y, et al. TNF-α, TGF-β, IL-10, IL-6 and IFN-γ gene polymorphisms as risk factors for brucellosis. New Microbiol 2009;32:173–8.

[66] Li X, Wu Q, Zhang X, Li C, Zhang D, Li G, et al. Whole-Genome Resequencing to Study Brucellosis Susceptibility in Sheep. Front Genet 2021;12:1–9. https://doi.org/10.3389/fgene.2021.653927.

[67] Zhao G, Li X, Miao H, Chen S, Hou Y. Estrogen promotes cAMP production in Mesenchymal stem cells by regulating ADCY2. Int J Stem Cells 2020;13:55–64. https://doi.org/10.15283/IJSC19139.

[68] Matika O, Riggio V, Anselme-Moizan M, Law AS, Pong-Wong R, Archibald AL, et al. Genome-wide association reveals QTL for growth, bone and in vivo carcass traits as assessed by computed tomography in Scottish Blackface lambs. Genet Sel Evol 2016;48:1–15. https://doi.org/10.1186/s12711-016-0191-3.

[69] Xu SS, Gao L, Xie XL, Ren YL, Shen ZQ, Wang F, et al. Genome-wide association analyses highlight the potential for different genetic mechanisms for litter size among sheep breeds. Front Genet 2018;9:1–14. https://doi.org/10.3389/fgene.2018.00118.

[70] Abdoli R, Mirhoseini SZ, Ghavi Hossein-Zadeh N, Zamani P, Moradi MH, Ferdosi MH, et al. Runs of homozygosity and cross-generational inbreeding of Iranian fat-tailed sheep. Heredity (Edinb) 2023;130:358–67. https://doi.org/10.1038/s41437-023-00611-y.

[71] Li X, Ding N, Zhang Z, Tian D, Han B, Liu S, et al. Identification of Somatostatin Receptor Subtype 1 (SSTR1) Gene Polymorphism and Their Association with Growth Traits in Hulun Buir Sheep. Genes (Basel) 2022;13. https://doi.org/10.3390/genes13010077.

[72] Chen Y, Hu S, Mu L, Zhao B, Wang M, Yang N, et al. Slc7a11 modulated by POU2f1 is involved in pigmentation in rabbit. Int J Mol Sci 2019;20:1–16. https://doi.org/10.3390/ijms20102493.

[73] Yang N, Zhao B, Hu S, Bao Z, Liu M, Chen Y, et al. Characterization of POU2F1 gene and its potential impact on the expression of genes involved in fur color formation in rex rabbit. Genes (Basel) 2020;11. https://doi.org/10.3390/genes11050575.

[74] Ahbara A, Bahbahani H, Almathen F, Abri M Al, Agoub MO, Abeba A, et al. Genome-wide variation, candidate regions and genes associated with fat deposition and tail morphology in Ethiopian indigenous sheep. Front Genet 2019;10:1–21. https://doi.org/10.3389/fgene.2018.00699.

[75] Casas E, Shackelford SD, Keele JW, Stone RT, Kappes SM, Koohmaraie M. Quantitative trait loci affecting growth and carcass composition of cattle segregating alternate forms of myostatin. J Anim Sci 2000;78:560–9. https://doi.org/10.2527/2000.783560x.

[76] McClure MC, Morsci NS, Schnabel RD, Kim JW, Yao P, Rolf MM, et al. A genome scan for quantitative trait loci influencing carcass, post-natal growth and reproductive traits in

commercial Angus cattle. Anim Genet 2010;41:597–607. https://doi.org/10.1111/j.1365-2052.2010.02063.x.

[77] Samani A, Karuppasamy M, English KG, Siler CA, Wang Y, Widrick JJ, et al. glucose metabolism 2024;37:1–27. https://doi.org/10.1096/fj.202300386RR.DOCK3.

[78] Marina H, Pelayo R, Suárez-Vega A, Gutiérrez-Gil B, Esteban-Blanco C, Arranz JJ. Genome-wide association studies (GWAS) and post-GWAS analyses for technological traits in Assaf and Churra dairy breeds. J Dairy Sci 2021;104:11850–66. https://doi.org/10.3168/jds.2021-20510.

[79] Oliveira RD, Mousel MR, Gonzalez M V., Durfee CJ, Davenport KM, Murdoch BM, et al. A high-density genome-wide association with absolute blood monocyte count in domestic sheep identifies novel loci. PLoS One 2022;17:1–15. https://doi.org/10.1371/journal.pone.0266748.

[80] Cinar MU, Oliveira RD, Hadfield TS, Lichtenwalner A, Brzozowski RJ, Settlemire CT, et al. Genome-wide association with footrot in hair and wool sheep. Front Genet 2023;14:1–9. https://doi.org/10.3389/fgene.2023.1297444.

[81] Tuersuntuoheti M, Zhang J, Zhou W, Zhang CL, Liu C, Chang Q, et al. Exploring the growth trait molecular markers in two sheep breeds based on Genome-wide association analysis. PLoS One 2023;18:1–18. https://doi.org/10.1371/journal.pone.0283383.

[82] Revelo HA, López-Alvarez D, Palacios YA, Vergara OD, Yánez MB, Ariza MF, et al. Genome-wide association study reveals candidate genes for traits related to meat quality in Colombian Creole hair sheep. Trop Anim Health Prod 2023;55:1–13. https://doi.org/10.1007/s11250-023-03688-z.

[83] Johnston SE, McEwan JC, Pickering NK, Kijas JW, Beraldi D, Pilkington JG, et al. Genome-wide association mapping identifies the genetic basis of discrete and quantitative variation in sexual weaponry in a wild sheep population. Mol Ecol 2011;20:2555–66. https://doi.org/10.1111/j.1365-294X.2011.05076.x.

[1] Darwin C. 1859The origin of species. London: Murray: 1962.

[2] Fay JC, Wu CI. Hitchhiking under positive Darwinian selection. Genetics 2000;155:1405–13. https://doi.org/10.1093/genetics/155.3.1405.

[3] Jensen JD, Foll M, Bernatchez L. The past, present and future of genomic scans for selection. Mol Ecol 2016. https://doi.org/10.1111/mec.13493.

[4] Kim Y, Nielsen R. Linkage Disequilibrium as a Signature of Selective Sweeps 2004;1524:1513–24. https://doi.org/10.1534/genetics.103.025387.

[5] Qanbari S, Simianer H. Mapping signatures of positive selection in the genome of livestock. Livest Sci 2014;166:133–43. https://doi.org/10.1016/j.livsci.2014.05.003.

[6] Illa SK, Mukherjee S, Nath S, Mukherjee A. Genome-Wide Scanning for Signatures of Selection Revealed the Putative Genomic Regions and Candidate Genes Controlling Milk Composition and Coat Color Traits in Sahiwal Cattle 2021;12:1–14. https://doi.org/10.3389/fgene.2021.699422.

[7] Karsli T. Genome-wide genetic characterization and selection signatures in Anatolian Merino sheep. Arch Anim Breed 2025;68:161–9. https://doi.org/10.5194/aab-68-161-2025.

[8] Karabaş M, Yılmaz O. Identification of selection signatures and genetic diversity in the

sheep. Trop Anim Health Prod 2025;57:68. https://doi.org/10.1007/s11250-025-04307-9.

[9] Rodrigues JL, Braga LG, Watanabe RN, Schenkel FS, Berry DP, Buzanskas ME, et al. Genetic diversity and selection signatures in sheep breeds. J Appl Genet 2025. https://doi.org/10.1007/s13353-025-00941-z.

[10] Grossman SR, Shylakhter I, Karlsson EK, Byrne EH, Morales S, Frieden G, et al. A composite of multiple signals distinguishes causal variants in regions of positive selection. Science (80-) 2010. https://doi.org/10.1126/science.1183863.

[11] Staubach F, Lorenc A, Messer PW, Tang K, Petrov DA, Tautz D. Genome Patterns of Selection and Introgression of Haplotypes in Natural Populations of the House Mouse (Mus musculus). PLoS Genet 2012;8. https://doi.org/10.1371/journal.pgen.1002891.

[12] Ma Y, Ding X, Qanbari S, Weigend S, Zhang Q, Simianer H. Properties of different selection signature statistics and a new strategy for combining them. Heredity (Edinb) 2015. https://doi.org/10.1038/hdy.2015.42.

[13] Lotterhos KE, Card DC, Schaal SM, Wang L, Collins C, Verity B. Composite measures of selection can improve the signal-to-noise ratio in genome scans. Methods Ecol Evol 2017. https://doi.org/10.1111/2041-210X.12774.

[14] Kijas JW, Lenstra JA, Hayes B, Boitard S, Neto LR, Cristobal MS, et al. Genome-wide analysis of the world's sheep breeds reveals high levels of historic mixture and strong recent selection. PLoS Biol 2012;10. https://doi.org/10.1371/journal.pbio.1001258.

[15] DADHF. Annual report. 2023.

[16] Saravanan KA, Panigrahi M, Kumar H, Parida S, Bhushan B, Gaur GK, et al. Genomic

scans for selection signatures revealed candidate genes for adaptation and production traits in a variety of cattle breeds. Genomics 2021;113:955–63. https://doi.org/10.1016/j.ygeno.2021.02.009.

[17] Ahmad SF, Mehrotra A, Charles S, Ganai NA. Analysis of selection signatures reveals important insights into the adaptability of high-altitude Indian sheep breed Changthangi. Gene 2021;799:145809. https://doi.org/10.1016/j.gene.2021.145809.

[18] Sempéré G, Moazami-Goudarzi K, Eggen A, Laloë D, Gautier M, Flori L. WIDDE: A Web-Interfaced next generation database for genetic diversity exploration, with a first application in cattle. BMC Genomics 2015;16:1–8. https://doi.org/10.1186/s12864-015-2181-1.

[19] Cao Y, Jia P, Wu Z, Huang M, Chen S, Zhang J, et al. A novel SNP of MYO1A gene associated with heat-tolerance in Chinese cattle. Anim Biotechnol 2020;0:1–6. https://doi.org/10.1080/10495398.2020.1837147.

[20] Zheng X, Levine D, Shen J, Gogarten SM, Laurie C, Weir BS. A high-performance computing toolset for relatedness and principal component analysis of SNP data. Bioinformatics 2012. https://doi.org/10.1093/bioinformatics/bts606.

[21] Nei M, Li WH. Mathematical model for studying genetic variation in terms of restriction endonucleases. Proc Natl Acad Sci U S A 1979. https://doi.org/10.1073/pnas.76.10.5269.

[22] Weir BS, Cockerham CC. Estimating F-statistics for the analysis of population structure. Evolution (N Y) 1984. https://doi.org/10.1111/j.1558-5646.1984.tb05657.x.

[23] Tajima F. Statistical method for testing the neutral mutation hypothesis by DNA

polymorphism. Genetics 1989.

[24] Garud NR, Messer PW, Buzbas EO, Petrov DA. Recent Selective Sweeps in North American Drosophila melanogaster Show Signatures of Soft Sweeps. PLoS Genet 2015. https://doi.org/10.1371/journal.pgen.1005004.

[25] Delaneau O, Marchini J, Zagury JF. A linear complexity phasing method for thousands of genomes. Nat Methods 2012. https://doi.org/10.1038/nmeth.1785.

[26] Barbato M, Orozco-terWengel P, Tapio M, Bruford MW. SNeP: A tool to estimate trends in recent effective population size trajectories using genome-wide SNP data. Front Genet 2015. https://doi.org/10.3389/fgene.2015.00109.

[27] Petit M, Astruc JM, Sarry J, Drouilhet L, Fabre S, Moreno CR, et al. Variation in recombination rate and its genetic determinism in sheep populations. Genetics 2017;207:767–84. https://doi.org/10.1534/genetics.117.300123.

[28] Danecek P, Auton A, Abecasis G, Albers CA, Banks E, DePristo MA, et al. The variant call format and VCFtools. Bioinformatics 2011. https://doi.org/10.1093/bioinformatics/btr330.

[29] Yurchenko AA, Daetwyler HD, Yudin N, Schnabel RD, Vander Jagt CJ, Soloshenko V, et al. Scans for signatures of selection in Russian cattle breed genomes reveal new candidate genes for environmental adaptation and acclimation. Sci Rep 2018;8:1–16. https://doi.org/10.1038/s41598-018-31304-w.

[30] Verity R, Collins C, Card D, Schaal S, Wang L, Lotterhos K. MINOTAUR: A platform for the analysis and visualization of multivariate results from genome scans with R Shiny.

Minotaur A Platf Anal Vis Multivar Results from Genome Scans with R Shiny 2016. https://doi.org/10.1101/062158.

[31] Todorov V, Templ M, Filzmoser P. Detection of multivariate outliers in business survey data with incomplete information. Adv Data Anal Classif 2011. https://doi.org/10.1007/s11634-010-0075-2.

[32] Venables WN, Ripley BD. Modern Applied Statistics with S Fourth edition by. 2002.

[33] Hochberg YB and Y. Controlling the False Discovery Rate: A Practical and Powerful Approach to Multiple Testing. J R Stat Soc 1995;57:289–300.

[34] Storey JD, Tibshirani R. Statistical significance for genomewide studies. Proc Natl Acad Sci U S A 2003. https://doi.org/10.1073/pnas.1530509100.

[35] Fonseca PAS, Suárez-Vega A, Marras G, Cánovas Á. GALLO: An R package for genomic annotation and integration of multiple data sources in livestock for positional candidate loci. Gigascience 2020. https://doi.org/10.1093/gigascience/giaa149.

[36] Hu ZL, Park CA, Reecy JM. Building a livestock genetic and genomic information knowledgebase through integrative developments of Animal QTLdb and CorrDB. Nucleic Acids Res 2019. https://doi.org/10.1093/nar/gky1084.

[37] Saravanan KA, Panigrahi M, Kumar H, Bhushan B, Dutt T, Mishra BP. Genome-wide analysis of genetic diversity and selection signatures in three Indian sheep breeds. Livest Sci 2021;243:104367. https://doi.org/10.1016/j.livsci.2020.104367.

[38] Eydivandi S, Roudbar MA, Karimi MO, Sahana G. Genomic scans for selective sweeps through haplotype homozygosity and allelic fixation in 14 indigenous sheep breeds from

Middle East and South Asia. Sci Rep 2021;11:1–18. https://doi.org/10.1038/s41598-021-82625-2.

[39] Hirschhorn J, Ingelsson E, Genet N. Association analyses of 249,796 individuals reveal eighteen new loci associated with body mass index HHS Public Access Author manuscript 2011;42:937–48. https://doi.org/10.1038/ng.686.Association.

[40] Zielke LG, Bortfeldt RH, Tetens J, Brockmann GA. BDNF contributes to the genetic variance of milk fat yield in German Holstein cattle. Front Genet 2011;2:1–8. https://doi.org/10.3389/fgene.2011.00016.

[41] Rahmatalla SA, Arends D, Reissmann M, Wimmers K, Reyer H, Brockmann GA. Genome-wide association study of body morphological traits in Sudanese goats. Anim Genet 2018;49:478–82. https://doi.org/10.1111/age.12686.

[42] Duan Y, Su P, Gu Y, Lv X, Cao X, Wang S, et al. A Study of the Resistance of Hu Sheep Lambs to Escherichia coli F17 Based on Whole Genome Sequencing. Animals 2024;14:1–12. https://doi.org/10.3390/ani14010161.

[43] Purdie AC, Plain KM, Begg DJ, de Silva K, Whittington RJ. Gene expression profiles during subclinical Mycobacterium avium subspecies paratuberculosis infection in sheep can predict disease outcome. Sci Rep 2019;9:1–15. https://doi.org/10.1038/s41598-019-44670-w.

[44] Ito M, Yamanashi Y, Toyoda Y, Izumi-Nakaseko H, Oda S, Sugiyama A, et al. Disruption of Stard10 gene alters the PPARα-mediated bile acid homeostasis. Biochim Biophys Acta - Mol Cell Biol Lipids 2013;1831:459–68. https://doi.org/10.1016/j.bbalip.2012.11.008.

[45] Yang J, Wang DF, Huang JH, Zhu QH, Luo LY, Lu R, et al. Structural variant landscapes reveal convergent signatures of evolution in sheep and goats. Genome Biol 2024;25:1–46. https://doi.org/10.1186/s13059-024-03288-6.

[46] Xu P, Wang C, Xiang W, Liang Y, Li Y, Zhang X, et al. P2RY6 Has a Critical Role in Mouse Skin Carcinogenesis by Regulating the YAP and β-Catenin Signaling Pathways. J Invest Dermatol 2022;142:2334-2342.e8. https://doi.org/10.1016/j.jid.2022.02.017.

[47] Tian D, Pei Q, Jiang H, Guo J, Ma X, Han B, et al. Comprehensive analysis of the expression profiles of mRNA, lncRNA, circRNA, and miRNA in primary hair follicles of coarse sheep fetal skin. BMC Genomics 2024;25:1–15. https://doi.org/10.1186/s12864-024-10427-7.

[48] Pan R, Qi L, Xu Z, Zhang D, Nie Q, Zhang X, et al. Weighted single-step GWAS identified candidate genes associated with carcass traits in a Chinese yellow-feathered chicken population. Poult Sci 2024;103:1–14. https://doi.org/10.1016/j.psj.2023.103341.

[49] Gu S, Huang Q, Sun C, Wen C, Yang N. Transcriptomic and epigenomic insights into pectoral muscle fiber formation at the late embryonic development in pure chicken lines. Poult Sci 2024;103:103882. https://doi.org/10.1016/j.psj.2024.103882.

[50] Al-Mamun HA, Kwan P, Clark SA, Ferdosi MH, Tellam R, Gondro C. Genome-wide association study of body weight in Australian Merino sheep reveals an orthologous region on OAR6 to human and bovine genomic regions affecting height and weight. Genet Sel Evol 2015;47:1–11. https://doi.org/10.1186/s12711-015-0142-4.

[51] Zhang L, Ma X, Xuan J, Wang H, Yuan Z, Wu M, et al. Identification of MEF2B and TRhDE gene polymorphisms related to growth traits in a new ujumqin sheep population.

PLoS One 2016;11:1–12. https://doi.org/10.1371/journal.pone.0159504.

[52] Pasandideh M, Gholizadeh M, Rahimi-Mianji G. A genome-wide association study revealed five SNPs affecting 8-month weight in sheep. Anim Genet 2020;51:973–6. https://doi.org/10.1111/age.12996.

[53] Armstrong E, Ciappesoni G, Iriarte W, Da Silva C, Macedo F, Navajas EA, et al. Novel genetic polymorphisms associated with carcass traits in grazing Texel sheep. Meat Sci 2018;145:202–8. https://doi.org/10.1016/j.meatsci.2018.06.014.

[54] Gonzalez M V., Mousel MR, Herndon DR, Jiang Y, Dalrymple BP, Reynolds JO, et al. A Divergent Artiodactyl MYADM-like Repeat Is Associated with Erythrocyte Traits and Weight of Lamb Weaned in Domestic Sheep. PLoS One 2013;8. https://doi.org/10.1371/journal.pone.0074700.

[55] Li H, Wu XL, Tait RG, Bauck S, Thomas DL, Murphy TW, et al. Genome-wide association study of milk production traits in a crossbred dairy sheep population using three statistical models. Anim Genet 2020;51:624–8. https://doi.org/10.1111/age.12956.

[56] Peng WF, Xu SS, Ren X, Lv FH, Xie XL, Zhao YX, et al. A genome-wide association study reveals candidate genes for the supernumerary nipple phenotype in sheep (Ovis aries). Anim Genet 2017;48:570–9. https://doi.org/10.1111/age.12575.

[57] Wen J, Toomer KH, Chen Z, Cai X. Genome-wide analysis of alternative transcripts in human breast cancer. Breast Cancer Res Treat 2015;151:295–307. https://doi.org/10.1007/s10549-015-3395-2.

[58] Chen W, Li Z, Zhong R, Sun W, Chu M. Expression profiles of oviductal mRNAs and

lncRNAs in the follicular phase and luteal phase of sheep (Ovis aries) with 2 fecundity gene (FecB) genotypes. G3 Genes, Genomes, Genet 2024;14:1–16. https://doi.org/10.1093/g3journal/jkad270.

[59] Deng M, Zhang G, Cai Y, Liu Z, Zhang Y, Meng F, et al. DNA methylation dynamics during zygotic genome activation in goat. Theriogenology 2020;156:144–54. https://doi.org/10.1016/j.theriogenology.2020.07.008.

[60] Bao J, Xiong J, Huang J, Yang P, Shang M, Zhang L. Genetic Diversity, Selection Signatures, and Genome-Wide Association Study Identify Candidate Genes Related to Litter Size in Hu Sheep. Int J Mol Sci 2024;25. https://doi.org/10.3390/ijms25179397.

[61] Sugimoto M, Gotoh Y, Kawahara T, Sugimoto Y. Molecular effects of polymorphism in the 3'UTR of unc-5 homolog C associated with conception rate in Holsteins. PLoS One 2015;10:1–14. https://doi.org/10.1371/journal.pone.0131283.

[62] Zhao F, Xie R, Fang L, Xiang R, Yuan Z, Liu Y, et al. Analysis of 206 whole-genome resequencing reveals selection signatures associated with breed-specific traits in Hu sheep. Evol Appl 2024;17:1–12. https://doi.org/10.1111/eva.13697.

[63] Zhong T, Hou D, Zhao Q, Zhan S, Wang L, Li L, et al. Comparative whole-genome resequencing to uncover selection signatures linked to litter size in Hu Sheep and five other breeds. BMC Genomics 2024;25:1–13. https://doi.org/10.1186/s12864-024-10396-x.

[64] Eydivandi S, Roudbar MA, Ardestani SS, Momen M, Sahana G. A selection signatures study among Middle Eastern and European sheep breeds. J Anim Breed Genet 2021;138:574–88. https://doi.org/10.1111/jbg.12536.

[65] Karaoglan I, Pehlivan S, Namiduru M, Pehlivan M, Kilinçarslan C, Balkan Y, et al. TNF-α, TGF-β, IL-10, IL-6 and IFN-γ gene polymorphisms as risk factors for brucellosis. New Microbiol 2009;32:173–8.

[66] Li X, Wu Q, Zhang X, Li C, Zhang D, Li G, et al. Whole-Genome Resequencing to Study Brucellosis Susceptibility in Sheep. Front Genet 2021;12:1–9. https://doi.org/10.3389/fgene.2021.653927.

[67] Zhao G, Li X, Miao H, Chen S, Hou Y. Estrogen promotes cAMP production in Mesenchymal stem cells by regulating ADCY2. Int J Stem Cells 2020;13:55–64. https://doi.org/10.15283/IJSC19139.

[68] Matika O, Riggio V, Anselme-Moizan M, Law AS, Pong-Wong R, Archibald AL, et al. Genome-wide association reveals QTL for growth, bone and in vivo carcass traits as assessed by computed tomography in Scottish Blackface lambs. Genet Sel Evol 2016;48:1–15. https://doi.org/10.1186/s12711-016-0191-3.

[69] Xu SS, Gao L, Xie XL, Ren YL, Shen ZQ, Wang F, et al. Genome-wide association analyses highlight the potential for different genetic mechanisms for litter size among sheep breeds. Front Genet 2018;9:1–14. https://doi.org/10.3389/fgene.2018.00118.

[70] Abdoli R, Mirhoseini SZ, Ghavi Hossein-Zadeh N, Zamani P, Moradi MH, Ferdosi MH, et al. Runs of homozygosity and cross-generational inbreeding of Iranian fat-tailed sheep. Heredity (Edinb) 2023;130:358–67. https://doi.org/10.1038/s41437-023-00611-y.

[71] Li X, Ding N, Zhang Z, Tian D, Han B, Liu S, et al. Identification of Somatostatin Receptor Subtype 1 (SSTR1) Gene Polymorphism and Their Association with Growth Traits in Hulun Buir Sheep. Genes (Basel) 2022;13.

https://doi.org/10.3390/genes13010077.

[72] Chen Y, Hu S, Mu L, Zhao B, Wang M, Yang N, et al. Slc7a11 modulated by POU2f1 is involved in pigmentation in rabbit. Int J Mol Sci 2019;20:1–16. https://doi.org/10.3390/ijms20102493.

[73] Yang N, Zhao B, Hu S, Bao Z, Liu M, Chen Y, et al. Characterization of POU2F1 gene and its potential impact on the expression of genes involved in fur color formation in rex rabbit. Genes (Basel) 2020;11. https://doi.org/10.3390/genes11050575.

[74] Ahbara A, Bahbahani H, Almathen F, Abri M Al, Agoub MO, Abeba A, et al. Genome-wide variation, candidate regions and genes associated with fat deposition and tail morphology in Ethiopian indigenous sheep. Front Genet 2019;10:1–21. https://doi.org/10.3389/fgene.2018.00699.

[75] Casas E, Shackelford SD, Keele JW, Stone RT, Kappes SM, Koohmaraie M. Quantitative trait loci affecting growth and carcass composition of cattle segregating alternate forms of myostatin. J Anim Sci 2000;78:560–9. https://doi.org/10.2527/2000.783560x.

[76] McClure MC, Morsci NS, Schnabel RD, Kim JW, Yao P, Rolf MM, et al. A genome scan for quantitative trait loci influencing carcass, post-natal growth and reproductive traits in commercial Angus cattle. Anim Genet 2010;41:597–607. https://doi.org/10.1111/j.1365-2052.2010.02063.x.

[77] Samani A, Karuppasamy M, English KG, Siler CA, Wang Y, Widrick JJ, et al. glucose metabolism 2024;37:1–27. https://doi.org/10.1096/fj.202300386RR.DOCK3.

[78] Marina H, Pelayo R, Suárez-Vega A, Gutiérrez-Gil B, Esteban-Blanco C, Arranz JJ.

Genome-wide association studies (GWAS) and post-GWAS analyses for technological traits in Assaf and Churra dairy breeds. J Dairy Sci 2021;104:11850–66. https://doi.org/10.3168/jds.2021-20510.

[79] Oliveira RD, Mousel MR, Gonzalez M V., Durfee CJ, Davenport KM, Murdoch BM, et al. A high-density genome-wide association with absolute blood monocyte count in domestic sheep identifies novel loci. PLoS One 2022;17:1–15. https://doi.org/10.1371/journal.pone.0266748.

[80] Cinar MU, Oliveira RD, Hadfield TS, Lichtenwalner A, Brzozowski RJ, Settlemire CT, et al. Genome-wide association with footrot in hair and wool sheep. Front Genet 2023;14:1–9. https://doi.org/10.3389/fgene.2023.1297444.

[81] Tuersuntuoheti M, Zhang J, Zhou W, Zhang CL, Liu C, Chang Q, et al. Exploring the growth trait molecular markers in two sheep breeds based on Genome-wide association analysis. PLoS One 2023;18:1–18. https://doi.org/10.1371/journal.pone.0283383.

[82] Revelo HA, López-Alvarez D, Palacios YA, Vergara OD, Yánez MB, Ariza MF, et al. Genome-wide association study reveals candidate genes for traits related to meat quality in Colombian Creole hair sheep. Trop Anim Health Prod 2023;55:1–13. https://doi.org/10.1007/s11250-023-03688-z.

[83] Johnston SE, McEwan JC, Pickering NK, Kijas JW, Beraldi D, Pilkington JG, et al. Genome-wide association mapping identifies the genetic basis of discrete and quantitative variation in sexual weaponry in a wild sheep population. Mol Ecol 2011;20:2555–66. https://doi.org/10.1111/j.1365-294X.2011.05076.x.

Fig 1: PCA analysis showing the population structure of the three Indian sheep breeds.

Fig 2: Percent QTL classes annotated in three Indian sheep breeds

Fig 3: QTL classes enriched in three Indian sheep breeds

Fig 4: Manhattan plot showing signatures of selection in three Indian sheep breeds

Click here to access/download;Figure;Fig 1.tiff

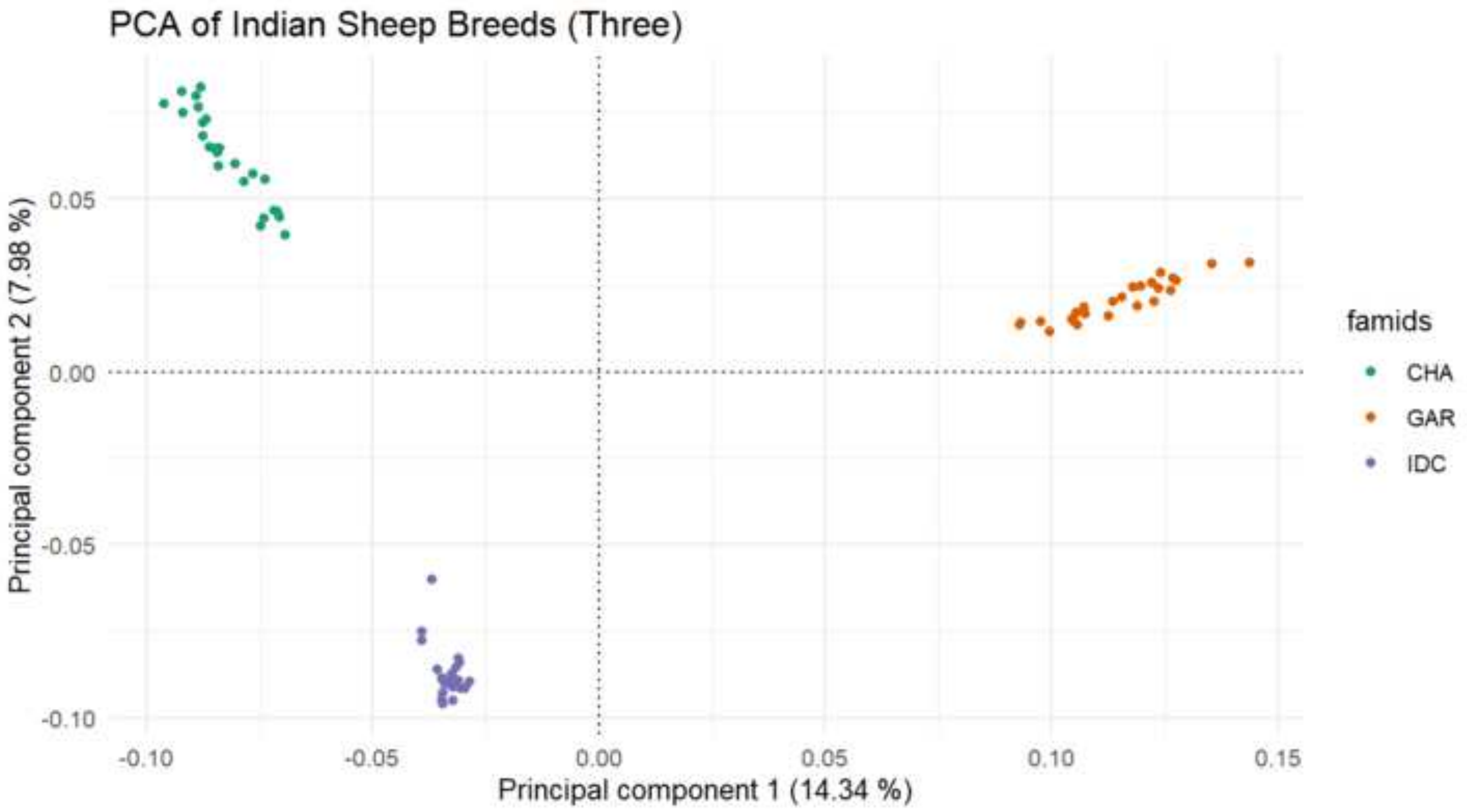

 Click here to access/download;Figure;Fig 2.tiff

Figure 2 Percent QTL classes annotated in three Indian sheep breeds

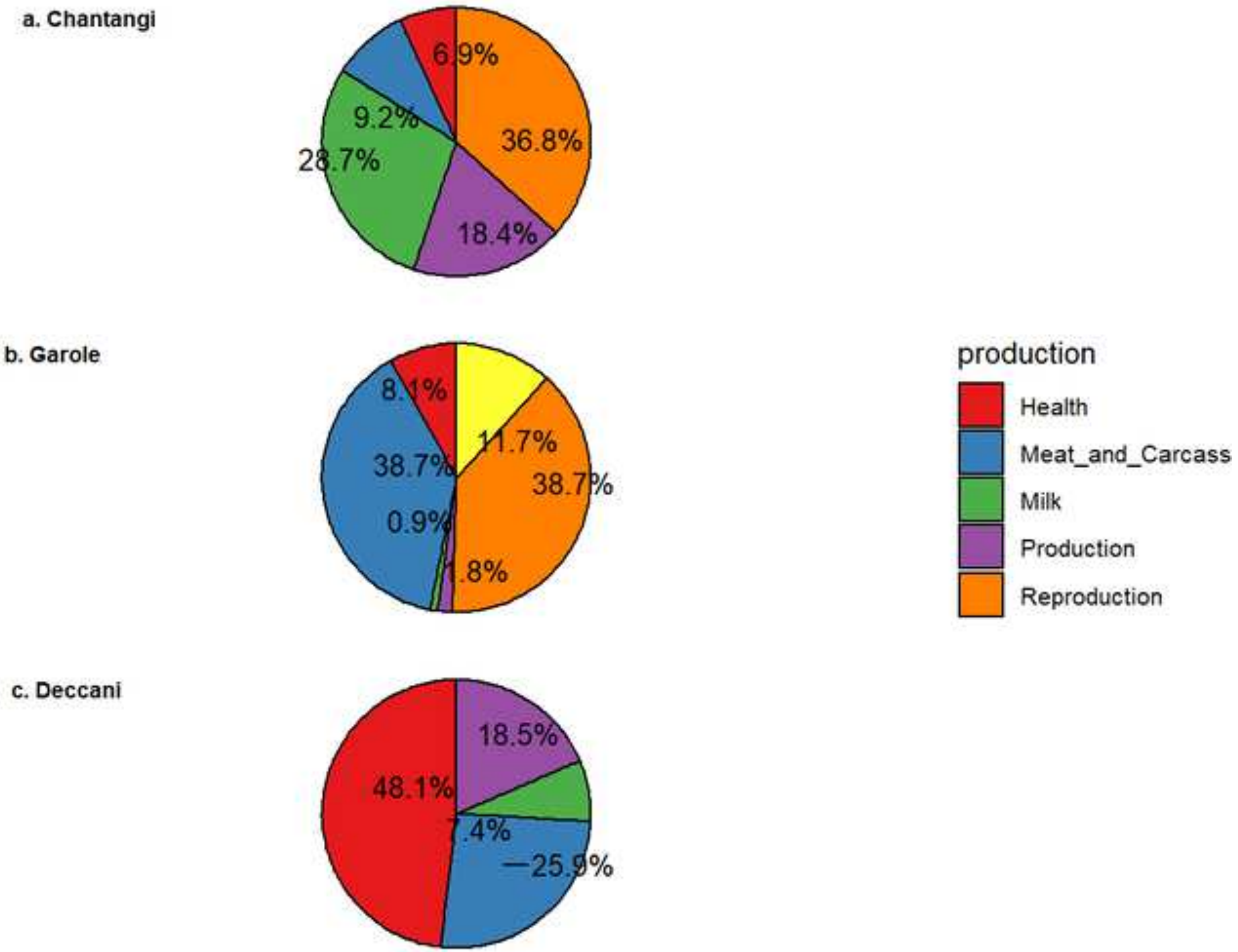

Click here to access/download;Figure;Fig 3.tiff

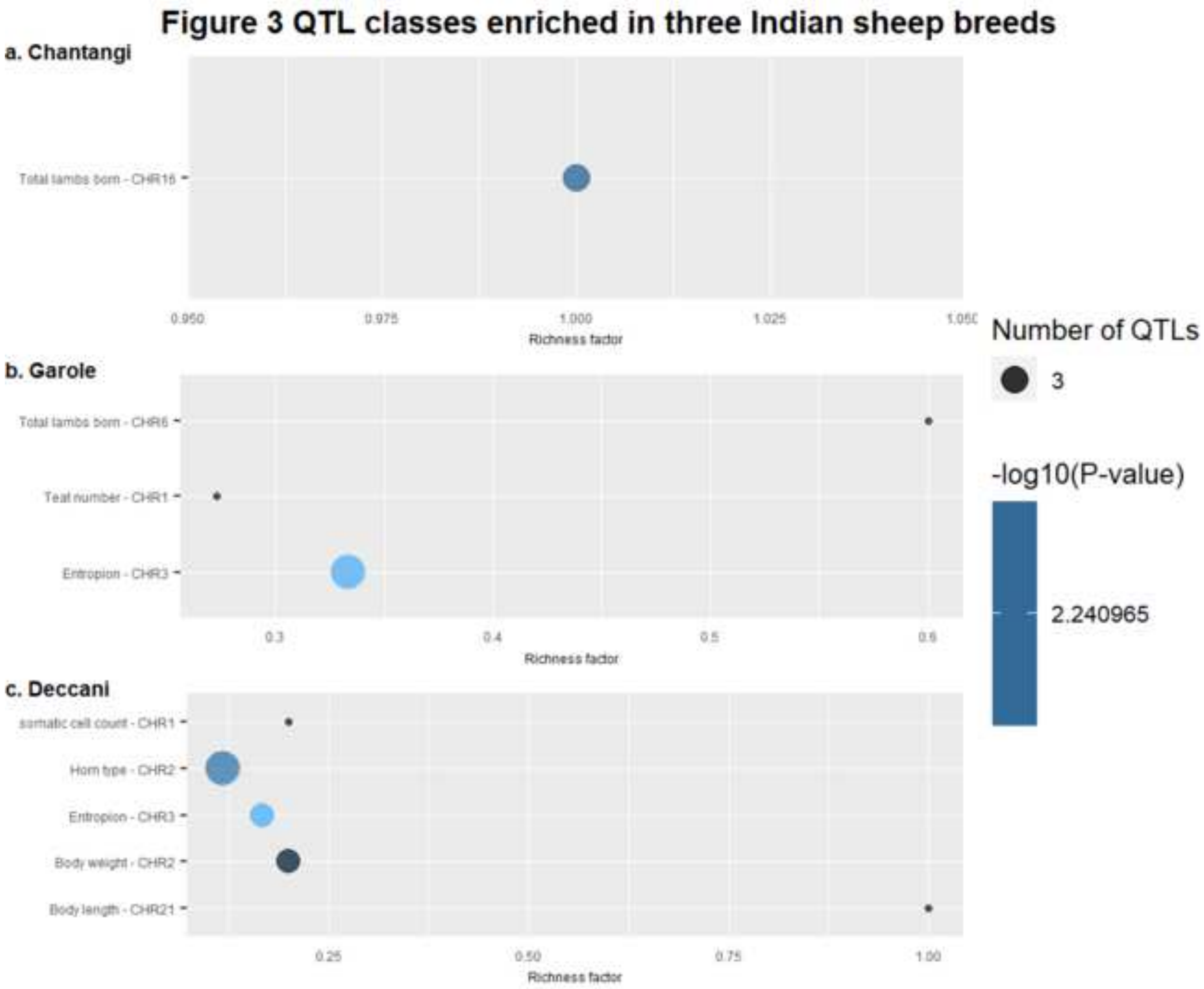

Figure

Click here to access/download;Figure;Fig 4.tiff

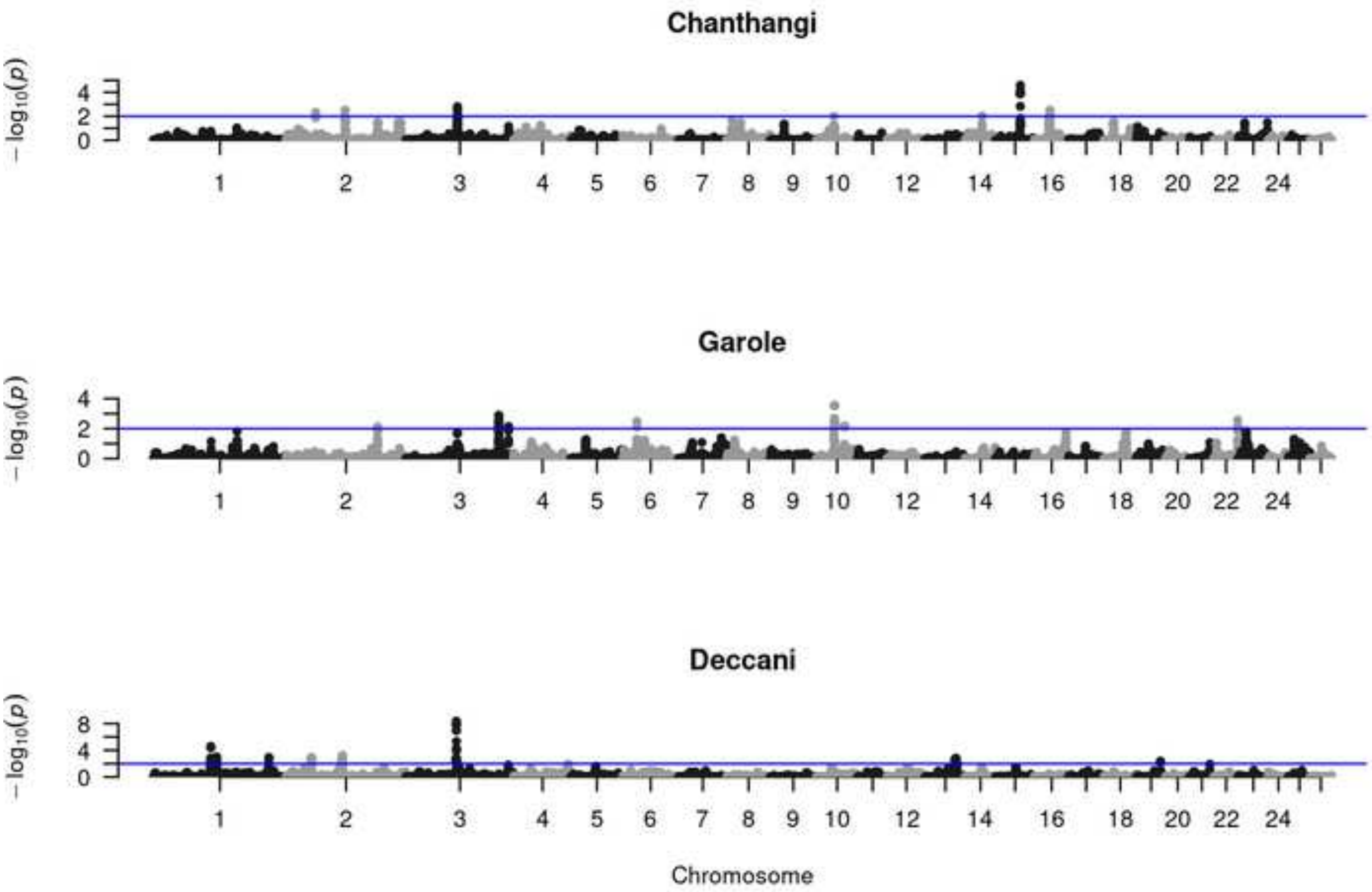

Composite Selection Signal Analysis: Uncovering Candidate Genes and Quantitative Trait Loci in Indian Sheep Breeds

Sapna Nath[1¶], Satish Kumar Illa[2¶], Destaw Worku Mengistu[3*], Sabyasachi Mukherjee[4], Anupama Mukherjee[4], Vinod Kumar Yata[5]

[1]College of Veterinary Science, Garividi, Sri Venkateswara Veterinary University, Andhra Pradesh, India.

[2]Livestock Research Station, Garividi, Sri Venkateswara Veterinary University, Andhra Pradesh, India.

[3]Department of Animal Science, Injibara University, Ethiopia

[4]Division of Animal Genetics and Breeding, Indian Council of Agricultural Research-National Dairy Research Institute, Karnal, Haryana, India.

[5]Department of Molecular Biology, Central University of Andhra Pradesh, Ananthapuramu, Andhra Pradesh, India

*Corresponding author

Email: destawworku@gmail.com

¶Authors contributed equally to the work

Abstract:

Natural and artificial selection are fundamental demographic forces that contribute significantly to the shaping of genomes. Exploring the molecular mechanisms implicated in the adaptation processes can assist in creating efficient breeding programs designed to preserve and improve the genetic characteristics of native sheep. The study used a novel method that combines the *p*-values into a single measure to investigate the genomic regions under selection. This method improves the resolution power and prioritises the candidate genes more efficiently than other single statistics. The 50K SNP data on three Indigenous sheep breeds (n=79) were Chantangi (CHA, n= 29), Garole (GAR, n=24), and Deccani (IDC, n=26) native to diverse regions of India and were used in this study. The study combined five statistics into a composite selection signal called a De-Correlated composite of multiple signals (DCMS). The *p*-values of all the single statistics were corrected for multiple testing at the False Discovery Rate (FDR) threshold of < 5%, and the putative genomic regions were detected. Gene and quantitative trait loci (QTL) annotation of the significant regions was performed. The DCMS analysis identified 21, 10, and 14 novel putative genes in the Chanthangi, Garole, and Deccani breeds, respectively. Furthermore, the study also revealed 10, 28, and 13 significant quantitative trait loci regions within the studied breeds. The identified genes and QTLs are related to a wide variety of characteristics, including growth and muscle development (*CNTNAP5, DOCK3*), reproduction (*TCERG1L, BUB1, UNC5C, C2CD5, BBX*), wool trait (*TPPP3, P2RY6, FGF10, POU2F1, FAM168A*), disease resistance

(*MTSS1, B4GALNT3*), environment adaptation (*TRMT12, MAPKAPK3*), domestication (*LRRC36*). The QTLs identified are associated with body conformation (body measurements and bone area), production (milk fat yield), reproduction (total lambs born), disease resistance (hemonchus resistance, foot rot and pneumonia susceptibility), and health (platelet count and entropion). The DCMS method pinpointed novel genomic regions and putative genes more efficiently than other single statistics. It could provide a reliable comprehension of the molecular mechanisms involved in all the essential traits in the Indian sheep.

Keywords: DCMS, Indian Sheep, SNP, QTL, Selection Signature

Introduction:

Natural selection enhances the fitness value of individuals and allows them to reproduce. Hence, the frequency of beneficial mutations increases in the population [1]. Thus, selection pressure on the genome leaves a distinct pattern, and those unique footprints are referred to as selection signatures [2,3]. It provides fundamental insights into the evolutionary forces that alter the genetic makeup of individuals, aids in QTL mapping of quantitative traits in domestic animals, and also reveals beneficial putative mutations with a selection advantage in livestock populations [4]. However, complicated evolutionary processes, including population admixture, inbreeding, population bottlenecks, genetic drift, and mutations, obscure the patterns of selection signatures.

SNP genotyping arrays have revolutionized the study of genomic variations in populations, and nowadays, they are commonly used in genomic studies. These arrays consist of genetically stable single nucleotide polymorphisms distributed across the genome. These SNP arrays provide a deeper understanding of evolution, domestication, breed diversity, the formulation of population theories, the analysis of genetic processes underlying complex traits, and the enhancement of traits through genomic selection. Several methods are described in the literature to identify the signatures of selection based on allele frequency spectrum, linkage disequilibrium, and population differentiation [5,6]. The selection signature analysis previously described has been utilised in various sheep breeds distributed worldwide [7–9]. However, Grossman et al., 2010 proposed a method that combines different signals into a composite of signals (CMS) to enhance the details of the genomic region. This method is believed to be more sensitive and robust than the single selection signal method. The integral haplotype score method yields more reliable signals when the genome undergoes incomplete or ongoing selection. Meanwhile, the XPEHH method efficiently identifies the signals that are almost fixed. It analyzes the genomes of populations to determine the alleles that are becoming fixed in one group but remain variable in others. Hence, most of the studies on farm animals used complementary methods to improve the resolution of signals [5,11].

However, Lotterhos et al., 2017; Ma et al., 2015, proposed an efficient method called a de-correlated composite of multiple signals (DCMS) that combines the results obtained from univariate analysis. The covariance structure among the output signals of

single methods is accounted for to obtain more robust and sensitive signals. DCMS provides accurate signals owing to its better resolution power. The composite signals compute P-values by ranking genomic data, while univariate statistics rely on traditional probability and significance frameworks. Therefore, the DCMS provides advantages over univariate methods due to their lack of standardization, and this method is preferred in the selection signature analysis. SNP arrays are widely used genotyping tools for determining selection signatures in livestock. The OvineSNP50 Bead Chip (Illumina Inc., San Diego, CA) is the most widely used SNP array for studying variations in the sheep genome. This array was developed by incorporating highly polymorphic SNPs from sheep breeds worldwide, with a few Indian sheep breeds also included. [14].

The sheep production systems in India are diverse and include cold arid regions of the Western Himalayas, hot arid regions of Rajasthan and neighbouring states, semi-arid regions of the Deccan Plateau and sub-humid regions of West Bengal and Odisha. According to the 19th Census in India, the sheep population is 74.44 million heads, including 44 registered breeds [15]. The Indian sheep genetic resources comprise diverse morphological, physiological, and behavioral attributes developed over centuries of adaptation to various ecological niches and production systems.

Genomics can aid in identifying variants associated with productivity, disease resistance, and adaptability to diverse environmental conditions, enabling more informed selection decisions. Genomic selection programs can optimise traits such as

growth rate, wool quality, and reproductive efficiency while also helping to preserve the unique characteristics of native sheep. Despite the rich phenotypic diversity observed in Indian sheep, comprehensive genomic studies aimed at uncovering the molecular basis of these traits have been relatively limited. However, few studies on selection signatures in Indian sheep using a single statistic or a combination of univariate statistics have been reported [16,17]. Eydivandi et al., 2021 studied the selection signature analysis in Indian sheep using a combined statistic accounting for the correlations. However, the study did not use the composite signal method to detect the signals within the breed. In this study, the composite signals method (DCMS) was utilized to identify selection signatures in the genomes of three Indian sheep breeds (Chanthangi, Deccani, and Garole), which are adapted to different ecological niches and production systems across India. Additionally, gene annotation and QTL enrichment were performed.

Materials & Methods

Animals and Genotyping

In the present study, the high dense genotypes of three Indian sheep breeds i. e., Changthangi (CHA, n = 29), Deccani (IDC, n = 24), and Garole (GAR, n = 26) genotyped with Illumina Ovine SNP50 BeadChip were obtained from WIDDE (Web-Interfaced next generation Database dedicated to genetic Diversity Exploration) [18]. It is specifically designed to store and manage high-density genotyping datasets pertaining

to bovine and ovine species. The Illumina Ovine SNP50 BeadChip consists of 54,241 SNPs distributed across the sheep genome, with an average density of one SNP every 51 kb. Quality control (QC) of the genotypes was performed based on the following criteria: minor allele frequency (maf < 0.05), Hardy-Weinberg equilibrium (HWE p < 0.0001), individuals with missing genotypes (--mind < 0.1), and SNPs with missing genotype data (geno < 0.1) [19]. These criteria were applied to eliminate low-quality SNPs and advance to subsequent phases of the analysis. A total of 41,997 SNPs were retained for further analysis. The SNPs with unknown coordinates and those on X-chromosomes were excluded, as the study aims to uncover only the genes on autosomes. The SNPs with precise coordinates based on OAR Rambouilliet reference genome assembly were included for analysis.

Ethics Statement:

The research does not involve human or animal participants, and the data examined are publicly available. Therefore, no ethical approval or consent is necessary.

Principal Component Analysis:

The principal component analysis (PCA) was performed on the SNP genotypes of the studied sheep breeds to investigate the structure and clustering of the individuals in the dataset. PCA categorizes individuals into several specific clusters, and an analysis of selection signatures will be conducted on those individuals within each cluster. The PCA analysis was implemented in the R environment using the *snprelate* package [20].

De-Correlated Composite of Multiple Selection Signals

DCMS is a novel combined statistic used to detect selection signals, as it provides more reliable and precise estimates than other univariate statistics. The present study utilized the Composite of Multiple Signals (DCMS) method, as detailed in Yurchenko et al. (2018), combining five univariate statistics: FST, Haplotype Homozygosity (H1), Modified Haplotype Homozygosity (H12), Tajima's D, and Nucleotide Diversity (π) [21–24].

The computation of the DCMS statistic for a locus l is given below

$$DCMS_l = \sum_{t=1}^{n} \frac{\log\left|\frac{1-P_{lt}}{P_{lt}}\right|}{\sum_{i=1}^{n}|r_{it}|} \qquad (1)$$

The DCMS statistic combines the p-values of individual statistics and their correlations to improve the sensitivity and robustness of detecting selection signals. The DCMS statistic is composed of two components. The numerator component includes P_{lt}, which indicates the p-value at a given locus 'l' of each univariate statistic 't'. The denominator contains the correlation parameter 'r_{it}', which accounts for the covariance structure among all the included univariate statistics. It is also described as weighing factors among the statistics that describe the correlations between the statistics [12].

Univariate statistics in DCMS

Fixation Index (F_{ST}):

The F_{ST} index denotes the degree of differentiation between the populations. The functions in *PLINKV1.9*, such as --fst and --within, were used to determine the FST values for each SNP, and the negative F_{ST} values were changed to zeros. Later, the F_{ST} values were smoothed using the *runmed* function in the R program. The F_{ST} values for each breed were estimated.

Haplotype Homozygosity Statistics (H1 and H12):

Haplotype homozygosity assesses the conservation of haplotypes, which can aid in identifying regions subject to selective pressure. The haplotypes in each data set were determined using the SHAPEIT2 version program [25]. The conditional states parameter was kept as the default for the phasing of the haplotypes. The SNePv1.1 program computed the effective population size of each breed, and the estimate for each breed was used in the phasing of the chromosomes [26]. A high-resolution Ovine recombination map was used to correct the variations due to the recombination rates at all the autosomes [27]. An R script was used to convert the phased haplotypes to the input files as required in the H12_H1H2.py program. The window size of SNPs was kept to 14, and the step size was 1 (-window 14 -jump 1) [24].

Tajima's D and Nucleotide Diversity (π)

Tajima's D detects departures from neutrality, with negative values indicating potential selective sweeps, while nucleotide diversity (π) measures the genetic variation within a population. The statistics Tajima's D and (π) at each coordinate in the dataset of all the studied breeds were computed using the vcftools program [28], and the estimates were

computed for each chromosome separately. The nonoverlapping sliding window of 300 MB (-Tajima 300) was used to compute the Tajima's D index. Further, the *p-values* were allotted to the SNPs present in the bin. Meanwhile, the –site-pi function in the vcftools program is employed to compute the π values of each SNP. The runmed function smooths the *p*-values as outlined in [29].

DCMS Estimation

The stat_to_p-value function in the MINOTAUR package was implemented based on the five statistics, depending on the functional ranks [30]. All five statistic parameters (H1, H12, Tajima's D index, π, and FST) for each SNP were combined into a new composite signal as DCMS. The covNAMcd function of the rrcovNA R package (alpha = 0.75, nsamp = 50,000) was utilised to compute a correlation matrix of n x n order [31]. The correlation matrix is useful for accounting for the correlations among the univariate statistics, which combine to produce a composite statistic. The output thus obtained was given as input for DCMS estimation, and the DCMS values were normalised using the rlm function of the MASS package in the R environment [32] as described in Yurchenko et al. (2018). The [33]A correction that reduces the error rate from multiple tests was applied to q-values transformed from p-values [34].

Identification of Functional Genes and QTL regions

The putative genomic regions were determined based on the q-value. A q-value threshold 0.05 was chosen to identify genomic regions with strong evidence of selection, while adjacent SNPs with q-values greater than 0.1 were excluded to reduce false

positives. The gene and QTL annotation of the candidate genomic regions was carried out using the Genomic Annotation in Livestock for positional candidate Loci (GALLO) package v4.2.0 [35]. The '.gtf' file related to ARS-UI_Ramb_v2.0 of the sheep genome was accessed from NCBI, and the corresponding '.gff' belonged to the ARS-UI_Ramb_v2.0 was downloaded from the Sheep QTLdatabase [36].

The candidate genes and quantitative trait loci (QTLs) located within the significant genomic regions are considered to be putative if they are distributed within an interval of 0.5 Mb upstream and downstream. Further, QTL enrichment analysis was performed to identify overrepresented traits within the significant regions, using a genome-wide approach and a false discovery rate (FDR) threshold of 0.05.

Results:

Quality Control of genotypic data:

The quality control of genotypic data included the filtering of SNPs with minor allele frequency (maf<0.05), Hardy Weinberg equilibrium of the SNPs (hwe<0.0001), missingness thresholds for SNP genotypes (geno <0.1) and individual genotypes (mind <0.1. The SNPs excluded for maf, hwe, geno and mind were 3845, 299, 678 and 0, respectively. Further, the SNPs with unknown coordinates (n=1232) on the chromosomes other than the autosomes were removed. The final data set contains 41997 variants used for further downstream analysis.

Principal Component Analysis (PCA)

The principal component analysis (PCA) was employed to ascertain the distinct clusters of SNP genotypes for the three Indian sheep breeds under investigation. The results of PCA are presented in Fig 1. The PCA confirmed the genetic differentiation among the three breeds, as indicated by their clear separation into distinct clusters. PC1 accounted for 14.34% of the variation, highlighting the strongest genetic differences between the populations, while PC2 explained an additional 7.98% of the total variation, capturing finer distinctions.

De-Correlated Composite of Multiple Selection Signals (DCMS)

Chanthangi sheep:

Gene Annotation:

The DCMS analysis revealed twenty-one putative regions in the Chanthangi sheep genome. The total size of the genome under selection was 2871.54 Kb. The mean genome length under selection was 136.74±229.77 Kb, ranging from 1.08 to 1.83 Kb (Table 1).

Table 1. shows the genomic regions of Indian sheep breeds identified as being under selection through DCMS analysis

S.No.	Breed	Total Size (kb)	Mean ± Standard Deviation (Kb)	Maximum Length (kb)	Minimum Length (kb)	Total No. of genes
01	Chanthangi (CHA)	2871.54	136.74±229.77	1080.32	1.83	21
02	Garole (GAR)	2857.84	259.80±310.04	1080.32	34.11	10
03	Deccani (IDC)	2513.30	167.55±216.86 Kb	817.32	2.20	14

These genes were located on BTA 2, 3, 9, 14, 15, and 26. A greater number of significant genomic regions were identified on BTA 15 (Table 2).

Table 2. shows the results of gene annotation in significant genomic regions in CHANTHANGI sheep

CHR*	BP*	START COORDINATE	END COORDINATE	Width	Strand	Gene Name	*Pvalue*	Qvalue
2	190267950	189526649	190606970	1080322	+	*CNTNAP5*	*4.95e-05*	*0.032*
3	106221882	106222211	106279403	57193	+	*FBLN7*	*1.29e-05*	*0.015*
3	107958569	107917450	107989336	71887	+	*TBC1D15*	*1.28e-05*	*0.015*
9	28518494	28426199	28586681	160483	+	*MTSS1*	*0.008*	*0.048*
9	28610511	28596736	28628449	31714	+	*TATDN1*	*9.16e-05*	*0.045*
9	28651053	28650081	28651912	1832	-	*TRMT12*	*0.000*	*0.048*
9	28651053	28627373	28650325	22953	-	*RNF139*	*0.000*	*0.048*
14	35090205	35080140	35173351	93212	+	*NFATC3*	*4.59e-06*	*0.009*
14	34566935	34510632	34569231	58600	-	*TPPP3*	*7.71e-05*	*0.042*
14	34566935	34504860	34579222	74363	+	*LRRC36*	*7.71e-05*	*0.042*
15	51070453	51028830	51075998	47169	-	*STARD10*	*2.34e-08*	*0.000*
15	51107183	51099187	51353916	254730	-	*FCHSD2*	*2.34e-08*	*0.000*
15	51474825	51404925	51511918	106994	+	*P2RY2*	*1.39e-08*	*0.000*
15	51517148	51481919	51516379	34461	+	*P2RY6*	*1.39e-08*	*0.000*
15	51578641	51521915	51579398	57484	+	*ARHGEF17*	*6.12e-10*	*2.57e-05*
15	51591704	51585751	51604071	18321	+	*RELT*	*3.07e-09*	*6.44e-05*
15	51632422	51613275	51814944	201670	-	*FAM168A*	*1.19e-05*	*0.015*
16	30667706	30604422	30702581	98160	+	*FGF10*	*7.11e-06*	*0.010*
16	31588015	31575745	31651791	76047	-	*NIM1K*	*4.12e-05*	*0.031*
16	31687647	31660770	31688311	27542	-	*ZNF131*	*1.85e-05*	*0.018*
16	32196927	32069778	32366181	296404	-	*GHR*	*9.62e-07*	*0.003*

*CHR – Chromosome; *BP – Base Position

The gene annotation of the significant genomic regions uncovered twenty-one potential candidate genes related to a variety of traits in this sheep, including *CNTNAP5* (body growth), *MTSS1* (host resistance to bacterial infections), *TRMT12* (immune system and environment adaptation), *TPPP3* (hair follicle growth), *LRRC36* (domestication and early development), *STARD10* (resilience to paratuberculosis), *P2RY6*, *FGF10* and

FAM168A (hair follicle morphogenesis), *ARHGEF17* and *NIM1K* (meat quality), *GHR* (litter size).

Quantitative Trait Loci (QTL) Annotation and Enrichment:

Table 3 presents the results of QTL annotation in Chanthangi sheep. Our study revealed the significant genomic regions annotated with ten QTLs on OAR 2, 3, 14, 15, and 16.

Table 3. shows the Quantitative Trait Loci annotation results in significant genomic regions in CHANTHANGI sheep.

CHR*	BP*	START COORDINATE	END COORDINATE	Name	Flank Marker	P-value
2	190267950	190457084	190457088	Body weight	rs424714738	1.61E-03
3	107991182	108401221	108401225	Body weight	rs426980328	0.026
14	35090205	35417680	35417684	Body weight	rs417231209	<0.05
15	51070453	50955799	50955803	Milk fat yield	rs404288918	0.0006245
15	51474825	51832265	51832269	Platelet count	rs413264572	5.50E-06
16	31588015	32070073	32070077	Total lambs born	rs161146164	<0.05
16	31654423	32118461	32118465	Total lambs born	rs426666828	<0.05
16	32569758	32119256	32119260	Milk fat yield	rs422431427	0.00031338
16	32569758	32192693	32192697	Body weight	rs408124092	<0.0001
16	32569758	32192693	32192697	Soft tissue depth at GR site	rs408124092	0.0014

*CHR – Chromosome; *BP – Base Position

The annotated traits are known to be associated with body weight, milk fat yield, platelet count, total lambs born, and soft tissue depth at the GR site. The QTLs identified in each breed were categorized into various trait classes, including body weight, reproduction, milk yield, and health, and the enrichment of QTLs is shown in Fig 2. The highest per cent of QTLs annotated in the significant regions of Chanthangi were milk (37.5%), followed by reproduction (34.38%). The other classes of QTLs annotated were meat and carcass (9.38%), health (9.38%) and production (9.38%). The

QTL enrichment analysis revealed the top significant (False Discovery Rate (FDR)-corrected p-value - 0.05) QTL total lambs born on OAR 16.

Garole sheep:

Gene Annotation:

The DCMS analysis found eleven (11 No's) potential regions in the Garole sheep genome covering the overall size of the genome, which was 2857.84 Kb. The average size of the genome under selection was found to be 259.80±310.04 Kb (Table 1). The maximum and minimum size of the genome ranged between 1080.32 and 34.11 Kb. Several putative genes were harboured in this study's significant genomic regions and were situated on OAR 1, 2, 3, 6, 16 and 18 (Table 4).

Table 4. shows the gene annotation results in significant genomic regions in GAROLE sheep.

CHR*	BP*	START COORDINATE	END COORDINATE	Width	Strand	Gene	*Pvalue*	*Qvalue*
1	173424721	173196985	173483458	286474	+	*BBX*	*2.01e-05*	*0.015*
2	189539157	189526649	190606970	1080322	+	*CNTNAP5*	*8.19e-05*	*0.042*
3	193287569	193251622	193339539	87918	+	*C2CD5*	*8.81e-07*	*0.003*
3	212967581	212887405	212973893	86489	+	*IQSEC3*	*6.47e-06*	*0.007*
3	213086338	213068112	213128627	60516	-	*KDM5A*	*1.07e-05*	*0.009*
3	213195386	213175874	213273093	97220	+	*B4GALNT3*	*7.64e-06*	*0.007*
6	29809690	29597358	30017600	420243	+	*UNC5C*	*6.21e-06*	*0.007*
16	65681259	65471620	65927572	455953	-	*ADCY2*	*7.56e-05*	*0.041*
18	46100361	46097255	46131370	34116	-	*CLEC14A*	*4.00e-05*	*0.024*
22	49602008	49599602	49793506	193905	-	*TCERG1L*	*6.19e-06*	*0.007*

*CHR – Chromosome; *BP – Base Position

However, OAR 3 anchors more potential candidate genes in the GAR breed. In this study, we identified eleven putative genes associated with a wide range of

characteristics, including *BBX* (teat number), *CNTNAP5* (body growth), *C2CD5* (fecundity), *KDM5A* (follicular development), *B4GALNT3* (paratuberculosis resistance), *UNC5C* (litter size), *ADCY2* and *TCERG1L* (reproduction).

Quantitative Trait Loci (QTL) Annotation and Enrichment:

The present study unveils the findings of QTL annotation in the GAR breed, shown in Table 5.

Table 5. shows the Quantitative Trait Loci annotation results in significant genomic regions in GAROLE sheep.

CHR*	BP*	START COORDINATE	END COORDINATE	Name	Flank Marker	P-value
1	173424721	173432597	173432601	Teat number	rs430157497	<0.05
1	173424721	173534798	173534802	Teat number	rs426598066	<0.05
2	190019411	190457084	190457088	Body weight	rs424714738	1.61E-3
3	192453314	192442586	192442590	Entropion	rs411640492	3.19E-04
3	192935483	192442586	192442590	Entropion	rs411640492	3.19E-04
3	193336800	193016493	193016497	Entropion	rs404605277	1.12E-04
3	212967581	213021781	213021785	Entropion	rs159913124	3.61E-04
3	213209196	213213768	213213772	Entropion	rs418752307	2.81E-04
6	29809690	29344134	29344138	Bone area	rs418089550	1.12E-5
6	29809690	29366036	29366040	Milk fat yield	rs424558688	0.036982472
6	29847992	30096782	30096786	Total lambs born	rs421635584	<0.05
18	46159259	46086980	46086984	Body weight	rs426187704	<0.05
18	46100361	46086980	46086984	Body depth	rs426187704	<0.01
18	46062053	46086980	46086984	Body weight	rs426187704	<0.05
18	46062053	46086980	46086984	Body length	rs426187704	<0.05
18	46159259	46086980	46086984	Body circumference	rs426187704	<0.05
18	46159259	46086980	46086984	Body depth	rs426187704	<0.01
18	46159259	46086980	46086984	Rump width	rs426187704	<0.05
18	46159259	46087004	46087008	Body weight	rs404696179	<0.05
18	46159259	46087004	46087008	Body length	rs404696179	<0.01
18	46100361	46087004	46087008	Body height	rs404696179	<0.01
18	46159259	46087004	46087008	Body circumference	rs404696179	<0.05

18	46062053	46087004	46087008	Body height	rs404696179	<0.05
18	46159259	46087646	46087650	Chest width	rs405457403	<0.05
18	46159259	46087646	46087650	Shin circumference	rs405457403	<0.05
18	46159259	46087646	46087650	Body depth	rs405457403	<0.05
18	47232764	47716852	47716856	Haemonchus contortus resistance	rs428856771	3.23E-4
22	49796078	49873766	49873770	Lambs born alive	rs402605786	9.79E-3

*CHR – Chromosome; *BP – Base Position

Our study revealed the significant genomic regions annotated with twenty-eight QTLs on OAR 1, 2, 3, 6, 18 and 22. The annotated traits are known to be associated with teat number, body weight, milk fat yield, entropion, bone area, total lambs born, body depth, body length, body circumference, shin circumference, body weight, body height, chest width, rump width, lambs born alive and hemonchus contortus resistance. Furthermore, the annotated QTLs are categorised into different classes, and the enrichment of QTLs is depicted in Fig 2A. Garole's most significant genomic regions had the greater proportion of QTLs associated with health (38.74%) and production (38.74%). The remaining per cent of QTL classes annotated in the breed were reproduction (11.71%), exterior (8.11%), milk (1.8%) and meat and carcass (0.9%). An analysis of QTL enrichment disclosed the three most remarkable QTLs (False Discovery Rate (FDR)-corrected p-value - 0.05) associated with the total lambs born, teat number and entropion on OAR 6, 1 and 3.

Deccani sheep:

Gene Annotation:

The analysis carried out by the DCMS has uncovered fourteen putative regions in the Deccani sheep genome. The genomic regions under selection ranged in size from 2.20 to 817.32 Kb, with an average length of 167.55 ± 216.86 Kb (Table 1). The findings of gene annotation are summarised in Table 6.

Table 6. shows the gene annotation results in significant genomic regions in DECCANI sheep.

S.No	CHR*	BP*	START COORDINATE	END COORDINATE	width	strand	Gene	*Pvalue*	Qvalue
1	1	119053600	119027142	119081828	54687	+	*MAEL*	*9.08e-09*	*4.24e-05*
2	1	131092601	130838877	131151750	312874	+	*APP*	*3.55e-07*	*0.000*
3	1	119491972	119428361	119624526	196166	+	*POU2F1*	*1.33e-05*	*0.007*
4	3	104706333	104671436	104716760	45325	-	*MALL*	*9.21e-13*	*1.29e-08*
5	3	104775041	104716342	104784266	67925	-	*NPHP1*	*9.21e-13*	*1.29e-08*
6	3	104885280	104870169	104915494	45326	-	*BUB1*	*1.00e-13*	*4.20e-09*
7	3	212967581	212887405	212973893	86489	+	*IQSEC3*	*0.000*	*0.044*
8	3	104917562	104914948	105286674	371727	+	*ACOXL*	*1.64e-11*	*1.15e-07*
9	4	101967194	101379345	102196659	817315	+	*CHRM2*	*1.64e-11*	*1.15e-07*
10	19	49657211	49447036	49738575	291540	-	*DOCK3*	*6.78e-06*	*0.005*
11	19	49778830	49759229	49789524	30296	-	*MAPKAPK3*	*5.45e-06*	*0.004*
12	2	52731947	52731991	52734550	2560	+	*HINT2*	*5.26e-07*	*0.001*
13	2	52790093	52790313	52801134	10822	-	*RGP1*	*5.26e-07 0.001*	*0.001*
14	2	52790093	52790904	52793100	2197	+	*MSMP*	*5.26e-07 0.001*	*0.001*

*CHR – Chromosome; *BP – Base Position

The gene annotation of the significant genomic regions revealed fourteen potential candidate genes related to a variety of traits in this sheep, including *BUB1* (oocyte and follicle development), *POU2F1* (wool colour), *CHRM2* (hair follicle growth), *DOCK3* (muscle development), *MAPKAPK3* (heat stress response). These genomic regions dock potential candidate genes associated with various traits and are located on OAR 1, 2, 3, 4 and 19, with a maximum number of significant genomic regions on BTA 19.

Quantitative Trait Loci (QTL) Annotation and Enrichment:

The findings outlined in Table 7 detail the results of QTL annotation in Deccani sheep.

Table 7. shows the Quantitative Trait Loci annotation results in significant genomic regions in DECCANI sheep.

CHR*	BP*	start_pos	end_pos	Name	FlankMarker	P-value
1	131092601	130616460	130616464	somatic cell count	rs409795528	<0.05
3	212967581	213021781	213021785	Entropion	rs159913124	3.61E-04
3	212967581	213213768	213213772	Entropion	rs418752307	2.81E-04
4	101967194	101960285	101960289	Monocyte number	rs399619443	2.55E-6
4	101967194	102026928	102026932	Footrot susceptibility	rs416121047	2.84E-6
4	101967194	102039511	102039515	Pneumonia susceptibility	rs403394816	<0.10
21	41665172	41243033	41243037	Body length	rs406947061	<0.05
2	52731947	52720586	52720590	Body weight	rs426272889	<0.05
2	52790093	52737532	52737536	Body weight	rs160159557	<0.05
2	52790093	52848103	52848107	Water holding capacity	rs420647640	<0.05
2	116594826	116133033	116133037	Horn type	rs416045857	<0.05
2	116594826	116839657	116839661	Horn type	rs405044765	<0.05
2	116953479	116872298	116872302	Horn type	rs402400571	<0.05

*CHR – Chromosome; *BP – Base Position

The study has identified significant genomic regions annotated with thirteen QTLs on OAR 1, 2, 3, 4 and 21. The QTLs are linked to the traits viz., somatic cell count,

Entropion, Monocyte number, Footrot susceptibility, Pneumonia susceptibility, Body length, Body weight, Water holding capacity, and horn type. Furthermore, the annotated QTLs are sorted into various categories, and the distribution of QTLs is illustrated in Fig 2. The regions with the most significant impact on the evolvement of the Deccani breed had the highest percentage of QTLs annotated for exterior (48.15%), followed by health (25.93%). The other classes of QTLs annotated were production (18.52%), and meat and carcass (7.41%). The QTL enrichment analysis unveiled the top most significant QTLs (False Discovery Rate (FDR)-corrected p-value - 0.05) on OAR 1,2,3 controlling traits like somatic cell count, horn type, Entropion, Body weight, and Body length (Fig 3). Further, the signatures of selection in the genome of the studied breeds are depicted in Fig 4.

In addition, the summary of putative genes is presented in the Table 8.

Table 8. Summary of putative genes identified in Indian sheep

CHR*	BP*	START COORDINATE	END COORDINATE	Gene	Trait
2	189539157	189526649	190606970	*CNTNAP5*	Growth and muscle development
3	193287569	193251622	193339539	*C2CD5*	Reproduction
9	28518494	28426199	28586681	*MTSS1*	Disease resistance
3	213195386	213175874	213273093	*B4GALNT3*	Disease resistance
9	28651053	28650081	28651912	*TRMT12*	Adaptation
3	104885280	104870169	104915494	*BUB1*	Reproduction
6	29809690	29597358	30017600	*UNC5C*	Reproduction
14	34566935	34504860	34579222	*LRRC36*	Domestication
19	49657211	49447036	49738575	*DOCK3*	Growth and muscle development
19	49778830	49759229	49789524	*MAPKAPK3*	Adaptation
15	51517148	51481919	51516379	*P2RY6*	Wool trait
16	30667706	30604422	30702581	*FGF10*	Wool trait

Discussion:

This study unveiled comprehensive information about the genomic regions under selection in three Indian sheep breeds adapted to the country's highly harsh climatic conditions. The DCMS analysis uncovered the potential candidate genes linked to various traits of economic importance in sheep. The complex and obscured evolutionary histories of livestock species indicate that a single univariate statistic may not adequately capture the full selection signals present in their genomes. The outcome of the univariate genome scan methods is mostly biased as they are greatly affected by the outliers, which determine the selection signals. Further, the application of background selection to neutral loci linked to recessive alleles could reveal biases in demographic inference. If a species exhibits widespread linkage disequilibrium, the impact of non-independence among loci on statistical outcomes remains uncertain, particularly when selection is present [13]. Keeping this in view, the present investigation used a novel statistic that combines *p*-values obtained from five different univariate statistics into a composite signal that accounts for the correlation among all univariate statistics. Few studies have used multiple complementary statistics to analyze selection signatures in these breeds, and many do not account for correlations between these statistics [17,37]. However, a recent study explored the genomic regions of Indian sheep using four complementary statistics accounting for their absolute correlations between the statistics [38]. This study mainly compared selection signatures in Iranian breeds with a comparison note on Indian and Afghani breeds. This study combined five univariate statistics, such as H1, H12, F_{ST}, TajimaD and Nucleotide

diversity, into a composite signal that accounts for correlations between them. It is well documented in the literature that the DCMS method provides absolute and accurate information compared to the results obtained from univariate statistics [10,12,13]. Hence, this study used composite signals to exhaustively study the signatures of the within-breed selections in Indian sheep breeds. As a result, the selection signatures analysis unveiled the potential candidate genes and quantitative traits in the genomes of the studied breeds.

Principal Component Analysis (PCA)

Principal Component Analysis (PCA) is a fundamental underpinning for exploratory data analysis. In short, the PCA computes the eigenvectors from the covariance matrix of the markers genotyped in the samples. Subsequently, the genotypic coefficients of the principal components will be visualized through plotting to illustrate the clustering resulting from population stratification. Thus, the dimensionality of the huge genotype matrix is reduced to principal components. The distinctive clustering of breeds into three categories, as depicted in the PC plot presented here, was not entirely consistent with the findings of Ahmad et al., 2021. The researchers could not differentiate three sheep breeds into separate clusters based on the first principal component. This could be due to the inclusion of a more significant number of other breeds, representing an additional variation source. Regional climatic conditions and environmental factors predominantly influence distribution patterns of livestock. These factors encompass temperature ranges, precipitation patterns, topography, and vegetation types, all of

which play critical roles in shaping the sustainable demographics of livestock. The study's findings indicated that the breeds evolved into unique and more specialised breeds owing to the prevailing local climate conditions. This research identified several candidate genes and genomic regions linked to local adaptation in sheep, highlighting essential QTLs associated with domestication, environmental adaptation, heat and cold tolerance, and resistance to bacterial diseases. In summary, the prevailing climatic and ecological conditions have facilitated the adaptation of the examined sheep breeds, each exhibiting specialized traits suited to their environments. The Garole breed, known for its high fecundity, thrives in the hot and humid conditions of West Bengal. In contrast, the Chanthangi breed, characterized by its wool quality, is predominantly found in the colder regions of the Himalayas. Similarly, the Deccani breed has adapted effectively to the arid environments of central India, showcasing its resilience to dry conditions (Nbagr.res.in).

Chanthangi sheep:

Gene Annotation:

The sheep production system in India spans a cold climate in the foothills of the Northern Himalayas to a hot, arid climate in Central Peninsular India and hot and humid regions of Eastern India. Based on our findings, none of the identified genes within the detected regions demonstrated significance across all three analysed breeds. However, the potential genomic regions docked the genes linked to body growth, reproduction, fecundity, heat stress response, host resistance to bacterial infections,

domestication, environmental adaptation, hair follicle growth, meat quality, and litter size. The study revealed minimal overlap of the genomic regions among the breeds, suggesting breed-specific selection processes in the past.

~~The significant genomic regions of the Chanthangi breed consist of the genes *CNTNAP5* (body growth), *MTSS1* (host resistance to bacterial infections), *TRMT12* (immune system and environment adaptation), *TPPP3* (reproduction), *LRRC36* (domestication and early development), *STARD10* (resilience to paratuberculosis), *P2RY6* and *FGF10* (hair follicle morphogenesis), *ARHGEF17* and *NIM1K* (meat quality), and *GHR* (litter size).~~

The present study found the *CNTNAP5* (Contactin-associated protein-like 5B) in the significant genomic regions harboured on OAR2, a protein-coding gene. This gene belongs to the neurexin family and plays a significant role as a cell adhesion molecule and receptor in the nervous system of most vertebrates. The mechanism of action of *CNTNAP5* is comparable with the brain-derived neurotrophic factor (*BDNF*) gene, which affects the body mass index. Like other epidermal growth factors, it potentially affects growth by enhancing the functions of the brain that have a role in growth regulation [39,40]. The genomic investigation of the morphological traits of Sudanese goats revealed that the *CNTNAP5* gene located on chromosome 2 is associated with the bicoastal diameter [41].

The indigenous sheep of India are known for their resistance to infectious agents. Our findings also identified specific genomic regions in Chanthangi sheep that harbour the *MTSS1* gene on chromosome OAR9 related to resistance to bacterial infections. These

findings are consistent with the breed characteristics of Chanthangi. The bacteria's endotoxins disrupt the endothelial lining in the host cell and cause infection. However, the *MTSS1* gene alters the host endothelium and improves its integrity to avoid infections [42]. The DCMS analysis also revealed significant genomic regions that anchor the *STARD10* gene, reportedly involved in the resistance to paratuberculosis [43]. It is involved in regulating bile acid metabolism by modulating the PPARα-mediated pathway [44]. The study found two essential candidate genes, *TRMT12* and *LRRC36*, associated with environmental adaptation and domestication in sheep. The study on the structural variant landscape in sheep and goats revealed that the *LRRC36* gene was involved in the early domestication of sheep and goats [45]. The *TRMT12* gene facilitates the codon-interactions while de-coding at the ribosomes, whereas, *LRRC36* is a leucine rich repeat containing 36 gene differentially expressed in testis and lung tissues.

Chanthangi sheep are reared mainly by Changpa, a nomadic tribe of the Northern hills, along with the Chanthangi goats. This dual-purpose sheep breed, maintained for wool and mutton, contributes significantly to the livelihood of the nomadic tribes. The income of the shepherds mainly was deployed from selling wool and meat. Interestingly, our study highlighted the critical candidate genes associated with hair follicle development, such as *P2RY6* (OAR 15) and *FGF10* (OAR 16). A gene expression study in mice uncovered the role of *P2RY6* in controlling the MEK1/2–b-catenin signalling pathways and MST–LATS1–YAP signal cascades. It indicated the gene function in the growth and development of keratinocytes [46]. Moreover, gene

expression investigation on the hair follicles of coarse sheep fetal skin revealed that the *FGF10* gene is involved in the hair follicle development by the process of competitive adsorption of miR-184 that produces *FGF10* from chi-circRNA-0001141 [47].

We also identified genes under selection in Chanthangi sheep, including *ARHGEF17* and *NIM1K,* which are known to be related to meat quality. Weighted single-step genome-wide association study (GWAS) findings have revealed a significant association between the *ARHGEF17* gene and meat traits within the Chinese yellow-feathered chicken population [48]. Further, Whereas, *ARHGEF17* is a key component of actin cytoskeleton organization and it enhances the guanyl-nucleotide exchange factor activity. Transcriptomic and epigenomic studies in the pectoral muscles in chicken embryos that belong to pure lines revealed the significant role of the *NIM1K* gene in muscle fibre formation, *NIM1K* gene Enables ATP binding activity; magnesium ion binding activity; and protein serine/threonine kinase activity and involved in protein phosphorylation [49].

Quantitative Trait Loci (QTL) Annotation and Enrichment:

The DCMS analysis revealed important QTL regions associated with body weight on OAR 2, 3, 14 and 16. The flanking markers or SNPs in the QTL regions, including rs424714738, rs426980328, rs417231209 and rs408124092, respectively. Interestingly, A genome-wide association study in Australian Merino sheep identified a significant QTL region on OAR2 associated with body weight. The study also found an orthologous region on OAR6, similar to human and bovine genomic regions associated

with height and weight [50]. Our study also found the same QTL as significant regions in the studied breeds. In a study, Zhang et al., 2016 identified the *MEF2B* and *TRHDE* gene polymorphisms linked to body weight at four months of age in New Ujumqin Sheep Populations located on OAR 3 [51]. The DCMS analysis revealed the same QTL region under selection in the Indian sheep populations. In Baluchi sheep, the genome-wide association study confirmed the location of the QTL peak associated with 8-month body weight [52]. Interestingly, we found the QTL on OAR 14 in the Chanthangi sheep. Likewise, Armstrong et al., (2018) investigated the genetic polymorphisms in the *GHR* gene and revealed their association with carcass traits in grazing Texel sheep. Interestingly, the same QTL peak was observed in the Chanthangi sheep.

We also identified QTL related to platelet count and milk yield on OAR15 in Chanthangi sheep. Interestingly, Gonzalez et al., (2013) reported the QTL association with mean corpuscular haemoglobin concentration (MCHC, $P = 6.2 \times 10-14$) and decreased mean corpuscular volume in Columbia, Polypay, and Ramboulliet sheep. The DCMS analyses also identified the QTL related to the platelet count in the significant genomic regions of Chanthangi sheep. Using three statistical models, the genomic investigation in the crossbred dairy sheep population revealed the QTL regions with the milk yield [55]. We also found the QTL region in the Chanthangi sheep population. We also found the same QTLs annotated in the significant genomic regions of Chanthangi.

Garole sheep:

The Garole sheep is renowned for its petite stature, remarkable fertility, better meat quality and ability to thrive in the saline marshlands of the hot and humid Sundarban region in West Bengal. This breed is also distributed across the southern regions of Bangladesh. Its small size is a result of its adaptation to the harsh climate. Although the genetic potential is relatively limited, these sheep could be raised within a management system requiring minimal inputs (Pan & Sahoo, 2008). This particular breed is highly esteemed due to its fecundity-related genes.

Gene Annotation:

The gene annotation results revealed a few candidate genes associated with essential traits in Garole sheep. We identified the *BBX* gene on OAR 1, which is involved in the supernumerary teats in sheep. Interestingly, a genome-wide association study in Wadi sheep of China reported the candidate genes linked to the supernumerary nipple phenotype in sheep [56]. This study unveiled a few candidate genes related to the trait, including the *BBX* gene. The *BBX* gene is involved in processes such as cell proliferation and breast tumors. It produces a high mobility group domain (HMG) transcription factor that can be seen in breast tumors [57]. The DCMS analysis revealed *the CNTNAP5* gene on OAR 2, which regulates the body growth in sheep, and this significant region was also annotated in the Chanthangi breed [41]. The same genomic region is similarly subject to selection in the Chanthangi breed, suggesting that the variants within this gene are undergoing selective pressure and have demonstrated influence on body growth in both breeds.

In this region, we identified a few candidate genes on OAR 3 in GAR sheep, including the *C2CD5, IQSEC3, KDM5A*, and *B4GALNT3* genes. An expression study in the sheep on oviductal mRNAs and lncRNAs in the follicular and luteal phase uncovered the C2CD5 gene [58]. Our study also identified the C2CD5 gene, which is involved in calcium-dependent phospholipid binding activity and regulates the insulin receptor signalling pathway. This is one of the interesting genes under selection linked with prolificacy and increased litter size in sheep, which is aligned with the demographic history of this sheep breed. Another gene, *KDM5A,* plays a significant role in the zygote genome activation in the cloned embryos in goats [59]. The DCMS analysis also pinpointed the *KDM5A* gene in our study of GAR sheep. We identified another promising candidate gene, *UNC5C,* under selection in GAR sheep. A genome-wide study in Hu sheep confirmed the role of the *UNC5C* gene in litter size in Hu sheep along with the *BMPR1B* gene [60]. According to Sugimoto et al., the *UNC5C* is a protein-coding gene that is a part of the UNC-5 family of netrin receptors and is associated with the conception rate in cattle [61]. Several whole genome studies confirmed the gene role in litter size in Hu sheep [62–64].

Another gene of interest in this study is the *ADCY2* gene, known for its pleiotropic effects. It produces cAMP, a signalling molecule in response to G protein signal transduction. This ultimately leads to increased IL-6 formation, a significant change in Brucellosis [65,66]. This gene also plays a significant role in reproduction, as it enhances the expression of estradiol [67]. Interestingly, we identified this gene in our study.

Quantitative Trait Loci (QTL) Annotation and Enrichment:

The DCMS analysis identified several QTL regions associated with teat number, body weight, body measurements, entropion, resistance to the Hemonchus infection, total lambs born, and total lambs alive.

Our study identified the peak QTL region at 201.6 (cM); the markers were rs430157497 and rs426598066. This genomic region on OAR1 surrounding the functional candidate genes, viz., BBX and CD47, was associated with supernumerary teats in the Wadi sheep of China [56]. One of the significant genomic regions on OAR6 is associated with bone area in Garole sheep. Previous research has identified a genomic region containing putative genes influencing bone density and area in Scottish Blackface lambs through a Genome-Wide Association Study (GWAS) [68].

The Garole sheep is known for its prolificacy and is much valued among breeders owing to its litter size. A few studies confirmed the genomic regions on OAR 6 and 22 that were potentially related to litter size, especially in the Wadi, Hu, Icelandic, Finnsheep, Romanov, and Lori-Bakhtiari sheep breeds [69,70]. The genes in the above potential region, including *BMPR1B*, *CTNNB1*, and *LHCGR*, are known to significantly affect litter size in some sheep breeds. Remarkably, the QTL associated with the litter size is consistent with the breeding history of the Garole breed.

The study on Somatostatin Receptor Subtype 1 (SSTR1) Gene Polymorphism and its relation with growth traits in the Hulun Buir Sheep and confirmed their significant association polymorphism [71]. Interestingly, we observed more QTLs on OAR18

associated with various body measurements such as body weight, length, circumference, rump width, body depth, chest width and shin circumference.

Deccani sheep:

Gene Annotation:

The Deccani breed represents the sole pure black coarse wool breed indigenous to India**.** Our study observed a significant genomic region harbouring a few candidate genes, such as MAEL, APP, and *POU2F1*. The *POU2F1* gene controls melanin production by binding with SLC7A11 and preventing its function [72]. This gene also affects the role of other candidate genes, including SLC7A11, MITF, SLC24A5, MC1R, and ASIP, and thus impacts melanin production and wool colour [73]. The results support the unique traits of the Deccani sheep breed.

Another remarkable region on OAR3 identified in this study consists of several significant genes viz., *MALL, NPHP1, BUB1, IQSEC3* and *ACOXL*. The *MALL* and *NPHP1* genes operate cell division and facilitate cell-matrix adhesion signalling. The *BUB1* gene encodes a protein that plays a role in DNA damage response. *IQSEC3* gene controls the small GTPase mediated signal transduction and *ACOXL* is associated with fatty acid beta-oxidation using acyl-CoA oxidase. Previous genomic studies on these breeds also confirmed the role of this region in conferring the immune response to several factors [37]. Surprisingly, our study also identified the genomic region.

We detected another signature genomic region on OAR2, which comprises the genes controlling sheep growth and fat deposition, including *HINT2, RGP1*, and *MSMP*. GWAS in Indigenous sheep of Ethiopia revealed a strong association of these genes with fat deposition [74] and growth traits. The HINT2 gene is involved in growth and fat deposition by facilitating the hydrolysis of various bonds, such as C-O, C-N, and C-C. Meanwhile, the RGP1 gene promotes the retrograde movement of membrane-bound vesicles to the trans-Golgi network, and the MSMP gene secretes a member of the beta-microseminoprotein family. A few other studies have reported the association of these genes with fat deposition and growth in sheep. [75,76].

In the present study, we identified another significant genomic portion of OAR14 associated with muscle development in cattle. *DOCK3* is a functional candidate gene in the genomic signature region. The molecular study in Bashbay sheep pinpointed the significant role of the *DOCK3* gene in the significant development of skeletal muscle in the specific breed (Zhang et al., 2021). The DOCK gene family is an 11-member class of guanine nucleotide exchange factors that play a crucial role in myoblast fusion and migration across various tissues. Additionally, these proteins are prominently expressed within the skeletal muscle system by stimulating the RAC1 and WAVE signalling pathways in skeletal muscle [77].

Quantitative Trait Loci (QTL) Annotation and Enrichment:

The QTL analysis uncovered a few putative signature genomic regions under selection, including those QTLs controlling traits such as somatic cell count, entropion, foot rot

and pneumonia susceptibility, monocyte number, body weight, body length, water holding capacity and horn length.

The DCMS analyses identified a significant putative genomic region associated with somatic cell count in this sheep. In a GWAS study, significant associations between SNPs and milk production, composition, coagulation, and cheese traits in Assaf and Churra dairy sheep were reported in the literature; somatic cell count is one of the associated traits in the study [78].

Monocytes are a type of white blood cell formed in the bone marrow. They play a vital role in the body's immune response. Differentiated monocytes play a role in phagocytosis and antigen presentation, contributing to adaptive immunity. In sheep, monocytes and macrophages act synergistically to counteract the septicemic conditions of Coxiella burnetii and lentivirus [79]. The genome-wide association study in Ramboulliet, Polypay and Columbia sheep identified the novel loci associated with the monocyte count on OAR 1, 2, 3, 4, 9, 10, 15, and 16. The genomic analysis revealed the loci associated with the monocyte count in the Deccani sheep genome on OAR4.

The DCMS analyses identified two other significant loci on chromosome 4 associated with foot rot and pneumonia susceptibility in sheep. Interestingly, the GWAS study in Katahdin, Blackbelly, and European-influenced crossbred sheep determined the strong associations of foot rot susceptibility in hairy sheep [80]. Their study uncovered a few loci on chromosome 4 related to foot rot susceptibility.

The genomic analyses revealed a significant association between body length and body weight on chromosomes 21 and 2. Interestingly, a genome-wide association

investigation on Qira Black and German Merino sheep indicated the association between the QTL region with the body measurements and body weight traits [81].

The present study identified two solid genomic associations on chromosome 2 in Deccani sheep related to water-holding capacity (a meat quality trait) and horn type. Deccani sheep are valued among consumers for their better meat quality traits. In addition, the sheep's physical characteristics, especially horns, are unique to this breed. Interestingly, the GWAS on the Colombian Creole hair sheep showed strong associations between the genomic regions on chromosome 2 and the water-holding capacity in meat [82]. Another genomic study demonstrated the genomic basis for the horn polymorphism in wild Soay sheep. The study reported a strong association of the genomic portions on chromosome 2 [83].

The findings of this study closely align with previous genomic research on Chantangi sheep [14], reinforcing the results concerning significant genomic regions and their associations with various traits. Similar to earlier studies [13,14,48], our investigation highlights key candidate genes that influence crucial characteristics such as growth, wool production, and disease resistance. These findings not only validate the genomic insights previously established for Indian sheep breeds but also contribute to a deeper understanding of the genetic foundations of these traits across different sheep populations. The consistency across studies emphasises the importance of these genomic regions in sheep breeding programmes aimed at enhancing desirable traits.

Conclusion:

We identified 51 putative QTL regions encompassing 45 protein-coding genes in Indian sheep breeds under selection and adapted to unique and distinct local climates. However, previous studies on the selection signatures of these breeds have been reported using few single statistics. Our study used a novel composite selection signature method (The de-correlated composite selection signal method) combining *p*-values into a single measure. This method improves the resolution power of the genomic signals and prioritises the functional candidate genes more effectively than single statistics. The putative QTLs and functional candidate genes revealed in the study were based on the historical background of these breeds. The identified signature genomic regions were associated with economic traits, including body growth, fecundity, heat stress response, domestication, environmental adaptation, hair follicle growth, horn type, and meat quality yield. Interestingly, this study identified the genomic regions associated with hair follicle growth in Chanthangi, fecundity and the number of lambs born alive in Garole, and meat quality traits in Deccani. Our research findings indicate the requisite knowledge to better comprehend the molecular processes implicated in crucial traits and local adaptation. The potential limitations of the present study include the small sample size and the ascertainment bias in the genomic data. The genotypic data from the SNP chip used in the selection signature studies is derived from a restricted set of known markers. Furthermore, alleles with a low minor allele frequency (MAF) are often overlooked. This ascertainment bias may result in misleading findings. The bottleneck effect, founder effects, and reduced recombination rates can create patterns that resemble those caused by natural selection in small sample

sizes. However, whole genome studies with better depth of sequencing, greater population size, and phenotypic observations could uncover the potential candidate genes under selection.

Declarations

Ethics approval and consent to participate

Not applicable

Consent for publication

Not applicable

Availability of data and materials

The genotypic data of indigenous Sheep breeds of India (Chantangi, Garole, and Deccani; n=29, 24, and 26, respectively) were obtained from the WIDDE database with the following link: http://widde.toulouse.inra.fr/widde/widde/main.do?module = sheep.

Competing interests

The authors declare that they have no competing interests.

Funding

This research received no specific grant from the public, commercial, or not-for-profit funding agencies.

Authors' contributions

SKI and SN conceived the idea, SN, SKI, DWM, SSM, AM and VKY performed the entire bioinformatics analysis, SKI and SN wrote the manuscript, SSM, DWM, AM and VKY edited and proofread the manuscript, and DWM and SKI supervised the whole project. All authors read the manuscript and approved the final manuscript.

Acknowledgements

The first and second authors duly acknowledge the Sri Venkateswara Veterinary University, Tirupati, Andhra Pradesh, for the necessary help and support in conducting this study.

References:

[1] Darwin C. 1859The origin of species. London: Murray: 1962.

[2] Fay JC, Wu CI. Hitchhiking under positive Darwinian selection. Genetics 2000;155:1405–13. https://doi.org/10.1093/genetics/155.3.1405.

[3] Jensen JD, Foll M, Bernatchez L. The past, present and future of genomic scans for selection. Mol Ecol 2016. https://doi.org/10.1111/mec.13493.

[4] Kim Y, Nielsen R. Linkage Disequilibrium as a Signature of Selective Sweeps 2004;1524:1513–24. https://doi.org/10.1534/genetics.103.025387.

[5] Qanbari S, Simianer H. Mapping signatures of positive selection in the genome of

livestock. Livest Sci 2014;166:133–43. https://doi.org/10.1016/j.livsci.2014.05.003.

[6] Illa SK, Mukherjee S, Nath S, Mukherjee A. Genome-Wide Scanning for Signatures of Selection Revealed the Putative Genomic Regions and Candidate Genes Controlling Milk Composition and Coat Color Traits in Sahiwal Cattle 2021;12:1–14. https://doi.org/10.3389/fgene.2021.699422.

[7] Karsli T. Genome-wide genetic characterization and selection signatures in Anatolian Merino sheep. Arch Anim Breed 2025;68:161–9. https://doi.org/10.5194/aab-68-161-2025.

[8] Karabaş M, Yılmaz O. Identification of selection signatures and genetic diversity in the sheep. Trop Anim Health Prod 2025;57:68. https://doi.org/10.1007/s11250-025-04307-9.

[9] Rodrigues JL, Braga LG, Watanabe RN, Schenkel FS, Berry DP, Buzanskas ME, et al. Genetic diversity and selection signatures in sheep breeds. J Appl Genet 2025. https://doi.org/10.1007/s13353-025-00941-z.

[10] Grossman SR, Shylakhter I, Karlsson EK, Byrne EH, Morales S, Frieden G, et al. A composite of multiple signals distinguishes causal variants in regions of positive selection. Science (80-) 2010. https://doi.org/10.1126/science.1183863.

[11] Staubach F, Lorenc A, Messer PW, Tang K, Petrov DA, Tautz D. Genome Patterns of Selection and Introgression of Haplotypes in Natural Populations of the House Mouse (Mus musculus). PLoS Genet 2012;8. https://doi.org/10.1371/journal.pgen.1002891.

[12] Ma Y, Ding X, Qanbari S, Weigend S, Zhang Q, Simianer H. Properties of different selection signature statistics and a new strategy for combining them. Heredity (Edinb)

2015. https://doi.org/10.1038/hdy.2015.42.

[13] Lotterhos KE, Card DC, Schaal SM, Wang L, Collins C, Verity B. Composite measures of selection can improve the signal-to-noise ratio in genome scans. Methods Ecol Evol 2017. https://doi.org/10.1111/2041-210X.12774.

[14] Kijas JW, Lenstra JA, Hayes B, Boitard S, Neto LR, Cristobal MS, et al. Genome-wide analysis of the world's sheep breeds reveals high levels of historic mixture and strong recent selection. PLoS Biol 2012;10. https://doi.org/10.1371/journal.pbio.1001258.

[15] DADHF. Annual report. 2023.

[16] Saravanan KA, Panigrahi M, Kumar H, Parida S, Bhushan B, Gaur GK, et al. Genomic scans for selection signatures revealed candidate genes for adaptation and production traits in a variety of cattle breeds. Genomics 2021;113:955–63. https://doi.org/10.1016/j.ygeno.2021.02.009.

[17] Ahmad SF, Mehrotra A, Charles S, Ganai NA. Analysis of selection signatures reveals important insights into the adaptability of high-altitude Indian sheep breed Changthangi. Gene 2021;799:145809. https://doi.org/10.1016/j.gene.2021.145809.

[18] Sempéré G, Moazami-Goudarzi K, Eggen A, Laloë D, Gautier M, Flori L. WIDDE: A Web-Interfaced next generation database for genetic diversity exploration, with a first application in cattle. BMC Genomics 2015;16:1–8. https://doi.org/10.1186/s12864-015-2181-1.

[19] Cao Y, Jia P, Wu Z, Huang M, Chen S, Zhang J, et al. A novel SNP of MYO1A gene associated with heat-tolerance in Chinese cattle. Anim Biotechnol 2020;0:1–6.

https://doi.org/10.1080/10495398.2020.1837147.

[20] Zheng X, Levine D, Shen J, Gogarten SM, Laurie C, Weir BS. A high-performance computing toolset for relatedness and principal component analysis of SNP data. Bioinformatics 2012. https://doi.org/10.1093/bioinformatics/bts606.

[21] Nei M, Li WH. Mathematical model for studying genetic variation in terms of restriction endonucleases. Proc Natl Acad Sci U S A 1979. https://doi.org/10.1073/pnas.76.10.5269.

[22] Weir BS, Cockerham CC. Estimating F-statistics for the analysis of population structure. Evolution (N Y) 1984. https://doi.org/10.1111/j.1558-5646.1984.tb05657.x.

[23] Tajima F. Statistical method for testing the neutral mutation hypothesis by DNA polymorphism. Genetics 1989.

[24] Garud NR, Messer PW, Buzbas EO, Petrov DA. Recent Selective Sweeps in North American Drosophila melanogaster Show Signatures of Soft Sweeps. PLoS Genet 2015. https://doi.org/10.1371/journal.pgen.1005004.

[25] Delaneau O, Marchini J, Zagury JF. A linear complexity phasing method for thousands of genomes. Nat Methods 2012. https://doi.org/10.1038/nmeth.1785.

[26] Barbato M, Orozco-terWengel P, Tapio M, Bruford MW. SNeP: A tool to estimate trends in recent effective population size trajectories using genome-wide SNP data. Front Genet 2015. https://doi.org/10.3389/fgene.2015.00109.

[27] Petit M, Astruc JM, Sarry J, Drouilhet L, Fabre S, Moreno CR, et al. Variation in recombination rate and its genetic determinism in sheep populations. Genetics 2017;207:767–84. https://doi.org/10.1534/genetics.117.300123.

[28] Danecek P, Auton A, Abecasis G, Albers CA, Banks E, DePristo MA, et al. The variant call format and VCFtools. Bioinformatics 2011. https://doi.org/10.1093/bioinformatics/btr330.

[29] Yurchenko AA, Daetwyler HD, Yudin N, Schnabel RD, Vander Jagt CJ, Soloshenko V, et al. Scans for signatures of selection in Russian cattle breed genomes reveal new candidate genes for environmental adaptation and acclimation. Sci Rep 2018;8:1–16. https://doi.org/10.1038/s41598-018-31304-w.

[30] Verity R, Collins C, Card D, Schaal S, Wang L, Lotterhos K. MINOTAUR: A platform for the analysis and visualization of multivariate results from genome scans with R Shiny. Minotaur A Platf Anal Vis Multivar Results from Genome Scans with R Shiny 2016. https://doi.org/10.1101/062158.

[31] Todorov V, Templ M, Filzmoser P. Detection of multivariate outliers in business survey data with incomplete information. Adv Data Anal Classif 2011. https://doi.org/10.1007/s11634-010-0075-2.

[32] Venables WN, Ripley BD. Modern Applied Statistics with S Fourth edition by. 2002.

[33] Hochberg YB and Y. Controlling the False Discovery Rate: A Practical and Powerful Approach to Multiple Testing. J R Stat Soc 1995;57:289–300.

[34] Storey JD, Tibshirani R. Statistical significance for genomewide studies. Proc Natl Acad Sci U S A 2003. https://doi.org/10.1073/pnas.1530509100.

[35] Fonseca PAS, Suárez-Vega A, Marras G, Cánovas Á. GALLO: An R package for genomic annotation and integration of multiple data sources in livestock for positional

candidate loci. Gigascience 2020. https://doi.org/10.1093/gigascience/giaa149.

[36] Hu ZL, Park CA, Reecy JM. Building a livestock genetic and genomic information knowledgebase through integrative developments of Animal QTLdb and CorrDB. Nucleic Acids Res 2019. https://doi.org/10.1093/nar/gky1084.

[37] Saravanan KA, Panigrahi M, Kumar H, Bhushan B, Dutt T, Mishra BP. Genome-wide analysis of genetic diversity and selection signatures in three Indian sheep breeds. Livest Sci 2021;243:104367. https://doi.org/10.1016/j.livsci.2020.104367.

[38] Eydivandi S, Roudbar MA, Karimi MO, Sahana G. Genomic scans for selective sweeps through haplotype homozygosity and allelic fixation in 14 indigenous sheep breeds from Middle East and South Asia. Sci Rep 2021;11:1–18. https://doi.org/10.1038/s41598-021-82625-2.

[39] Hirschhorn J, Ingelsson E, Genet N. Association analyses of 249,796 individuals reveal eighteen new loci associated with body mass index HHS Public Access Author manuscript 2011;42:937–48. https://doi.org/10.1038/ng.686.Association.

[40] Zielke LG, Bortfeldt RH, Tetens J, Brockmann GA. BDNF contributes to the genetic variance of milk fat yield in German Holstein cattle. Front Genet 2011;2:1–8. https://doi.org/10.3389/fgene.2011.00016.

[41] Rahmatalla SA, Arends D, Reissmann M, Wimmers K, Reyer H, Brockmann GA. Genome-wide association study of body morphological traits in Sudanese goats. Anim Genet 2018;49:478–82. https://doi.org/10.1111/age.12686.

[42] Duan Y, Su P, Gu Y, Lv X, Cao X, Wang S, et al. A Study of the Resistance of Hu Sheep

Lambs to Escherichia coli F17 Based on Whole Genome Sequencing. Animals 2024;14:1–12. https://doi.org/10.3390/ani14010161.

[43] Purdie AC, Plain KM, Begg DJ, de Silva K, Whittington RJ. Gene expression profiles during subclinical Mycobacterium avium subspecies paratuberculosis infection in sheep can predict disease outcome. Sci Rep 2019;9:1–15. https://doi.org/10.1038/s41598-019-44670-w.

[44] Ito M, Yamanashi Y, Toyoda Y, Izumi-Nakaseko H, Oda S, Sugiyama A, et al. Disruption of Stard10 gene alters the PPARα-mediated bile acid homeostasis. Biochim Biophys Acta - Mol Cell Biol Lipids 2013;1831:459–68. https://doi.org/10.1016/j.bbalip.2012.11.008.

[45] Yang J, Wang DF, Huang JH, Zhu QH, Luo LY, Lu R, et al. Structural variant landscapes reveal convergent signatures of evolution in sheep and goats. Genome Biol 2024;25:1–46. https://doi.org/10.1186/s13059-024-03288-6.

[46] Xu P, Wang C, Xiang W, Liang Y, Li Y, Zhang X, et al. P2RY6 Has a Critical Role in Mouse Skin Carcinogenesis by Regulating the YAP and β-Catenin Signaling Pathways. J Invest Dermatol 2022;142:2334-2342.e8. https://doi.org/10.1016/j.jid.2022.02.017.

[47] Tian D, Pei Q, Jiang H, Guo J, Ma X, Han B, et al. Comprehensive analysis of the expression profiles of mRNA, lncRNA, circRNA, and miRNA in primary hair follicles of coarse sheep fetal skin. BMC Genomics 2024;25:1–15. https://doi.org/10.1186/s12864-024-10427-7.

[48] Pan R, Qi L, Xu Z, Zhang D, Nie Q, Zhang X, et al. Weighted single-step GWAS identified candidate genes associated with carcass traits in a Chinese yellow-feathered chicken population. Poult Sci 2024;103:1–14. https://doi.org/10.1016/j.psj.2023.103341.

[49] Gu S, Huang Q, Sun C, Wen C, Yang N. Transcriptomic and epigenomic insights into pectoral muscle fiber formation at the late embryonic development in pure chicken lines. Poult Sci 2024;103:103882. https://doi.org/10.1016/j.psj.2024.103882.

[50] Al-Mamun HA, Kwan P, Clark SA, Ferdosi MH, Tellam R, Gondro C. Genome-wide association study of body weight in Australian Merino sheep reveals an orthologous region on OAR6 to human and bovine genomic regions affecting height and weight. Genet Sel Evol 2015;47:1–11. https://doi.org/10.1186/s12711-015-0142-4.

[51] Zhang L, Ma X, Xuan J, Wang H, Yuan Z, Wu M, et al. Identification of MEF2B and TRhDE gene polymorphisms related to growth traits in a new ujumqin sheep population. PLoS One 2016;11:1–12. https://doi.org/10.1371/journal.pone.0159504.

[52] Pasandideh M, Gholizadeh M, Rahimi-Mianji G. A genome-wide association study revealed five SNPs affecting 8-month weight in sheep. Anim Genet 2020;51:973–6. https://doi.org/10.1111/age.12996.

[53] Armstrong E, Ciappesoni G, Iriarte W, Da Silva C, Macedo F, Navajas EA, et al. Novel genetic polymorphisms associated with carcass traits in grazing Texel sheep. Meat Sci 2018;145:202–8. https://doi.org/10.1016/j.meatsci.2018.06.014.

[54] Gonzalez M V., Mousel MR, Herndon DR, Jiang Y, Dalrymple BP, Reynolds JO, et al. A Divergent Artiodactyl MYADM-like Repeat Is Associated with Erythrocyte Traits and Weight of Lamb Weaned in Domestic Sheep. PLoS One 2013;8. https://doi.org/10.1371/journal.pone.0074700.

[55] Li H, Wu XL, Tait RG, Bauck S, Thomas DL, Murphy TW, et al. Genome-wide association study of milk production traits in a crossbred dairy sheep population using

three statistical models. Anim Genet 2020;51:624–8. https://doi.org/10.1111/age.12956.

[56] Peng WF, Xu SS, Ren X, Lv FH, Xie XL, Zhao YX, et al. A genome-wide association study reveals candidate genes for the supernumerary nipple phenotype in sheep (Ovis aries). Anim Genet 2017;48:570–9. https://doi.org/10.1111/age.12575.

[57] Wen J, Toomer KH, Chen Z, Cai X. Genome-wide analysis of alternative transcripts in human breast cancer. Breast Cancer Res Treat 2015;151:295–307. https://doi.org/10.1007/s10549-015-3395-2.

[58] Chen W, Li Z, Zhong R, Sun W, Chu M. Expression profiles of oviductal mRNAs and lncRNAs in the follicular phase and luteal phase of sheep (Ovis aries) with 2 fecundity gene (FecB) genotypes. G3 Genes, Genomes, Genet 2024;14:1–16. https://doi.org/10.1093/g3journal/jkad270.

[59] Deng M, Zhang G, Cai Y, Liu Z, Zhang Y, Meng F, et al. DNA methylation dynamics during zygotic genome activation in goat. Theriogenology 2020;156:144–54. https://doi.org/10.1016/j.theriogenology.2020.07.008.

[60] Bao J, Xiong J, Huang J, Yang P, Shang M, Zhang L. Genetic Diversity, Selection Signatures, and Genome-Wide Association Study Identify Candidate Genes Related to Litter Size in Hu Sheep. Int J Mol Sci 2024;25. https://doi.org/10.3390/ijms25179397.

[61] Sugimoto M, Gotoh Y, Kawahara T, Sugimoto Y. Molecular effects of polymorphism in the 3'UTR of unc-5 homolog C associated with conception rate in Holsteins. PLoS One 2015;10:1–14. https://doi.org/10.1371/journal.pone.0131283.

[62] Zhao F, Xie R, Fang L, Xiang R, Yuan Z, Liu Y, et al. Analysis of 206 whole-genome

resequencing reveals selection signatures associated with breed-specific traits in Hu sheep. Evol Appl 2024;17:1–12. https://doi.org/10.1111/eva.13697.

[63] Zhong T, Hou D, Zhao Q, Zhan S, Wang L, Li L, et al. Comparative whole-genome resequencing to uncover selection signatures linked to litter size in Hu Sheep and five other breeds. BMC Genomics 2024;25:1–13. https://doi.org/10.1186/s12864-024-10396-x.

[64] Eydivandi S, Roudbar MA, Ardestani SS, Momen M, Sahana G. A selection signatures study among Middle Eastern and European sheep breeds. J Anim Breed Genet 2021;138:574–88. https://doi.org/10.1111/jbg.12536.

[65] Karaoglan I, Pehlivan S, Namiduru M, Pehlivan M, Kilinçarslan C, Balkan Y, et al. TNF-α, TGF-β, IL-10, IL-6 and IFN-γ gene polymorphisms as risk factors for brucellosis. New Microbiol 2009;32:173–8.

[66] Li X, Wu Q, Zhang X, Li C, Zhang D, Li G, et al. Whole-Genome Resequencing to Study Brucellosis Susceptibility in Sheep. Front Genet 2021;12:1–9. https://doi.org/10.3389/fgene.2021.653927.

[67] Zhao G, Li X, Miao H, Chen S, Hou Y. Estrogen promotes cAMP production in Mesenchymal stem cells by regulating ADCY2. Int J Stem Cells 2020;13:55–64. https://doi.org/10.15283/IJSC19139.

[68] Matika O, Riggio V, Anselme-Moizan M, Law AS, Pong-Wong R, Archibald AL, et al. Genome-wide association reveals QTL for growth, bone and in vivo carcass traits as assessed by computed tomography in Scottish Blackface lambs. Genet Sel Evol 2016;48:1–15. https://doi.org/10.1186/s12711-016-0191-3.

[69] Xu SS, Gao L, Xie XL, Ren YL, Shen ZQ, Wang F, et al. Genome-wide association analyses highlight the potential for different genetic mechanisms for litter size among sheep breeds. Front Genet 2018;9:1–14. https://doi.org/10.3389/fgene.2018.00118.

[70] Abdoli R, Mirhoseini SZ, Ghavi Hossein-Zadeh N, Zamani P, Moradi MH, Ferdosi MH, et al. Runs of homozygosity and cross-generational inbreeding of Iranian fat-tailed sheep. Heredity (Edinb) 2023;130:358–67. https://doi.org/10.1038/s41437-023-00611-y.

[71] Li X, Ding N, Zhang Z, Tian D, Han B, Liu S, et al. Identification of Somatostatin Receptor Subtype 1 (SSTR1) Gene Polymorphism and Their Association with Growth Traits in Hulun Buir Sheep. Genes (Basel) 2022;13. https://doi.org/10.3390/genes13010077.

[72] Chen Y, Hu S, Mu L, Zhao B, Wang M, Yang N, et al. Slc7a11 modulated by POU2f1 is involved in pigmentation in rabbit. Int J Mol Sci 2019;20:1–16. https://doi.org/10.3390/ijms20102493.

[73] Yang N, Zhao B, Hu S, Bao Z, Liu M, Chen Y, et al. Characterization of POU2F1 gene and its potential impact on the expression of genes involved in fur color formation in rex rabbit. Genes (Basel) 2020;11. https://doi.org/10.3390/genes11050575.

[74] Ahbara A, Bahbahani H, Almathen F, Abri M Al, Agoub MO, Abeba A, et al. Genome-wide variation, candidate regions and genes associated with fat deposition and tail morphology in Ethiopian indigenous sheep. Front Genet 2019;10:1–21. https://doi.org/10.3389/fgene.2018.00699.

[75] Casas E, Shackelford SD, Keele JW, Stone RT, Kappes SM, Koohmaraie M. Quantitative trait loci affecting growth and carcass composition of cattle segregating alternate forms of

myostatin. J Anim Sci 2000;78:560–9. https://doi.org/10.2527/2000.783560x.

[76] McClure MC, Morsci NS, Schnabel RD, Kim JW, Yao P, Rolf MM, et al. A genome scan for quantitative trait loci influencing carcass, post-natal growth and reproductive traits in commercial Angus cattle. Anim Genet 2010;41:597–607. https://doi.org/10.1111/j.1365-2052.2010.02063.x.

[77] Samani A, Karuppasamy M, English KG, Siler CA, Wang Y, Widrick JJ, et al. glucose metabolism 2024;37:1–27. https://doi.org/10.1096/fj.202300386RR.DOCK3.

[78] Marina H, Pelayo R, Suárez-Vega A, Gutiérrez-Gil B, Esteban-Blanco C, Arranz JJ. Genome-wide association studies (GWAS) and post-GWAS analyses for technological traits in Assaf and Churra dairy breeds. J Dairy Sci 2021;104:11850–66. https://doi.org/10.3168/jds.2021-20510.

[79] Oliveira RD, Mousel MR, Gonzalez M V., Durfee CJ, Davenport KM, Murdoch BM, et al. A high-density genome-wide association with absolute blood monocyte count in domestic sheep identifies novel loci. PLoS One 2022;17:1–15. https://doi.org/10.1371/journal.pone.0266748.

[80] Cinar MU, Oliveira RD, Hadfield TS, Lichtenwalner A, Brzozowski RJ, Settlemire CT, et al. Genome-wide association with footrot in hair and wool sheep. Front Genet 2023;14:1–9. https://doi.org/10.3389/fgene.2023.1297444.

[81] Tuersuntuoheti M, Zhang J, Zhou W, Zhang CL, Liu C, Chang Q, et al. Exploring the growth trait molecular markers in two sheep breeds based on Genome-wide association analysis. PLoS One 2023;18:1–18. https://doi.org/10.1371/journal.pone.0283383.

[82] Revelo HA, López-Alvarez D, Palacios YA, Vergara OD, Yánez MB, Ariza MF, et al. Genome-wide association study reveals candidate genes for traits related to meat quality in Colombian Creole hair sheep. Trop Anim Health Prod 2023;55:1–13. https://doi.org/10.1007/s11250-023-03688-z.

[83] Johnston SE, McEwan JC, Pickering NK, Kijas JW, Beraldi D, Pilkington JG, et al. Genome-wide association mapping identifies the genetic basis of discrete and quantitative variation in sexual weaponry in a wild sheep population. Mol Ecol 2011;20:2555–66. https://doi.org/10.1111/j.1365-294X.2011.05076.x.

[1] Darwin C. 1859The origin of species. London: Murray: 1962.

[2] Fay JC, Wu CI. Hitchhiking under positive Darwinian selection. Genetics 2000;155:1405–13. https://doi.org/10.1093/genetics/155.3.1405.

[3] Jensen JD, Foll M, Bernatchez L. The past, present and future of genomic scans for selection. Mol Ecol 2016. https://doi.org/10.1111/mec.13493.

[4] Kim Y, Nielsen R. Linkage Disequilibrium as a Signature of Selective Sweeps 2004;1524:1513–24. https://doi.org/10.1534/genetics.103.025387.

[5] Qanbari S, Simianer H. Mapping signatures of positive selection in the genome of livestock. Livest Sci 2014;166:133–43. https://doi.org/10.1016/j.livsci.2014.05.003.

[6] Illa SK, Mukherjee S, Nath S, Mukherjee A. Genome-Wide Scanning for Signatures of Selection Revealed the Putative Genomic Regions and Candidate Genes Controlling Milk Composition and Coat Color Traits in Sahiwal Cattle 2021;12:1–14. https://doi.org/10.3389/fgene.2021.699422.

[7] Karsli T. Genome-wide genetic characterization and selection signatures in Anatolian Merino sheep. Arch Anim Breed 2025;68:161–9. https://doi.org/10.5194/aab-68-161-2025.

[8] Karabaş M, Yılmaz O. Identification of selection signatures and genetic diversity in the sheep. Trop Anim Health Prod 2025;57:68. https://doi.org/10.1007/s11250-025-04307-9.

[9] Rodrigues JL, Braga LG, Watanabe RN, Schenkel FS, Berry DP, Buzanskas ME, et al. Genetic diversity and selection signatures in sheep breeds. J Appl Genet 2025. https://doi.org/10.1007/s13353-025-00941-z.

[10] Grossman SR, Shylakhter I, Karlsson EK, Byrne EH, Morales S, Frieden G, et al. A composite of multiple signals distinguishes causal variants in regions of positive selection. Science (80-) 2010. https://doi.org/10.1126/science.1183863.

[11] Staubach F, Lorenc A, Messer PW, Tang K, Petrov DA, Tautz D. Genome Patterns of Selection and Introgression of Haplotypes in Natural Populations of the House Mouse (Mus musculus). PLoS Genet 2012;8. https://doi.org/10.1371/journal.pgen.1002891.

[12] Ma Y, Ding X, Qanbari S, Weigend S, Zhang Q, Simianer H. Properties of different selection signature statistics and a new strategy for combining them. Heredity (Edinb) 2015. https://doi.org/10.1038/hdy.2015.42.

[13] Lotterhos KE, Card DC, Schaal SM, Wang L, Collins C, Verity B. Composite measures of selection can improve the signal-to-noise ratio in genome scans. Methods Ecol Evol 2017. https://doi.org/10.1111/2041-210X.12774.

[14] Kijas JW, Lenstra JA, Hayes B, Boitard S, Neto LR, Cristobal MS, et al. Genome-wide

analysis of the world's sheep breeds reveals high levels of historic mixture and strong recent selection. PLoS Biol 2012;10. https://doi.org/10.1371/journal.pbio.1001258.

[15] DADHF. Annual report. 2023.

[16] Saravanan KA, Panigrahi M, Kumar H, Parida S, Bhushan B, Gaur GK, et al. Genomic scans for selection signatures revealed candidate genes for adaptation and production traits in a variety of cattle breeds. Genomics 2021;113:955–63. https://doi.org/10.1016/j.ygeno.2021.02.009.

[17] Ahmad SF, Mehrotra A, Charles S, Ganai NA. Analysis of selection signatures reveals important insights into the adaptability of high-altitude Indian sheep breed Changthangi. Gene 2021;799:145809. https://doi.org/10.1016/j.gene.2021.145809.

[18] Sempéré G, Moazami-Goudarzi K, Eggen A, Laloë D, Gautier M, Flori L. WIDDE: A Web-Interfaced next generation database for genetic diversity exploration, with a first application in cattle. BMC Genomics 2015;16:1–8. https://doi.org/10.1186/s12864-015-2181-1.

[19] Cao Y, Jia P, Wu Z, Huang M, Chen S, Zhang J, et al. A novel SNP of MYO1A gene associated with heat-tolerance in Chinese cattle. Anim Biotechnol 2020;0:1–6. https://doi.org/10.1080/10495398.2020.1837147.

[20] Zheng X, Levine D, Shen J, Gogarten SM, Laurie C, Weir BS. A high-performance computing toolset for relatedness and principal component analysis of SNP data. Bioinformatics 2012. https://doi.org/10.1093/bioinformatics/bts606.

[21] Nei M, Li WH. Mathematical model for studying genetic variation in terms of restriction

endonucleases. Proc Natl Acad Sci U S A 1979. https://doi.org/10.1073/pnas.76.10.5269.

[22] Weir BS, Cockerham CC. Estimating F-statistics for the analysis of population structure. Evolution (N Y) 1984. https://doi.org/10.1111/j.1558-5646.1984.tb05657.x.

[23] Tajima F. Statistical method for testing the neutral mutation hypothesis by DNA polymorphism. Genetics 1989.

[24] Garud NR, Messer PW, Buzbas EO, Petrov DA. Recent Selective Sweeps in North American Drosophila melanogaster Show Signatures of Soft Sweeps. PLoS Genet 2015. https://doi.org/10.1371/journal.pgen.1005004.

[25] Delaneau O, Marchini J, Zagury JF. A linear complexity phasing method for thousands of genomes. Nat Methods 2012. https://doi.org/10.1038/nmeth.1785.

[26] Barbato M, Orozco-terWengel P, Tapio M, Bruford MW. SNeP: A tool to estimate trends in recent effective population size trajectories using genome-wide SNP data. Front Genet 2015. https://doi.org/10.3389/fgene.2015.00109.

[27] Petit M, Astruc JM, Sarry J, Drouilhet L, Fabre S, Moreno CR, et al. Variation in recombination rate and its genetic determinism in sheep populations. Genetics 2017;207:767–84. https://doi.org/10.1534/genetics.117.300123.

[28] Danecek P, Auton A, Abecasis G, Albers CA, Banks E, DePristo MA, et al. The variant call format and VCFtools. Bioinformatics 2011. https://doi.org/10.1093/bioinformatics/btr330.

[29] Yurchenko AA, Daetwyler HD, Yudin N, Schnabel RD, Vander Jagt CJ, Soloshenko V, et al. Scans for signatures of selection in Russian cattle breed genomes reveal new candidate

genes for environmental adaptation and acclimation. Sci Rep 2018;8:1–16. https://doi.org/10.1038/s41598-018-31304-w.

[30] Verity R, Collins C, Card D, Schaal S, Wang L, Lotterhos K. MINOTAUR: A platform for the analysis and visualization of multivariate results from genome scans with R Shiny. Minotaur A Platf Anal Vis Multivar Results from Genome Scans with R Shiny 2016. https://doi.org/10.1101/062158.

[31] Todorov V, Templ M, Filzmoser P. Detection of multivariate outliers in business survey data with incomplete information. Adv Data Anal Classif 2011. https://doi.org/10.1007/s11634-010-0075-2.

[32] Venables WN, Ripley BD. Modern Applied Statistics with S Fourth edition by. 2002.

[33] Hochberg YB and Y. Controlling the False Discovery Rate: A Practical and Powerful Approach to Multiple Testing. J R Stat Soc 1995;57:289–300.

[34] Storey JD, Tibshirani R. Statistical significance for genomewide studies. Proc Natl Acad Sci U S A 2003. https://doi.org/10.1073/pnas.1530509100.

[35] Fonseca PAS, Suárez-Vega A, Marras G, Cánovas Á. GALLO: An R package for genomic annotation and integration of multiple data sources in livestock for positional candidate loci. Gigascience 2020. https://doi.org/10.1093/gigascience/giaa149.

[36] Hu ZL, Park CA, Reecy JM. Building a livestock genetic and genomic information knowledgebase through integrative developments of Animal QTLdb and CorrDB. Nucleic Acids Res 2019. https://doi.org/10.1093/nar/gky1084.

[37] Saravanan KA, Panigrahi M, Kumar H, Bhushan B, Dutt T, Mishra BP. Genome-wide

analysis of genetic diversity and selection signatures in three Indian sheep breeds. Livest Sci 2021;243:104367. https://doi.org/10.1016/j.livsci.2020.104367.

[38] Eydivandi S, Roudbar MA, Karimi MO, Sahana G. Genomic scans for selective sweeps through haplotype homozygosity and allelic fixation in 14 indigenous sheep breeds from Middle East and South Asia. Sci Rep 2021;11:1–18. https://doi.org/10.1038/s41598-021-82625-2.

[39] Hirschhorn J, Ingelsson E, Genet N. Association analyses of 249,796 individuals reveal eighteen new loci associated with body mass index HHS Public Access Author manuscript 2011;42:937–48. https://doi.org/10.1038/ng.686.Association.

[40] Zielke LG, Bortfeldt RH, Tetens J, Brockmann GA. BDNF contributes to the genetic variance of milk fat yield in German Holstein cattle. Front Genet 2011;2:1–8. https://doi.org/10.3389/fgene.2011.00016.

[41] Rahmatalla SA, Arends D, Reissmann M, Wimmers K, Reyer H, Brockmann GA. Genome-wide association study of body morphological traits in Sudanese goats. Anim Genet 2018;49:478–82. https://doi.org/10.1111/age.12686.

[42] Duan Y, Su P, Gu Y, Lv X, Cao X, Wang S, et al. A Study of the Resistance of Hu Sheep Lambs to Escherichia coli F17 Based on Whole Genome Sequencing. Animals 2024;14:1–12. https://doi.org/10.3390/ani14010161.

[43] Purdie AC, Plain KM, Begg DJ, de Silva K, Whittington RJ. Gene expression profiles during subclinical Mycobacterium avium subspecies paratuberculosis infection in sheep can predict disease outcome. Sci Rep 2019;9:1–15. https://doi.org/10.1038/s41598-019-44670-w.

[44] Ito M, Yamanashi Y, Toyoda Y, Izumi-Nakaseko H, Oda S, Sugiyama A, et al. Disruption of Stard10 gene alters the PPARα-mediated bile acid homeostasis. Biochim Biophys Acta - Mol Cell Biol Lipids 2013;1831:459–68. https://doi.org/10.1016/j.bbalip.2012.11.008.

[45] Yang J, Wang DF, Huang JH, Zhu QH, Luo LY, Lu R, et al. Structural variant landscapes reveal convergent signatures of evolution in sheep and goats. Genome Biol 2024;25:1–46. https://doi.org/10.1186/s13059-024-03288-6.

[46] Xu P, Wang C, Xiang W, Liang Y, Li Y, Zhang X, et al. P2RY6 Has a Critical Role in Mouse Skin Carcinogenesis by Regulating the YAP and β-Catenin Signaling Pathways. J Invest Dermatol 2022;142:2334-2342.e8. https://doi.org/10.1016/j.jid.2022.02.017.

[47] Tian D, Pei Q, Jiang H, Guo J, Ma X, Han B, et al. Comprehensive analysis of the expression profiles of mRNA, lncRNA, circRNA, and miRNA in primary hair follicles of coarse sheep fetal skin. BMC Genomics 2024;25:1–15. https://doi.org/10.1186/s12864-024-10427-7.

[48] Pan R, Qi L, Xu Z, Zhang D, Nie Q, Zhang X, et al. Weighted single-step GWAS identified candidate genes associated with carcass traits in a Chinese yellow-feathered chicken population. Poult Sci 2024;103:1–14. https://doi.org/10.1016/j.psj.2023.103341.

[49] Gu S, Huang Q, Sun C, Wen C, Yang N. Transcriptomic and epigenomic insights into pectoral muscle fiber formation at the late embryonic development in pure chicken lines. Poult Sci 2024;103:103882. https://doi.org/10.1016/j.psj.2024.103882.

[50] Al-Mamun HA, Kwan P, Clark SA, Ferdosi MH, Tellam R, Gondro C. Genome-wide association study of body weight in Australian Merino sheep reveals an orthologous region on OAR6 to human and bovine genomic regions affecting height and weight. Genet

Sel Evol 2015;47:1–11. https://doi.org/10.1186/s12711-015-0142-4.

[51] Zhang L, Ma X, Xuan J, Wang H, Yuan Z, Wu M, et al. Identification of MEF2B and TRhDE gene polymorphisms related to growth traits in a new ujumqin sheep population. PLoS One 2016;11:1–12. https://doi.org/10.1371/journal.pone.0159504.

[52] Pasandideh M, Gholizadeh M, Rahimi-Mianji G. A genome-wide association study revealed five SNPs affecting 8-month weight in sheep. Anim Genet 2020;51:973–6. https://doi.org/10.1111/age.12996.

[53] Armstrong E, Ciappesoni G, Iriarte W, Da Silva C, Macedo F, Navajas EA, et al. Novel genetic polymorphisms associated with carcass traits in grazing Texel sheep. Meat Sci 2018;145:202–8. https://doi.org/10.1016/j.meatsci.2018.06.014.

[54] Gonzalez M V., Mousel MR, Herndon DR, Jiang Y, Dalrymple BP, Reynolds JO, et al. A Divergent Artiodactyl MYADM-like Repeat Is Associated with Erythrocyte Traits and Weight of Lamb Weaned in Domestic Sheep. PLoS One 2013;8. https://doi.org/10.1371/journal.pone.0074700.

[55] Li H, Wu XL, Tait RG, Bauck S, Thomas DL, Murphy TW, et al. Genome-wide association study of milk production traits in a crossbred dairy sheep population using three statistical models. Anim Genet 2020;51:624–8. https://doi.org/10.1111/age.12956.

[56] Peng WF, Xu SS, Ren X, Lv FH, Xie XL, Zhao YX, et al. A genome-wide association study reveals candidate genes for the supernumerary nipple phenotype in sheep (Ovis aries). Anim Genet 2017;48:570–9. https://doi.org/10.1111/age.12575.

[57] Wen J, Toomer KH, Chen Z, Cai X. Genome-wide analysis of alternative transcripts in

human breast cancer. Breast Cancer Res Treat 2015;151:295–307. https://doi.org/10.1007/s10549-015-3395-2.

[58] Chen W, Li Z, Zhong R, Sun W, Chu M. Expression profiles of oviductal mRNAs and lncRNAs in the follicular phase and luteal phase of sheep (Ovis aries) with 2 fecundity gene (FecB) genotypes. G3 Genes, Genomes, Genet 2024;14:1–16. https://doi.org/10.1093/g3journal/jkad270.

[59] Deng M, Zhang G, Cai Y, Liu Z, Zhang Y, Meng F, et al. DNA methylation dynamics during zygotic genome activation in goat. Theriogenology 2020;156:144–54. https://doi.org/10.1016/j.theriogenology.2020.07.008.

[60] Bao J, Xiong J, Huang J, Yang P, Shang M, Zhang L. Genetic Diversity, Selection Signatures, and Genome-Wide Association Study Identify Candidate Genes Related to Litter Size in Hu Sheep. Int J Mol Sci 2024;25. https://doi.org/10.3390/ijms25179397.

[61] Sugimoto M, Gotoh Y, Kawahara T, Sugimoto Y. Molecular effects of polymorphism in the 3’UTR of unc-5 homolog C associated with conception rate in Holsteins. PLoS One 2015;10:1–14. https://doi.org/10.1371/journal.pone.0131283.

[62] Zhao F, Xie R, Fang L, Xiang R, Yuan Z, Liu Y, et al. Analysis of 206 whole-genome resequencing reveals selection signatures associated with breed-specific traits in Hu sheep. Evol Appl 2024;17:1–12. https://doi.org/10.1111/eva.13697.

[63] Zhong T, Hou D, Zhao Q, Zhan S, Wang L, Li L, et al. Comparative whole-genome resequencing to uncover selection signatures linked to litter size in Hu Sheep and five other breeds. BMC Genomics 2024;25:1–13. https://doi.org/10.1186/s12864-024-10396-x.

[64] Eydivandi S, Roudbar MA, Ardestani SS, Momen M, Sahana G. A selection signatures study among Middle Eastern and European sheep breeds. J Anim Breed Genet 2021;138:574–88. https://doi.org/10.1111/jbg.12536.

[65] Karaoglan I, Pehlivan S, Namiduru M, Pehlivan M, Kilinçarslan C, Balkan Y, et al. TNF-α, TGF-β, IL-10, IL-6 and IFN-γ gene polymorphisms as risk factors for brucellosis. New Microbiol 2009;32:173–8.

[66] Li X, Wu Q, Zhang X, Li C, Zhang D, Li G, et al. Whole-Genome Resequencing to Study Brucellosis Susceptibility in Sheep. Front Genet 2021;12:1–9. https://doi.org/10.3389/fgene.2021.653927.

[67] Zhao G, Li X, Miao H, Chen S, Hou Y. Estrogen promotes cAMP production in Mesenchymal stem cells by regulating ADCY2. Int J Stem Cells 2020;13:55–64. https://doi.org/10.15283/IJSC19139.

[68] Matika O, Riggio V, Anselme-Moizan M, Law AS, Pong-Wong R, Archibald AL, et al. Genome-wide association reveals QTL for growth, bone and in vivo carcass traits as assessed by computed tomography in Scottish Blackface lambs. Genet Sel Evol 2016;48:1–15. https://doi.org/10.1186/s12711-016-0191-3.

[69] Xu SS, Gao L, Xie XL, Ren YL, Shen ZQ, Wang F, et al. Genome-wide association analyses highlight the potential for different genetic mechanisms for litter size among sheep breeds. Front Genet 2018;9:1–14. https://doi.org/10.3389/fgene.2018.00118.

[70] Abdoli R, Mirhoseini SZ, Ghavi Hossein-Zadeh N, Zamani P, Moradi MH, Ferdosi MH, et al. Runs of homozygosity and cross-generational inbreeding of Iranian fat-tailed sheep. Heredity (Edinb) 2023;130:358–67. https://doi.org/10.1038/s41437-023-00611-y.

[71] Li X, Ding N, Zhang Z, Tian D, Han B, Liu S, et al. Identification of Somatostatin Receptor Subtype 1 (SSTR1) Gene Polymorphism and Their Association with Growth Traits in Hulun Buir Sheep. Genes (Basel) 2022;13. https://doi.org/10.3390/genes13010077.

[72] Chen Y, Hu S, Mu L, Zhao B, Wang M, Yang N, et al. Slc7a11 modulated by POU2f1 is involved in pigmentation in rabbit. Int J Mol Sci 2019;20:1–16. https://doi.org/10.3390/ijms20102493.

[73] Yang N, Zhao B, Hu S, Bao Z, Liu M, Chen Y, et al. Characterization of POU2F1 gene and its potential impact on the expression of genes involved in fur color formation in rex rabbit. Genes (Basel) 2020;11. https://doi.org/10.3390/genes11050575.

[74] Ahbara A, Bahbahani H, Almathen F, Abri M Al, Agoub MO, Abeba A, et al. Genome-wide variation, candidate regions and genes associated with fat deposition and tail morphology in Ethiopian indigenous sheep. Front Genet 2019;10:1–21. https://doi.org/10.3389/fgene.2018.00699.

[75] Casas E, Shackelford SD, Keele JW, Stone RT, Kappes SM, Koohmaraie M. Quantitative trait loci affecting growth and carcass composition of cattle segregating alternate forms of myostatin. J Anim Sci 2000;78:560–9. https://doi.org/10.2527/2000.783560x.

[76] McClure MC, Morsci NS, Schnabel RD, Kim JW, Yao P, Rolf MM, et al. A genome scan for quantitative trait loci influencing carcass, post-natal growth and reproductive traits in commercial Angus cattle. Anim Genet 2010;41:597–607. https://doi.org/10.1111/j.1365-2052.2010.02063.x.

[77] Samani A, Karuppasamy M, English KG, Siler CA, Wang Y, Widrick JJ, et al. glucose

metabolism 2024;37:1–27. https://doi.org/10.1096/fj.202300386RR.DOCK3.

[78] Marina H, Pelayo R, Suárez-Vega A, Gutiérrez-Gil B, Esteban-Blanco C, Arranz JJ. Genome-wide association studies (GWAS) and post-GWAS analyses for technological traits in Assaf and Churra dairy breeds. J Dairy Sci 2021;104:11850–66. https://doi.org/10.3168/jds.2021-20510.

[79] Oliveira RD, Mousel MR, Gonzalez M V., Durfee CJ, Davenport KM, Murdoch BM, et al. A high-density genome-wide association with absolute blood monocyte count in domestic sheep identifies novel loci. PLoS One 2022;17:1–15. https://doi.org/10.1371/journal.pone.0266748.

[80] Cinar MU, Oliveira RD, Hadfield TS, Lichtenwalner A, Brzozowski RJ, Settlemire CT, et al. Genome-wide association with footrot in hair and wool sheep. Front Genet 2023;14:1–9. https://doi.org/10.3389/fgene.2023.1297444.

[81] Tuersuntuoheti M, Zhang J, Zhou W, Zhang CL, Liu C, Chang Q, et al. Exploring the growth trait molecular markers in two sheep breeds based on Genome-wide association analysis. PLoS One 2023;18:1–18. https://doi.org/10.1371/journal.pone.0283383.

[82] Revelo HA, López-Alvarez D, Palacios YA, Vergara OD, Yánez MB, Ariza MF, et al. Genome-wide association study reveals candidate genes for traits related to meat quality in Colombian Creole hair sheep. Trop Anim Health Prod 2023;55:1–13. https://doi.org/10.1007/s11250-023-03688-z.

[83] Johnston SE, McEwan JC, Pickering NK, Kijas JW, Beraldi D, Pilkington JG, et al. Genome-wide association mapping identifies the genetic basis of discrete and quantitative variation in sexual weaponry in a wild sheep population. Mol Ecol 2011;20:2555–66.

https://doi.org/10.1111/j.1365-294X.2011.05076.x.

Fig 1: PCA analysis showing the population structure of the three Indian sheep breeds.

Fig 2: Percent QTL classes annotated in three Indian sheep breeds

Fig 3: QTL classes enriched in three Indian sheep breeds

Fig 4: Manhattan plot showing signatures of selection in three Indian sheep breeds

Manuscript Number: PONE-D-24-51732<br>Title: Composite Selection Signal Analysis: Uncovering Candidate Genes and Quantitative Trait Loci in Indian Sheep Breeds		
**S.No.**	**Reviewers Comments**	**Response to Reviewer's Comments**
01	Certain paragraphs in the introduction and discussion are redundant and could be condensed.	**Thank you for pointing that out. The redundancy part in the discussion is removed**
02	Specific Comments:<br>The abstract provides a good summary but lacks specific details about key findings. Including numerical results or specific genes/QTLs would enhance its informativeness.	**Thank you for bringing that issue to our attention. The main findings and significant numerical results have been clearly detailed in the abstract section.**
03	The introduction is comprehensive but overly descriptive in some parts. Focus more on the knowledge gap and the study's unique contributions.	**I am grateful for your bringing this matter to my attention. I have incorporated a paragraph that addresses the existing knowledge gaps and underscores the distinctiveness of the current study.**
04	Include more references to recent studies on selection signals and genomic studies in sheep from diverse geographical contexts.	**Thank you for your valuable input.** Recent research on selection signals and global studies on sheep have been included in the references.
05	Describe the data quality control steps in more detail. For example, how were outlier SNPs or missing data handled?	**Thank you for your comments about it. Detailed the steps for controlling data quality in the methods section.**
06	Clarify the composite selection signal (CSS) methodology. Provide justification for using this approach over others (e.g., iHS, XP-EHH).	**I'm thankful for your notice on this matter. The methodology of composite selection signals and its advantages compared to alternative methods were incorporated.**
07	Were any statistical corrections applied to account for population structure or relatedness among individuals?	**Thank you for your comment. Pi hat values are computed for all individuals in the study, and the genomic relatedness among them is low. As a result, all individuals are included in the selection signature analysis.**

08	The presentation of the results is clear, but: Include figures or tables summarizing key candidate genes or QTLs identified.	**I appreciate you bringing that to my attention. The text included a table that summarizes the important candidate genes**.
09	Highlight specific loci and their putative roles in traits relevant to Indian sheep (e.g., drought tolerance, disease resistance).	**I'm grateful for your insight on this. The proposed genetic locations associated with traits such as drought tolerance and disease resistance were highlighted in the updated version.**
10	Consider including a heatmap or Manhattan plot for better visualisation of selection signals.	**I appreciate you pointing that out to me.** The manuscript now includes a Manhattan plot that effectively illustrates the selection signals identified in our analysis.
11	The discussion lacks depth in interpreting biological functions of identified genes.	**Thanks for shedding light on that**. The specifics regarding the molecular functions of the proposed genes were included in the discussion section.
12	Provide insights into how these genes/QTLs align with known traits in sheep.	**Thanks for making me aware of it. The specified details have been incorporated into the manuscript in accordance with the recommendations provided.**
13	Compare findings with results from other studies (e.g., on sheep from different regions or breeds).	**Thanks for the comment. Comparison of findings of this study with other studies were included in the revised manuscript.**
14	Discuss potential limitations of the study, such as sample size or marker density, and how they might affect the robustness of the conclusions.	**I'm grateful for your comment. The potential limitations of the study are added in the manuscript.**
15	Figures are clear but lack detailed legends. Add more context to explain what the reader should infer from each figure.	**Thank you for pointing out. However, the existing pie chart has the common legend, that explains the name of the QTL classes.**
16	Tables summarizing selection signals should include effect sizes and p-values where relevant	**Thanks for the comment. Changes incorporated as per the comment.**

Manuscript Number: PONE-D-24-51732<br>Title:Title: Composite Selection Signal Analysis: Uncovering Candidate Genes and Quantitative Trait Loci in Indian Sheep Breeds		
**Reviewer 02**		
**Specific Comments**		
**S.No.**	**Reviewer's Comments**	**Response**
01	Link Between Genomic Regions and Local Adaptation: While the discussion mentions the environmental adaptations of the breeds, it could further elaborate on how these adaptations might have influenced the specific genomic regions under selection. For example, more direct comparisons to how the genetic variations might correlate with the climate or geographical features of the areas where these breeds are found could enhance the reader's understanding of the adaptive significance of these genes.	Thank you for the suggestion.<br>Incorporated the text as suggested
02	Consistency in Gene References: Some genes are mentioned repeatedly (e.g., CNTNAP5), but the context of how they were identified in different breeds could be more explicitly connected to avoid confusion. For instance, you might clarify how CNTNAP5's role in body growth is shared across breeds like CHA and GAR, yet each breed's unique genomic context could influence the gene's effect.	I'm grateful for your suggestion and have integrated the text as suggested.
03	Discussion on Statistical Methodology: The use of the DCMS method is discussed, but more detailed insight into why this method improves the resolution power of the genomic signals could help readers understand the methodological benefits. Briefly touching on the weaknesses of other statistical approaches and how the DCMS method overcomes them would strengthen the argument	Thanks for the recommendation; I've added the text accordingly.
04	Gene Functionality and Mechanisms: While candidate genes are identified, more information on their molecular mechanisms or the processes through which they impact the phenotypic traits (e.g., how MTSS1 influences bacterial resistance) could help provide a clearer understanding of their biological relevance	I am thankful for your suggestion and have made the changes to the text as proposed.
05	Comparison with Other Studies: The discussion of prior research, such as the studies from Ahmad et al. (2021) and Zhang et al. (2016), provides useful context. However, it would be helpful to briefly	I appreciate your suggestion and have appended the changes to the text as proposed.

	explain how the findings in the current study either align or contrast with these studies, reinforcing the study's contribution to the field	
**General Comments**		
01	Rephrase as "False Discovery Rate (FDR) threshold of < 5%"	Modified as suggested
02	Genes could be presented by trait type e.g., morphology (putative genes with sub-trait), growth (putative genes with sub-trait), reproduction (putative genes with sub-trait), adaptation (putative genes with sub-trait), disease resistance (putative genes with sub-trait), etc.	Changes made as per the correction- Genes rearranged into sub-traits.
03	QTLs could be grouped by trait also.	The QTLs were grouped according to the the trait - modified as per the recommendations
04	Referencing [1] is just akwarding as the sentence approaches to introduce a concept and hence remove the reference	Removed the reference as suggested
05	Explain why the concept of selection signatures is important or how understanding them benefits evolutionary biology.	Updated as advised
06	The phrase "mutations" seems redundant, as mutations are already mentioned in the previous sentence, so restructure the present sentence.	Restructure the sentence as recommended
07	This is clear but could be more engaging. Consider adding a sentence or two on how SNP arrays have specifically impacted genomic studies, perhaps by providing concrete examples of successful applications.	Enhanced as proposed
08	Give slight details about XPEHH	Refined based on your feedback
09	How does DCMS method work or why it seems more robust and sensitive?	Edited in line with your comments.
10	This sentence seems to appear abruptly. A sentence or two linking the SNP arrays discussed previously to the OvineSNP50 Bead Chip would provide a smoother transition.	Revised according to your recommendation
11	Authors might add a bit more context about why the OvineSNP50 is specifically chosen for Indian sheep genome studies. For example, is it due to its size, variety of SNPs, or its validation in Indian sheep breeds?	Updated as advised
12	Authors might use terms like "cold arid regions of the Western Himalayas" or "semi-arid regions of the Deccan Plateau", etc. for clarity	Adjusted as indicated
13	Correct spelling (Odisha)	Corrected as suggested

14	This is a strong statement that highlights the gap in research. Authors could emphasize why this gap is significant, perhaps briefly mentioning how genomics could benefit sheep production in India.	Amended as suggested
15	Rephrase for clarity, such as: "In this study, we applied the composite signals method (DCMS) to detect selection signatures in the genomes of three Indian sheep breeds (Chantangi, Deccani, and Garole), which are adapted to diverse ecological niches and production systems across India.	Changed as per your suggestion.
16	Briefly mention why these particular breeds are chosen. Are they important economically, or do they exhibit distinct phenotypic traits?	Updated following your suggestion
17	It will be better to represent in a single sentence highlighting the goat or objective of the study.	Amended in accordance with your feedback
18	Rephrase for better flow: "In the present study, we analyzed high-density genotypes from three Indian sheep breeds: Changthangi (CHA, n = 29), Deccani (IDC, n = 24), and Garole (GAR, n = 26), genotyped using the Illumina Ovine SNP50 BeadChip, which were obtained from WIDDE [14]."	Refined as you advised
19	Consider providing a bit more context about WIDDE (e.g., what it stands for or its relevance) for clarity.	Enhanced based on your thoughts
20	For clarity, authors might say: "The Illumina Ovine SNP50 BeadChip consists of 54,241 SNPs distributed across the sheep genome, with an average density of one SNP every 51 kb."	Rephrased as recommended
21	Improve for clarity and consistency: "Quality control (QC) of the genotypes was performed based on the following criteria: minor allele frequency (maf < 0.05), Hardy-Weinberg equilibrium (HWE p < 0.0001), individuals with missing genotypes (--mind < 0.1), and SNPs with missing genotype data (geno < 0.1)."	Updated following your suggestion
22	It would be helpful to clarify why these specific thresholds (maf, HWE, etc.) were chosen.	
23	Authors might briefly explain why X-chromosome SNPs were excluded, especially if it's relevant to the study design (e.g., if focusing only on autosomes).	Explained following your feedback
24	Authors could expand this purpose of PCA slightly on what they hoped to achieve with PCA	Expanded as suggested

	(e.g., confirming population structure or identifying outliers).	
25	Mention briefly what the "snprelate" package does. For example, authors could say: "The PCA was performed using the 'snprelate' package in R, which is designed for efficient analysis of large-scale genotypic data."	Refined as you advised.
26	Authors might also consider adding a sentence explaining why DCMS is preferred in this context or how it enhances the analysis of selection signals.	Enhanced as proposed
27	The present study utilized the Composite of Multiple Signals (DCMS) method, as detailed in Yurchenko et al. (2018), combining five univariate statistics: FST, Haplotype Homozygosity (H1), Modified Haplotype Homozygosity (H12), Tajima's D, and Nucleotide Diversity (π) [18–21]."	Corrected following your input
28	Correct as 'locus'	Edited as per your suggestion
29	Consider adding a more intuitive explanation of why these components are combined (e.g., "The DCMS statistic combines both the p-values of individual statistics and their correlations to improve the sensitivity and robustness of detecting selection signals.").	Refined based on your feedback
30	briefly mention why FST is used (e.g., to quantify genetic differentiation).	Updated following your suggestion
31	explain a bit more about why these statistics are important and how they contribute to detecting selection signals (e.g., "Haplotype homozygosity measures the extent to which haplotypes are conserved, which can help identify regions under selective pressure.").	Explained, following your advice
32	consider elaborating on how they relate to detecting selection. For example, "Tajima's D detects departures from neutrality, with negative values indicating potential selective sweeps, while nucleotide diversity (π) measures the genetic variation within a population."	Enhanced as proposed
33	Authors might rephrase as "The MINOTAUR package was used to compute p-values for the five statistics based on their functional ranks,	Altered as per the comment

	integrating multiple signals into a composite measure."	
34	Consider adding a sentence or two that briefly explains the role of these steps in improving the accuracy of the results.	Adapted per your suggestion
35	Modify the criteria for determining putative regions as "A q-value threshold of 0.05 was chosen to identify genomic regions with strong evidence of selection, while adjacent SNPs with q-values greater than 0.1 were excluded to reduce false positives.").	Changed in response to your advice
36	clarify how the QTL enrichment analysis is performed. Authors might write as "QTL enrichment analysis was performed to identify overrepresented traits within the significant regions, using a genome-wide approach and a false discovery rate (FDR) threshold of 0.05.").	Revised based on your input
37	The terms are essentially redundant. Consider: "missingness thresholds for SNP genotypes (geno <0.1) and individual genotypes (mind <0.1)."	Altered per your guidance
38	Exact number or percentages of SNPs excluded for each criterion are missing.	Included in accordance with your feedback
39	Exact number or percentage of SNPs excluded for this criterion is missing. Providing these numbers or percentages of all criteria would add more transparency to the data quality process.	Included in accordance with your feedback
40	Modify as "The PCA confirmed the genetic differentiation among the three breeds, as indicated by their clear separation into distinct clusters.	Corrected following your input
41	May be presented as "PC1 accounted for 14.34% of the variation, highlighting the strongest genetic differences between the populations, while PC2 explained an additional 7.98% of the total variation, capturing finer distinctions."	Refined as you advised
42	correct whether it is "Chantangi" or "Changthangi."	Corrected as per your suggestion
43	The size range for genomic regions under selection (from 1080.32 to 1.83 Kb) is inconsistent with the typical size of genomic regions under selection in animal breeding studies. Consider reviewing these figures (DCMS Metrics) for accuracy.	We agreed to the statement you pointed out. However, the average length of genomic regions under selection was consistent with the literature, but not range. In our study, the range of genomic size we observed was not consistent with the literature.

44	It would help to summarize the QTL classes before delving into breed-specific information by adding one sentence like "The QTLs identified in each breed were categorized into various trait classes, including body weight, reproduction, milk yield, and health."	Added a sentence following your suggestion
45	but the results could be more digestible if key QTLs from the tables were also summarized in the text. For example, "The most significant QTL associated with milk yield in CHA sheep was found on OAR 16, as shown in Table 3."	Modified the sentence as per your feedback
46	Recheck to ensure the consistency and biological relevance of the findings.	
47	Can be expanded to mention any statistical findings or significance levels, if available	The significance levels were added in the sentence
48	Rephrase as "The genomic regions under selection ranged in size from 2.20 to 817.32 Kb, with an average length of 167.55 ± 216.86 Kb."	Altered per your guidance.
49	Could be expanded by specifying if any of these findings were novel or aligned with previous studies.	Expanded following your suggestions
50	Could be expanded by specifying if any of these findings were novel or aligned with previous studies.	Expanded following your suggestions
51	Can be expanded to mention any statistical findings or significance levels, if available.	The significance levels were added in the sentence
52	Consider rephrasing as: "Few studies have used multiple complementary statistics to analyze selection signatures in these breeds, and many do not account for correlations between these statistics."	Changed in response to your advice
53	Elaborate on how these adaptations might have influenced the specific genomic regions under selection. For example, more direct comparisons to how the genetic variations might correlate with the climate or geographical features of the areas where these breeds are found could enhance the reader's understanding of the adaptive significance of these genes.	Refined as per your advice
54	Explain how the findings in the current study either align or contrast with these studies.	Specified the following, per your suggestions

55	Crosscheck for completeness of citations.	Checked the reference citations as per your suggestion
56	Check for the journal style – Table heading	Checked in response to your advice

Manuscript Number: PONE-D-24-51732

Title: Composite Selection Signal Analysis: Uncovering Candidate Genes and Quantitative Trait Loci in Indian Sheep Breeds

S.No.	Reviewers Comments	Response to Reviewer's Comments
	**Reviewer 03**	
01	The manuscript, especially the discussion part is exceedingly long with redundancy in a few parts which is repeated in the results and discussion section	Thank you for pointing out it. The redundancy part in the discussion is removed
02	Check the spelling "Chantangi" which is wrongly written	Chanthangi is replaced with Chantangi
03	Concise the discussion part and avoid redundancy.	Thank you for highlighting it. The Discussion part is concised as suggested

Manuscript Number: PONE-D-24-51732<br>Title: Composite Selection Signal Analysis: Uncovering Candidate Genes and Quantitative Trait Loci in Indian Sheep Breeds		
**S.No.**	**Journal requirements**	**Response to Comments**
01	Please ensure that your manuscript meets PLOS ONE's style requirements, including those for file naming. The PLOS ONE style templates can be found at	**Thank you for pointing that out.** Followed the instructions
02	In the online submission form, you indicated that The genotypic data and other material related to this study are available with the corresponding author.<br>All PLOS journals now require all data underlying the findings described in their manuscript to be freely available to other researchers, either a. In a public repository, b. Within the manuscript itself, or c. Uploaded as supplementary information	**Thank you for bringing that issue to our attention. The data used in the study is public domain genotypic data. This was also mentioned in the manuscript.**
03	Ethics statement does not appear in the manuscript file:<br>Please include your full ethics statement in the 'Methods' section of your manuscript file. In your statement, please include the full name of the IRB or ethics committee who approved or waived your study, as well as whether or not you obtained informed written or verbal consent. If consent was waived for your study, please include this information in your statement as well.	**I am grateful for your bringing this matter to my attention. The research does not involve human or animal participants, and the data examined are publicly available. Therefore, no ethical approval or consent is necessary. This was also mentioned in the manuscript**
04	Please include a copy of Table 3 which you refer in your text	**Thank you for your valuable input.** Included now in the manuscript.
