# Supplementary material for: Composite selection signal analysis: Uncovering candidate genes and quantitative trait loci in Indian sheep breeds
Source: PLoS One. 2026 Mar 25;21(3):e0344299. doi: 10.1371/journal.pone.0344299 (PMC13016353; doi:10.1371/journal.pone.0344299)
Supplement: Attachment — Submitted filename: Review report.docx [file pone.0344299.s002.docx]

**Review Report for Manuscript: PONE-D-24-51732**

Title: “Composite Selection Signal Analysis: Uncovering Candidate Genes and Quantitative Trait Loci in Indian Sheep Breeds”

- **Authors are requested to revise the manuscript as per the comments or suggestions put into the manuscript file uploaded in the system also expected to address the issues only under Discussion section in this Review Report.**

**Strengths of the research manuscript:**

1. Clear purpose and relevance by establishing the context—exploring natural and artificial selection, its role in shaping genomes, and its relevance for breeding programs in native sheep.
2. Novelty is the introduction of the novel method (DCMS) that combines multiple p-values is well-articulated and highlights the significance of the study.
3. Key Findings are the novel genes and QTLs discovered in the Indian three native sheep breeds.

**ABSTRACT**

- The abstract is well-written and communicates the core aspects of the research, but there are some areas that could be refined to enhance clarity and flow. The list of genes and QTLs is comprehensive, but the description of their functions could be streamlined and the presentation of QTLs could be clearer if grouped by trait type (e.g., reproduction, disease resistance, growth).

**Introduction Section**

- This introduction section of the manuscript provides a good foundation, covering the importance of selection signatures and the methods used to detect them. It can be strengthened by improving the structure for clarity and flow, adding more context about why the specific methods and breeds are chosen, and making transitions smoother between sections.
  1. Authors might briefly explain why the concept of selection signatures is important or how understanding them benefits evolutionary biology.
  2. Consider adding a sentence or two on how SNP arrays have specifically impacted genomic studies, perhaps by providing concrete examples of successful applications.
  3. "Despite the rich phenotypic diversity observed in Indian sheep, comprehensive genomic studies that aim to uncover these traits' molecular basis have been relatively limited." is a strong statement that highlights the gap in research. Authors could emphasize why this gap is significant, perhaps briefly mentioning how genomics could benefit sheep production in India.
  4. The goal /objective of the study might be rephrased for clarity.
  5. It's also good to briefly mention why authors chose particular Chanthangi, Deccani, and Garole sheep breeds—are they important economically, or do they exhibit distinct phenotypic traits?

**Materials & Methods Section**

- The "Materials & Methods" section provides a detailed and technical explanation of the study's approach. However, there are a few areas where clarity could be improved, especially in terms of explaining technical terms and their significance.
  1. Instead of repeating "missing genotypes," authors could simplify it by saying: *"...individuals with missing genotypes (mind < 0.1) and SNPs with missing data (geno < 0.1)."*
  2. It would be helpful to clarify why these specific thresholds (maf, HWE, etc.) were chosen.
  3. Authors might briefly explain why X-chromosome SNPs were excluded, especially if it's relevant to the study design (e.g., if focusing only on autosomes).
  4. The purpose of PCA is clearly explained. Authors could expand slightly on what you hoped to achieve with PCA (e.g., confirming population structure or identifying outliers).
  5. It’s a good idea to mention briefly what the "snprelate" package does, especially for readers unfamiliar with it. For example, authors could say: *"The PCA was performed using the 'snprelate' package in R, which is designed for efficient analysis of large-scale genotypic data."*
  6. To make “The computation of the DCMS statistic….” sentence clearer, consider adding a more intuitive explanation of why these components are combined (e.g., *"The DCMS statistic combines both the p-values of individual statistics and their correlations to improve the sensitivity and robustness of detecting selection signals."*).
  7. Authors could briefly mention why FST is used (e.g., to quantify genetic differentiation).
  8. Authors could explain a bit more about why the H1 and H12 statistics are important and how they contribute to detecting selection signals (e.g., *"Haplotype homozygosity measures the extent to which haplotypes are conserved, which can help identify regions under selective pressure."*).
  9. Consider elaborating on how the Tajima’s D and Nucleotide Diversity (π) statistics relate to detecting selection. For example, *"Tajima's D detects departures from neutrality, with negative values indicating potential selective sweeps, while nucleotide diversity (π) measures the genetic variation within a population."*
  10. Authors could add more explanation about why these specific thresholds for determining putative regions were used (e.g., *"A q-value threshold of 0.05 was chosen to identify genomic regions with strong evidence of selection, while adjacent SNPs with q-values greater than 0.1 were excluded to reduce false positives."*).
  11. Authors could clarify how the QTL enrichment analysis is performed (e.g., *"QTL enrichment analysis was performed to identify overrepresented traits within the significant regions, using a genome-wide approach and a false discovery rate (FDR) threshold of 0.05."*).

**Results Section**

- The results section provides a detailed and comprehensive account of the DCMS, gene annotation, and QTL analysis. However, it could benefit from more precise details and clearer presentation in some parts, particularly around the filtering criteria for SNPs and more consistent formatting of breed names.

1. It is mentioned that certain SNPs were excluded for specific reasons (maf, hwe, Geno, mind), but the exact number or percentages of SNPs excluded for each criterion are missing. Providing these numbers or percentages would add more transparency to the data quality process.
2. The phrase "missingness of the genotypes (geno <0.1) and missingness genotypes (mind <0.1)" are essentially redundant. Consider: "missingness thresholds for SNP genotypes (geno <0.1) and individual genotypes (mind <0.1)."
3. The size range for genomic regions under selection (from 1080.32 to 1.83 Kb) is inconsistent with the typical size of genomic regions under selection in animal breeding studies. Consider reviewing these figures for accuracy.
4. The QTL annotation results are well-described, but it would help to summarize the QTL classes before delving into breed-specific information. For example, a sentence that explains the general approach to categorizing QTLs would provide context: "The QTLs identified in each breed were categorized into various trait classes, including body weight, reproduction, milk yield, and health."
5. The sentence "The QTL enrichment analysis revealed the top significant QTL total lambs born on OAR 16" can be expanded to mention any statistical findings or significance levels, if available.

**Discussion Section**

- The discussion provides a comprehensive and informative overview of the study’s findings. It clearly identifies key candidate genes and their potential functional roles, while also emphasizing the unique contributions of each breed. With minor revisions to improve clarity and enhance the connection between genomic findings and breed-specific traits, the discussion would be even more accessible and impactful. The study significantly advances the understanding of genomic selection in Indian sheep breeds, offering valuable insights for breeding and conservation strategies.

Areas for Improvement:

1. Link Between Genomic Regions and Local Adaptation: While the discussion mentions the environmental adaptations of the breeds, it could further elaborate on how these adaptations might have influenced the specific genomic regions under selection. For example, more direct comparisons to how the genetic variations might correlate with the climate or geographical features of the areas where these breeds are found could enhance the reader's understanding of the adaptive significance of these genes.
2. Consistency in Gene References: Some genes are mentioned repeatedly (e.g., CNTNAP5), but the context of how they were identified in different breeds could be more explicitly connected to avoid confusion. For instance, you might clarify how CNTNAP5's role in body growth is shared across breeds like CHA and GAR, yet each breed’s unique genomic context could influence the gene's effect.
3. Discussion on Statistical Methodology: The use of the DCMS method is discussed, but more detailed insight into why this method improves the resolution power of the genomic signals could help readers understand the methodological benefits. Briefly touching on the weaknesses of other statistical approaches and how the DCMS method overcomes them would strengthen the argument.
4. Gene Functionality and Mechanisms: While candidate genes are identified, more information on their molecular mechanisms or the processes through which they impact the phenotypic traits (e.g., how MTSS1 influences bacterial resistance) could help provide a clearer understanding of their biological relevance.
5. Comparison with Other Studies: The discussion of prior research, such as the studies from Ahmad et al. (2021) and Zhang et al. (2016), provides useful context. However, it would be helpful to briefly explain how the findings in the current study either align or contrast with these studies, reinforcing the study’s contribution to the field.

Bottom of Form

**Note: Authors are requested to revise the manuscript as per the comments or suggestions put into the manuscript file uploaded in the system, also expected to address the issues only under Discussion section in this Review Report.**
