# Supplementary material for: Composite selection signal analysis: Uncovering candidate genes and quantitative trait loci in Indian sheep breeds
Source: PLoS One. 2026 Mar 25;21(3):e0344299. doi: 10.1371/journal.pone.0344299 (PMC13016353; doi:10.1371/journal.pone.0344299)
Supplement: Attachment — Submitted filename: comments.docx [file pone.0344299.s003.docx]

**Minor Comments**

1. The manuscript, especially the discussion part is exceedingly long with redundancy in a few parts which is repeated in the results and discussion section.
2. Check the spelling “Chantangi” which is wrongly written.
3. Concise the discussion part and avoid redundancy.
