# Supplementary material for: Composite selection signal analysis: Uncovering candidate genes and quantitative trait loci in Indian sheep breeds
Source: PLoS One. 2026 Mar 25;21(3):e0344299. doi: 10.1371/journal.pone.0344299 (PMC13016353; doi:10.1371/journal.pone.0344299)
Supplement: Attachment — Submitted filename: Response_to_Journal_requirements.docx [file pone.0344299.s006.docx]

| **Manuscript Number: PONE-D-24-51732**  **Title: Composite Selection Signal Analysis: Uncovering Candidate Genes and Quantitative Trait Loci in Indian Sheep Breeds** | | |
| --- | --- | --- |
| **S.No.** | **Journal requirements** | **Response to Comments** |
| 01 | Please ensure that your manuscript meets PLOS ONE's style requirements, including those for file naming. The PLOS ONE style templates can be found at | **Thank you for pointing that out. Followed the instructions** |
| 02 | In the online submission form, you indicated that The genotypic data and other material related to this study are available with the corresponding author.  All PLOS journals now require all data underlying the findings described in their manuscript to be freely available to other researchers, either a. In a public repository, b. Within the manuscript itself, or c. Uploaded as supplementary information | **Thank you for bringing that issue to our attention. The data used in the study is public domain genotypic data. This was also mentioned in the manuscript.** |
| 03 | Ethics statement does not appear in the manuscript file:  Please include your full ethics statement in the ‘Methods’ section of your manuscript file. In your statement, please include the full name of the IRB or ethics committee who approved or waived your study, as well as whether or not you obtained informed written or verbal consent. If consent was waived for your study, please include this information in your statement as well. | **I am grateful for your bringing this matter to my attention.** **The research does not involve human or animal participants, and the data examined are publicly available. Therefore, no ethical approval or consent is necessary. This was also mentioned in the manuscript** |
| 04 | Please include a copy of Table 3 which you refer in your text | **Thank you for your valuable input.**  Included now in the manuscript. |
