# Supplementary material for: Composite selection signal analysis: Uncovering candidate genes and quantitative trait loci in Indian sheep breeds
Source: PLoS One. 2026 Mar 25;21(3):e0344299. doi: 10.1371/journal.pone.0344299 (PMC13016353; doi:10.1371/journal.pone.0344299)
Supplement: Attachment — Submitted filename: Response to Reviewers Comment_R3.docx [file pone.0344299.s007.docx]

| Manuscript Number: PONE-D-24-51732  Title: Composite Selection Signal Analysis: Uncovering Candidate Genes and Quantitative Trait Loci in Indian Sheep Breeds | | |
| --- | --- | --- |
| S.No. | Journal requirements | Response to Comments |
| 01. | Line number 83: Follow authors' instruction for citing reference(s); here needs reference number in parenthesis i.e. [10] | The authors thank you for pointing that out. We followed the authors' instructions in citing the references |
| 02. | Line number 93: Follow authors' instruction for citing reference(s); here needs reference numbers in parentheses i.e. [.....] and [.....] | Thank you for pointing that out. We followed the author’s instructions in citing the references. |
| 03 | Line number 103-104: Rephrase the sentence as “including few Indian sheep breeds.” | Rephrased the sentence as per the comment. |
| 04 | Line number 109: Give data as per the 20^th^ census | The sheep population, according to the 20th livestock census, is provided. |
| 05 | Line number 124: Follow authors' instruction for citing reference(s); here needs reference number in parenthesis i.e. [........] | Thank you for pointing that out. We followed the author’s instructions in citing the references. |
| 06 | Line number 156: Change De-Correlated Composite of Multiple Selection Signals to DCMS | Correction made according to the comment. |
| 07 | Line number 157: Change DCMS to ‘The DCMS’ | Revision is made as per the comment. |
| 08 | Line number 158: Follow authors' instruction for citing reference(s); here needs reference number in parenthesis i.e. [.......] | Thank you for pointing that out, sir. We followed the author’s instructions in citing the references. |
| 09 | Line number 160: Change FST to Fixation index (FST) | Revised the name of the statistic. |
| 10 | Line number 161: Change Tajima’s D to Tajima D index | Corrected the name of the statistic. |
| 11 | Line number 162: Shift into the space just before line 162. | The sentence at line 165 has been shifted. |
| 12 | Line number 178: format in subscript Fst | Formatted according to the comment. |
| 13 | Line number 196: Change the sentence to “These statistics”. | Revised the sentence according to the comment. |
| 14 | Line number 202: Delete the sentence “as outlined in” | Deleted the redundant sentence |
| 15 | Line number 205: use 'statistics' only because statistics are specific to sample estimates and parameters are specific to population estimates. | The word statistic is used instead of statistic parameters as suggested. |
| 16 | Line number 213: Cite reference number in parenthesis [.......] | Thank you for pointing that out, sir. We followed the author’s instructions in citing the references. |
| 17 | Line number 214: ??? |  |
| 18 | Line number 234: close the bracket | Changes were made as per the correction |
| 19 | Line number 251: crosscheck with the tabulated data | The correct values are now added after cross-check with the data in the table |
| 20 | Line number 282: QTL associated with the total.... | Changes made as per the suggestion. |
| 21 | Line number 321: Delete “has” | According to the correction, the word ‘has’ is deleted. |
| 22 | Line number 341: Delete “has”; delete and follow the simple past tense throughout the Results. | According to the correction, the word ‘has’ is deleted, and throughout the results section, the simple past tense is used. |
| 23 | Line number 378: check formatting | The changes have been made as suggested. |
| 24 | Line number 395: Cite reference number in parenthesis [.......] | Thank you for pointing that out, sir. We followed the author’s instructions in citing the references. |
| 25 | Line number 413: check formatting | The changes have been made as suggested. |
| 26 | Line number 490: Cite reference number in parenthesis [.......] | Thank you for pointing that out, sir. We followed the author’s instructions in citing the references. |
| 27 | Line number 497: Cite reference number in parenthesis [.......] | Thank you for pointing that out, sir. We followed the author’s instructions in citing the references. |
| 28 | Line number 502: Cite reference number in parenthesis [.......] | Thank you for pointing that out, sir. We followed the author’s instructions in citing the references. |
| 29 | Line number 514: Cite reference number in parenthesis [.......] | Thank you for pointing that out, sir. We followed the author’s instructions in citing the references. |
| 30 | Line number 604: Cite reference number in parenthesis [.......] |  |
| 31 | Line number 655: delete and shift to line 656. | The sentence at line 649 is removed and shifted to line number 656 as per the suggestion. |
| 32 | Line 657-658: Delete lines 651 and 652. | The lines at 651 and 652 are deleted as per the comment. |
