# Supplementary material for: Composite selection signal analysis: Uncovering candidate genes and quantitative trait loci in Indian sheep breeds
Source: PLoS One. 2026 Mar 25;21(3):e0344299. doi: 10.1371/journal.pone.0344299 (PMC13016353; doi:10.1371/journal.pone.0344299)
Supplement: Attachment — Submitted filename: Response_to_Reviewer_Comments_02.08.2025.docx [file pone.0344299.s008.docx]

| **Manuscript Number: PONE-D-24-51732R2**  **Title: Composite Selection Signal Analysis: Uncovering Candidate Genes and Quantitative Trait Loci in Indian Sheep Breeds** | | |
| --- | --- | --- |
| **S.No.** | **Reviewer’s comments** | **Response to Comments** |
| 01. | The manuscript mentions that DCMS combines five statistics including FST, H1, H12, Tajima’s D, and π, but does not elaborate on the rationale for selecting this specific combination of five indicators. Have other statistics (such as iHS and XP-EHH) been evaluated for their complementarity? How are the weights of each statistic determined in the composite calculation? | - Thank you, Sir, for pointing this out. The rationale for selecting this specific set of five indicators in the study arises from the need to assess genomic selection signatures through combining the selection signals obtained from the univariate statistics. We utilised FST to evaluate population differentiation, while h1 and h12 were employed for identifying haplotype-based signatures based on linkage disequilibrium. To capture signals related to the allele spectrum, we applied Tajima’s D and nucleotide diversity (π). The same is incorporated at the line numbers from 89-93. - Further, h12 is a comparable statistic to integrated Haplotype Score (iHS), and XP-EHH is also utilized for examining between-breed signatures only. This study is mainly based on within breed signatures. Hence, XP-EHH is not used in this study. - We obtained the q value (combined) by the following steps:  1. For each statistic within a given breed, genome-wide *p*-values based on fractional ranks (i.e. the *stat_to_pvalue* function) of the *Minotour* R package 2. Left tailed and right tailed tests were performed on the statistic, based on the distributions. 3. n x n correlation matrix among the statistic (minimum covariance determinant estimator of the multivariate location and scatter) using the *covNAMcd* function of the *Minotour* R package. Then, DCMS values are generated. 4. The DCMS values are fitted to a normal distribution using the MASS package in R. 5. Then *p-values* are calculated from the DCMS values using the *pnorm* function in the MASS package. 6. Finally, the *p-values* are converted to the *q-values* using the Benjamini and Hochberg method using the *p.adjust* R function. 7. The DCMS calculation employed in this study is based on the Yurchenko et al., 2018, which is further based on Lotterhos et al., 2017. 8. The DCMS values are mainly obtained by the nxn correlation matrix, which is obtained due to the covariance existed among the univariate statistics. 9. This method is more efficient and different from the method proposed by Randhawa et al., 2015. The latter study is based on the calculation of weights for the univariate statistic. |
| 02. | Line81, It is recommended to add literature citations, such as doi: 10.1038/s41467-020-16485-1, doi: 10.24272/j.issn.2095-8137.2022.124. | Thank you for your suggestion, the recommended references were added at line 80. |
| 03 | Line 130, why choose these three breeds for the research? | In this research, we focused on three indigenous sheep breeds—Changtangi, Garole, and Deccani—selected for their unique agroclimatic adaptations and production attributes. The Changtangi breed grows in the frigid, high-altitude areas of the Northern Himalayas, while Garole is adapted to hot and humid climates, and Deccani thrives in the arid, dry regions of India. This deliberate selection allows for the identification of quantitative trait loci (QTLs) linked to adaptation to thermal and cold stress across varying environmental conditions. Moreover, these breeds collectively encompass a diverse range of production traits: Changtangi is valued for its fine wool, Deccani is preferred for meat production, and Garole is noted for its prolific reproductive capabilities. Incorporating these breeds into our study significantly broadens the investigation into the genetic architecture that governs environmental adaptability and economically relevant traits in Indian sheep. |
| 04 | Line225, please remove the colon | Thank you for the comment. The colon was removed |
| 05 | It is recommended to adjust the format of the manuscript, such as Table 8. | Thank you for the comment. The format of the Table 8 was adjusted |
| 06 | Has the QTL identified in this study overlapped with those previously reported? | In this study, we observed a minimal overlap with the findings of the other studies such as Ahmad et al., 2021 and Sarvanan et al., 2021. |
