# Supplementary material for: Composite selection signal analysis: Uncovering candidate genes and quantitative trait loci in Indian sheep breeds
Source: PLoS One. 2026 Mar 25;21(3):e0344299. doi: 10.1371/journal.pone.0344299 (PMC13016353; doi:10.1371/journal.pone.0344299)
Supplement: Attachment — Submitted filename: Response to reviewers.docx [file pone.0344299.s009.docx]

*We sincerely thank the reviewer for their insightful comments and suggestions regarding our manuscript titled “****Composite Selection Signal Analysis: Uncovering Candidate Genes and Quantitative Trait Loci in Indian Sheep Breeds****” (Manuscript ID: PONE-D-24-51732R3). We have carefully revised the manuscript to address the concerns raised. Below are our detailed responses to each comment.*

**Comment 1:**

*The manuscript has undergone slight modifications. However, there are numerous grammatical errors. In several instances, a sentence is written in the past tense, while the following one is in the future tense. For consistency, it is recommended that the Methods section be written entirely in the past tense and maintain uniformity. A thorough review of the manuscript’s grammar is also recommended to avoid overall redundancy.*

**Response:**
We appreciate the reviewer’s careful reading and valuable feedback. In response, we have conducted a comprehensive grammatical review of the entire manuscript. Particular attention was paid to tense consistency, especially in the **Methods section**, which has now been revised to use the **past tense uniformly.** We have also removed redundant phrases and improved sentence structure throughout the manuscript to enhance clarity and readability.

**Reviewer Comment 2:**

*In line 395, the phrase “researchers could not differentiate” is unclear. It is indistinct whether the author is referring to findings from the previously published paper by Ahmed et al. or to their own data. Recommended to rephrase this sentence to clearly convey the intended meaning.*

**Response:**
Thank you for pointing out this ambiguity. We have revised the sentence to clarify the reference as suggested.

During the revision process, we became aware of the recent publication of a related article, “Comparative genomic insights into adaptation, selection signatures, and population dynamics in indigenous Indian sheep and foreign breeds” (Frontiers in Genetics, August 2025, Muthusamy et al.). We have cited this paper in our revised manuscript and clearly discussed both the similarities and the novel aspects of our work. Our study differs from theirs in analytical approach, breed focus, and resolution, as well as the discovery of several novel candidate genes and QTLs specific to Indian sheep breeds.

We hope these revisions adequately address the reviewer’s concerns. We are grateful for the opportunity to improve our manuscript and thank you for your continued consideration.

Destaw Worku, PhD

Corresponding Author
