# Supplementary material for: Composite selection signal analysis: Uncovering candidate genes and quantitative trait loci in Indian sheep breeds
Source: PLoS One. 2026 Mar 25;21(3):e0344299. doi: 10.1371/journal.pone.0344299 (PMC13016353; doi:10.1371/journal.pone.0344299)
Supplement: Attachment — Submitted filename: Response_to_Reviewer 5th_Comments.docx [file pone.0344299.s011.docx]

**Response to Reviewer Comments**

Manuscript Title: **Composite Selection Signal Analysis: Uncovering Candidate Genes and Quantitative Trait Loci in Indian Sheep Breeds**

Manuscript ID: **PONE-D-24-51732R4**

General Comment: Rewrite the entire document.

Response: **We fully appreciate this directive and have thoroughly revised the document to enhance professionalism, conciseness, and coherence. The narrative flows logically from evolutionary context to methods, results, implications, and conclusions, and the scientific tone has been elevated, avoiding casual phrasing**.

**Abstract:** Modify the statements such as “line 32: Natural and artificial selection shape the genomes of sheep”, “line 34: to preserve and enhance native genetic traits”, and “line 48: wide variety of characteristics”. The way of expression is not professional.

Response: **"line 32: Natural and artificial selection shape the genomes of sheep" has been modified to a more precise statement: "Selective pressures, both natural and artificial, have shaped the genomic architecture of domestic sheep**

**"line 34: to preserve and enhance native genetic traits" has been rephrased for greater clarity and specificity to: "to conserve and improve economically important traits in native breeds.**

**"line 48: wide variety of characteristics" has been updated to the professional terminology: "diverse phenotypic traits”.**

Thank you for pointing this out.

1. Give a description for each breed in the introduction section.

Response: **Thank you for your valuable feedback on the breed descriptions. We have added the paragraphs emphasizing the importance of each breed in the introduction section, as you suggested.**

2. Rewrite from line: 87-90.

Response: **We appreciate the reviewer's comments regarding our methodology. Lines 87-90 have been rewritten to enhance clarity and conciseness, address grammatical issues, and emphasize the methodological strengths**.

3. A quick transition disrupts the flow of idea between paragraphs 2 and 3.

Response: **Thank you for highlighting this issue. A few sentences have been incorporated as a bridge between the two paragraphs to ensure logical coherence.**

4. Line 118: “highly dense genotypes”. What does it mean? Please be sure to express the statement in professional terms.

Response: **We appreciate the comment, and the terms have been rewritten using professional language.**

5. Modify as 2.1 Ethics Statement and 2.2 Animal Selection and Genotyping.

Response: **Thank you for the suggestion; the paragraph numbering has been modified accordingly.**

6. Line 246-247: 3.3.1.

Response: **Thank you for pointing this out. The paragraph numbering has been revised accordingly.**

7. Line 282: production (9.38%). Which type of production?

Response: **Thank you for your comment. The sentence has been expanded to provide clearer context as you suggested.**

8. Table 1 is not under the appropriate position because it is a standard table for all breeds. You have already written the information of table 1 in text. No need of duplication.

Response: **Thank you for the feedback. Since the information is already included in the text, the table has been removed to avoid duplication**.

9. In the discussion section try to elaborate on the general implication of your study, any limitation, and agreeing or disagreeing with previous reports with citations. No need of repeating the methodology or introductory information.

Response: **We acknowledge the suggestion. A new paragraph emphasizing the general implications, limitations, and the stance on previous reports, supported by citations, has been incorporated at the conclusion of the discussion. Additionally, care has been taken to avoid redundancy in the methodology and introduction sections.**

10. Captions of figures should be self-explanatory. So, explain each graph in detail.

Response: **Thank you for your valuable feedback on the figure captions. Accordingly, we have provided detailed explanations for each graph in the revised manuscript.**

These revisions comprehensively resolve all points, resulting in a stronger manuscript. We thank Reviewer 5 for their thorough guidance and look forward to further feedback.

Sincerely,

The Authors
