# Supplementary material for: Composite selection signal analysis: Uncovering candidate genes and quantitative trait loci in Indian sheep breeds
Source: PLoS One. 2026 Mar 25;21(3):e0344299. doi: 10.1371/journal.pone.0344299 (PMC13016353; doi:10.1371/journal.pone.0344299)
Supplement: Attachment — Submitted filename: plos review_Dec 2025.docx [file pone.0344299.s010.docx]

**Comments:**

1. Rewrite the paragraph line no 198-204, heading 2.5.4. DCMS Estimation, with proper citation.
2. The author should revise the paragraph from lines no 344–349 with proper citation, as its current sentence is unclear and the intended meaning is difficult to understand.
3. Rewrite paragraphs of line no 353-357 with proper meaning. A clear and coherent rewrite is required.
4. Write the word ‘*p*-value’ and ‘*q*-value’ consistently throughout the manuscript.
5. The authors stated that the study identified two key candidate genes i.e. *TRMT12*, associated with environmental adaptation and *LRRC36*, associated with domestication in sheep. While Ji Yang et al. (2024) have been cited as evidence supporting the role of *LRRC36* in domestication, however, no reference has been provided to substantiate the association of *TRMT12* with environmental adaptation. The authors are required to cite an appropriate and relevant study demonstrating the significant association of *TRMT12* with environmental adaptation. Without such supporting literature, the current claim remains unsubstantiated.
6. It is unclear why reference [20] has been cited in line 219, immediately after the section, titled *2.6 Identification of Functional Genes and QTL Regions*. If information from this reference has been used, the authors should clearly rewrite the paragraph to properly reflect and justify the citation. If the reference is not relevant to the content, it should be removed.
7. Please write the full form of OAR when it is first mentioned in the manuscript and include the specific version of the OAR Rambouillet genome assembly.
8. Does OAR and CHR represent the same thing in table no 2? Does flank marker represent rsID? How does Flank marker id were obtained with rsID, as rsID of non human database are obsolete and discontinued? Author need to clarify what is meant by ‘flank marker’ in this context?
9. Please describe the significance of the p-value and q-value, specifically explaining their importance in interpreting the study’s analytical outcomes relevant to this study.
10. The manuscript requires extensive language editing and careful revision to ensure clarity and scientific accuracy, as much of the current writing is unclear and fuzzy in several sections. Additionally, the authors should remove irrelevant or nonessential content, as the paper is unnecessarily long and contains information that does not directly contribute to the study’s objectives.
