# Supplementary material for: Composite selection signal analysis: Uncovering candidate genes and quantitative trait loci in Indian sheep breeds
Source: PLoS One. 2026 Mar 25;21(3):e0344299. doi: 10.1371/journal.pone.0344299 (PMC13016353; doi:10.1371/journal.pone.0344299)
Supplement: Attachment — Submitted filename: Response_to_5th_Reviewers_Comments.docx [file pone.0344299.s012.docx]

**Response to Reviewer Comments**

Manuscript Title: **Composite Selection Signal Analysis: Uncovering Candidate Genes and Quantitative Trait Loci in Indian Sheep Breeds**

Manuscript ID: **PONE-D-24-51732R5**

**Comment 1: Check affiliation 5**

- **Response:** We thank the reviewer for pointing out this potential issue. We have carefully checked and verified the accuracy and correct formatting of affiliation 5.
- **Action Taken:**
  - **Location:** Affiliation list, affiliation 5.

**Comment 2: Remove the word “shows the” from each table caption**

- **Response:** We agree that this phrase is redundant and can be removed for conciseness. We have revised all table captions accordingly.
- **Action Taken:**
  - **Location:** Table captions for Tables 1, 2, 3, 4, 5, and 6.

**Comment 3: Since this study lacks experimental validation, clearly explain this limitation in the discussion and conclusion section.**

- **Response:** We acknowledge this important point regarding the scope and limitations of our study. We have explicitly addressed the absence of experimental validation in both the Discussion and Conclusion sections to provide a balanced perspective on our findings.
- **Action Taken:**
  - **Location:**
    - **Discussion Section:** Added a new paragraph towards the end of the main discussion.
    - **Conclusion Section:** Incorporated a brief statement.
